# An organophotocatalytic late-stage N–CH_3_ oxidation of trialkylamines to *N*-formamides with O_2_ in continuous flow†

**DOI:** 10.1039/d1sc05840a

**Published:** 2021-12-28

**Authors:** Mark John P. Mandigma, Jonas Žurauskas, Callum I. MacGregor, Lee J. Edwards, Ahmed Shahin, Ludwig d'Heureuse, Philip Yip, David J. S. Birch, Thomas Gruber, Jörg Heilmann, Matthew P. John, Joshua P. Barham

**Affiliations:** Fakultät für Chemie und Pharmazie, Universität Regensburg 93040 Regensburg Germany Joshua-Philip.Barham@chemie.uni-regensburg.de; GlaxoSmithKline Medicines Research Centre Gunnels Wood Road Stevenage SG1 2NY UK; Chemistry Department, Faculty of Science, Benha University 13518 Benha Egypt; Department of Physics, SUPA, University of Strathclyde 107 Rottenrow East Glasgow G4 0NG UK

## Abstract

We report an organophotocatalytic, N–CH_3_-selective oxidation of trialkylamines in continuous flow. Based on the 9,10-dicyanoanthracene (DCA) core, a new catalyst (DCAS) was designed with solubilizing groups for flow processing. This allowed O_2_ to be harnessed as a sustainable oxidant for late-stage photocatalytic N–CH_3_ oxidations of complex natural products and active pharmaceutical ingredients bearing functional groups not tolerated by previous methods. The organophotocatalytic gas–liquid flow process affords cleaner reactions than in batch mode, in short residence times of 13.5 min and productivities of up to 0.65 g per day. Spectroscopic and computational mechanistic studies showed that catalyst derivatization not only enhanced solubility of the new catalyst compared to poorly-soluble DCA, but profoundly diverted the photocatalytic mechanism from singlet electron transfer (SET) reductive quenching with amines toward energy transfer (E_n_T) with O_2_.

## Abstract 2

An N–CH_3_-selective trialkylamine oxidation to *N*-formamides is reported in continuous flow using gaseous O_2_. A novel, enhanced-solubility dicyanoanthracene organophotocatalyst switched the photochemical mechanism from electron to energy transfer.

## Graphical abstract

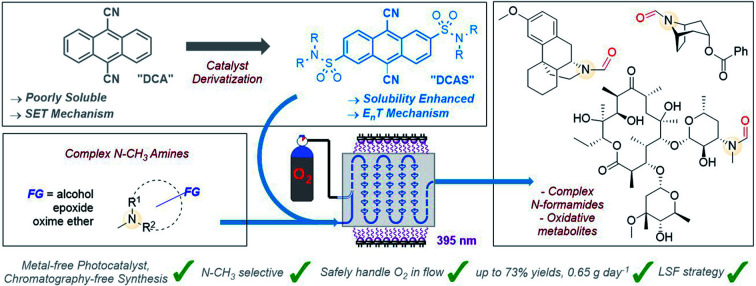

## Introduction

**Fig. 1:**
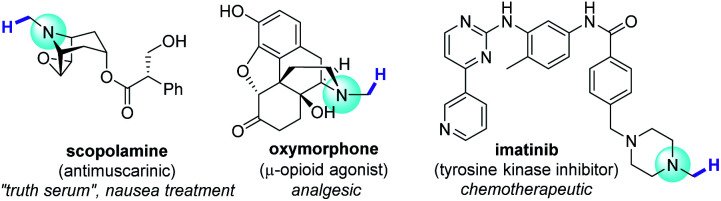
Bioactive trialkylamines and target sites for N–CH_3_ C–H functionalization.

A quintessential theme in medicinal chemistry is probing structure activity relationships. While strategies such as *de novo* and diversity-oriented synthesis are powerful tools to achieve this task, late-stage functionalization (LSF) has gained traction over the past decade as it offers a quicker route to access libraries of complex bioactive molecules from a defined core structure.^1,2^ Among the myriad of methods that are applied in LSF, C–H functionalization is undeniably an attractive and potent addition to a synthetic chemist's arsenal, given the ubiquity of C–H bonds in molecules.^1–3^ This umbrella term stretches over traditional transition metal catalysis to alkali and base-metal catalysis to organocatalysis and photocatalytically-enabled transformations. Recent examples demonstrate the value of C–H functionalization of simple and complex amides through ionic^4^ or radical^5^ mechanisms. Trialkylamines are especially important targets since they are well represented in the alkaloids, a family of potent bioactive molecules that has shaped the natural sciences.^6,7^ N–CH_3_ groups are attractive loci for C–H functionalization in pharmaceutical research, since incremental structural variations carry substantial pharmacological effects (Fig. 1), for example in bioactivities of opiates.^8^ However, C–H bonds α to N are relatively inert. Access to derivatives was historically carried out stepwise, leveraging the nucleophilicity of the N atom, usually requiring initial demethylations of trialkylamine N–CH_3_ groups to free N–H secondary amines for subsequent transformations.^9^ That is until the renaissance of single electron transfer (SET) redox methods, partly driven by photoredox catalysis, which have revolutionized practices in organic synthesis.^10^ This allowed direct C–H functionalizations α to N, of benzylic amines with nucleophiles^11^ and a few examples of aliphatic amines with electrophiles.^12^ A direct and highly N–CH_3_-selective LSF of trialkylamines was achieved using stoichiometric quantities of an SET-generated hydrogen atom transfer agent (DABCO˙^+^).^13^ Powerful catalytic or photocatalytic LSF strategies for complex trialkylamines have emerged, but remain scarce.^14^

**Fig. 2:**
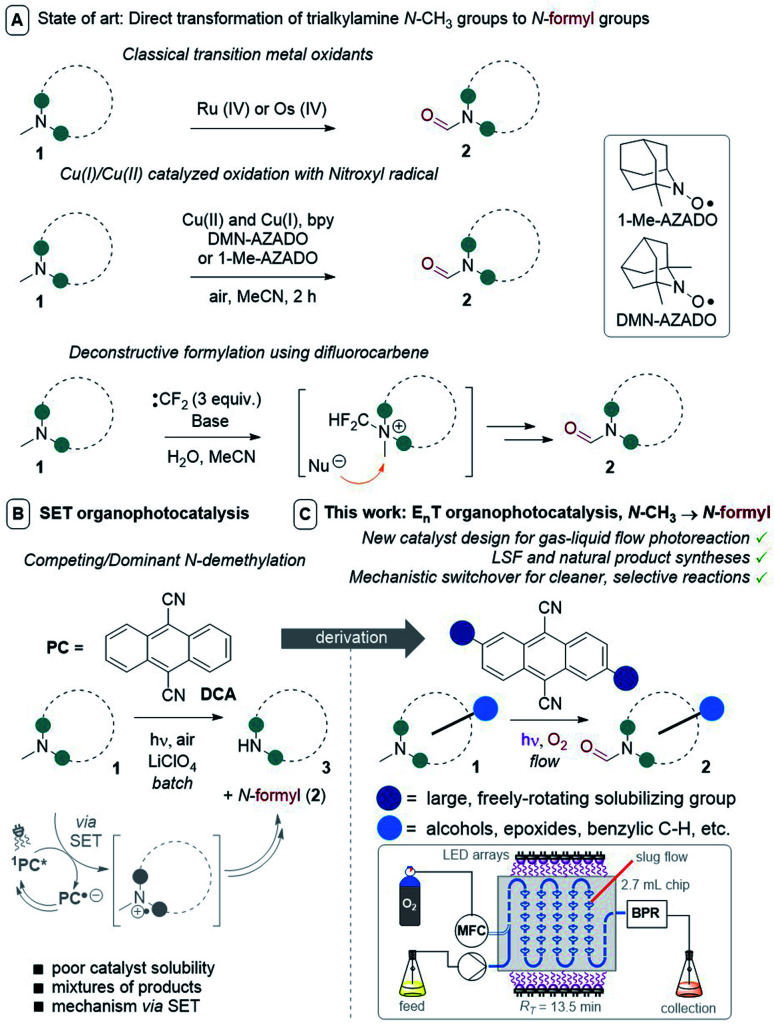
Strategies for selective N–CH_3_ to *N*-formyl oxidations. PC = photocatalyst, MFC = mass flow controller (O_2_), BPR = back pressure regulator, *R*_T_ = retention time.

Direct C–H oxidation of a trialkylamine's N–CH_3_ group to an *N*-formyl group is a worthy endeavour as *N*-formamide products (and mechanisms to access them) are relevant to oxidative metabolite research,^15^ are natural products,^6,16^ and serve as synthetic handles^17^ for further modifications including Barbier-type amidations,^18^ C–C cross-couplings,^19^ amino-carbonylations of alkenes or alkynes,^20^ and couplings with phenols^21^ or amines^22^ (affording carbamates or ureas, respectively). *N*-Formamides are classically accessed from trialkylamines by toxic Ru(iv) or Os(iv) oxidants (Fig. 2A).^23–27^

Recently, Yamaguchi and co-workers circumvented this *via* an elegant Cu(ii)/Cu(i) and nitroxyl radical catalyst system.^28^ Song and co-workers reported a transition metal (T.M.)-free deconstructive formylation reaction.^29^ The main drawbacks of such previous methods are (i) the incompatibility of redox-sensitive functionalities common to complex pharmaceuticals, hence limiting their application to relatively simple amines, and (ii) the expense of reagents (hindered nitroxyl radical catalysts^28*b*^ and excess difluorocarbene reagents) which are economically impractical for scale-up. We contemplated an alternative strategy using an organophotocatalyst, in order to avoid toxic, precious, unsustainable T.M.-based photocatalysts.^30^ Simultaneously, and inspired by numerous reports of gas–liquid photocatalytic flow processes,^31^ we envisaged that continuous flow would allow us to efficiently and safely handle O_2_ as a simple, abundant, sustainable terminal oxidant. The rapid uptake of continuous flow reactors in the synthesis of fine materials and pharmaceuticals is worth noting, as is their innovative marriage with visible light irradiation which drastically enhanced the efficiency, sustainability and safety of photochemical processes.^32^ Previous organophotocatalytic activations of trialkylamines using O_2_ were reported, but those instead targeted (i) *N*-demethylations of opiates and tropanoids,^33^ (ii) endocyclic C–H cyanations α to N^34^ or (iii) oxidations of benzylic amines.^35^ We were particularly drawn to 9,10-dicyanoanthracene (DCA) as used by Santamaria and co-workers (Fig. 2B). Using DCA as a potent photooxidizing catalyst (*E*_1/2_[^1^DCA*/DCA˙^−^] = +1.99 V *vs.* SCE),^30^ air (O_2_) as terminal oxidant, and an LiClO_4_ additive, they reported variable amounts of *N*-formyl products (2) in batch, however *N*-demethylation (nor-amines (3)) competed or dominated reactions.^36,37^ Herein, we report a late-stage organophotocatalytic oxidation of N–CH_3_ groups that selectively delivers *N*-formyl compounds (2). Our method leverages mild conditions and continuous flow processing to handle O_2_ safely as a terminal oxidant (Fig. 2C). Key to the aforementioned achievements was the design of a novel dicyanoanthracene catalyst that not only enhanced solubility for flow processing, but switched the excited state mechanism from single electron transfer with amines toward energy transfer with O_2_.

## Results and discussion

### Photocatalyst and process design

**Fig. 3:**
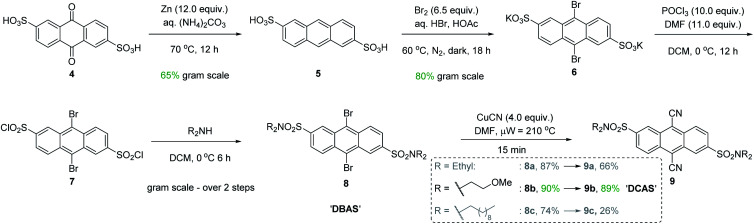
Chromatography-free gram scale synthesis of DCAS photocatalyst.

At the onset, our attempts to use DCA using modified reaction conditions of Santamaria in batch (and in flow) were severely obstructed by its poor solubility in MeCN (*i.e.*, turbidity and sedimentation were observed). The suspended, undissolved photocatalyst was detrimental to photochemistry due to hindering light penetration of the reaction. Furthermore, in continuous flow this often led to flow channel blockages and longer reaction times (for details, see ESI† file). Thus, design of a catalyst with enhanced solubility was required (Fig. 3).

Intuitively, introduction of polar substituents improves solubility of compounds in polar aprotic solvents. Nitro- and sulfonic acid- groups are good choices for polyaromatic compounds as the synthetic process to access them is straightforward. Glöcklhofer and co-workers reported the synthesis of a dinitro derivative of DCA with improved solubility.^38^ On the other hand, sulfonic acids carry the advantage of further derivatization *via* their sulfonyl chlorides. Inspired by intermediates reported in the synthesis of a water-soluble DCA analogue,^39^ we began our catalyst synthesis (Fig. 3). Anthraquinone-2,6-disulfonic acid 4, commercially supplied or easily synthesized from cheap anthraquinone,^40^ was reduced by activated Zn in aq. (NH_4_)_2_CO_3_ to afford anthracene-2,6-disulfonic acid 5 in good (65%) yield after acidic workup and recrystallisation from aq. KCl. Electrophilic bromination of the central ring of 5 gave 6 in high (80%) yield. At this stage, our synthesis deviated from the literature cyanation which digested the crude product (containing CuCN) in conc. HNO_3_ and liberated toxic HCN gas. However, both Rosenmund von-Braun and Pd-catalysed cyanations failed to cyanate 6 due to its poor solubility in organic solvents. Tohnai and co-workers had reported that the derivatization of anthraquinone disulfonic acids (ADS) as their organic ammonium salts (*i.e.*, *n*-heptyl and *n*-pentyl) prevented π-stacking interactions of ADS as observed by crystallography.^41,42^ Instead of ammonium salts which would hinder characterization and reaction workup, we achieved this covalently with sulfonamides.

Therefore, 6 was derivatized to increase its solubility in polar aprotic organic solvents and to increase prospects for successful cyanation. Chlorination of 6 with POCl_3_ and subsequent trapping of 7 with secondary amines of various chain lengths gave 9,10-dibromoanthracene-2,6-disulfonamides (DBAS) 8a, 8b and 8c in 87, 90 and 74% yields, respectively. Pleasingly, Rosenmund von-Braun cyanations under microwave-assisted (15 min) or thermal (see ESI†) heating afforded 9,10-dicyanoanthracene-2,6-disulfonamides 9a, 9b (DCAS) and 9c as ‘brilliant yellow’ solids in 66%, 89% and 26% yields, respectively. We note that our entire synthesis to 9 is carried out on gram scale, with straightforward purification *via* recrystallisation instead of chromatography. Photocatalyst 9b (henceforth coined ‘DCAS’) was progressed to evaluation in reactions since it: (i) displayed the highest solubility in MeCN (1.900 ± 0.100 mg mL^−1^*vs.* 0.340 ± 0.006 mg mL^−1^ for DCA) consistent with its calculated physical property values (which showed that it was the least lipophilic and had the highest topological polar surface area, see ESI†),^43^ and (ii) was obtained in the highest overall yield (42% over 5 steps).

### Studies using a homogeneous liquid flow photoreactor

Next, DCAS was tested under some initial photocatalytic flow conditions (Table 1) in a commercial tubular coil continuous flow photoreactor (Vapourtec Ltd R-series/UV-150). Using 1a (12 mM) as our substrate and 5 mol% of DCAS at rt, a maximum yield of 25% for 2a (with 4 : 1 of 2a : 3a selectivity) was obtained under recycling conditions (90 min) no matter whether dry air, O_2_, or (1 : 1) N_2_/O_2_ were used (entry 2). The absence of catalyst (entry 2) or O_2_ led to no reaction. We found out that in the absence of LiClO_4_, single pass conditions gave a similar yield (25%) and with much improved selectivity for 2a (entry 4, 3a was not detected). When the temperature was increased to 40 °C, the yield improved to 40% (entry 5). Under similar conditions but employing DCA as catalyst afforded 2a in 15% yield, confirming superiority of DCAS under flow conditions. A batch reaction mimicking Santamaria and co-workers' condition (entry 8) afforded a complex reaction mixture (see ESI†). Our previously reported batch anaerobic conditions for SET oxidation of *N*-alkyl tetrahydroisoquinolines with [Ru(bpy)_3_]^2+^ photocatalysis^11*b*^ in batch (entry 7) gave no reaction, and when the more potent photooxidizing SET catalyst [Ru(bpz)_3_]^2+^ was used only traces of 3a were observed. As such and due to cost of the catalysts, we did not examine these any further in flow.

**Table 1:** Initial photocatalyst screening for N–CH_3_ to *N*-formyl oxidation of a trialkylamine

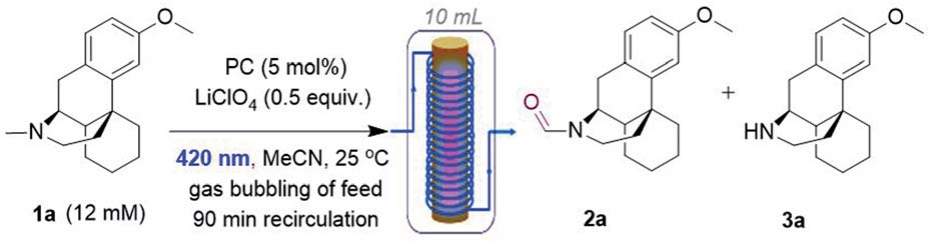				
Entry	PC	Deviation from condition	2a : 3a^a^	% Yield of 2a^a^
1	DCAS	Dry air, O_2_ or N_2_/O_2_ (1 : 1)	4 : 1	25
2	—	Dry air	n.r.	n.d.
3	DCAS	N_2_	n.r.	n.d.
4^b^	DCAS	O_2_, *R*_T_ = 20 min, no LiClO_4_	>30 : 1	25
5^b^^,^^c^	DCAS	O_2_, *R*_T_ = 20 min, no LiClO_4_	>30 : 1	40
6^b^^,^^c^	DCA	O_2_, *R*_T_ = 20 min, no LiClO_4_	>30 : 1	15
7^d^	DCA	Batch, *hν* > 420 nm	—	c.r.m.
8^d^	[Ru]^e^	Batch, 459 nm	—	Traces 3a

a Selectivity and yields determined by ^1^H NMR of the crude reaction mixture using 1,3,5-trimethoxybenzene (TMB) as internal standard.

b Single pass.

c *T* = 40 °C.

d For exact details of conditions including terminal oxidants attempted, see ESI.

e 1 mol% of Ru(bpy)_3_Cl_2_ or Ru(bpz)_3_(PF_6_)_2_. PC = photocatalyst, n.r. = no reaction, n.d. = not detected, c.r.m. = complex reaction mixture.

When under N_2_ protection (entry 3), a purple coloration in the post-reactor flowing reaction mixture was observed (see ESI†) which hinted at formation of DCAS˙^−^. We note that the related parent structure DCA˙^−^ is well-known to be purple in color.^44^ When the purple post-reactor reaction mixture was collected and exposed to air, immediate discoloration back to yellow was observed. From these observations, we had initially assumed a reductive SET quenching of ^1^DCAS* by the amine, as originally proposed by Santamaria and co-workers (Fig. 2B).^36,37^ However, this was later refuted (see the Mechanistic studies for details).

Based on this mechanistic assumption, we reasoned that formation of 2a reached its upper limit due to limiting oxygen solubility at ambient conditions in the tubular reactor, preventing catalyst turnover. The solubility of O_2_ in an O_2_-saturated solution of MeCN is 8.1 mM,^45^ and considering the theoretical requirement of 2 equiv. O_2_ to remove 2 electrons from the trialkylamine, mass transfer limits full conversion of a reaction mixture containing 12.0 mM trialkylamine (later in the revised mechanism, we find that [O_2_] is still a limiting factor for the reaction yield).

### Studies using a gas–liquid flow photoreactor

Considering the abovementioned observations, we opted for a photoreactor designed for biphasic gas–liquid reactions. A commercial microfluidic continuous flow photoreactor (Corning Lab Photoreactor©) designed for excellent mixing *via* turbulent slug flow allowed us to safely operate up to 60 °C and 8 bar backpressures. The hazard of the flammable reaction mixture was safely contained by the thermal isolation of the flow path and the small volume of reaction mixture (2.7 mL) at any given time. A summary of reaction condition optimization is shown in Table 2 (see ESI† for full optimization). Transferring conditions from the previous tubular reactor (Table 1, entry 5), 2a was afforded in 22% yield (Table 2, entry 1), as expected since the decreased yield exactly consists with (is proportional to) the decreased residence time (*R*_T_). However, the yield almost doubled when 395 nm LEDs were used (entry 2), which accorded with a higher extinction coefficient of DCAS's UV-vis band at *ca.* 395 nm compared to its 420 nm band (*vide infra*). At 24 mM 1a and double the residence time, the yield increased to 44% (entry 7). At 48 mM of 1a the yield decreased to 24% (entry 8), presumably again due to the limiting [O_2_]. At *T* = 60 °C and 24 mM 1a, the yield of 2a marginally improved to 46% (entry 9). The inherent back pressure on the flow by the microfluidic module was sufficient to ensure precise, reproducible, low flow rates (down to 0.1 mL min^−1^) up to 60 °C. To our delight, tropine 1b afforded 2b in 60% under reaction conditions at *T* = 40 °C and *R*_T_ = 27 min (entry 10) despite its free 2° alcohol typically prone to oxidation under similar oxidative conditions.^23–28,46^ Decreasing catalyst loading decreased the yield (entries 11 and 12). Like the case of substrate 1a, a marginal increase of yield to 61% occurred at 60 °C (entry 13). At this stage, we explored the effect of a back pressure (8 bar) to evaluate higher O_2_ solubility (entries 14–16). At lower backpressures, the flow was heterogeneous slug flow but at 7–8 bar, homogenous flow was observed indicating full solubilization of O_2_ and higher dissolved [O_2_]. At 7–8 bar, doubling the concentration to 48 mM or using a residence time as short as *R*_T_ = 6.8 min negatively impacted the yield of 2b (entries 14 and 15), but we found that yield (61%) was preserved at *R*_T_ = 13.5 min (entry 16 *vs.* 13). This doubled productivity of 2b to 0.65 g per day which was the upper limit of the gas–liquid organophotocatalytic flow reaction in this system.

**Table 2:** Reaction optimization in a gas–liquid flow reactor^a^^b^

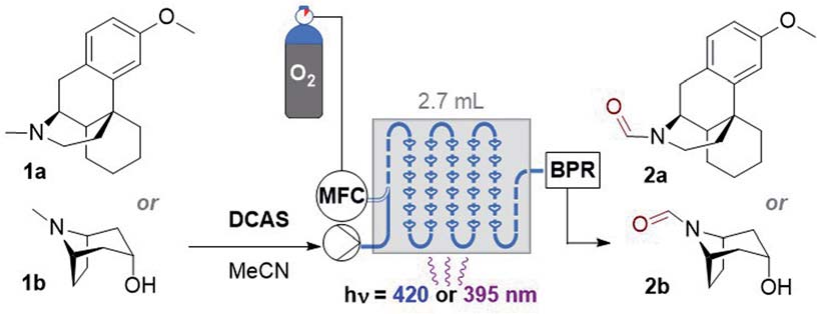						
Entry	Amine	Conc. (mM)	*R*_T_ c (min)	*hν* (nm)	*T* (°C)	% Yield^d^
1	1a	12	13.5	420	40	22 (2a)
2	1a	12	13.5	395	40	40 (2a)
3	1a	12	5.4	395	40	12 (2a)
4	1a	12	∼1.0	395	40	Trace (2a)
5	1a	12	∼1.0	None	40	n.r.
6	1a	24	13.5	395	40	40 (2a)
7	1a	24	27.0	395	40	44 (2a)
8	1a	48	27.0	395	40	24 (2a)
9^e^	1a	24	27.0	395	60	46 (2a)
10	1b	24	27.0	395	40	60 (2b)
11^f^	1b	24	27.0	395	40	53 (2b)
12^g^	1b	24	27.0	395	40	42 (2b)
13^e^	1b	24	27.0	395	60	61 (2b)
14^e^^,^^h^	1b	48	13.5	395	60	48 (2b)
15^e^^,^^h^	1b	24	6.8	395	60	31 (2b)
16^e^^,^^h^	1b	24	13.5	395	60	61 (2b)

a Unless otherwise stated, reaction conditions: DCAS (5 mol%), O_2_ (ambient pressure), at 40 °C.

b _T_ = 25 °C.

c *R*_T_ = residence time = (2.7 mL)/(flow rate).

d Yield determined by ^1^H NMR using 1,3,5-TMB as internal standard.

e *T* = 60 °C.

f DCAS (3 mol%).

g DCAS (1 mol%).

h At 7–8 bar back pressure. MFC = mass flow controller (O_2_), BPR = back pressure regulator, n.r. = no reaction.

Next, we tested the scope of the reaction (Table 3). Since isolations of polar formamides were oftentimes challenging due to the *N*-formyl group being a weak chromophore, the following discussion deems ^1^H NMR yields more representative of reaction efficiency. Compounds 2c (59%) and 2d (67%) were obtained from natural products tropane and (free alcohol-bearing) atropine. Even scopolamine, which has a free alcohol, an ester, and an epoxide, afforded 2e in 62% yield with no nor-scopolamine detected, albeit requiring 2 passes through the reactor (total *R*_T_ = 27 min). This contrasts with Santamaria and co-workers’ conditions using DCA and without LiClO_4_, which afforded a 1 : 1 mixture of 2e : nor-scopolamine.^36,37^ Compared to 2b, the yield of 2f was lower (34%) presumably due to the presence of the Si protecting group known to stabilize radicals and quench excited photosensitizers *via* different pathways.^47^ Benzoyl-containing compound 2g was afforded in good (73%) yield. Electron-poor (–CF_3_) and electron-rich (–OMe) substituents on the benzoyl group were tolerated equally, affording 2h (56%) and 2i (58%) respectively. We note both 2g and 2i are natural products; novel tropanoid compound 2g was recently isolated from *Pellacalyx saccardianus* and our method corroborated its proposed structure.^48^ Compound 2g (confoline) was isolated from *Convolvulus subhirsutus* and our method accessed it from convolvamine in a single step (in the literature, semi-synthesis of 2i was achieved by formylation of nor-convolvamine, hence a demethylation step from convolvamine culminates in a two-step process).^17^ Compounds 2j to 2o were obtained from piperazines as common API fragments (such as those present in sildenafil and danofloxaxin).^49^ Despite having 3 possible sites for functionalization (one exocyclic N–CH_3_ and two endocyclic N–CH_2_–R sites), selective oxidation at the N–CH_3_ (*exo*- : *endo*- = 5.7 : 1 for 2j, 3.4 : 1 for 2k, 3.7 : 1 for 2l, and 6.5 : 1 for 2p, see ESI†) was apparent, affording *N*-formyl compounds in respectable yields. Despite the modest yields of products 2l (55%), 2m (21%), and 2n (30%) (as well as 2h), we were surprised by the tolerance of halogen-bearing substrates under the reaction conditions. Especially, given the aforementioned putative presence of DCAS˙^−^*via* reductive quenching of ^1^DCAS* by trialkylamines (well known for DCA's case)^50,51^ and given that photoexcited radical anions are known to reductively cleave aryl halides and other strong bonds.^50–53^ C–F bonds and N–Ts groups are also prone to reductive cleavage under reductive photocatalysis^54^ or by photoexcited super electron donors.^55^

**Table 3:** Scope of organophotocatalytic flow N–CH_3_ to *N*-formyl oxidation

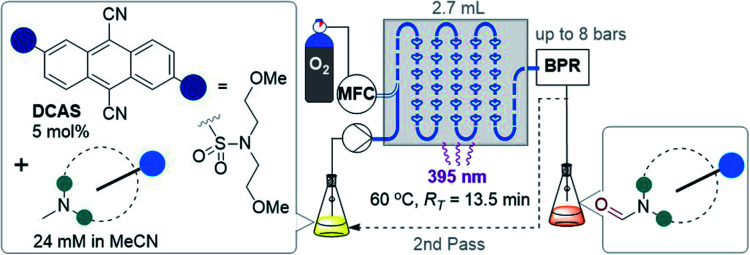
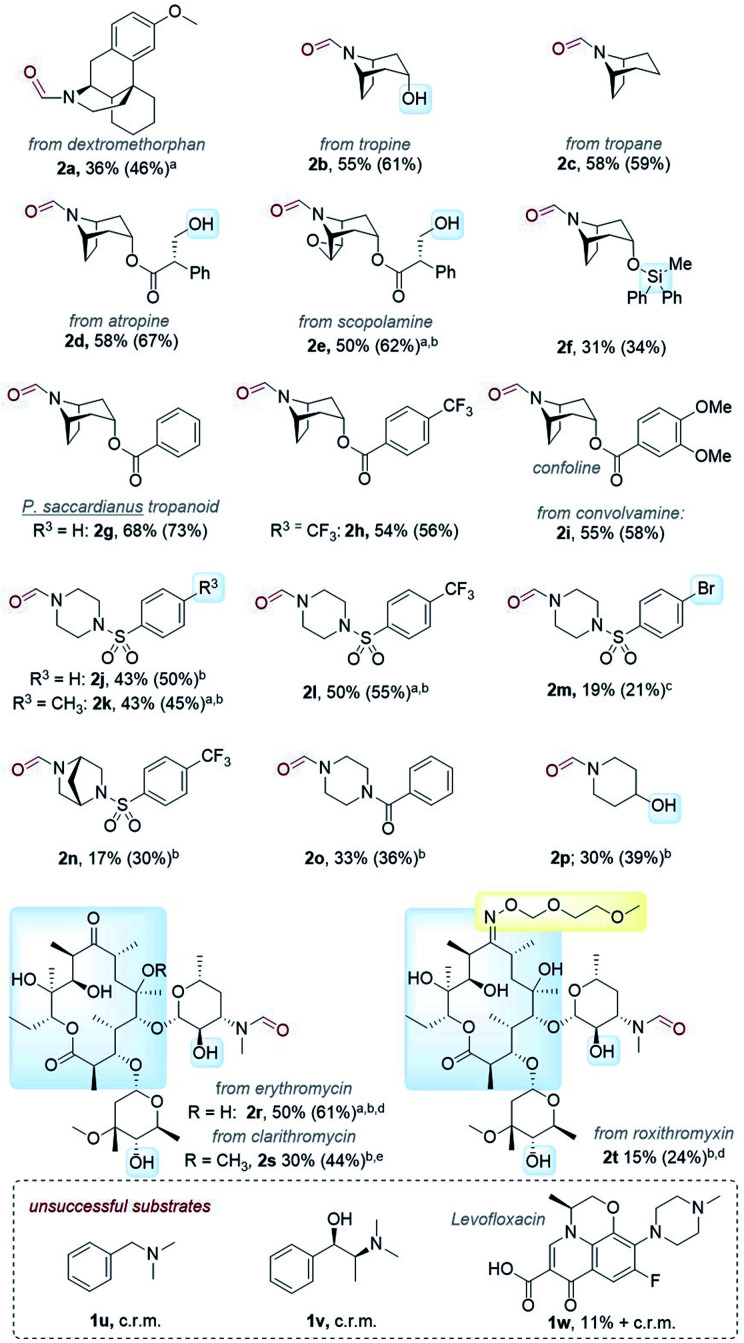

a *R*_T_ = 27 min, O_2_ (ambient pressure).

b 2 passes.

c 12 h recycling.

d 12 mM.

e 6 mM. Yields in parenthesis determined by ^1^H NMR of the reaction mixture using 1,3,5-TMB as internal standard. c.r.m. = complex reaction mixture.

A simple piperidine 2p (39%) was also tolerated. Our success with 2b, 2d, 2e, and 2p whose precursors bore free alcohol groups encouraged us to explore more complex molecules. Gratifyingly, conditions were successfully applied to macrolide antibiotics with dense functionalities (free alcohols, an oxime ether, and a ketone). Erythromycin, clarithromycin and roxithromycin afforded 2r, 2s, and 2t in 61%, 44%, and 24% yields, respectively. However, benzylic amines and trialkylamines containing benzylic alcohols or free carboxylic acids such as 1u, 1v and 1w, were unsuccessful. Benzaldehyde formation (C–N cleavage, possibly *via* endocyclic iminium ion formation and then hydrolysis) and intractable complex reaction mixtures were observed for these substrates.

### Mechanistic studies

**Fig. 4:**
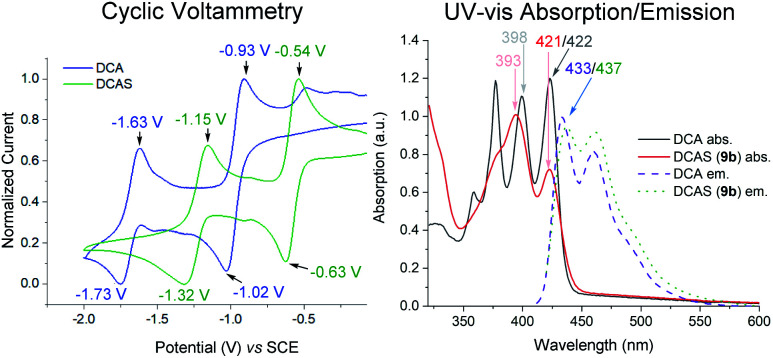
Left: cyclic voltammetry of catalysts. Conditions: 0.01 M DCA/DCAS in 0.1 M ^*n*^Bu_4_NPF_6_/MeCN, scan rate 50 mV s^−1^. Right: UV-vis and emission spectra.

**Fig. 5:**
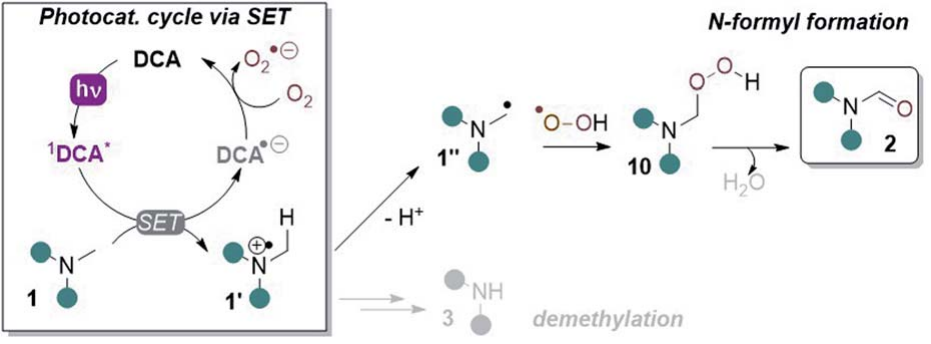
SET reductive quenching mechanism of DCA proposed by Santamaria and co-workers leading to demethylation and *N*-formyl products.

Cyclic voltammetry (CV) revealed DCAS (*E*_1/2_[DCAS/DCAS˙^−^] = −0.59 V *vs.* SCE) is substantially easier to reduce than DCA (*E*_1/2_[DCA/DCA˙^−^] = −0.98 V *vs.* SCE), due to the electron-withdrawing sulfonamide groups at the 2,6-positions (Fig. 4, left). UV-vis absorption and emission spectra were measured for DCA and DCAS (Fig. 4, right) and their comparison revealed that the 2,6-sulfonamides hardly affect the absorptive or emissive profiles of the dicyanoanthracene core. In both cases, overlap of the longest wavelength absorption band (*λ*_max_ = 422 nm) and shortest wavelength emission band (*λ*_max_ = 435 nm) allows to approximate *E*^0–0^ for the singlet excited state (≈2.90 eV). Taking this value together with measured redox potentials, the photocatalyst excited state oxidation potentials were approximated by a derivative of the Rehm–Weller equation.^56^^1^DCAS* (*E*_1/2_[^1^DCAS*/DCAS˙^−^] = +2.31 V *vs.* SCE) is a notably more potent photooxidant than ^1^DCA* (*E*_1/2_[^1^DCA*/DCA˙^−^] = +1.93 V *vs.* SCE). Our initial hypothesis thus continued to align with the SET mechanism proposed by Santamaria (Fig. 5).^36,37^ In this premise, ^1^DCAS* was assumed to behave like ^1^DCA* which underwent reductive quenching by trialkylamine 1, and oxidation of DCAS˙^−^ by O_2_ regenerated DCAS. Deprotonation of radical cation 1′ and radical combination of the α-amino radical and superoxide would ultimately afford *N*-formyl product 2. We note SET reactions were also proposed as the main pathways for trialkylamine activations by thiazine and fluorescein organophotocatalysts, either *via* oxidation to *N*-oxides or *via N*-demethylations.^57^

**Fig. 6:**
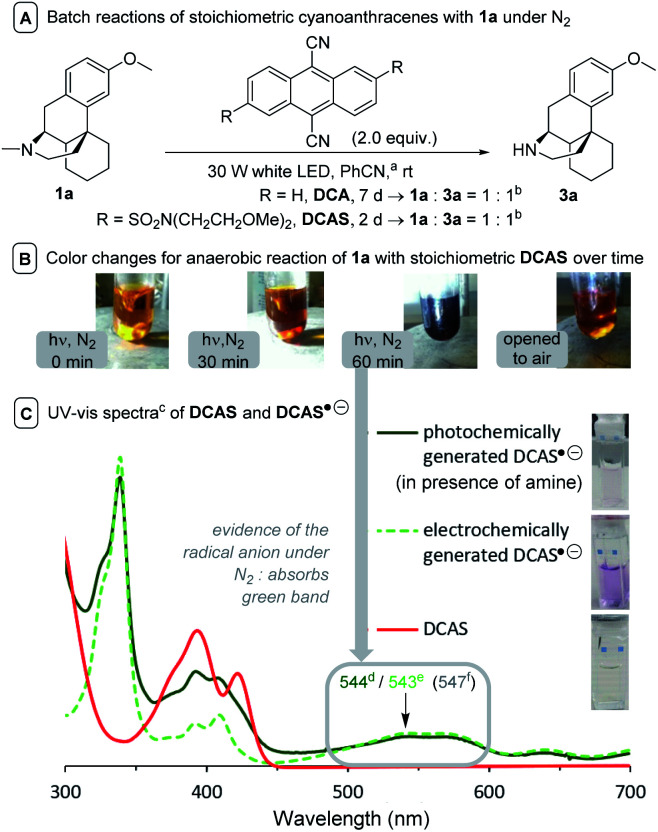
Experiments suggesting the generation of cyanoanthracene radical anions. (A) ^*a*^Due to stoichiometric PC loadings, PhCN was chosen as solvent for improved solubility. ^*b*^In the absence of additional base, 1a deprotonates 1a˙^+^ to afford 1a′′, meaning the reaction fundamentally could never exceed 50% conversion. (B) Observed color changes. (C) ^*c*^In MeCN, *λ*_max_ at the visible region when ^*d*^generated by photochemical reductive quenching of ^1^DCAS* with a trialkylamine; ^*e*^generated electrochemically; or ^*f*^calculated using TD-DFT at CAM-B3LYP/6-31++g(2d,p), CPCM(MeCN) level of theory (for details, see ESI†).

To test this initial hypothesis, two control batch experiments with stoichiometric (2.0 equiv.) DCA and DCAS were conducted under strict N_2_ protection in PhCN solvent to promote solubility. Both afforded clean conversion of 1a to a 1 : 1 mixture of 1a : 3a (Fig. 6A), although DCA's reaction required >3.5× reaction time due to poorer solubility. Upon irradiation, the reaction mixtures changed from a pale yellow color to dark purple (Fig. 6B). Removal the light and exposing to air, the colors of reaction solutions quickly reverted to yellow (consistent with aforementioned observations of the flow reaction under N_2_). The UV-vis spectra of DCA˙^−^ is well studied in the literature,^51,53^ and it is known to be purple in color.^44^ We confirmed the presence of DCAS˙^−^ spectroscopically by matching the spectra of a sample of DCAS treated by cathodic electrolysis to that treated photochemically in the presence of a trialkylamine reductive quencher (see ESI† for details). Both gave a new, broad absorption spectrum at the visible-green region (*λ*_max_ ≈ 544 nm, Fig. 6C), thus an apparent purple color.

**Fig. 7:**
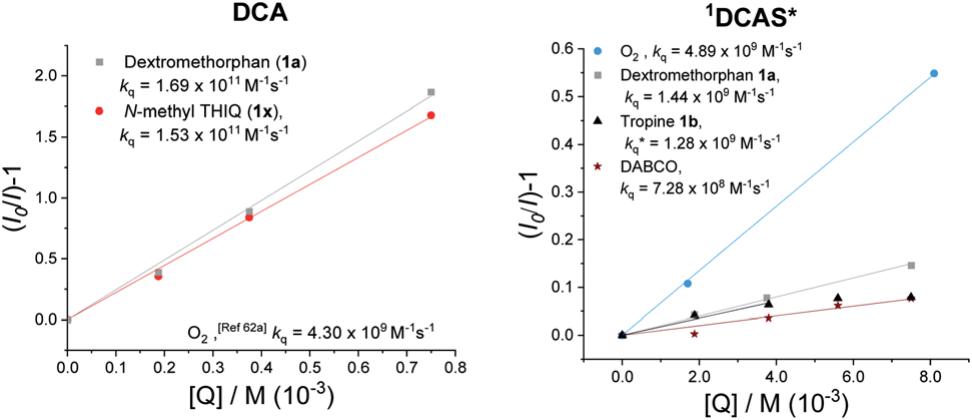
Stern–Volmer quenching experiments of DCA (right) and DCAS (left) with various amine quenchers (under N_2_) or O_2_.

A Time-Dependent Density Functional Theory (TD-DFT) calculation of the UV-vis transitions of DCAS˙^−^consisted with this green absorption peak (*λ*_max_ = 547 nm). The detection of these radical anions together with *N*-demethylation reaction confirmed that the SET oxidation of 1 to 1′ by the organophotocatalysts was possible, at least under anaerobic conditions. Considering the preceding discussion supportive of an SET mechanism in Fig. 5 and catalysts' redox potentials, one would expect ^1^DCAS* to undergo more rapid fluorescence quenching than ^1^DCA* by trialkylamines. Very surprisingly, the opposite was clearly true (Fig. 7). The Stern–Volmer rate constant for quenching of ^1^DCAS* by 1a (*k*_q_ = 1.44 × 10^9^ M^−1^ s^−1^) was two orders of magnitude smaller than that of ^1^DCA* (*k*_q_ = 1.69 × 10^11^ M^−1^ s^−1^).^58,59^ Presumably, either (i) the 2-methoxyethyl groups of DCAS affects the kinetics of bimolecular quenching by sterically obstructing the approach of trialkylamine, or (ii) aggregation of DCA accelerates reductive quenching by trialkylamines^60^ where DCAS exhibits a different kind of aggregation in solution.^61^

To probe the mechanistic role of the structural changes on the catalyst, and to rationalize the unexpected trend between the order of redox potentials of ^1^DCA* and ^1^DCAS* *vs.* their fluorescence quenching rates, DFT and TD-DFT calculations were performed. The activation energies (Δ*G*^‡^_SET_) for the photoinduced single electron transfer (SET) from 1b to photoexcited dicyanoanthracenes were determined using Marcus theory (Table 4). Aside from free energy (Δ*G*_SET_), another key parameter of Marcus theory is the reorganization energy (*λ*) which accounts for the properties of the solvent, the size of, and the distance between reacting species. The calculated vertical excitation energies (Δ*G*_Ex_ ≈ 3.3 eV) were close to *E*^0–0^ value obtained from optical spectroscopy (*vide supra*). As expected from the experimentally-determined photocatalyst redox potentials, SET of 1b with ^1^DCAS* is 1.3× more exergonic (Δ*G*_SET_ = −49.9 kcal mol^−1^) than with ^1^DCA* (Δ*G*_SET_ = −37.3 kcal mol^−1^). However, the kinetic barrier is notably (3×) higher for ^1^DCAS* (Δ*G*^‡^_SET_ = 13.7 kcal mol^−1^) than ^1^DCA* (Δ*G*^‡^_SET_ = 4.4 kcal mol^−1^). This agreed with the relatively slower fluorescence quenching of ^1^DCAS* by trialkylamines. However, this is juxtaposed with the greater synthetic efficiency of the reaction catalysed by DCAS compared to DCA. Taken together, these results show that although SET between excited cyanoanthracenes and trialkylamines can occur under anaerobic conditions, an alternative mechanism must operate for DCAS under aerobic conditions in order for it to deliver higher synthetic efficiencies.

**Table 4:** Calculated kinetics and thermodynamics for photoinduced SET of ^1^PC*s with 1b^a^

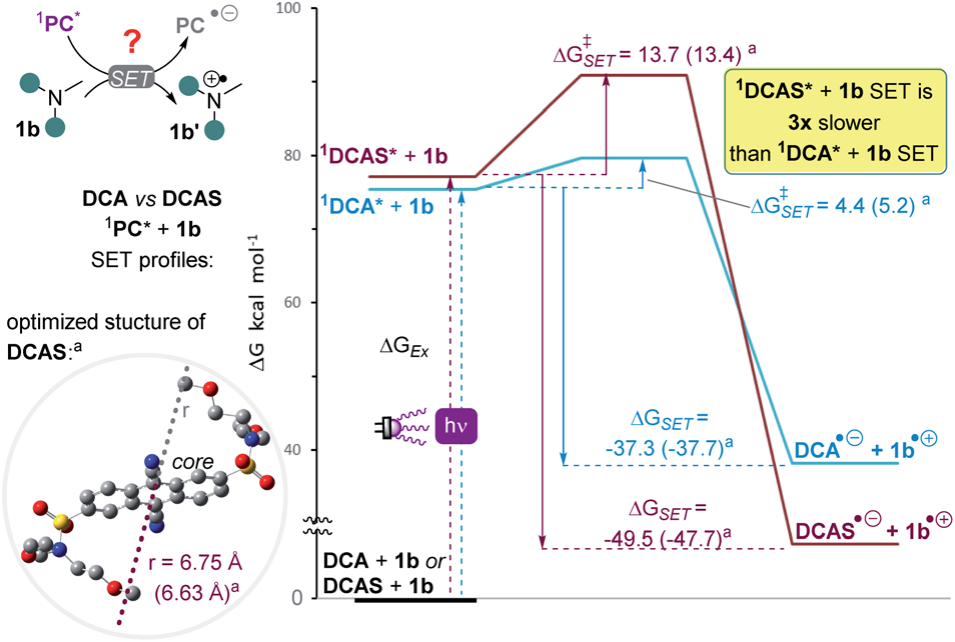				
^1^PC*	*k*_q_ b	Δ*G*_Ex_^c^	Δ*G*_SET_^d^	Δ*G*^‡^_SET_^e^
DCA	1.69 × 10^11^	75.4 (75.9)	−37.3 (−37.7)	4.4 (5.2)
DCAS	1.44 × 10^9^	77.2 (76.8)	−49.9 (−47.7)	13.7 (13.4)
DCAS/DCA	∼0.01	∼1 (∼1)	1.3 (1.3)	3.1 (2.6)

a Geometry optimization, molecular radius (*r*) and free energies calculated using DFT (ground state) or TD-DFT (excited state) at CAM-B3LYP (or ωB97X-D in parentheses)/6-31++g(2d,p), CPCM(acetonitrile) level of theory (see ESI).

b From Stern–Volmer analyses (Fig. 7), in M^−1^ s^−1^.

c Vertical excitation energy.

d Photoinduced-SET free energy.

e Photoinduced-SET activation energy. All free energy units in kcal mol^−1^. PC = photocatalyst. For further details, see ESI.

Elsewhere, ^1^DCA* is also known as an efficient singlet oxygen sensitizer (*k*_q_ = 4.3 × 10^9^ M^−1^ s^−1^) *via* a photosensitized energy transfer (E_n_T) mechanism.^62^ The high reported quantum yield (reaching 2.0) supports the generation of 2× ^1^O_2_ molecules per 1× ^1^DCA*.^62^ This quenching rate constant of ^1^DCA* by E_n_T is more than double that of the reductive SET quenching of ^1^DCAS* by trialkylamines. Thus, as [O_2_] increases and approaches that of the trialkylamine ([O_2_] ≈ [trialkylamine]), singlet oxygen generation dominates in the case of ^1^DCAS*. This consists with the increase in yields observed at higher back pressures, temperatures and thus higher dissolved [O_2_]. The *k*_q_ for quenching of ^1^DCAS* by O_2_ was slightly higher (4.89 × 10^9^ M^−1^ s^−1^Fig. 7, left) than that reported for ^1^DCA*.^62*a*^ Taken together with the fluorescence quenching rates (*k*_q_) with trialkylamines, this points to a photochemical mechanistic switchover: ^1^DCA* is quenched faster (39×) by 1a than O_2_, while ^1^DCAS* is quenched faster (3×) by O_2_ than 1a. Thus, under aerobic reaction conditions, ^1^DCA* favors an SET mechanism while ^1^DCAS* favors an E_n_T mechanism.

**Fig. 8:**
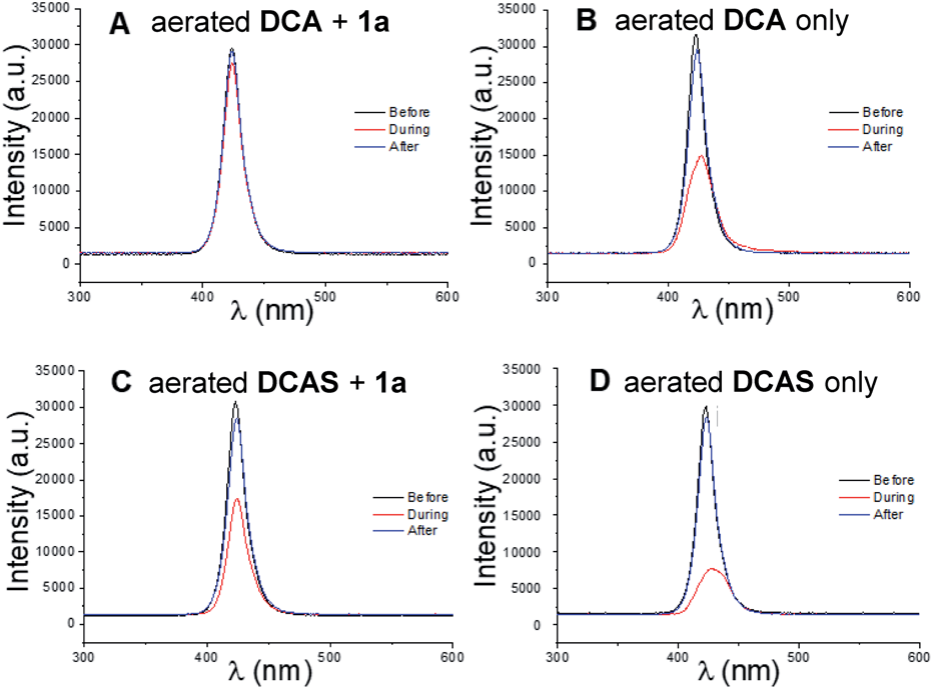
Transmission intensity of light through the tubular reactor. (A) DCA (5 mol%) + 1a (12 mM) in aerated MeCN; (B) DCA only in aerated MeCN; (C) DCAS (5 mol%) + 1a (12 mM) in aerated MeCN; (D) DCAS only in MeCN. Before/After = light transmission before or after the reaction mixture passed through the tubular coil reactor, *i.e.* flowing MeCN only. During = light transmission while the reaction mixture slug is flowing through the coil and upon reaching a steady value.

We then studied the behaviour of the excited cyanoanthracenes under the aerobic reaction conditions (*i.e.*, catalyst, trialkylamine and O_2_ (from air) are all present). This was done by comparing the relative intensity change of light (420 nm) transmitted through the coil of the tubular flow reactor. The principle is as follows: the faster the excited state catalyst can relax to the ground state, the greater the steady-state population of ground state photocatalyst is, leading to more absorption of light (therefore less transmission). The aerated, flowing reaction mixture of DCA (5 mol%) + 1a (12 mM), under the conditions of Table 1, entry 6, gave minimal light absorption (Fig. 8A). As discussed earlier, the reductive quenching of ^1^DCA* by 1a is even faster than quenching by O_2_ and does not directly afford DCA but affords DCA˙^−^ whose absorption^51,53^ is shifted far into the visible green region and thus is not detected by the probe. Regeneration of the ground-state catalyst relies on the oxidation of DCA˙^−^ by O_2_, which is comparatively slow. In the absence of 1a, the aerated solution of DCA (Fig. 8B) gave strong light absorption (decrease of transmitted light intensity to roughly half). O_2_ no longer competes with 1a and is now the exclusive quencher, regenerating and sustaining a large steady-state concentration of absorbing DCA*via* rapid E_n_T quenching of ^1^DCA*. In contrast, the reaction mixture (Table 1, entry 4) of DCAS (5 mol%) in MeCN gave notable light absorption even when 1a (12 mM) was present (Fig. 8C), since quenching of ^1^DCAS* by O_2_ now outcompetes reductive quenching by 1a, ensuring a larger steady-state concentration of absorbing DCAS. This agrees with the aforementioned differences in quenching rate constants. Finally, the light absorption of an aerated solution of DCAS in the absence of 1a (Fig. 8D) was greater in the absence of competing 1a, and was more pronounced than in the case of DCA (Fig. 8B). This reflects the enhanced fluorescence quenching of the former with O_2_ (for light transmission measurements under N_2_ or with 380 nm, see ESI†).

The lifetimes of ^1^DCA* and ^1^DCAS* as measured by Time-correlated Single Photon Counting (TSCPC) in MeCN under Ar were similar, at 14.5 and 13.8 ns, respectively (Table 5).

**Table 5:** TCSPC-determined lifetimes of dicyanoanthracene catalysts under Ar *vs.* under air^a^

Entry	Excited state PC	Sample preparation	*t* (ns)
1	DCA	Ar bubbling, 5 min	14.5 (14.9)^b^
2	DCA	Equilibrated in air	12.7 (12.6)^b^
3	DCAS	Ar bubbling, 5 min	13.8
4	DCAS	Equilibrated in air	9.1

a See ESI for experimental details of TCSPC.

b Literature values.

**Fig. 9:**
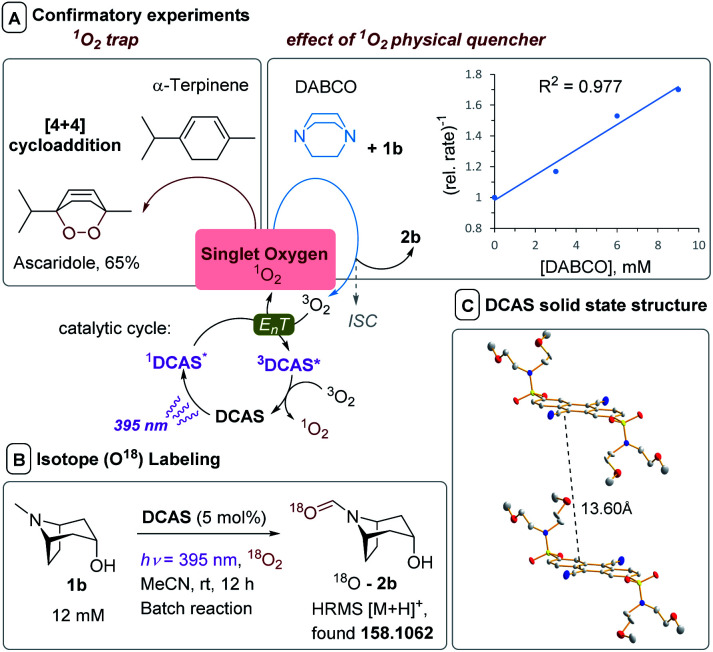
Experiments evidencing (i) an ^1^O_2_ mechanism, (ii) fixation of oxygen atoms from O_2_ and (iii) breakup of π-stacking for DCAS. (A) Left: ^1^O_2_ trapping *via* [4 + 2] cycloaddition. Reaction conditions: α-terpinene (12 mM) DCAS (5 mol%), O_2_ (8 bar), *R*_T_ = 13.5 min, *hν* = 395 nm; right: effect of increasing [DABCO] on the (relative rate)^−1^ of N–CH_3_1b oxidation. Reaction conditions: 1b (12 mM), DABCO (0 to 9 mM), DCAS (5 mol%), O_2_ (8 bar), *R*_T_ = 13.5 min, *hν* = 395 nm. Relative rate = (yield of 2b)/(yield of 2b with DABCO). (B) Isotope labeling batch reaction with ^18^O_2_ gas. (C) XRD crystal structure of 2 molecules of DCAS with the distance between the anthracene cores. Thermal ellipsoids are set at the 50% probability level. H atoms are omitted for clarity, C atoms (grey), N atoms (blue), O atoms (red) S atoms (yellow).

The lifetime of ^1^DCA* was 1.8 ns lower in presence of air, while the lifetime of ^1^DCAS* was 4.7 ns lower, confirming the slight enhancement of quenching by O_2_ (and consistent with the Stern–Volmer *k*_q_s of ^1^DCAS* and ^1^DCA*, *vide supra*). Further experiments supported the photosensitized E_n_T quenching of ^1^DCAS* as the dominant mechanism, rather than photoinduced SET to afford O_2_˙^−^ (Fig. 9A and B). Firstly, when α-terpinene was employed as the substrate, ascaridole was formed in 65% yield as quantified by ^1^H NMR. Endoperoxide formation is a hallmark reporter for ^1^O_2_ through its Diels–Alder [4 + 2]-cycloaddition with dienes (Fig. 9A, left),^63^ thus confirming ^1^DCAS* is capable of ^1^O_2_ generation. Secondly, the presence of DABCO as an additive inhibited conversion in 1b's reaction (Fig. 9B, right). Despite DABCO's low oxidation potential (*E*^p^_ox_ = +0.66 V),^13^ this inhibition was not due to its competitive SET reductive quenching of ^1^DCAS*, since the quenching rate constant (*k*_q_ = 7.28 × 10^8^ M^−1^ s^−1^) confirmed it was even less efficient as a quencher of ^1^DCAS* than O_2_ or 1a/1b (Fig. 7). Rather, DABCO is a well-known physical quencher of ^1^O_2_.^64,65^ This was confirmed by a linear correlation (*R*^2^ = 0.997) between the reciprocal relative rate and [DABCO], an experiment designed by Lapi and co-workers. Finally, as proof of the direct fixation of oxygen atoms from O_2_ gas into trialkylamines, ^18^O-2b was detected by HRMS when isotopically-enriched oxygen (^18^O_2_) gas was employed in the batch reaction of 1b (Fig. 9B).

In summary, increased efficiency of DCAS over DCA in the reaction is not only attributed to the former's enhanced solubility. The sulfonamide substituents at the 2,6-positions of the dicyanoanthracene markedly decrease the reductive quenching of ^1^DCAS* by trialkylamines, compared to that of ^1^DCA*. This observation may be explained by a change in the aggregation state of the organophotocatalyst,^61^ where the ordered π-stacking of DCA aggregates creates a large effective volume for collisions with amines, while DCAS behaves differently. The distance of π-sandwich planes for DCA = 3.37 Å and the usual range for 2 interacting planes is 3.3 to 3.8 Å.^66^ In the X-ray diffraction (XRD) structure of DCAS (Fig. 9C), the distance between π-planes of anthracene = 13.60 Å and considering that 2*r* = distance between molecules, this value agrees with 2× the calculated spherical radii of DCAS in MeCN (Table 4). From this, we tentatively propose that the bulky, freely-rotating sulfonamide substituents sterically inhibit bimolecular (or unimolecular)^59^ quenching events with trialkylamines. The smaller O_2_ molecules outcompete larger trialkylamines to reach the cyanoanthracene core, diverting the mechanism to ^1^O_2_ sensitization. This consists with the need for constrained trialkylamine substrates with protruding N–CH_3_ groups herein, and may rationalize DABCO's inefficiency as a reductive quencher on steric grounds.^13^ A similar “steric-bulk” strategy was recently employed using *tert*-butyl substituents to prevent an unproductive EDA complexation in a catalytic reaction.^67^

**Fig. 10:**
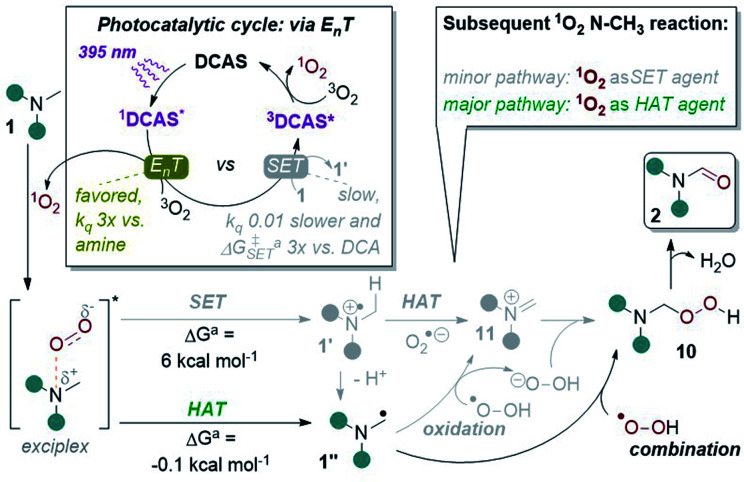
Proposed reaction mechanism with key experimental and computational evidence. ^*a*^DFT calculations at CAM-B3LYP/6-31++g(2d,p), CPCM(acetonitrile) level of theory.

In light of all the above, we propose the following mechanism (Fig. 10). Photoexcitation of DCAS affords ^1^DCAS* which undergoes E_n_T with ^3^O_2_. The generated ^1^O_2_ interacts with the trialkylamine *via* a well-studied exciplex,^62,64,68,69^ which can undergo one of two pathways: SET or HAT. Redox potentials dictate SET between trialkylamines (*E*^p^_ox_ > +0.9 V *vs.* SCE)^13^ and ^1^O_2_ (*E*^p^_red_ > +0.1 V *vs.* SCE)^68^ is endergonic, consistent with our DFT calculations of an endergonic free energy (Δ*G* = 6.0 kcal mol^−1^). Thus, we deemed SET within the exciplex as the minor pathway. Conversely, HAT within the exciplex was slightly exergonic (Δ*G* = −0.1 kcal mol^−1^) suggesting this is the major pathway. Combination of 1′′ with proximally-generated peroxyl radical affords 10 (which could also be accessed by SET oxidation of α-amino radical 1′′ by ^1^O_2_ followed by combination of 11 with O_2_˙^−^ and subsequent protonation is also possible). Finally, liberation of H_2_O from 10 affords 2 and DCAS is regenerated by the reported triplet–triplet annihilation of ^3^DCAS* with a second molecule of ^3^O_2_.^62^

In a recent study by Rovis, Schoenebeck and co-workers on the photocatalytic functionalizations of cyclic trialkylamines,^14*e*^ they proposed that a reversible and fast HAT is responsible for their endocyclic selectivity. Our computational studies point to a rapid, irreversible HAT in the ^1^O_2_-trialkylamine exciplex, thus steric factors must govern the selectivity (*i.e.* at the less sterically demanding N–CH_3_ position). In the case of less-constrained trialkylamines (1u, 1v), the ^1^O_2_-bound exciplex can react promiscuously in HAT with endocyclic/non-N–CH_3_ positions (*e.g.* benzylic groups, free alcohols) leading overall to degradation.

## Conclusions

Herein, we report DCAS as a new organophotocatalyst for late-stage N–CH_3_ to *N*-formyl oxidations of complex trialkylamine-containing natural products and pharmaceuticals, using molecular oxygen and continuous flow. Redox sensitive functionalities were tolerated, allowing the LSF post-modification of alkaloids and macrolide antibiotics to their *N*-formyl derivatives in good yields with excellent chemo- and regioselectivities, all in a continuous manner. The safe handling of O_2_ under increased back pressures and temperatures *via* gas–liquid continuous flow in turn promoted mass transfer of O_2_ to the reaction, increasing yields, shortening reaction (residence) times to several minutes and unleashing synthetically useful productivities (0.65 g per day). Mechanistic insights demonstrate how seemingly minor structural variations in an organophotocatalyst can not only increase solubility, but profoundly divert the excited state mechanism from photoinduced SET to E_n_T, followed by a downstream HAT mechanism. Precious metal photocatalysts of Ru- and Ir-based polypyridyl complexes are well known to participate in both E_n_T and SET, where structural tuning of ligands can affect switching between the divergent pathways. To our knowledge, such a concept has rarely been exploited in organophotocatalysis on the same core, privileged organophotocatalyst structures are typically developed either for SET or E_n_T pathways. Switching the mechanism offers opportunities to control selectivity, as indicated by the tolerance of reductively-labile groups herein. With the generation of ^1^O_2_ revealed, our study showcases one of few successful applications of ^1^O_2_ as a reagent in complex natural product synthesis.^31*c*,70^ Further investigations on the selectivity of ^1^O_2_'s reactions with trialkylamines and the nature of interactions between DCAS, O_2_ and trialkylamine quenchers are ongoing.^71^

## Data availability

Respectfully, all experimental and computational data is adequately available and retreivable from the ESI file.†

## Author contributions

M. J. P. M. contributed the major effort on the optimization of the organophotocatalytic reaction in flow (microfluidic reactor), synthesized substrates, synthesized and purified all products and performed experimental mechanistic studies and computation; J. Ž developed an efficient synthetic route to new catalyst DCAS and synthesized gram quantities for the study; C. I. M. contributed preliminary studies on the optimization of the organophotocatalytic reaction in flow (tubular reactor); L. J. E. supervised and guided J. P. B. and C. I. M. in preliminary studies, measured light source emissions and transmission spectroscopy; A. S. undertook Stern–Volmer quenching studies and measured UV-vis spectroscopy of radical anion catalyst forms; L. d'H. contributed to the synthesis of complex trialkylamine substrates. P. Y. measured catalyst lifetimes and steady state emission spectroscopy. D. J. S. B. supervised and guided P. Y.; T. G. contributed to the separation of highly polar products by preparative HPLC; J. H. supervised and guided T. G.; M. P. J. supervised and guided J. P. B. in preliminary studies; J. P. B. conceptualized the project, conducted first investigations of photocatalytic trialkylamine activations, first synthesized new catalyst DCAS, measured cyclic voltammetry and UV-vis spectroscopy, guided and supervised C. I. M., M. J. P. M., J. Ž, A. S. and L. d'H. in their contributions.

## Conflicts of interest

There are no conflicts to declare.

## Supplementary Material

SC-013-D1SC05840A-s001

SC-013-D1SC05840A-s002
