# Supplementary material for: An organophotocatalytic late-stage N–CH3 oxidation of trialkylamines to N-formamides with O2 in continuous flow
Source: Chem Sci. 2021 Dec 28;13(7):1912–24. doi: 10.1039/d1sc05840a (PMC8849051; doi:10.1039/d1sc05840a)
Supplement: SC-013-D1SC05840A-s001 [file SC-013-D1SC05840A-s001.pdf]

Electronic Supplementary Material (ESI) for Chemical Science.
This journal is © The Royal Society of Chemistry 2021

SUPPLEMENTARY INFORMATION

ORGANOPHOTOCATALYTIC LATE-STAGE *N*-CH_3 OXIDATION OF TRIALKYLAMINES WITH O_2 IN CONTINUOUS FLOW

Mark John P. Mandigma,[a] Jonas Žurauskas,[a] Callum I. MacGregor,[b] Lee J. Edwards,[b] Ahmed Shahin,[a,c] Ludwig d'Heureuse[a], Philip Yip,[c] David J. S. Birch,[c] Thomas Gruber,[a] Jörg Heilmann,[a] Matthew P. John[b] and Joshua P. Barham*,[a]

[a]Fakultät für Chemie und Pharmazie, Universität Regensburg, 93040 Regensburg, Germany.

[b]GlaxoSmithKline Medicines Research Centre, Gunnels Wood Road, Stevenage SG1 2NY, United Kingdom

[c]Chemistry Department, Faculty of Science, Benha University, 13518 Benha, Egypt

[c]Department of Physics, SUPA, University of Strathclyde, 107 Rottenrow East, Glasgow, G4 0NG, United Kingdom

*Corresponding Author's E-mail: Joshua-Philip.Barham@chemie.uni-regensburg.de

TABLE OF CONTENTS

1. General Experimental Information 2
2. Photoreactor Instrumentation Setups 5
3. Comments on Reagent Handling, Process Safety, and Catalyst Cost Comparisons 8
4. Gram Scale Syntheses of Catalysts 9
5. Initial Experiments and LC-MS Profiles 14
6. Complete Optimization Study in a Gas-Liquid Flow Microreactor 16
7. Data for Substrate Synthesis and Substrate Scope 19
8. UV-vis Absorption, Steady-State and Time-Resolved Emission Investigations 39
9. Calculation of Dicyanoanthracene Derivatives' Physical Properties and Other Mechanistic Investigations 48
10. Copies of NMR spectra 53
11. Estimation of *Exo*- : *Endo*- selectivity by ^{1}H NMR 99
12. X-Ray Cystallography 100
13. Density Functional Theory Computational Details and Cartesian Coordinates 105
14. References 115

1. General Experimental Information

Reagents, Solvents and Working Methods

Unless otherwise stated, all reactions were carried out in oven-dried glassware under atmospheric, benchtop conditions. Temperatures of 0 °C were obtained by means of an ice bath or ice/salt bath. 'Room temperature' (rt) indicates temperatures in the range of 20-25 °C. Removal of solvents (*in vacuo*) was achieved using Heidolph rotary evaporators or Vacuubrand high vacuum pumps protected by a liquid N_2 trap. Preparation of experiments that needed inert conditions were carried out using MBRAUN UNIlab glovebox under an N_2 atmosphere.

Chromatography

Thin layer chromatography (TLC), ALUGRAM® Xtra SIL G/UV_{254} silica plates were used, with UV light ($\lambda = 254$ nm), near-UV light ($\lambda = 366$ nm) and potassium permanganate or cerium molybdate (Hessian's) stain used for visualization. Purification was achieved by column chromatography using Macherey-Nagel silica gel 60 (0.063-0.2 mm) or Merck silica gel 60 (0.040-0.063 mm, 230-440 mesh) in glass columns with either G2 or G3 frits. For the purification of highly polar macrocyclic antibiotics, a preparative HPLC equipped with a 1260 Infinity binary pump, a 1260 Infinity manual injector, a 1260 Infinity fraction collector, a 1260 Infinity diode array detector (all Agilent Technologies, Santa Clara, CA, USA) and a Kinetex® column (Biphenyl, 100 Å, 5 _m, 21.2 _ 250 mm, Phenomenex, Aschaffenburg, Germany) was used.

LC-MS analysis was conducted at GlaxoSmithKline using a Waters ZQ 2000 instrument fitted with a Phenomenex Luna® C18 column.

To confirm the identity of compound **3a** (*nor*-dextromethorphan) formed in the reaction (main manuscript, Scheme 2), purification was achieved by preparative LCMS at GlaxoSmithKline using a Waters ZQ micromass ZQ 2000 instrument or, 'Mass Directed Autoprep', fitted with a Waters ZQ 2996 photodiode array detector.

NMR Spectroscopy

All NMR data were collected using a Bruker Avance 400 Ultrashield instrument (400 MHz, 376 MHz, 162 MHz and 101 MHz for ^{1}H, ^{19}F, and ^{13}C NMR), or a Bruker Avance 300 Ultrashield instrument (300 MHz, 282 MHz, 162 MHz and 75 MHz for ^{1}H, ^{19}F, and ^{13}C NMR) was used. ^{13}C NMR was run in ^{1}H decoupled mode. Analyses of spectra were done using either ACD/Spectrus Processor or Mestrenova version 6.0.2-5475 software. Automatic baseline and phase correction were carried out for all spectra. Multiplicities for coupled signals were denoted as: s = singlet, br. s = broad singlet, d = doublet, t = triplet, q = quartet, quint = quintet, sext = sextet, hept = heptet, m = multiplet, apt. = apparent and combinations thereof. Coupling constants (*J*) are given in Hz. Where appropriate, COSY, DEPT, HSQC and HMBC experiments were carried out to aid assignment. Chemical shifts for ^{1}H NMR were reported as δ, parts per million (ppm), relative to the signal of the solvent used such as: $CHCl_3$ at 7.26 ppm, CH_3CN at 2.13

ppm or CH_2Cl_2 at 5.32 ppm. Chemical shifts for ^{13}C NMR were reported as δ, parts per million (ppm), relative to the signal of the solvent used such as: $CHCl_3$ center line signal of the triplet at 77.16 ppm or CH_3CN at 118.26 ppm or CH_2Cl_2 at 54.0 ppm. There were apparent doubling of most signals due to the rotamer nature of amide compounds. The signals of the solvent use are annotated accordingly.

IR Spectroscopy

FTIR spectroscopy was carried out on a Cary 630 FTIR Spectrometer. Solid and liquid compounds were measured neat as a thin film; wave numbers are reported as cm^{-1}. For compounds **8a**, **8c**, **9a** and **9c**, (DBAS and DCAS derivatives) infrared data were collected using a Perkin-Elmer Spectrum 100 FTIR spectrometer as a neat thin film unless otherwise stated.

Mass Spectrometry

Unless otherwise specified, High Resolution Mass spectral analyses were carried out by the Central Analytical Department of the University of Regensburg in EI or ESI mode using a Finnigan MAT 95, Thermo Quest Finnigan TSQ 7000, Finnigan MATSSQ 710 A or an Agilent Q-TOF 6540 UHD instrument, masses observed are accurate to within ±5 ppm. For compounds **8a-c** and **9a-c**, High Resolution Mass Spectral analyses (HR-MS) were carried out by an in-house service at GlaxoSmithKline on either a Thermo Fisher Exactive instrument or a Bruker maXis impact instrument. Instruments used a quadrupole time-of-flight (Q-TOF) technique with Atmospheric Pressure Chemical Ionisation (APCI), Electrospray Ionisation (ESI) or High-Resolution Nano-Electrospray Ionisation (HNESP), and masses observed are accurate to within ±5 ppm.

UV-visible Spectroscopy

For compounds **8a-c** and **9a-c**, UV-vis spectra were collected using a Merck Spectroquant® Pharo 100 at GlaxoSmithKline.

X-ray Crystallography

X-ray crystallographic analysis was performed by the Central Analytic Department of the University of Regensburg using an Agilent Technologies SuperNova, an Agilent Technologies Gemini R Ultra, an Agilent GV 50 or a Rigaku GV 50 diffractometer. Suitable crystals were mounted on a Lindemann tube oil and kept at a steady temperature of T = 293 K during data collection. The structures were solved with the ShelXT (Scheldrick 2015) structure solution program using the Intrinsic Phasing solution method and by using Olex2 as the graphical interface. The model was refined with ShelXL using Least Squares minimization.

Melting Point Measurement

Melting points are uncorrected and were recorded using a Stuart melting point device up to 300 ^{o}C.

Cyclic voltammetry

Cyclic voltammetry was carried out in a glovebox under N_2 using an Autolab® /PGSTAT302N potentiostat. A platinum wire working electrode (d = 0.50 mm) and platinum gauge counter electrode were employed. The saturated calomel electrode (SCE) was used as a reference electrode. Ferrocene was purified by recrystallisation twice from hexane prior to use and measured as an external standard before and after sample measurements ($E_{1/2}$ = +0.40 V *vs* SCE), $^nBu_4N.PF_6$ was used as supplied commercially. Samples of **DCA** and **DCAS** were prepared at 10.0 mM concentration (in 0.1 M $^nBu_4NPF_6$/MeCN as solvent) using anhydrous, degassed MeCN. Samples were stirred between measurements and left to settle for 3 min. A 50 mV s^{-1} scan rate was used and potentials are given *vs* SCE. Poor solubility for both **DCA** and **DCAS** under these conditions precludes comparing the current height with ferrocene as an external standard) for both species. Nonetheless, signals were observed and plotted with normalized current (Figure S0). Reversibility in the CV infers stability of the corresponding radical anions and even dianions; they do not decompose to evolve cyanide anions. The voltammograms show that less negative potentials are required to reduce ground state **DCAS** to its radical anion and dianion than the potentials required to reduce ground state **DCA** to its corresponding radical anion and dianion. Peak potentials were reported as E^p_{red} while half potentials as $E_{1/2}$ which is the average of the peak potentials after a cycle (*e.g.*, for DCA $E_{1/2}$ = [(-0.54 V) + (-0.63 V)] / 2 = −0.59)

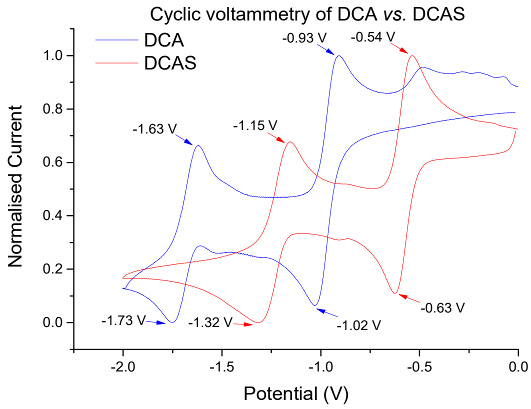

Figure S0. Overlayed cyclic voltammetry of **DCA** and **DCAS**.

2. PHOTOREACTOR INSTRUMENTATION SETUPS

Photoreactor setup in batch

Visible-light photochemical reactions were initially carried out in batch using a Professional LED 30 W floodlight (model no. A65KN from Maplin) positioned 0.10 - 0.15 m from the reaction (Figure 7, main paper). Reactions were performed in Radleys® (RR91080 quick-thread glass reaction tube and RR91088 quick-thread reduced volume) reaction vessels with lids (PTFE quick-thread) automated reaction vessels with lids (PTFE quick-thread) fitted with a gas tap and rubber septum. Degassing was achieved using 3 x freeze/pump/thaw cycles. Reaction vessels were triple evacuated/N_2 filled after sample taking.

Tubular Flow Reactor Setup

Initial studies on recirculated and single-pass photocatalytic flow reactions and mechanistic investigations on light transmission were carried out in Vapourtec UV-150 Photochemical Reactor (R-Series). The reactor was equipped with a fibre-optic probe (BWTEK Inc.) to detect transmission of light through the tubular reactor and a 60 W (input power) 380 nm or 420 nm LED bank. A 10 mL PTFE coil was fitted inside a mirrored cavity and the LED bank inserted into the middle of the coil (Figure S1). The temperature of the reactor was controlled precisely (±1 °C) by a regulated stream of N_2 cooled through a canister filled with dry ice. The reservoir containing the reaction mixture was bubbled with N_2 / dry compressed air / O_2 / a mixture of 1:1 N_2/O_2 for 20 min, before pumping through the flow reactor according to the following typical procedures:

Typical procedure for photochemical oxidation in continuous flow (recirculation approach)

A solution of dextromethorphan **1a** (30 mg, 11 µmol), $LiClO_4$ (11 mg, 11 µmol), DCAS (3.4 mg, 0.6 µmol) in anhydrous MeCN (9 mL) was (after gas bubbling) taken up into a Vapourtec UV-150 (2.0 mL/min, 25 °C). The reaction was further diluted with MeCN (9 mL) and the mixture circulated through the reactor continuously under irradiation from 420 nm LEDs for 3 h. The reactor was then flushed with MeCN (~20 mL) and the solvent removed *in vacuo*. 1,3,5-trimethoxybenzene (18 mg, 11 µmol) was added and the yield of **2a** determined by ^{1}H NMR analysis.

Typical procedure for photochemical oxidation in continuous flow (single pass approach).

A solution of dextromethorphan **1a** (30 mg, 11 µmol), DCAS (3.4 mg, 0.6 µmol) in anhydrous MeCN (9 mL) was (after gas bubbling) taken up into a Vapourtec UV-150 (0.5 mL/min). The reaction vessel was rinsed with MeCN (2 mL) and the reaction mixture irradiated with 420 nm LEDs for a single pass. The reactor was flushed with MeCN (20 mL) and the solvent removed *in vacuo*. 1,3,5-trimethoxybenzene (18 mg, 11 µmol) was added and the yield of **2a** determined by ^{1}H NMR analysis.

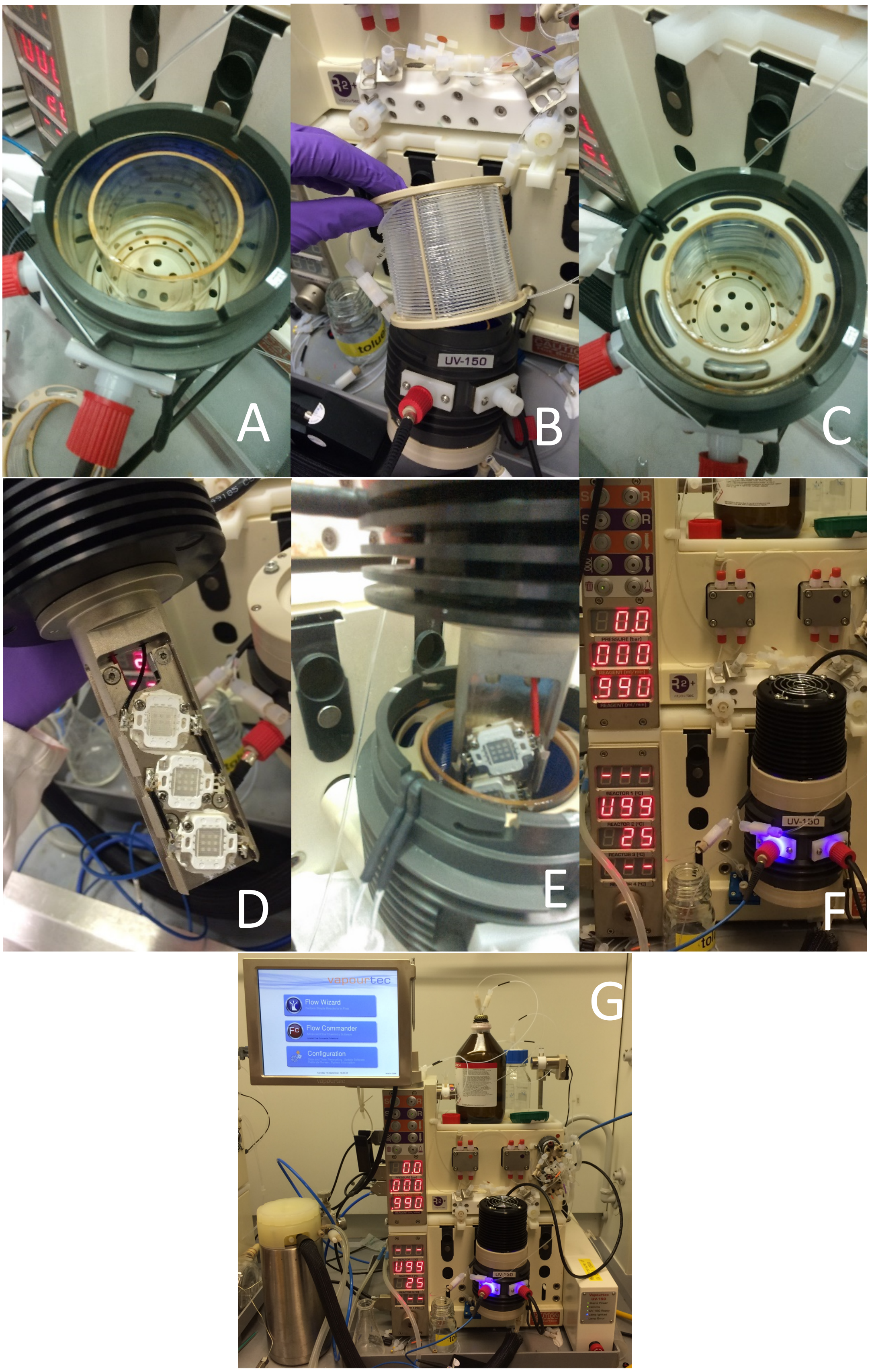

Figure S1. Photos of the tubular photoflow reactor setup. **A**. Mirrored cavity for reactor coil. **B**. 10 mL Fluoropolymer reactor coil. **C**. Assembled coil. **D**. LED bank. **E**. Assembled photoreactor coil. **F**, **G**. Fully assembled photoreactor flow system (Vapourtec R-series with Vapourtec UV-150 reactor).

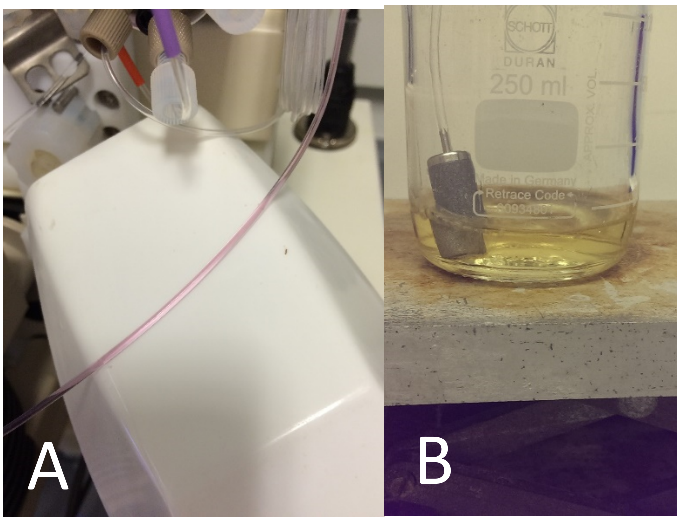

Figure S2. **A**. Photo of post-reactor coil (after irradiation) purple reaction mixture of **1a** + **DCAS** in the absence of O_2 (N_2-purged), see main manuscript, Table 1 entry 3. **B**. Photo of same post-reactor coil reaction mixture (main manuscript, Table 1 entry 3) upon collection / exposure to air.

Microfluidic Photochemical Flow Reactor Setup

Optimization and substrate scope were carried out using the Corning® Advanced-Flow™ Lab Photo Reactor set-up (Figure S3). It is comprised of one fluidic module type G1 LF (glass) for low flow rates, two LED panels composed of 6 x 20 LED each (20 LEDs of each wavelength on each panel, irradiation from both sides, radiant power of each LED at 100%: 1360 mW (700 mA), therefore 27.2 W input power for each panel), a dual piston pump with a metal-free Teflon pump head for the liquid reaction solution, a gas flow meter (Bronkhorst EL-FLOW Select – calibrated for O_2) and controller (Bronkhorst HIGH-TECH, calibrated for O_2), an adjustable back pressure regulator (BPR) and two thermostat units (for controlling temperature of LEDs and for controlling the user-specified temperature of the fluidic module). Settable wavelengths of the LEDs were 340, 375, 395, 420, 450 and 530 nm; with the aid of a smart phone and through WiFi connectivity, appropriate wavelength of light (395 nm or 420 nm) was used in this study as guided by the UV-vis spectra of the catalysts. The fluidic module or reactor chip was designed for the mixture of two flow paths (one for liquid and one for gas; with a total reactor volume of 2.7 mL). Before the liquid solution enters the reaction chip, O_2 gas was introduced to the system. Then, the liquid component was introduced through the inlet tube of the liquid pump set initially at a high flow rate. Whenever appropriate, desired back pressure (up to 8 bars – an operating pressure chosen by us – the reactor system can handle up to 13 bars at 60 ^oC or 120 ^oC at 8 bars) was applied by an integrated back pressure regulator and was monitored by a pressure sensor integrated into the pump module. Then, the flow rate of the liquid was gradually and incrementally brought down to the desired flow rates. For reactions at higher back pressures (8 bars), an S 2100 Quaternary Gradient HPLC pump (Sykam Chromatography) was employed to ensure higher precision of delivery at low liquid flow rates (0.2 mL/min) under higher back pressure.

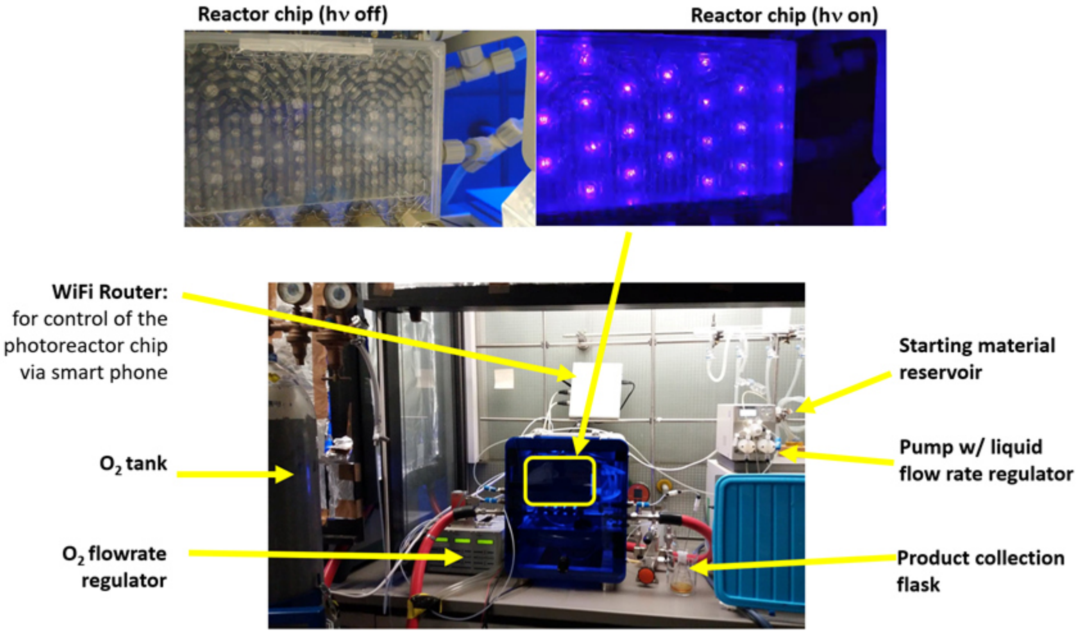

Figure S3. Photos of the microfluidic gas-liquid photo reactor setup.

3. Comments on Reagent Handling, Process Safety, and Catalyst Cost Comparisons

Working with organic solvents

The experimental set-up involves the use of acetonitrile as solvent and conditions were kept below the autoignition temperature of (auto-ignition temperature at 524 °C).[1] For reactions which involves high pressure of oxygen which has a tendency of decreasing the minimum autoignition temperature of the solvent[2] the following precautions / safety features were considered: i) worked under microfluidic setup to minimize the volume of Oxygen enriched Acetonitrile; ii) reactor chip is equipped with 2 layers of acrylic material (first layer for the reaction mixture and the second layer for temperature control system); iii) LED array is equipped with a cooling system and is kept between 20 to 25 °C.

Working with high pressures O_2

Safety considerations on the oxygen enriched organic solvent due to high back pressure (Henry's law) was as discussed above. To ensure no back flow of organic solvent to the oxygen cylinder, the oxygen tank's safety regulator was always strictly set at 1-2 bars higher than the applied back pressure on the flow reactor system. Oxygen gas flow rates are regulated, monitored by a mass flow meter/controller (Bronkhorst).

Working with hi-power light irradiation

The LEDs used for the entire study do not emit ionizing radiation. The flow equipment is geared with UV-light protective casing. Nonetheless, goggles with UV protection were employed during operation when working. Safety considerations for working with singlet oxygen were noted and considered.[3]

Cost of DCAS Precursors

The precursor to DCAS is anthraquinone-2,6-disulfonic acid, readily synthesized *via* classical chemistry procedures from oleum + anthraquinone which costs <0.1 EUR/g (Sigma-Aldrich largest pack size). It is possible to skip this step and buy directly disodium anthraquinone-2,6-disulfonate (**19 EUR/g**, TCI largest pack size). The only other reactant that carried significant expense is bis(2-methoxyethyl)amine, <0.6 EUR/mL (Sigma-Aldrich largest pack size). All other reactants (Zn, ammonium carbonate, bromine, $POCl_3$, CuCN) are found in most laboratories and none of them exceed >0.5 EUR/g. As mentioned in the main text and demonstrated in the SI file, the whole synthesis is end-to-end **chromatography-free**.

As an example comparison, the use of 1-Me-AZADO (manuscript Ref. 28) costs **1,980 EUR/g** (Sigma Aldrich largest pack size). DCAS is employed in 5 mol%, *vs* the employment of 10-20 mol% of 1-Me-AZADO.

4. Gram Scale Syntheses of Catalysts

Anthraquinone-2,6-disulfonic acid (**4**)

The title compound was purchased commercially or synthesized by known procedures.[4]

Anthracene-2,6-disulfonic acid (5)[5]

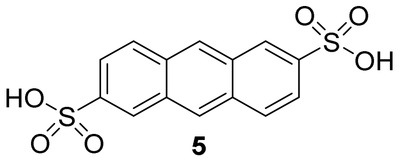

5

Prepared according to a literature procedure.[5] Zinc metal was activated by washing twice with 3% HCl (aq.) and once each with H_2O, EtOH and Et_2O. The Zn was dried *in vacuo* before use. Anthraquinone-2,6-disulfonic acid **4** (24.5 g, 68.1 mmol) was dissolved in 20% $(NH_4)_2CO_3$ (aq.) (138.0 g in 700 mL H_2O). Activated Zn (25.0 g, 382.0 mmol) was added and the reaction heated at 70 °C (liberated gases were passed through a water bubbler). After 3 h, a second portion of activated Zn (25.0 g, 382.0 mmol) was added and the reaction mixture heated at 70 °C for 12 h. The hot reaction mixture was filtered to remove Zn salts. The filtrate was cooled to 0 °C and conc. H_2SO_4 (ca. 80 mL) was added (until pH = 1). The resulting precipitate was collected and washed with acetone. The crude product was re-slurried in H_2O (750.0 mL) and KCl (50.0 g). The slurry was heated to 100 °C and filtered whilst hot. The collected precipitate was washed with acetone and dried in vacuo to yield analytically pure **5** as a sandy yellow powder (14.7 g, 65%). Data are consistent with the literature.[5]

m.p. >400 °C (lit. >400 °C);

^{1}H NMR (400 MHz, DMSO-d_6) δ 8.60 (br. s, 2H), 8.28 (s, 2H), 8.03 (d, J = 9.1 Hz, 2H); 7.70 (dd, J = 8.8, 1.7 Hz, 2H);

^{13}C NMR (101 MHz, $CDCl_3$) δ 145.1, 131.0, 130.6, 127.6, 126.6, 124.0, 123.8.

9,10-dibromoanthracene-2,6-disulfonate dipotassium salt (**6**)[5]

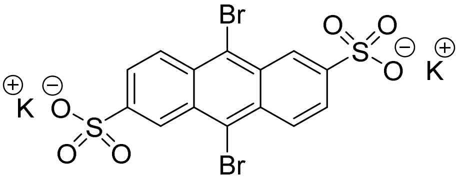

6

Prepared according to a literature procedure.[5] Anthracene-2,6-disulfonic acid **5** (14.7 g, 43.4 mmol) was slurried in a mixture of AcOH (350 mL) and 48% HBr (aq.) (350 mL). The reaction vessel was connected to a bubbler containing sodium hydroxide (5 M) (500 mL) to as a HBr scrubber. Bromine (14.6 mL, 284.0 mmol) was added dropwise before the reaction mixture was heated to 60 °C for 18 h under N_2 and secluding ambient light. After cooling to rt the reaction mixture was concentrated in vacuo to remove most of the solvent (collected liquors containing Br_2 were quenched with aq. $Na_2S_2O_3$ until the brown colour disappeared), before pouring onto ice water (100 mL). Recrystallisation from H_2O (1200 mL), EtOH (600 mL) and KOH (45%, 12.10 g) gave **6** as a fluffy yellow solid (11.27 g, 52%). Data are consistent with the literature.[5]

m.p. >400 °C, (lit. >400 °C);

^{1}H NMR (400 MHz, DMSO-*d*$_6$) δ 8.80 (d, *J* = 1.0 Hz, 2H), 8.52 (d, *J* = 9.1 Hz, 2H), 7.96 (dd, *J* = 9.1, 1.5 Hz, 2H);

^{13}C NMR (101 MHz, DMSO-*d*$_6$) δ 147.5, 130.3, 130.1, 127.9, 126.7, 123.7, 123.3;

9,10-dibromoanthracene-2,6-disulfonyl dichloride (**7**)[5]

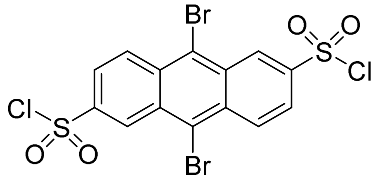

7

To a pre-dried reaction vessel containing anhydrous DMF (7.7 mL, 99.4 mmol) was added $POCl_3$ (8.4 mL, 90.0 mmol) dropwise at 0 °C. The resulting solution was stirred for 10 min before addition of DCM (30 mL) and portionwise addition of 9,10-dibromoanthracene-2,6-disulfonate dipotassium salt* **6** (5.16 g, 9.02 mmol) at 0 °C. The reaction mixture was allowed to reach rt and stirred for 12 h, before it was cooled to 0 °C and filtered into a conical flask containing NaOAc (45.0 g) as a $POCl_3$ quencher. The collected precipitate was washed with ice cold DCM (3 x 10 mL) and acetone (10 mL) and dried *in vacuo* to yield crude **7** as a bright yellow powder (6.32 g, quant.**).

*Alternatively, 9,10-dibromoanthracene-2,6-disulfonic acid could be used and added at -50 °C.

**Assuming quantitative conversion, this was used directly in the next steps without purification.

9,10-dibromo-N$_2$,N$_2$,N$_6$,N$_6$-tetraethylanthracene-2,6-disulfonamide (**DBAS, 8a**)

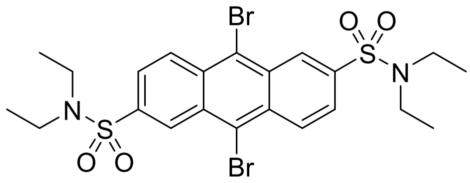

8a

To a suspension of **7** (760.0 mg, 1.43 mmol) in DCM (10.0 mL) was added diethylamine (1.20 mL, 11.6 mmol) at 0 °C. The reaction mixture was allowed to warm to rt and stirred for 6 h, before it was poured into a separatory funnel with DCM:IPA (9:1) (100 mL) and water (100 mL). The layers were separated and the aqueous layer was extracted with further DCM:IPA (9:1) (2 x 100 mL). The combined organic layers were dried ($MgSO_4$), filtered and concentrated in vacuo to yield a yellow solid which was purified by recrystallisation (1:1 DCM:MeOH; dissolving in minimal DCM and slowly adding MeOH (2 mL/h)) to afford analytically pure **8a** as a yellow microcrystalline solid (754.0 mg, 87%);
m.p. 240 - 242 °C;

^{1}H NMR (400 MHz, $CDCl_3$) δ 9.14 (s, 2H), 8.75 (d, J = 9.3 Hz, 2H), 7.96 (dd, J = 9.3, 2.0 Hz, 2H), 3.38 (q, J = 7.2 Hz; 8H), 1.20 (t, J = 7.2 Hz, 12H).
^{13}C NMR (101 MHz, $CDCl_3$) δ 140.7, 132.4, 131.4, 130.8, 128.9, 125.8, 124.6, 42.3, 14.4.
IR ν_{max} (neat) 2973 - 2936, 1618, 1466, 1382, 1338, 1293, 1262, 1202 cm^{-1};
LCMS (Low pH 8 min gradient): t_R = 7.4 min, [M+H$^+$] = 607.0;
HRMS (ESI) *m/z* calcd. for $C_{22}H_{27}Br_2N_2O_4S_2$ [M+H$^+$] 604.9759; found 604.9766.

9,10-dibromo-*N*$_2$,*N*$_2$,*N*$_6$,*N*$_6$-tetrakis(2-methoxyethyl)anthracene-2,6-disulfonamide (**DBAS, 8b**)

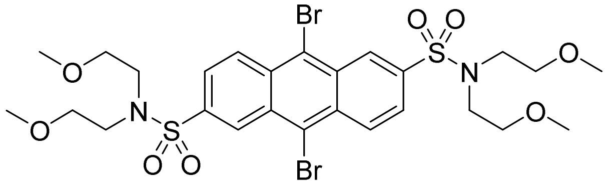

8b

Following procedure for preparation of **8a** using **7** (1.5 g, 2.8 mmol), bis(2-methoxyethyl)amine (2.7 mL, 22.4 mmol) in DCM (25 mL) yielded the analytically pure **8b** as a yellow microcrystalline solid (1.84 g, 90%);
m.p. 228 - 230 °C;
^{1}H NMR (400 MHz, $CDCl_3$) δ 9.16 (s, 2H), 8.74 (d, J = 9.3 Hz, 2H), 8.00 (dd, J = 9.3, 1.7 Hz, 2H), 3.57 (br. s, 16H), 3.27 (s, 12H);
^{13}C NMR (101 MHz, $CDCl_3$) δ 140.5, 132.4, 131.4, 130.6, 129.3, 125.8, 124.8, 71.5, 58.9, 48.6.
IR ν_{max} (neat) 2976 - 2815, 1618, 1486, 1450, 1398, 1369, 1343, 1294, 1262, 1240 cm^{-1};
LCMS (High pH 5 min gradient): t_R = 3.5 min, [M+H$^+$] = 727.0;
HRMS (ESI) *m/z* calcd. for $C_{26}H_{35}Br_2N_2O_8S_2$ [M+H]$^+$ 725.0181; found 725.0173.

9,10-dibromo-N_2,N_2,N_6,N_6-tetrakis(decyl)anthracene-2,6-disulfonamide (**DBAS, 8c**)

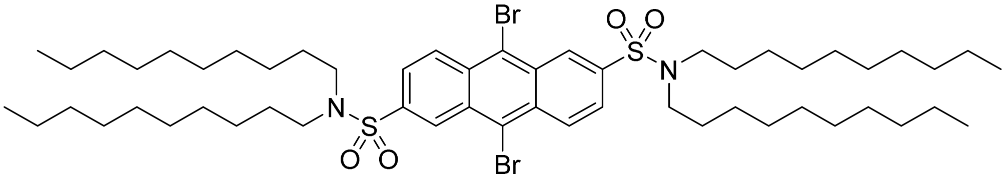

8c

Following procedure for preparation of **8a** using **7** (987.5 mg, 1.85 mmol), didecylamine (2.34 g, 7.90 mmol) in DCM (12.5 mL) yielded the analytically pure **8c** as a yellow microcrystalline solid (1.45 g, 74%);

m.p. 134 - 136 ^{o}C;

^{1}H NMR (400 MHz, $CDCl_3$) δ 9.13 (s, 2H), 8.74 (d, *J* = 9.3 Hz, 2H), 7.95 (dd, *J* = 9.3, 1.7 Hz, 2H), 3.25 (apt. t, *J* = 7.6 Hz, 8H), 1.30 - 1.20 (m, 58H), 0.94 - 0.89 (m, 8H), 0.86 (t, *J* = 7.0 Hz, 12H);

^{13}C NMR (101 MHz, $CDCl_3$) δ 140.6, 132.3, 131.4, 130.7, 129.0, 125.8, 124.8, 48.2, 32.0, 29.6 (2x), 29.4, 29.3, 28.8, 26.9, 22.8, 14.2;

IR ν_{max} (neat) 2955 - 2850 (C-H), 1617 (Ar), 1466, 1398, 1370, 1340, 1293, 1260, 1238, 1210 cm^{-1};

LCMS (High pH 5 min gradient): t_R = 4.1 min, [M+H$^+$] = not identified;

HRMS (ESI) *m/z* calcd. for $C_{54}H_{90}Br_2N_4O_4S_2$ [M+H$^+$] 1054.4709; not detected.*

*Compound was unstable to ESI HRMS.

9,10-dicyano-N_2,N_2,N_6,N_6-tetraethylanthracene-2,6-disulfonamide (**9a**)

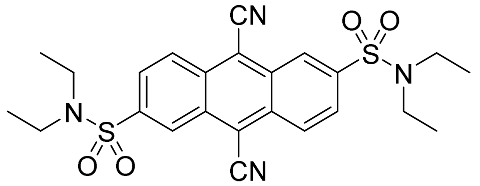

9a

A mixture of dibromide (645.5 mg, 1.06 mmol) and CuCN (380.0 mg, 4.25 mmol) in anhydrous DMF (24 mL) was degassed (3x freeze/pump/thaw) and heated at 210 ^{o}C under microwave irradiation until starting material depleted by LCMS (ca. 15 min). Upon cooling to rt, a fine orange precipitate was observed, which was collected by filtration, washed with DMF (2 x 10 mL) and water (2 x 10 mL) and dried *in vacuo* to afford analytically pure **9a** as a yellow powder (347.6 mg, 66%);

m.p. 313 - 315 ^{o}C;

^{1}H NMR (400 MHz, CD_2Cl_2*) δ 8.96 (s, 2H), 8.70 (d, *J* = 9.1 Hz, 2H), 8.17 (dd, *J* = 9.1, 1.7 Hz, 2H), 3.41 (q, *J* = 7.1 Hz, 8H), 1.18 (t, *J* = 7.1 Hz, 12H);

^{13}C NMR (101 MHz, CD_2Cl_2*) δ 143.6, 133.7, 132.7, 128.9, 127.5, 126.3, 115.3, 114.4, 42.8, 14.6;

IR ν_{max} (neat) 3085 - 2939, 2222, 1676, 1619, 1470, 1405, 1386, 1360, 1335, 1300, 1287, 1260, 1202 cm^{-1};

UV-vis (MeCN): λ_{max} = 393, 420 nm;

LCMS (High pH 5 min gradient): t_R = 3.4 min, [M+H$^+$] = not identified;

HRMS (ESI) *m/z* calcd. for $C_{24}H_{27}N_4O_4S_2$ [M+H$^+$] 499.1474; not detected.**

*Solubility in $CDCl_3$ was too low to obtain ^{13}C NMR. ^1H NMR (400 MHz, $CDCl_3$) δ 9.00 (s, 2H), 8.70 (d, *J* = 9.1 Hz, 2H), 8.20 (dd, *J* = 9.1, 1.5 Hz, 2H), 3.43 (q, *J* = 7.1 Hz, 8H), 1.22 (t, *J* = 7.1 Hz, 12H).
**Compound unstable to ESI HRMS.

9,10-dicyano-*N*₂,*N*₂,*N*₆,*N*₆-tetrakis(2-methoxyethyl)anthracene-2,6-disulfonamide (**9b**)

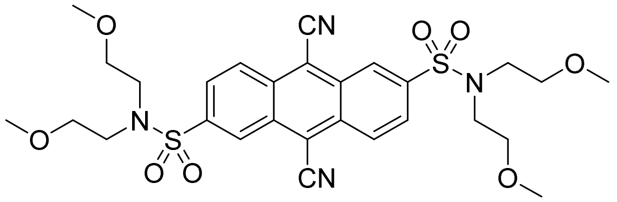

9b

Following procedure for preparation of **9a** using dibromide **8b** (802.0 mg, 1.10 mmol) and CuCN (400.0 mg, 4.47 mmol) afforded analytically pure **9b** as a yellow powder (605.0 mg, 89%);*

m.p. 207 - 209 °C;

^{1}H NMR (400 MHz, $CDCl_3$) δ 9.03 (d, *J* = 1.5 Hz, 2H), 8.64 (d, *J* = 9.3 Hz, 2H), 8.24 (dd, *J* = 9.1, 1.7 Hz, 2H), 3.66 - 3.58 (m, 16H), 3.25 (s, 12H); ^{13}C NMR (101 MHz, $CDCl_3$) δ 143.2, 133.3, 132.1, 127.8 (2x), 126.2, 114.8, 113.8, 71.2, 58.9, 48.2;

IR ν_{max} (neat) 3081 - 2813, 2221, 1617, 1619, 1444, 1404, 1386, 1375, 1358, 1339, 1297, 1258, 1201 cm^{-1};

UV-vis (MeCN) λ_{max} = 393, 422 nm;

LCMS (High pH 5 min gradient): t_R = 3.1 min, [M+H$^+$] = 619.0;

HRMS (ESI) *m/z* calcd. for $C_{28}H_{35}N_4O_8S_2$ $[M+H]^+$ 619.1896; found 619.1878.

Suitable crystals for X-ray analysis were obtained by crystallization using vapor diffusion method with DCM and *n*-hexane as solvents. A single clear light yellow needle shaped crystal with dimensions: 0.10 × 0.05 × 0.01 mm^3 was selected. **Crystal Data** $C_{28}H_{34}N_4O_8S_2$, M_r = 618.71, monoclinic, *C*2/*c* (No. 15), a = 26.7306(7) Å, b = 5.04920(10) Å, c = 24.8887(5) Å, β = 117.317(3)°, $\alpha = \gamma$ = 90°, *V* = 2984.57(14) Å^3, *T* = 123.01(10) K, *Z* = 4, *Z'* = 0.5, μ(Cu K_α) = 2.091, 25114 reflections measured, 2930 unique (R_{int} = 0.0354) which were used in all calculations. The final wR_2 was 0.1216 (all data) and R_1 was 0.0448 (I≥2 σ(I)).

*as an alternative to microwave heating, **8b** (1.40 g, 1.93 mmol) and CuCN (1.0 g, 11.16 mmol) could be heated at 160 °C (oil bath) under Nitrogen atmosphere in a sealed Schlenk tube for 24 h. This afforded **9b** in identical yields (1.06 g, 89%)

9,10-dicyano-*N*₂,*N*₂,*N*₆,*N*₆-tetrakis(decyl)anthracene-2,6-disulfonamide (**9c**)

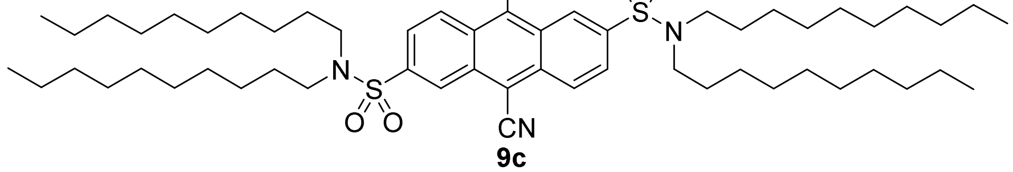

9c

Following procedure for preparation of **9a** using dibromide **8c** (580.7 mg, 0.55 mmol) and CuCN (200.0 mg, 4.40 mmol) afforded a light orange powder (677.2 mg, 65%) which was recrystallized to yield analytically pure **9c** as a yellow powder (275.7 mg, 26%).

m.p. 133 - 135 °C;

^{1}H NMR (400 MHz, $CDCl_3$) δ 8.99 (br. s, 2H), 8.68 (d, *J* = 9.1 Hz, 2H), 8.18 (dd, *J* = 9.1, 1.5 Hz, 2H), 3.28 (apt. t, *J* = 7.6 Hz, 8H), 1.61 - 1.52 (m, 8H), 1.32 - 1.19 (m, 58H), 0.87 (t, *J* = 6.8 Hz, 12H);

^{13}C NMR (101 MHz, $CDCl_3$) δ 143.3, 133.2, 132.2, 128.2, 127.4, 125.8, 114.7, 113.9, 48.3, 32.0, 29.6 (2x), 29.4 (2x), 28.4, 26.8, 22.8, 14.2;

IR ν_{max} (neat) 2956 - 2851, 2226, 1465, 1346, 1260 cm^{-1};

UV-vis (MeCN) λ_{max} = 397, 422, 456 nm;

LCMS (High pH 5 min gradient): t_R = not identified, [$M+H^+$] = not identified;

HRMS (ESI) *m/z* calcd. for $C_{56}H_{91}N_4O_4S_2$ $[M+H]^+$ 946.6403; not detected.*

*Compound unstable to ESI HRMS.

5. Initial Experiments and LC-MS Profiles

Attempts to apply our previously reported trialkylamine photocatalytic oxidation conditions (developed for *N*-alkyl tetrahydroisoquinoline functionalizations) to dextromethorphan **1a** (Figure S4) were caried out. Despite the many reports proposing reductive quenching of $[Ru(bpy)_3]^{2+}$ ($E_{1/2}$ [$^*Ru^{II}/Ru^{I}$] = +0.77 V *vs* SCE)[14] with trialkylamines like Et_3N (E^{p}_{ox} = +1.10 V *vs* SCE), no reaction of **1a** (E^{p}_{ox} = +0.89 V *vs* SCE) was observed with $[Ru(bpy)_3]^{2+}$ photocatalysis, either under anaerobic conditions with haloarene oxidants ($BrCCl_3$, $BrCH_2CN$ or $CH_3(Cl)CH_2CN$) or under air as the terminal oxidant. The more oxidizing excited state derived from $[Ru(bpz)_3]^{2+}$ ($E_{1/2}$ [$^*Ru^{II}/Ru^{I}$] = +1.40 V *vs* SCE) was found to drastically enhance reductive quenching by **1a**, and was attempted in the synthetic reaction. Surprisingly, only trace conversion was observed. When air was used as the terminal oxidant, greater conversion was observed, but over time led to complex reaction mixtures. The same electronic factors imparting a high oxidation potential to $^*Ru(bpz)_3^{2+}$ might have inhibited its reoxidation by the terminal oxidant, meaning that back electron transfer predominates.

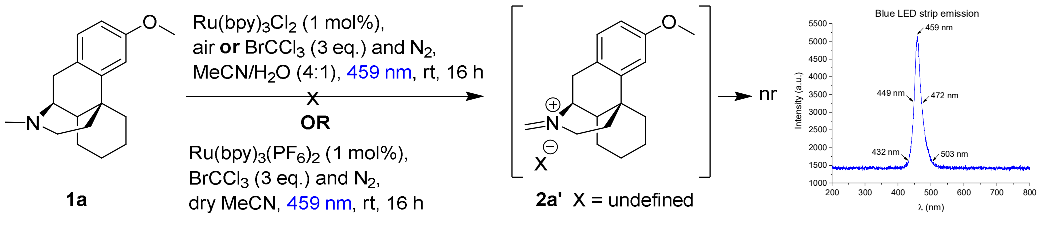

Figure S4. Failed reactions using Ru catalysts. Blue LED emission spectrum (right)

Attempts to repeat the reaction reported by Santamaria and coworkers (Figure S5)[6,7] in batch with visible light sources (30 W white LED floodlamp A65KN from Maplin; emission spectrum in Figure S6) led only to complex mixtures of products, in which main product was **2a** (Figure S7).

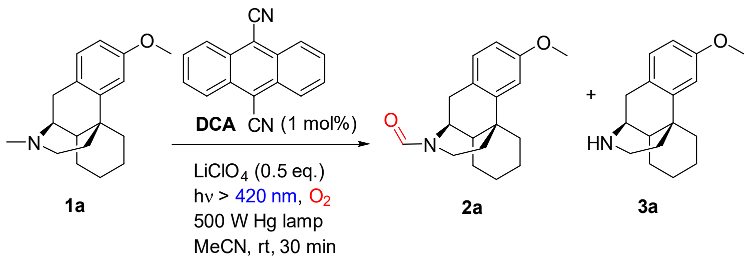

Figure S5. Conditions and results of Santamaria et al. with 500 W Hg lamp and O_2 atmosphere.[6,7]

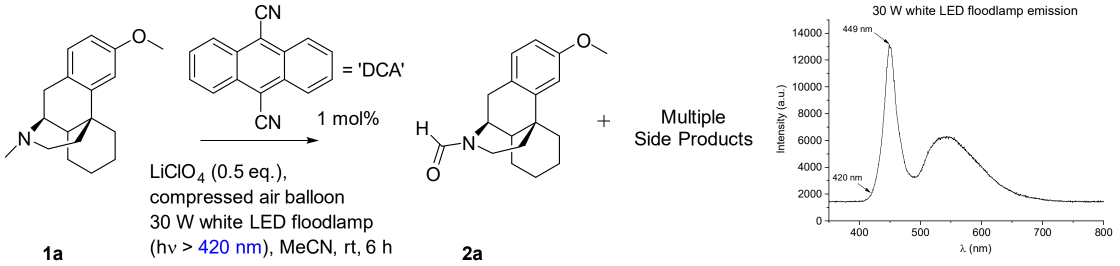

Figure S6. Attempt to use modified conditions of the reaction of Santamaria et al. with 30 W visible LEDs and compressed air bubbling in batch. 30 W visible LEDs flood lamp emission spectrum (right)

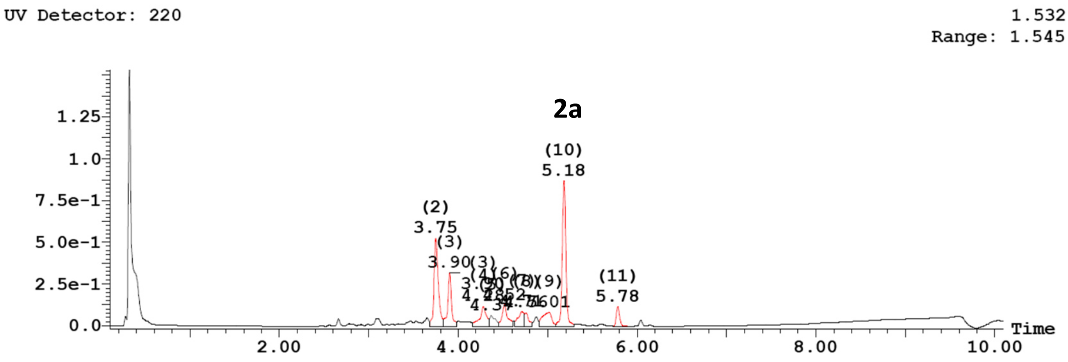

Figure S7. LC-MS chromatogram of reaction in Figure S6 (low pH, 8 min method). R_t = 5.18 min (**2a**).

Initial experiments in continuous flow were done using either **DCAS** or **DCA** (Figure S8). As can be seen in the comparison of LC-MS chromatograms, the reaction profile is cleaner in continuous flow with a 420 nm LED and open to air (Figure S9) *vs* the initial batch conditions (Figure S7). Moreover, **DCAS** affords superior conversion and a cleaner reaction profile than **DCA** under recycling flow conditions.

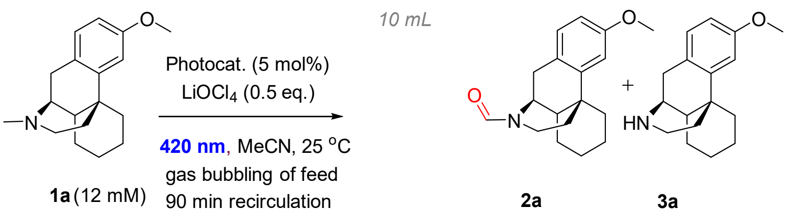

Figure S8. Continuous flow recirculating organophotocatalytic *N*-CH_3 oxidation.

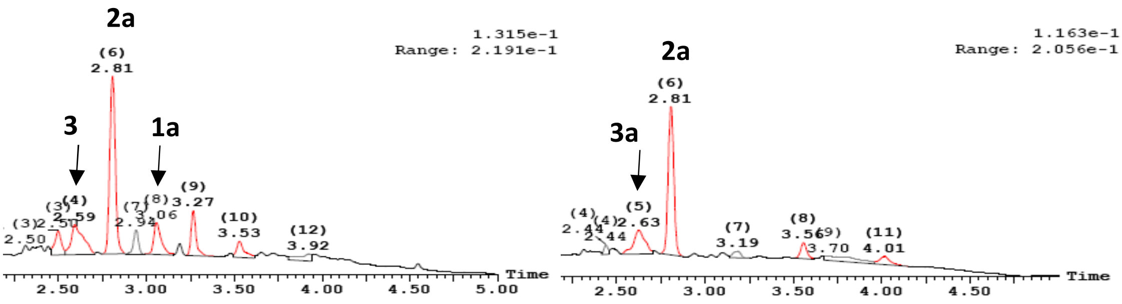

Figure S9. LC-MS chromatogram of the reactions carried out in flow. Left: **DCA** catalyst. Right: **DCAS** catalyst (high pH, 5 min method). R_t = 3.03-3.05 min (**1a**) 2.81 min (**2a**), 2.50-2.60 min broad (**3a**).

As can be seen in Figure S10, presence of water in the solvent system diverts the mechanism to *N*-CH_3 demethylation instead of *N*-CH_3 oxidation.

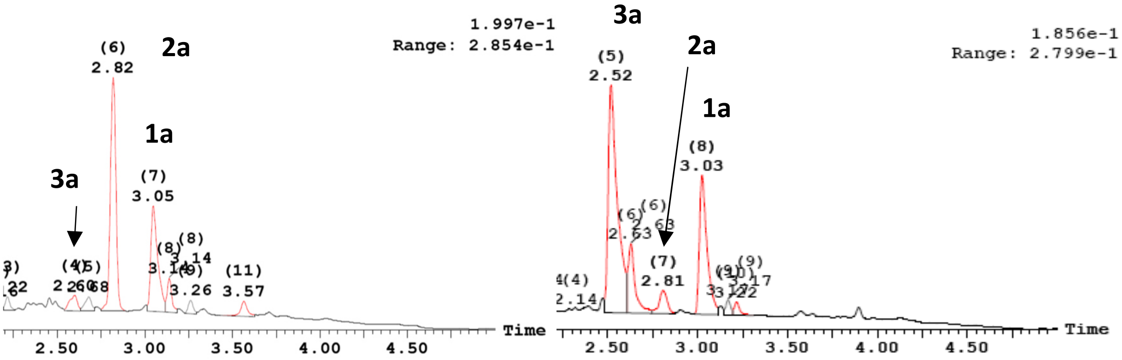

Figure S10. Left: **DCAS** catalyst, dry MeCN, after 3 h. Right: **DCAS** catalyst, MeCN:H_2O (4:1), after 1.5 h. R_t = 3.06 min (**1a**) 2.81 min (**2a**), 2.60 min (**3a**).

6. Complete Optimization Data in a Gas-Liquid Flow Microreactor

Table S1. Optimizations with Dextromethorphan (**1a**)

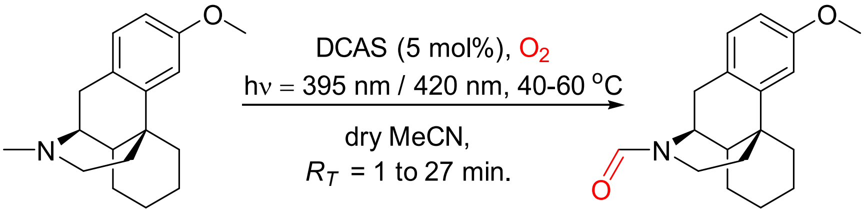

1a (12 - 24 mM) **2a**

Entry	Conditions (Substrate conc, R_T, hn)	% ^{1}H NMR yield
1	12 mM, 13.5 min, 420 nm	22
2	12 mM, 13.5 min, 395 nm	40
3	12 mM, 5.4 min, 420 nm	6

4	12 mM, 5.4 min, 395 nm	12
5	12 mM, ~ 1 min, 420 nm	Trace (2)
6	12 mM, ~ 1 min, 395 nm	Trace (2)
7	12 mM, ~ 1 min, **no light**	N.R. (0)
8	12 mM, 27.0 min, 420 nm	38
9	12 mM, 27.0 min, 395 nm	40
10	24 mM, 13.5 min, 420 nm	20
11	24 mM, 13.5 min, 395 nm	40
12	24 mM, 27.0 min, 420 nm	44
13	24 mM, 13.5 min, 395 nm	44
14	48 mM, 13.5 min, 395 nm	24
**15**	**24 mM, 13.5 min, 395 nm at 60 °C**	**46**

Table S2. Optimizations with Tropine (**1b**)

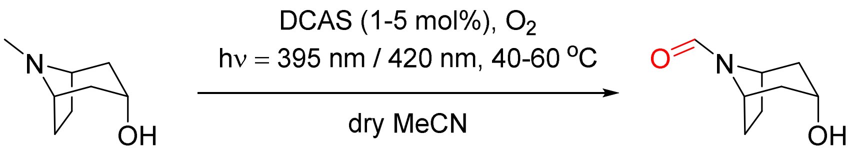

1b (12 - 24 mM) **2b**

Entry	Conditions (Substrate conc, R_T, hν, cat. mol%, temperature)	% yield
16	12 mM, 13.5 min, 420 nm, 5 mol%, 40 °C	10
17	12 mM, 27.0 min, 395 nm, 5 mol%, 40 °C	61
18	12 mM, 27.0 min, 395 nm, 5 mol%, 25 °C	43
19	12 mM, 27.0 min, 395 nm, 5 mol%, 60 °C	50
20	24 mM, 27.0 min, 395 nm, 5 mol%, 25 °C	53
21	24 mM, 27.0 min, 395 nm, 5 mol%, 40 °C	60
22	24 mM, 27.0 min, 395 nm, 3 mol%, 40 °C	53
23	24 mM, 27.0 min, 395 nm, 1 mol%, 40 °C	42
24	24 mM, 27.0 min, 395 nm, 3 mol%, 60 °C	54
**25**	**24 mM, 27.0 min, 395 nm, 5 mol%, 60 °C**	**61**

Table S3. Effect of Oxygen back pressure on the reaction of Tropine (**1b**)

Entry	Conditions (Substrate conc, R_T, hν, cat. mol%, temp., back pressure)	% yield	Productivity (g / day)
25	24 mM, 27.0 min, 395 nm, 5 mol%, 60 °C, none	61	0.33
**26**	**24 mM, 13.5 min, 395 nm, 5 mol%, 60 °C, 7 bars**	**61**	**0.65**
27	24 mM, 6.8 min, 395 nm, 5 mol%, 60 °C, 8 bars	31	0.66
28	48 mM, 27.0 min, 395 nm, 5 mol%, 60 °C, 8 bars	48	0.51
29	24 mM, 13.5 min, 395 nm, 5 mol%, 60 °C, none	43	0.46

Table S4 Sample Data for ^{1}H NMR % Yield determination.

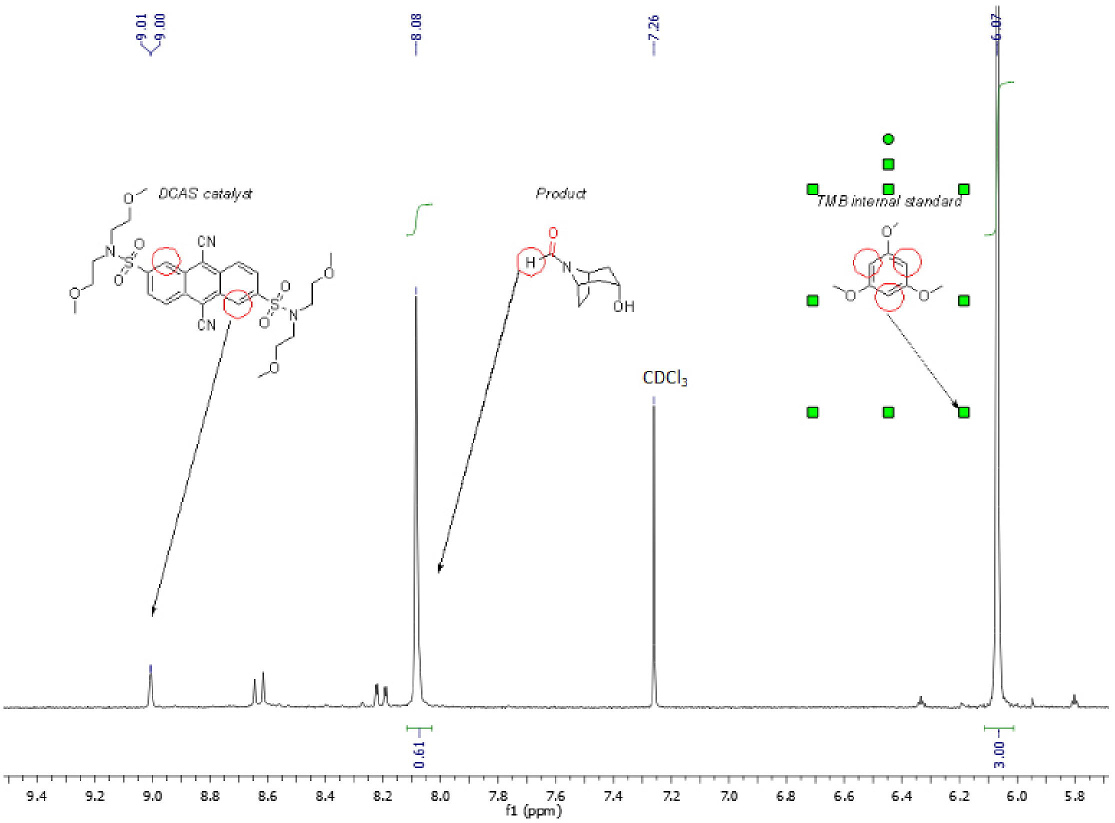

Component	Shift (ppm)	Integration	Number of Protons	Yield[b](%)
**TMB**	6.06	3.00[a]	3	-
**Prod**	8.08	0.61	1	61[b]

[a]1 equivalent of TMB as ^{1}H NMR standard was used, Integration at 6.06 ppm was normalized to 3; [b]% yield = Integration × 100.

7. DATA FOR SUBSTRATE SYNTHESIS AND SUBSTRATE SCOPE

Synthesis of tertiary amine substrates:

(4bS,9S)-3-methoxy-11-methyl-6,7,8,8a,9,10-hexahydro-5H-9,4b-(epiminoethano)phenanthrene (Dextromethorphan free base, 1a)

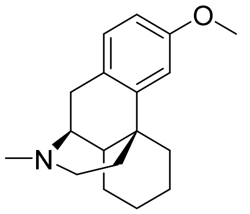

1a

Prepared according to a literature procedure.[8] Dextromethorphan hydrobromide (**1a**·HBr) (2.50 g, 7.10 mmol) was dissolved in H_2O (200 mL). NaOH (1 M) was added to basify the solution until pH 10, as indicated by pH paper, then TBME (30 mL) was added. The layers were separated, and the aqueous layer was extracted with TBME (2 x 30 mL). The combined organic layers were dried with $MgSO_4$, filtered, and concentrated *in vacuo* to yield **1a** as a white solid (1.90 g, 100%). Data are consistent with the literature.[8]

m.p. 109 - 111 °C (lit. 109 - 111 °C)[8]

^{1}H NMR (400 MHz, $CDCl_3$) δ 7.01 (d, *J* = 8.4 Hz, 1H), 6.79 (d, *J* = 2.5 Hz, 1H), 6.68 (dd, *J* = 8.4, 2.6 Hz, 1H), 3.77 (s, 3H), 2.97 (d, *J* = 18.1 Hz, 1H), 2.78 (dd, *J* = 5.2, 3.3 Hz, 1H), 2.56 (dd, *J* = 18.1, 5.7 Hz, 1H), 2.43 – 2.32 (m, 5H), 2.06 (td, *J* = 12.3, 3.3 Hz, 1H), 1.80 (dt, *J* = 13.0, 2.8 Hz, 1H), 1.72 (td, *J* = 12.6, 4.8 Hz, 1H), 1.64 – 1.61 (m, 1H), 1.53 – 1.47 (m, 1H), 1.44 – 1.24 (m, 5H), 1.13 (tt, *J* = 12.4, 6.2 Hz, 1H).

^{13}C NMR (101 MHz, $CDCl_3$) δ 158.2, 141.9, 130.0, 128.6, 111.2, 110.7, 58.1, 55.2, 47.4, 45.6, 43.0, 42.3, 37.4, 36.8, 26.9, 26.7, 23.4, 22.4.

IR ν_{max} (neat): 2922 – 2799, 1502, 1431, 1379, 1326, 1233, 1118 cm^{-1}

HRMS (ESI) *m/z* calcd. for $C_{18}H_{26}NO$ $[M+H]^+$ 272.2014, found 272.2013.

(1R,3r,5S)-8-methyl-8-azabicyclo[3.2.1]octan-3-yl (S)-3-hydroxy-2-phenylpropanoate (Atropine free base, 1d)

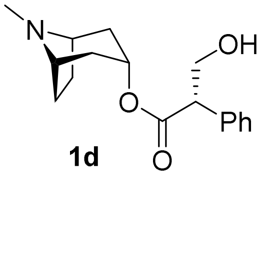

1d

Following procedure for preparation of **1a** using Atropine sulfate monohydrate ((**1d**)$_2$·H_2SO_4·H_2O) (1.00 g, 1.44 mmol) yielded **1d** as white oily solid (807.9 mg, 97%). Data are consistent with the literature.[9,10]

^{1}H NMR (400 MHz, $CDCl_3$) δ 7.44 – 7.20 (m, 5H), 5.03 (t, *J* = 5.3 Hz, 1H), 4.18 (dd, *J* = 13.8, 6.8 Hz, 1H), 3.88 – 3.74 (m, 2H), 3.13 – 2.96 (m, 1H), 2.95 – 2.85 (m, 1H), 2.43 (br. s, 1H), 2.20 (s, 3H), 2.14 – 1.97 (m, 2H), 1.92 – 1.78 (m, 1H), 1.76 – 1.61 (m, 3H), 1.47 (d, *J* = 15.1 Hz, 1H).

^{13}C NMR (101 MHz, $CDCl_3$) δ 172.5, 135.8, 129.1, 128.3, 127.9, 68.4, 64.4, 59.8, 59.7, 54.5, 40.5, 36.7, 36.4, 25.6, 25.1.

IR ν_{max} (neat): 3082, 2945 – 2833, 1718, 1450, 1360, 1274, 1257 cm^{-1}

HRMS (ESI): *m/z* calcd. for $C_{17}H_{24}NO_3$ [M+H]$^+$ 290.1756, found 290.1752.

(1R,2R,4S,5S,7s)-9-methyl-3-oxa-9-azatricyclo[3.3.1.02,4]nonan-7-yl (S)-3-hydroxy-2-phenylpropanoate (Scopolamine free base, 1e)

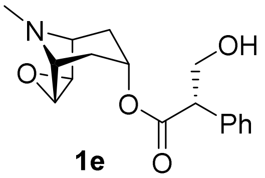

1e

Following procedure for preparation of **1a** using Scopolamine hydrobromide (**1e**·HBr) (0.50 g, 1.30 mmol) yielded **1d** as clear yellowish viscous oil (367.1 mg, 93%). Data are consistent with the literature.[10]

^{1}H NMR (400 MHz, $CDCl_3$): δ 7.38 – 7.27 (m, 3H), 7.22 (dd, *J* = 7.9, 1.3 Hz, 2H), 5.03 (t, *J* = 5.4 Hz, 1H), 4.24 – 4.02 (m, 1H), 3.85 – 3.62 (m, 2H), 3.37 (d, *J* = 2.9 Hz, 1H), 3.11 (d, *J* = 1.8 Hz, 1H), 2.97 (d, *J* = 1.9 Hz, 1H), 2.66 (d, *J* = 2.9 Hz, 1H), 2.46 (s, 3H), 2.25 – 1.92 (m, 2H), 1.58 (d, *J* = 15.2 Hz, 1H), 1.34 (d, *J* = 15.3 Hz, 1H).

^{13}C NMR (101 MHz, $CDCl_3$) δ 172.0, 135.8, 129.2, 128.2, 128.1, 67.0, 64.2, 58.1, 58.0, 56.5, 56.0, 54.4, 42.4, 31.1, 30.9.

IR ν_{max} (neat) 3347, 3030-2937, 1725, 1494, 1453, 1376, 1271, 1233, 1200 cm^{-1}

HRMS (ESI) *m/z* calcd. for $C_{17}H_{22}NO_4$ [M+H]$^+$ 304.1549, found 304.1543.

(1R,3r,5S)-8-methyl-3-((methyldiphenylsilyl)oxy)-8-azabicyclo[3.2.1]octane (1f)

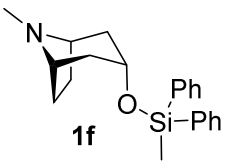

1f

Prepared similarly to a literature procedure.[8] Tropine **1b** (0.73 g, 5.16 mmol) and imidazole (0.61 g, 9.02 mmol) was dissolved in DCM (20 mL). Chloro(methyl)diphenylsilane (1.00 g, 4.3 mmol) in DCM (15 mL) was added dropwise to the solution and was allowed to stir for 12 h overnight. The reaction was quenched with sat. aq. $NaHCO_3$ (15 mL), and the organic layer was collected. The aqueous portion was extracted with DCM (3 x 15 mL). The combined organic layers were dried ($MgSO_4$), filtered and concentrated *in vacuo.* Purification by silica-gel column chromatography (9:1 EtOAc / pentane) gave **1f** (1.16 g, 80%) as clear viscous oil.

^{1}H NMR (300 MHz, $CDCl_3$) δ 7.74 – 7.46 (m, 4H), 7.46 – 7.31 (m, 6H), 4.08 (t, *J* = 4.8 Hz, 1H), 3.16 – 3.01 (m, 2H), 2.34 – 2.28 (m, 2H), 2.28 (s, 3H), 2.11 – 1.90 (m, 4H), 1.72 (d, *J* = 13.2 Hz, 2H), 0.63 (s, 3H).

^{13}C NMR (75 MHz, $CDCl_3$): δ 136.8, 134.3, 129.8, 127.9, 65.6, 60.5, 40.5, 39.6, 25.8, -2.6.

IR ν_{max} (neat): 2926 – 2833, 1468, 1427, 1379, 1342, 1252, 1114, 1047 cm^{-1}

HRMS (ESI) *m/z* calcd. for $C_{21}H_{28}NOSi$ $[M+H]^+$ 338.1940, found 338.1938.

(1R,3r,5S)-8-methyl-8-azabicyclo[3.2.1]octan-3-yl benzoate (1g)

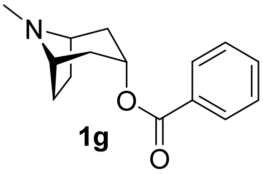

1g

Prepared according to a literature procedure.[11] Benzoyl chloride (0.45 mL, 3.89 mmol) was added dropwise to a solution of tropine **1b** (0.50 g, 3.54 mmol) and Et_3N (0.54 mL, 3.89 mmol) in toluene (20 mL). The mixture was heated at reflux temperature for 12 h. The mixture was washed with with sat. aq. $NaHCO_3$ solution (3 x 25 mL), brine (75 mL) and dried ($MgSO_4$) and concentrated *in vacuo*. The The residue was extensively washed with DCM. Purification by silica-gel column chromatography (9:1 EtOAc / pentane) gave **1g** (0.61 g, 70%) as white amorphous powder. Data are consistent with the literature.[12]

^{1}H NMR (400 MHz, $CDCl_3$) δ 8.01 (dd, *J* = 5.2, 3.3 Hz, 2H), 7.62 – 7.51 (m, 1H), 7.44 (dd, *J* = 10.5, 4.7 Hz, 2H), 5.25 (t, *J* = 5.3 Hz, 1H), 3.16 (br. s, 2H), 2.31 (s, 3H), 2.27 – 2.19 (m, 2H), 2.14 – 2.04 (m, 4H), 1.83 (d, *J* = 14.8 Hz, 2H).

^{13}C NMR (101 MHz, $CDCl_3$) δ 165.9, 132.9, 130.9, 129.5, 128.5, 68.1, 60.0, 40.6, 36.8, 25.9.

IR ν_{max} (neat) 2933 – 2851, 1714, 1449, 1274, 1114, 1036 cm^{-1}

HRMS (ESI) *m/z* calcd. for $C_{15}H_{20}NO_2$ $[M+H]^+$ 246.1494, found 246.1490.

(1R,3r,5S)-8-methyl-8-azabicyclo[3.2.1]octan-3-yl 4-(trifluoromethyl)benzoate (1h)

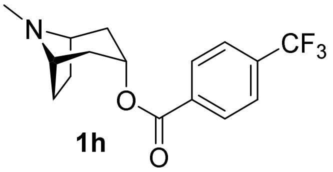

1h

Prepared according to a literature procedure.[12] Dicyclohexylmethanediimine (DCC) (1.49 g, 7.20 mmol) was dissolved in dry DCM (15 mL). To a stirred solution of tropine **1b** (0.85 g, 6.0 mmol), 4-(trifluoromethyl) benzoic acid (1.37 g, 7.20 mmol) and DMAP (0.15 g, 1.20 mmol) in dry DCM, the DCC solution was added dropwise at 0 °C. The resulting reaction mixture was stirred further at r.t. for 24 h, after which the solution was filtered and washed with DCM (2 x 30 mL) and concentrated *in vacuo*. The crude was then dissolved in $CHCl_3$ (30 mL) then washed with sat. aq. NaCl solution (10 mL) and neutralized with a solution of 5% NaOH (10 mL). The product was then extracted with $CHCl_3$ (3 x 30 mL) dried ($MgSO_4$) and concentrated *in vacuo*. Purification by silica-gel column chromatography (a gradient of 100% DCM to 95:5 DCM/MeOH) gave **1h** (1.41 g, 75%) as pale yellow-ish amorphous powder.

^{1}H NMR (300 MHz, $CDCl_3$) δ 8.14 (dd, *J* = 8.8, 0.7 Hz, 2H), 7.78 – 7.67 (m, 2H), 5.29 (t, *J* = 5.3 Hz, 1H), 3.20 (s, 2H), 2.33 (s, 3H), 2.33 – 2.23 (m, 2H), 2.15 – 1.97 (m, 4H), 1.86 (d, *J* = 14.7 Hz, 2H).

^{13}C NMR (101 MHz, $CDCl_3$) δ 164.8, 134.6 (q, *J* = 32.5 Hz), 134.2, 130.0, 125.7 (q, *J* = 3.8 Hz), 123.8 (*observed as* d, *J* = 272.5 Hz), 69.1, 60.0, 40.6, 36.9, 26.0.

^{19}F NMR (376 MHz, $CDCl_3$) δ -63.6.

IR ν_{max} (neat) 2937-2893, 1718, 1599, 1472, 1408, 1375, 1323, 1274, 1252, 1166, 1099 cm^{-1}

HRMS (ESI) *m/z* calcd. for $C_{16}H_{19}F_3NO_2$ $[M+H]^+$ 314.1368, found 314.1365.

(1R,3r,5S)-8-methyl-8-azabicyclo[3.2.1]octan-3-yl 3,4-dimethoxybenzoate (Convolvamine, 1i)

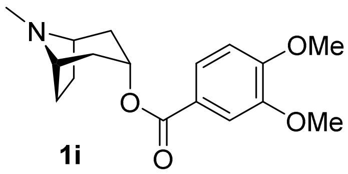

1i

Following procedure for preparation of **1h**[12] using DCC (1.49 g, 7.20 mmol), **1b** (0.85 g, 6.0 mmol), 3,4-dimethoxybenzoic acid (1.31 g, 7.20 mmol) and DMAP (0.15 g, 1.20 mmol) yielded **1i** as white amorphous powder (733.0 mg, 40%). Data are consistent with the literature. [13]

^{1}H NMR (400 MHz, $CDCl_3$) δ 7.66 (dd, *J* = 8.4, 2.0 Hz, 1H), 7.56 (d, *J* = 2.0 Hz, 1H), 6.91 (d, *J* = 8.4 Hz, 1H), 5.24 (t, *J* = 5.2 Hz, 1H), 3.94 (s, 3H), 3.94 (s, 4H), 3.17 (s, 2H), 2.32 (s, 3H), 2.23 (dt, *J* = 8.4, 4.5 Hz, 1H), 2.15 – 2.01 (m, 3H), 1.84 (d, *J* = 14.2 Hz, 2H).

^{13}C NMR (101 MHz, $CDCl_3$) δ 165.8, 153.1, 148.8, 123.6, 123.4, 112.1, 110.5, 68.0, 60.0, 56.2, 56.1, 40.6, 36.8, 25.9.

IR ν_{max} (neat) 2933-2840, 2799, 1707, 1599, 1465, 1416, 1353, 1271, 1223, 1178, 1133, 1107, cm^{-1}

HRMS (ESI) *m/z* calcd. for $C_{17}H_{24}NO_4$ $[M+H]^+$ 306.1705, found 306.1701.

1-methyl-4-(phenylsulfonyl)piperazine (1j)

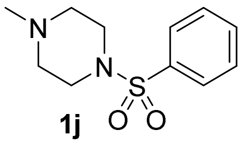

1j

Prepared according to a literature procedure.[14] To a solution of 1-methylpiperazine (0.94 mL, 8.49 mmol) in dry chloroform (5 mL), Et_3N (0.95 mL, 6.79 mmol) was added followed by benzenesulfonyl chloride (1.00 g, 5.66 mmol) and was stirred at room temperature for 2 h. The product was then extracted with $CHCl_3$ (3 x 30 mL) dried ($MgSO_4$) and concentrated *in vacuo*. Purification by silica-gel column chromatography (1:1 EtOAc/pentane) gave **1j** (1.20 g, 96%) as a white amorphous powder. Data are consistent with the literature.[14]

^{1}H NMR (400 MHz, $CDCl_3$) δ 7.76 (m, 2H), 7.56 (m, 3H), 3.03 (br. s, 4H), 2.48 (m, 4H), 2.26 (s, 3H)

^{13}C NMR (101 MHz, $CDCl_3$) δ 135.4, 133.0, 129.2, 128.0, 54.2, 46.0, 46.9

IR ν_{max} (neat) 2989 – 2803, 1449, 1379, 1289, 1170, 1000 cm^{-1}.

HRMS (ESI) *m/z* calcd. for $C_{11}H_{17}N_2O_2S$ $[M+H]^+$ 241.1011, found 241.1008.

1-methyl-4-tosylpiperazine (1k)

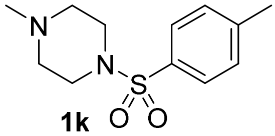

1k

Following procedure for preparation of **1j**[14] using 1-methylpiperazine (0.87 mL, 7.87 mmol), Et_3N (0.88 mL, 6.29 mmol) 4-methyl-benzenesulfonyl chloride (1.00 g, 5.25 mmol) yielded **1k** as a white amorphous powder (1.28 g, 96%). Data are consistent with the literature.[15]

^{1}H NMR (400 MHz, $CDCl_3$) δ 7.63 (d, *J* = 8.3 Hz, 2H), 7.31 (d, *J* = 8.0 Hz, 2H), 3.03 (bs, 4H), 2.49 (br. s, 4H), 2.42 (s, 3H), 2.27 (s, 3H).

^{13}C NMR (101 MHz, $CDCl_3$) δ 143.8, 132.4, 129.7, 128.0, 54.2, 46.1, 45.9, 21.6.

IR ν_{max} (neat) 2981 – 2851, 2796, 1595, 1446, 1341, 1282, 1166 cm^{-1}.

HRMS (ESI) *m/z* calcd. for $C_{12}H_{19}N_2O_2S$ $(M+H)^+$ 255.1167, found 255.1162.

1-methyl-4-((4-(trifluoromethyl)phenyl)sulfonyl)piperazine (1l)

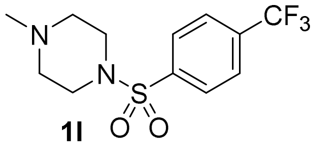

1l

Following procedure for preparation of **1j**[14] using 1-methylpiperazine (0.68 mL, 6.13 mmol), Et_3N (0.68 mL, 4.91 mmol) 4-trifluoromethyl-benzenesulfonyl chloride (1.00 g, 4.09 mmol) yielded **1l** as a white microcrystalline solid (0.64 g, 51%).

m.p. 110 - 112 °C

^{1}H NMR (400 MHz, $CDCl_3$) δ 7.84 (dd, *J* = 32.3, 8.3 Hz, 4H), 3.08 (br. s, 4H), 2.51 (br. s, 4H), 2.29 (s, 3H)

^{13}C NMR (101 MHz, $CDCl_3$) δ 139.2, 134.7 (q, *J* = 33.2 Hz), 128.4, 126.32 (q, *J* = 3.7 Hz), 123.3 (*observed as* d, *J* = 273.0 Hz), 54.1, 46.1, 45.8.

^{19}F NMR (376 MHz, $CDCl_3$) δ -63.7.

IR ν_{max} (neat) 2944 – 2803, 1454, 1405, 1357, 1319, 1289, 1170 cm^{-1}.

HRMS (ESI) *m/z* calcd. for $C_{12}H_{16}F_3N_2O_2S$ $(M+H)^+$ 309.0885, found 309.0880.

1-((4-bromophenyl)sulfonyl)-4-methylpiperazine (1m)

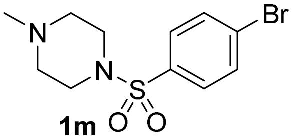

1m

Following procedure for preparation of **1j**[14] using 1-methylpiperazine (0.65 mL, 5.87 mmol), Et_3N (0.65 mL, 4.70 mmol) 4-bromobenzenesulfonyl chloride (1.00 g, 3.91 mmol) yielded **1m** as white amorphous powder (0.86 g, 69%).

^{1}H NMR (300 MHz, $CDCl_3$) δ 7.67 – 7.50 (m, 4H), 2.99 (br. s, 4H), 2.53 – 2.33 (m, 4H), 2.23 (s, 3H).

^{13}C NMR (75 MHz, $CDCl_3$) δ 134.3, 132.3, 129.3, 128.0, 53.9, 45.8, 45.56.

HRMS (ESI) *m/z* calcd. for $C_{11}H_{16}BrN_2O_2S$ $(M+H)^+$ 319.0116, found 319.0116.

IR ν_{max} (neat) 2989 – 2803, 1573, 1435, 1349, 1290, 1170, 1006 cm^{-1}.

(1S,4S)-2-methyl-5-((4-(trifluoromethyl)phenyl)sulfonyl)-2,5-diazabicyclo[2.2.1]heptane (1n)

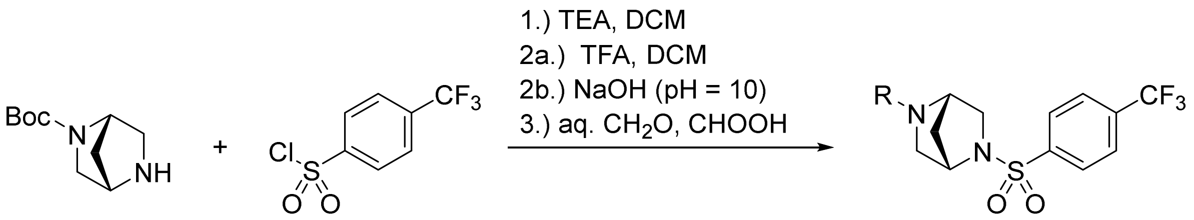

R = **Boc, 1na**
R = **H, 1nb**
R = **Me, 1n**

Following a modified procedure for preparation of **1j**[14] *tert*-butyl (1*S*,4*S*)-5-((4-(trifluoromethyl)phenyl)sulfonyl)-2,5-diazabicyclo[2.2.1]heptane-2-carboxylate **1na** was prepared using *tert*-butyl (1*S*,4*S*)-2,5-diazabicyclo[2.2.1]heptane-2-carboxylate (0.52 g, 2.62 mmol), Et_3N (0.40 mL, 2.88 mmol) and 4-bromobenzenesulfonyl chloride (0.71 g, 2.88 mmol) in DCM (20 mL). The crude viscous pale yellow liquid product **1na** (1.07 g, 100%), deemed pure by ^1H NMR and assuming quantitative yield, was subjected directly to Boc-deprotection by dissolving in DCM (50 mL) and adding trifluoroacetic acid (TFA) (15 mL, 196 mmol) dropwise at rt. After the mixture was stirred for 1 h, NaOH (1 M) was added to basify the solution until pH 10, as indicated by pH paper. The layers were separated, and the aqueous layer was extracted with DCM (2 x 20 mL). The combined organic layers were dried with $MgSO_4$, filtered, and concentrated *in vacuo* to yield **1nb** as yellow-ish oil (0.75 g, 98%). The crude was deemed pure by ^1H NMR and **1nb** (0.75 g, 2.45 mmol) was subjected directly to methylation *via* reductive amination with aqueous formaldehyde (37%, 0.73 mL, 9.79 mmol), formic acid (0.37 mL, 9.79 mmol). After the reaction mixture was refluxed for 24 h, it was allowed to cool to rt. NaOH (2 M, 50 mL) was added to basify the solution, which was extracted with TBME (3 x 20 mL). The organic layer was washed with brine (10 mL), dried ($MgSO_4$) and concentrated *in vacuo*. Purification by silica-gel column chromatography (1:1 EtOAc / pentane) gave **1n** (0.67 g, 85%, 83% over 3 steps) as a pale brown amourphous powder.

^{1}H NMR (300 MHz, $CDCl_3$) δ 7.96 (d, *J* = 8.2 Hz, 2H), 7.78 (d, *J* = 8.3 Hz, 2H), 4.30 (s, 1H), 3.58 (dd, *J* = 9.6, 1.1 Hz, 1H), 3.37 (s, 1H), 3.04 (dd, *J* = 9.6, 2.3 Hz, 1H), 2.85 (dd, *J* = 10.0, 2.4 Hz, 1H), 2.65 (dd, *J* = 10.0, 1.2 Hz, 1H), 2.34 (s, 3H), 1.75 (d, *J* = 10.1 Hz, 1H), 1.16 (d, *J* = 10.1 Hz, 1H).

^{13}C NMR (75 MHz, $CDCl_3$) δ 142.5, 134.5 (q, *J* = 33.1 Hz), 127.9, 126.4 (q, *J* = 3.7 Hz), 123.3 (q, *J* = 272.9 Hz), 63.1, 61.4, 61.2, 50.4, 40.5, 35.4.

^{19}F NMR (376 MHz, $CDCl_3$) δ -63.58.

IR ν_{max} (neat) 2944 – 2862, 2788, 1453, 1401, 1313, 1230, 1162, 1125, 1062 cm^{-1}.

HRMS (ESI) *m/z* calcd. for $C_{13}H_{16}F_3N_2O_2S$ $(M+H)^+$ 321.0885, found 321.0879.

(4-methylpiperazin-1-yl)(phenyl)methanone (1o)

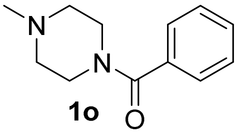

1o

Prepared according to a literature procedure.[16] Benzoyl chloride (1.16 mL, 10 mmol), Et_3N (2.78 mL, 20 mmol) and DMAP (80 mg, 0.63 mmol) were added to a solution of 1-methypiperidine (0.74 mL, 6.7 mmol) in DCM (10 ml) at 0 °C under an N_2 atmosphere. After stirring the reaction for 2 h at rt, 10% aqueous NaOH solution (5 ml) was added. The aqueous layer was extracted with DCM (3 x 10 mL). The organic layer was washed with brine (10 mL), dried ($MgSO_4$) and concentrated *in vacuo.* Purification by silica-gel column chromatography (100:1, DCM/MeOH) gave **1o** as yellow oil (1.82 g, 89%). Data are consistent with the literature.[16]

^{1}H NMR (400 MHz, $CDCl_3$) δ 7.40 (m, 5H), 3.80 (br. s, 2H), 3.44 (br. s, 2H), 2.48 (br. s, 2H), 2.34 (br. s, 2H), 2.33 (s, 3H).

^{13}C NMR (101 MHz, $CDCl_3$) δ 170.5, 135.7, 129.9, 128.6, 127.1, 54.7, 47.3, 45.8, 41.7.

IR ν_{max} (neat) 3056, 2937, 2847, 2788, 1629, 1580, 1495, 1424, 1368, 1290, 1170, 1074, 999, 783, 708 cm^{-1}.

HRMS (ESI) *m/z* calcd. for $C_{12}H_{16}N_2O$ $(M+H)^+$ 205.1341, found 205.1335

<u>Gas liquid flow photoreaction</u>

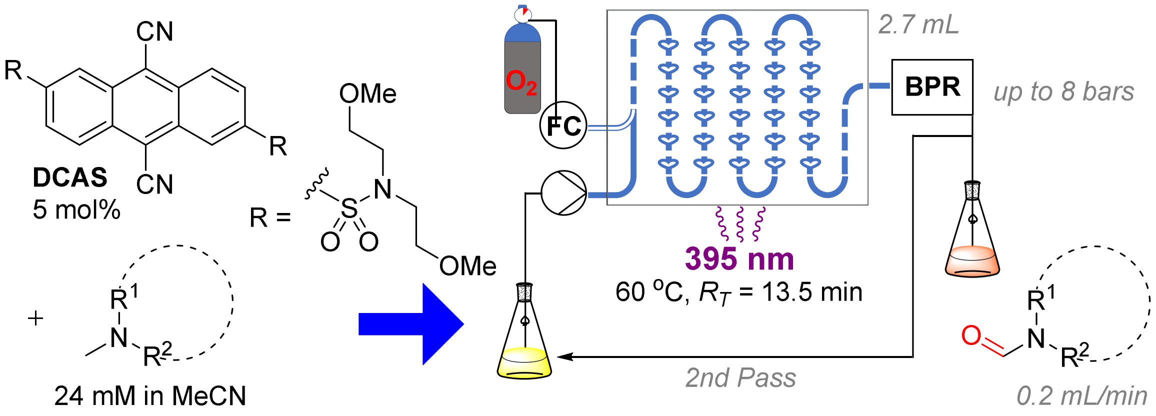

General procedure A. Gas-Liquid photoreactor start-up:

The LED module chiller system was switched on and set to 20 °C. The temperature regulator of the reactor chip was set to 60 °C. The appropriate wavelength of light (395 nm or 420 nm) and intensity was chosen *via* a smart phone application through WiFi connectivity to the reactor, and the LED lights were switched on. Before the liquid solution entered the reaction chip, O_2 gas was introduced to the system at an input flow rate of 7.0 to 8.0 mL/min (for reactions run without back pressure, 0.3 to 0.4 mL/min was set). The liquid component was introduced through the inlet tube of the liquid pump. After purging

the pump with the reaction solution, the liquid flow rate was set at a high value and a back pressure of 8 bars was applied. Then, the liquid flow rate was gradually brought down (at an increment of *ca* 0.5 mL/min) to the desired flow rate of 0.2 mL/min (for reactions run without back pressure / ambient O_2 pressure, 0.1 mL/min was set). This gas-liquid flowrate and backpressure combination gave an average measured liquid output flowrate of 0.2 mL/min.

General procedure B. Flow reaction setup

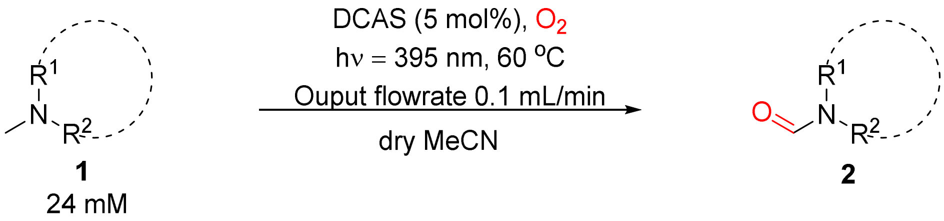

In a 25 mL volumetric flask, a solution of amine **1** (24 mM, 0.60 mmol) and DCAS catalyst (5 mol%, 18.6 mg, 0.03 mmol) was prepared in dry MeCN. The instrument startup outlined in **General Procedure A** was carried and all parameters allowed to stabilize. The flask was covered with parafilm to prevent solvent evaporation and a syringe needle was inserted to counteract the vacuum generated as the pump siphoned the reaction solution. After the gas-liquid (reaction mixture) flow rate stabilized, the reaction was started, and the crude reaction mixture was collected at the outlet tubing with a clean flask. Exactly 2.5 mL of the crude reaction mixture were collected (0.06 mmol based on starting material) for ^{1}H NMR yield analysis and 1 equiv. of 1,3,5-trimethoxybenzene (0.06 mmol, 10.1 mg) was added as an internal standard. Then the rest of the crude reaction mixture were collected, measured, and resubjected to a second pass or the solvent was evaporated *in vacuo* for product isolation.

Isolated *N*-CHO-containing compounds occur as mixtures of rotamers. For compounds which are not possible to distinguish between the individual rotamers, combined data are reported. In the following data section, an integration of 1H in ^{1}H NMR spectra corresponds to 1H of an individual rotamer. For rotamer mixtures with detectable overlapping ^{1}H NMR signals (due to the symmetry of the molecule / fragment), a "per rotamer" annotation was included. In the assignment of ^{13}C NMR spectra, all observed ^{13}C peaks are listed.

(4b*S*,9*S*)-3-methoxy-6,7,8,8a,9,10-hexahydro-5*H*-9,4b-(epiminoethano)phenanthrene-11-carbaldehyde (2a)

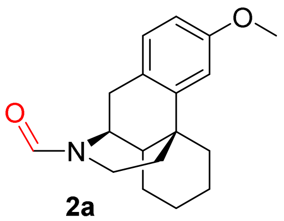

2a

Prepared according to **General Procedure B** with ambient O_2 pressure and R_T = 27 min. After 1 pass through the flow photoreactor, 15 mL of crude reaction mixture was collected and concentrated *in vacuo* (46% ^{1}H NMR yield). Purification by silica-gel column chromatography (a gradient of 1:1 EtOAc / pentane to pure EtOAc) **2a** as viscous yellow oil (37.0 mg, 36%) as a 1:1 mixture of rotamers. Data are consistent with the literature.[6,7]

^{1}H NMR (400 MHz, $CDCl_3$) rotamer 1 / rotamer 2: δ 8.14/7.02 (s, 1H per rotamer) , 7.02 (t, *J* = 7.5 Hz, 2 X 1H per rotamer), 6.84 (d, *J* = 2.1 Hz, 2 X 1H per rotamer), 6.73 (dd, *J* = 8.4, 2.5 Hz, 2 × 1H per rotamer), 4.70 – 4.48 (m, 1H), 4.15 (dd, *J* = 13.7, 4.7 Hz, 1H), 3.79 (s, 2 × 6H per rotamer), 3.70 – 3.65 (m, 1H), 3.27 (dd, *J* = 13.4, 4.5 Hz, 1H), 3.16 (td, *J* = 17.6, 5.9 Hz, 2H), 2.95 (td, *J* = 13.1, 3.6 Hz, 1H), 2.66 (t, *J* = 18.0 Hz, 2H), 2.46 (td, *J* = 13.3, 4.0 Hz, 1H), 2.40 – 2.26 (m, 2H), 1.73 – 1.40 (m, 12H), 1.39 – 1.24 (m, 6H), 1.18 – 1.03 (m, 2H);

^{13}C NMR (101 MHz, $CDCl_3$) rotamer 1 and rotamer 2: δ 160.7, 160.6, 158.7, 158.6, 140.2, 140.2, 129.2, 129.1, 128.2, 127.6, 111.5, 111.4, 111.4, 111.3, 55.2, 53.8, 46.4, 45.0, 43.8, 42.1, 41.2, 40.9, 36.6, 36.5, 35.0, 32.3, 30.8, 26.4, 26.2, 26.2, 22.0, 21.9;

IR ν_{max} (neat) 2929-2855, 1659, 1610, 1576, 1498, 1431, 1326 1267, 1241 cm^{-1};

HRMS (ESI) *m/z* calcd. for $C_{18}H_{24}NO_2$ $(M+H)^+$ 286.1807, found 286.1804.

(1R,3r,5S)-3-hydroxy-8-azabicyclo[3.2.1]octane-8-carbaldehyde (2b)

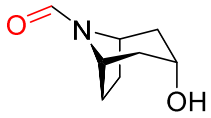

2b

Prepared according to **General Procedure B**. After 1 pass through the flow photoreactor, 15 mL of crude reaction mixture was collected and concentrated *in vacuo* (61% ^{1}H NMR yield). Purification by silica-gel column chromatography (a gradient of 1:1 EtOAc / pentane to 100% EtOAc) afforded **2b** as a pale yellow amorphous solid (30.9 mg, 55%).

IR ν_{max} (neat) 3339, 2989-2866, 1629 cm^{-1};

^{1}H NMR (400 MHz, $CDCl_3$) δ 8.09 (s, 1H), 4.56 (s, 1H), 4.16 (t, *J* = 4.6 Hz, 1H), 4.01 (d, *J* = 3.0 Hz, 1H), 2.30 – 2.22 (m, 2H), 2.10 – 1.79 (m, 6H);

^{13}C NMR (75 MHz, $CDCl_3$) rotamer 1 and rotamer 2: δ 157.4, 65.2, 54.0, 49.1, 41.4, 38.9, 28.3, 27.6;

HRMS (ESI) *m/z* calcd. for $C_8H_{13}NO_2$ $[M+H]^+$ 156.1019, found 156.1018.

(1R,5S)-8-azabicyclo[3.2.1]octane-8-carbaldehyde (2c)

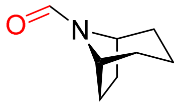

2c

Prepared according to **General Procedure B**. After 1 pass through the flow photoreactor, 15 mL of crude reaction mixture was collected and concentrated *in vacuo* (59% ^{1}H NMR yield). Purification by silica-gel column chromatography (a gradient of 1:1 EtOAc / pentane to 100% EtOAc) afforded **2c** as brown oil (29.1 mg, 58%). Data are consistent with the literature.[17]

^{1}H NMR (400 MHz, $CDCl_3$) δ 8.08 (s, 1H), 4.54 (bs, 1H), 3.99 (bs, 1H), 1.95 – 1.88 (m, 2H), 1.80 – 1.69 (m, 4H), 1.66 – 1.46 (m, 4H);

^{13}C NMR (101 MHz, $CDCl_3$) δ 157.4, 55.3, 50.3, 33.7, 31.0, 28.0, 27.5, 17.1.

IR ν_{max} (neat) 2947 – 2877, 1648, 1427, 1386, 1312, 1252, 1200, 1118, 1080 cm^{-1};

HRMS (ESI) *m/z* calcd. for $C_8H_{14}NO$ $[M+H]^+$ 140.1075, found 140.1070.

(1R,3r,5S)-8-formyl-8-azabicyclo[3.2.1]octan-3-yl (S)-3-hydroxy-2-phenylpropanoate (2d)

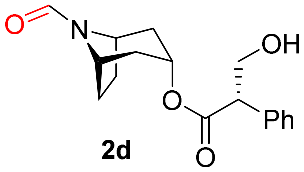

2d

Prepared according to **General Procedure B**. After 1 pass through the flow photoreactor, 13 mL of crude reaction mixture was collected and concentrated *in vacuo* (46% ^{1}H NMR yield). Purification by silica-gel column chromatography (a gradient of 1:1 EtOAc / pentane to 100% EtOAc) afforded **2d** as a viscous yellow oil (54.9 mg, 58%) and as a 1:1 mixture of rotamers. Data are consistent with the literature[6,7,18]

^{1}H NMR (400 MHz, $CDCl_3$) rotamer 1 / rotamer 2: δ 8.06/8.04 (s, 1H per rotamer), 7.38 – 7.29 (m, 2 X 3H per rotamer), 7.27 – 7.24 (m, 2 X 2H per rotamer), 5.16 (t, *J* = 4.8 Hz, 2 X 1H per rotamer), 4.60 – 4.49 (bm, 1H), 4.45 – 4.33 (bm, 1H), 4.24 – 4.09 (m, 2 X 1H per rotamer), 4.03 – 3.92 (bm, 1H), 3.88 – 3.77 (m, *overlapping peaks*, 1H and 2 X 2H per rotamer), 2.14 – 1.59 (m, 2 X 8H per rotamer);

^{13}C NMR (101 MHz, $CDCl_3$) δ 172.3 (2x), 157.5, 135.5 (2x), 129.2 (2x), 128.2 (2x), 128.1 (2x), 128.1, 68.38, 64.3, 64.2, 54.5, 54.4, 53.5, 53.4, 48.7, 48.6, 38.6, 38.3, 36.0, 35.7, 28.0, 27.5, 27.3, 26.8.

IR ν_{max} (neat) 3274, 3064-2960, 1715, 1659, 1462, 1386, 1349, 1323, 1778, 1111 cm^{-1};

HRMS (ESI) *m/z* calcd. for $C_{17}H_{22}NO_4$ $[M+H]^+$ 304.1543, found 304.1548

(1R,2R,4S,5S,7s)-9-formyl-3-oxa-9-azatricyclo[3.3.1.02,4]nonan-7-yl (S)-3-hydroxy-2-phenylpropanoate (**2e**)

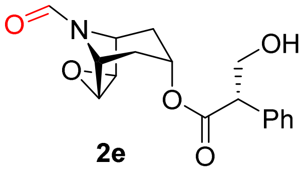

2e

Prepared according to **General Procedure B**. After 2 passes through the flow photoreactor, 17 mL of crude reaction mixture was collected and concentrated *in vacuo* (46% ^{1}H NMR yield). Purification by silica-gel column chromatography (a gradient of 1:1 EtOAc / pentane to 100% EtOAc) afforded **2e** as a viscous yellow oil (50.0 mg, 36%) and as a 1:1 mixture of rotamers. Data are consistent with the literature.[6,7]

^{1}H NMR (400 MHz, $CDCl_3$) rotamer 1 / rotamer 2: δ 7.96/7.9j (s, 1H per rotamer), 7.39 – 7.27 (m, 2 X 3H per rotamer), 7.23 – 7. (m, 2 X 2H per rotamer), 5.12 (t, *J* = 4.9 Hz, 2 x 1H per rotamer), 4.57 (bs, 1H), 4.43 (bs, 1H), 4.22 – 4.12 (m, 2 X 1H), 3.92 (bd, *J* = 2.0 Hz, 1H), 3.80 (m, *overlapping peaks*, 1H and 2 X 2H per rotamer), 3.29 (t, *J* = 3.4 Hz, 2 X 1H per rotamer), 2.56 (dd, *J* = 15.8, 3.2 Hz, 2 X 1H per rotamer), 2.18 – 1.90 (m, 2 X 2H per rotamer), 1.84 (t, *J* = 14.9 Hz, 2 X 1H per rotamer), 1.61 (t, *J* =14.8, Hz, 2 X 1H per rotamer);

^{13}C NMR (101 MHz, $CDCl_3$) δ 171.7, 162.2 (2x), 135.5 (2x), 129.1 (2x), 128.2 (2x), 128.0 (2x), 66.7, 63.9 (2x), 54.3 (2x), 53.7, 53.6, 52.0, 51.5 (2x), 51.5, 51.0, 49.7, 49.6, 32.3, 32.0, 30.7, 30.5;

IR ν_{max} (neat) cm^{-1} : 3354, 3060, 2978-2881, 2739, 2602, 2531, 2497, 1722, 1655;

HRMS (ESI) *m/z* calcd. for $C_{17}H_{19}NO_5Na^+$ $[M+Na]^+$ 340.1161, found 340.1158.

(1R,3r,5S)-3-((methyldiphenylsilyl)oxy)-8-azabicyclo[3.2.1]octane-8-carbaldehyde (2f)

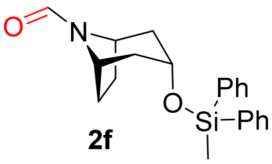

2f

Prepared according to **General Procedure B**. After 1 pass through the flow photoreactor, 10 mL of crude reaction mixture was collected and concentrated *in vacuo* (67% ^{1}H NMR yield). Purification by silica-gel column chromatography (a gradient of 1:1 EtOAc / pentane to 100% EtOAc) afforded **2f** as a viscous brown oil (26.1 mg, 31%) and as a 1:1 mixture of rotamers;

^{1}H NMR (400 MHz, $CDCl_3$) δ 8.07 (s, 1H), 7.78 – 7.50 (m, 4H), 7.45 – 7.29 (m, 6H), 4.57 (bs, 1H), 4.19 (t, *J* = 4.2 Hz, 1H), 4.00 (bs, 1H), 2.54 – 2.32 (bm, 2H), 2.11 – 1.80 (m, 6H), 0.71 – 0.60 (s, 3H);

^{13}C NMR (101 MHz, $CDCl_3$) δ 157.4, 134.4, 134.3, 130.1, 128.1, 66.4, 54.3, 49.4, 41.8, 39.1, 28.5, 27.8, -2.7;

IR ν_{max} (neat) 3071 – 2918, 1662, 1427, 1375, 1312, 1256, 1222, 1162, 1080 cm^{-1}

HRMS (ESI) *m/z* calcd. for $C_{21}H_{26}NO_2Si$ $[M+H]^+$ 352.1733, found 352.1731.

(1R,3r,5S)-8-formyl-8-azabicyclo[3.2.1]octan-3-yl benzoate (2g)

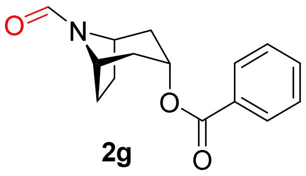

2g

Prepared according to **General Procedure B**. After 1 pass through the flow photoreactor, 10 mL of crude reaction mixture was collected and concentrated *in vacuo* (73% ^1H NMR yield). Purification by silica-gel column chromatography (a gradient of 1:1 EtOAc / pentane to 100% EtOAc) afforded **2g** as viscous pale yellow viscous oil (42.5 mg, 68%). Data are consistent with the literature.[19]

IR ν_{max} (neat) 2952 – 2878, 1722, 1640, 1438, 1386, 1312, 1226, 1159, 1073, 1036 cm^{-1};

^{1}H NMR (400 MHz, $CDCl_3$) δ 8.15 (s, 1H), 8.09 – 7.97 (m, 2H), 7.61 – 7.52 (m, 1H), 7.47 (t, *J* = 7.7 Hz, 2H), 5.40 (t, *J* = 4.8 Hz, 1H), 4.67 (bs, 1H), 4.12 (bs, 1H), 2.28 – 2.01 (m, 8H);

^{13}C NMR (101 MHz, $CDCl_3$) δ 165.8, 157.6, 133.3, 130.5, 129.6, 128.8, 68.4, 53.7, 48.8, 38.8, 36.2, 28.5, 27.7.

HRMS (ESI) *m/z* calcd. for $C_{15}H_{18}NO_3$ $[M+H]^+$ 260.1287, found 260.1282.

(1R,3r,5S)-8-formyl-8-azabicyclo[3.2.1]octan-3-yl 4-(trifluoromethyl)benzoate (2h)

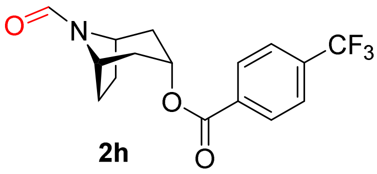

2h

Prepared according to **General Procedure B**. After 1 pass through the flow photoreactor, 12 mL of crude reaction mixture was collected and concentrated *in vacuo* (56% ^1H NMR yield). Purification by silica-gel column chromatography (a gradient of 1:1 EtOAc / pentane to 100% EtOAc) afforded **2h** as a pale yellow solid (51.3 mg, 54%);

m.p. 122-124 °C

IR ν_{max} (neat) 2959 – 2870, 1717, 1647, 1513, 1431, 1405, 1323, 1274, 1103 cm^{-1};

^{1}H NMR (400 MHz, $CDCl_3$) δ 8.18 (s, 1H), 8.13 (d, *J* = 8.1 Hz, 2H), 7.75 (d, *J* = 8.2 Hz, 2H), 5.43 (t, *J* = 4.8 Hz, 1H), 4.70 (bs, 1H), 4.15 (bs, 1H), 2.34 – 1.99 (m, 8H);

^{13}C NMR (101 MHz, $CDCl_3$) δ 164.6, 157.7, 134.9 (*observed as* d, *J* = 32.7 Hz), 133.6, 130.0, 125.8 (q, *J* = 3.7 Hz), 123.8 (*observed as* d, *J* = 272.8 Hz), 69.1, 53.7, 48.9, 38.8, 36.2, 28.5, 27.7;

^{19}F NMR (377 MHz, $CDCl_3$) δ -63.68;

HRMS (ESI) *m/z* calcd. for $C_{16}H_{17}F_3NO_3$ $[M+H]^+$ 328.1161, found 328.1155.

(1R,3r,5S)-8-formyl-8-azabicyclo[3.2.1]octan-3-yl 3,4-dimethoxybenzoate (2i)

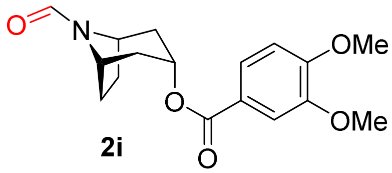

2i

Prepared according to **General Procedure B**. After 1 pass through the flow photoreactor, 15 mL of crude reaction mixture was collected and concentrated *in vacuo* (58% ^{1}H NMR yield). Purification by silica-gel column chromatography (a gradient of 1:1 EtOAc / pentane to 100% EtOAc) afforded **2i** as a pale yellow solid (63.7 mg, 55%)[20]

m.p. 140-141 (lit 140-141)[20]

IR ν_{max} (neat) 2956 – 2840, 1703, 1654, 1599, 1416, 1349, 1267, 1218, 1178, 1133, 1103, 1036 cm^{-1};

^{1}H NMR (300 MHz, $CDCl_3$) δ 8.18 (s, 1H), 7.64 (dd, *J* = 8.4, 2.0 Hz, 1H), 7.54 (d, *J* = 2.0 Hz, 1H), 6.92 (d, *J* = 8.5 Hz, 1H), 5.38 (t, *J* = 4.4 Hz, 1H), 4.69 (bs, 1H), 4.16 (bs, 1H), 3.95 (s, 3H), 3.94 (s, 3H), 2.31 – 2.00 (m, 8H);

^{13}C NMR (101 MHz, $CDCl_3$) δ 165.5, 157.5, 153.2, 148.9, 123.3, 122.8, 112.0, 110.4, 68.0, 56.1, 56.0, 53.6, 48.7, 38.7, 36.1, 28.4, 27.6.

HRMS (ESI) *m/z* calcd. for $C_{17}H_{22}NO_5$ $(M+H)^+$ 320.1498; found 320.1497.

4-(phenylsulfonyl)piperazine-1-carbaldehyde (2j)

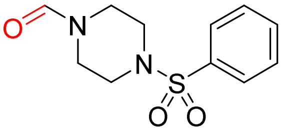

2j

Prepared according to **General Procedure B**. After 2 passes through the flow photoreactor, 6 mL of crude reaction mixture was collected and concentrated *in vacuo* (52% ^{1}H NMR yield). Purification by silica-gel column chromatography (1:1 EtOAc / pentane) **2j** as a pale yellow viscous oil (15.7 mg, 43%).

IR ν_{max} (neat) 2922 – 2858, 1673, 1446, 1401, 1349, 1278, 1237, 1170, 1022 cm^{-1};

^{1}H NMR (400 MHz, $CDCl_3$) δ 7.97 (s, 1H), 7.77 – 7.72 (m, 2H), 7.66 – 7.52 (m, 3H), 3.69 – 3.61 (m, 2H), 3.50 – 3.41 (m, 2H), 3.09 – 2.99 (m, 4H);

^{13}C NMR (101 MHz, $CDCl_3$) δ 160.75, 135.55, 133.47, 129.46, 127.80, 46.70, 45.66, 45.12, 39.47;

HRMS (ESI) *m/z* calcd. for $C_{11}H_{15}N_2O_3S$ $[M+H]^+$ 255.0798; found 255.0802

4-tosylpiperazine-1-carbaldehyde (2k)

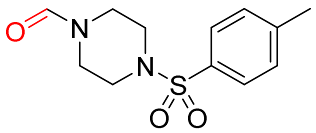

2k

Prepared according to **General Procedure B** with ambient O_2 pressure and R_T = 27 min. After 2 passes through the flow photoreactor, 10 mL of crude reaction mixture was collected and concentrated *in vacuo* (45% ^{1}H NMR yield). Purification by silica-gel column chromatography (1:1 EtOAc / pentane) **2k** as a viscous pale yellow viscous oil (27.7 mg, 43%).

IR ν_{max} (neat) 2956 – 2926, 1670, 1599, 1435, 1401, 1334, 1278, 1237, 1192, 1118, 1047 cm^{-1};

^{1}H NMR (400 MHz, $CDCl_3$) δ 7.97 (s, 1H), 7.63 (d, *J* = 8.3 Hz, 2H), 7.34 (d, *J* = 8.0 Hz, 2H), 3.77 – 3.56 (m, 2H), 3.53 – 3.41 (m, 2H), 3.18 – 2.92 (m, 4H), 2.44 (s, 3H);

^{13}C NMR (101 MHz, $CDCl_3$) δ 160.71, 144.41, 132.40, 130.04, 127.84, 46.69, 45.64, 45.08, 39.42, 21.68;

HRMS (ESI) *m/z* calcd. for $C_{12}H_{17}N_2O_3S$ $[M+H]^+$ 269.0960, found 269.0959.

4-((4-(trifluoromethyl)phenyl)sulfonyl)piperazine-1-carbaldehyde (2l)

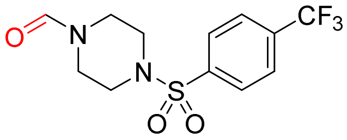

2l

Prepared according to **General Procedure B** with ambient O_2 pressure and R_T = 27 min. After 2 passes through the flow photoreactor, 10 mL of crude reaction mixture was collected and concentrated *in vacuo* (55% ^{1}H NMR yield). Purification by silica-gel column chromatography (1:1 EtOAc / pentane) afforded **2l** as a white microcrystaline solid (38.9 mg, 50%).

m.p. 187 – 189 °C

IR ν_{max} (neat) 2929 – 2855, 1673, 1442, 1405, 1326, 1278, 1170, 1133 cm^{-1};

^{1}H NMR (400 MHz, $CDCl_3$) δ 7.98 (s, 1H), 7.88 (d, *J* = 8.4 Hz, 2H), 7.83 (d, *J* = 8.4 Hz, 2H), 3.75 – 3.59 (m, 2H), 3.56 – 3.38 (m, 2H), 3.15 – 3.00 (m, 4H);

^{13}C NMR (101 MHz, $CDCl_3$) δ 160.7, 139.4, 135.2 (q, J = 33.4 Hz), 128.3, 126.7 (q, J = 3.7 Hz). 123.2 (*observed as* d, J = 273.0 Hz), 46.6, 45.6, 45.1, 39.4;

^{19}F NMR (376 MHz, $CDCl_3$) δ -63.7;

HRMS (ESI) *m/z* calcd. for $C_{12}H_{14}F_3N_2O_3S$ $[M+H]^+$ 323.0677, found 323.0675.

4-((4-bromophenyl)sulfonyl)piperazine-1-carbaldehyde (2m)

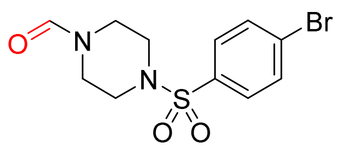

2m

Prepared according to **General Procedure B**. After 12 h of recycling through the flow photoreactor, 7 mL of crude reaction mixture was collected and concentrated *in vacuo* (21% ^{1}H NMR yield, 47% returned starting material). Purification by silica-gel column chromatography (1:1 EtOAc / pentane) afforded **2m** as a pale light-brown amorphous powder (10.6 mg, 19%).

IR ν_{max} (neat) 2922 – 2863, 1669, 1572, 1438, 1390, 1353, 1278, 1006 cm^{-1};

^{1}H NMR (400 MHz, $CDCl_3$) δ 7.98 (s, 1H), 7.72 – 7.67 (m, 2H), 7.65 – 7.52 (m, 2H), 3.72 – 3.59 (m, 2H), 3.51 – 3.45 (m, 2H), 3.13 – 2.99 (m, 4H).

^{13}C NMR (101 MHz, $CDCl_3$) δ 160.74, 134.65, 132.81, 129.25, 128.69, 46.62, 45.62, 45.07, 39.44;

HRMS (ESI) *m/z* calcd. for $C_{11}H_{14}BrN_2O_3S$ $[M+H]^+$ 332.9909, found 332.9907.

(1S,4S)-5-((4-(trifluoromethyl)phenyl)sulfonyl)-2,5-diazabicyclo[2.2.1]heptane-2-carbaldehyde (2n)

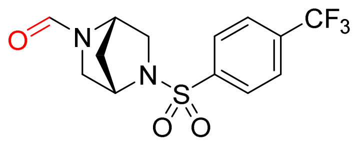

Prepared according to **General Procedure B**. After 2 passes on the flow photoreactor, 6.8 mL of crude reaction mixture was collected and concentrated *in vacuo* (30% ^{1}H NMR yield). Purification by silica-gel column chromatography (a gradient of 1:1 EtOAc / pentane to 100% EtOAc) afforded **2m** as a pale-yellow oil (9.3 mg, 17%,) and as 1:1 mixture of rotamers.

^{1}H NMR (400 MHz, $CDCl_3$) rotamer 1 / rotamer 2: δ 8.30/8.07 (s, 1H per rotamer), 7.98 (d, J = 8.2 Hz, 2 X 2H per rotamer), 7.82 (d, J = 8.3 Hz, 2 X 2H per rotamer), 4.85 (s, 1H), 4.62 (s, 1H), 4.61 (s, 1H), 4.46 (s, 1H), 3.69 – 3.18 (m, 2 X 4H per rotamer), 1.97 – 1.18 (m, 2 X 2H per rotamer).

^{13}C NMR (101 MHz, $CDCl_3$) δ 160.8, 160.0, 127.9, 127.8, 126.7 (2x), 59.6, 57.8, 55.9, 55.0, 54.6, 53.6, 53.3, 52.0, 37.1, 36.1. Quaternary aromatic carbons and -CF_3 were not observed.

^{19}**F NMR** (376 MHz, $CDCl_3$) δ -63.63.

IR ν_{max} (neat) 2952 – 2892, 1659, 1404, 1326, 1170, 1133, 1062, cm^{-1};

HRMS (ESI) *m/z* calcd. for $C_{13}H_{14}F_3N_2O_3S$ $[M+H]^+$ 335.0677, found 335.0672

4-benzoylpiperazine-1-carbaldehyde (2o)

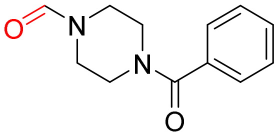

2o, 33% (40%)

Prepared according to **General Procedure B**. After 2 passes on the flow photoreactor, 11 mL of crude reaction mixture was collected and concentrated *in vacuo* (40% ^{1}H NMR yield). Purification by silica-gel column chromatography (a gradient of 1:1 EtOAc / pentane to 100% EtOAc) afforded **2o** as a viscous yellow oil (19.0 mg, 33%). Data are consistent with the literature.[21]

IR n_{max} (neat) 2959 – 2858, 1700, 1636, 1494, 1431, 1282, 1241, 1155, 1003 cm^{-1};

^{1}**H NMR** (400 MHz, $CDCl_3$) δ 8.10 (s, 1H), 7.52 – 7.30 (m, 5H), 3.84 – 3.23 (bm, 8H).

^{13}**C NMR** (101 MHz, $CDCl_3$) δ 170.9, 161.1, 135.1, 130.4, 128.8, 127.2, 45.7, 40.3;

HRMS (EI, 70 eV) *m/z* calcd. for $C_{12}H_{15}N_2O_2$ $(M)^{+\cdot}$ 218.1050, found 218.10502

4-hydroxypiperidine-1-carbaldehyde (2p)

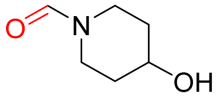

2p

Prepared according to **General Procedure B**. After 2 passes through the flow photoreactor, 10 mL of crude reaction mixture was collected and concentrated *in vacuo* (39% ^{1}H NMR yield). Purification by silica-gel column chromatography (a gradient of 1:1 EtOAc / pentane to 100% EtOAc) afforded **2a** as viscous yellow oil (9.4 mg, 30%);

IR ν_{max} (neat) 3392, 2948 – 2870, 1654, 1446, 1401, 1368, 1267, 1207, 1111, 1073 cm^{-1};

^{1}**H NMR** (300 MHz, $CDCl_3$) δ 8.02 (s, 1H), 4.12 – 3.85 (m, 2H), 3.68 – 3.53 (m, 1H), 3.20 (ddd, J = 17.4, 8.7, 4.3 Hz, 2H), 1.97 – 1.80 (m, 2H), 1.53 (dddd, J = 21.5, 13.0, 8.5, 4.2 Hz, 3H);

^{13}**C NMR** (75 MHz, $CDCl_3$) δ 160.9, 67.4, 43.1, 36.8, 34.7, 33.5;

HRMS (EI, 70 eV) *m/z* calcd. for $C_6H_{11}NO_2$ $(M)^{+\cdot}$ 129.0790, found 129.0783.

***N*-((2*S*,3*R*,4*S*,6*R*)-2-(((3*R*,4*S*,5*S*,6*R*,7*R*,9*R*,11*R*,12*R*,13*S*,14*R*)-14-ethyl-7,12,13-trihydroxy-4-(((2*R*,4*R*,5*S*,6*S*)-5-hydroxy-4-methoxy-4,6-dimethyltetrahydro-2*H*-pyran-2-yl)oxy)-3,5,7,9,11,13-hexamethyl-2,10-dioxooxacyclotetradecan-6-yl)oxy)-3-hydroxy-6-methyltetrahydro-2*H*-pyran-4-yl)-*N*-methylformamide (De(*N*-methyl)-*N*-formyl erythromycin A, 2r)**

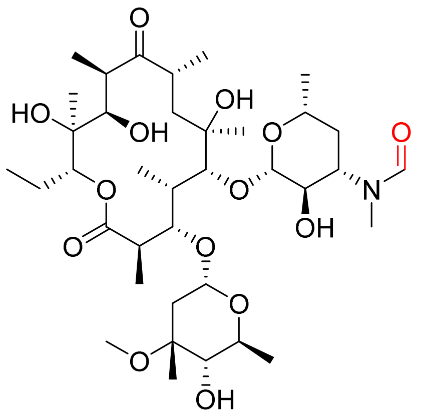

Prepared according to **General Procedure B** with erythromycin (12 mM, 0.30 mmol) as starting material, ambient O_2 pressure and R_T = 27 min. After 2 passes through the flow photoreactor, exactly 2.5 mL of crude reaction mixture was collected for yield determination (61% ^{1}H NMR yield) and another 5 mL of crude reaction mixture was collected and concentrated *in vacuo* to approximately half its volume (61% ^{1}H NMR yield). Then the mixture was filtered through a syringe filter before injecting to preparative HPLC for purification which afforded **2r** as white amorphous solid as 0.39:1 mixture of rotamers (22.4 mg, 50%). Data are consistent with the literature.[22]

^{1}H NMR (400 MHz, CD_3CN) rotamer 1 / rotamer 2: δ 8.03/8.01 (s, 1H per rotamer), 4.98 (dd, *J* = 10.7, 2.4 Hz, 1H per rotamer), 4.86 (d, *J* = 4.5 Hz, 1H per rotamer), 4.51/4.50 (d, *overlapping peaks, J* = 7.4/7.0 Hz, 1H per rotamer), 4.07 – 3.97 (m, 1H per rotamer), 3.85 (d, *J* = 8.8 Hz, 1H per rotamer), 3.31 (s, 3H), 3.30 (s, 3H), 2.84 (s, 3H), 2.71 (s, 3H), 1.36 (s, 3H per rotamer), 1.26 – 1.01 (apt.m, 9 X 3H per rotamer), 0.82 (t, *J* = 7.4 Hz, 3H per rotamer).

^{13}C NMR (101 MHz, CD_3CN) δ 221.5, 176.7, 164.5, 164.3, 103.6, 103.3, 97.1, 97.0, 84.5, 80.2, 78.9, 78.8, 77.5, 75.6, 75.4, 73.8, 73.7, 73.6, 71.2, 70.9, 70.0, 68.7, 68.5, 66.4 (2x), 59.9, 53.4, 49.8, 45.7, 44.7, 39.1, 37.2, 35.5, 30.6, 26.7, 25.4, 22.1, 21.50, 21.4, 21.3, 19.0, 18.6, 17.0, 16.1, 12.2, 11.1, 10.0.

HRMS (ESI) *m/z* calcd. for $C_{37}H_{65}NO_{14}$ $[M+Na]^+$ 770.4297, found 770.4306.

***N*-((2*S*,3*R*,4*S*,6*R*)-2-(((3*R*,4*S*,5*S*,6*R*,7*R*,9*R*,11*R*,12*R*,13*S*,14*R*)-14-ethyl-12,13-dihydroxy-4-(((2*R*,4*R*,5*S*,6*S*)-5-hydroxy-4-methoxy-4,6-dimethyltetrahydro-2*H*-pyran-2-yl)oxy)-7-methoxy-3,5,7,9,11,13-hexamethyl-2,10-dioxooxacyclotetradecan-6-yl)oxy)-3-hydroxy-6-methyltetrahydro-2*H*-pyran-4-yl)-*N*-methylformamide (De(*N*-methyl)-*N*-formyl clarithromycin, 2s)**

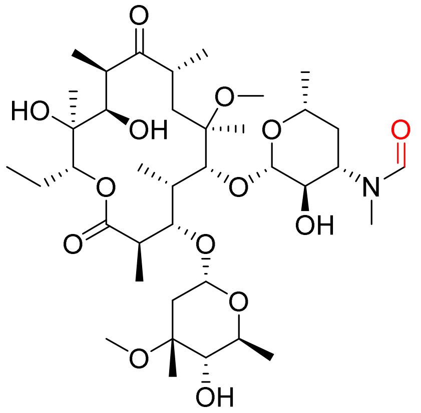

Prepared according to **General Procedure B** with clarithromycin (6 mM, 0.15mmol) as starting material. After 2 passes through the flow photoreactor, exactly 5.0 mL of crude reaction mixture was collected for yield determination (44% ^{1}H NMR yield) and another 2.5 mL of crude reaction mixture was collected and concentrated *in vacuo* to approximately half its volume (44% ^{1}H NMR yield). Then the mixture was filtered through a syringe filter before injecting to preparative HPLC for purification which afforded **2s** as white amorphous solid and as 0.36:1 mixture of rotamers (3.4mg, 30%) ; data are consistent with the literature.[22]

^{1}H NMR (400 MHz, CD_3CN) rotamer 1 / rotamer 2: δ 8.03/8.01 (s, 1H per rotamer), 4.99 (dd, *J* = 11.0, 2.3 Hz, 1H per rotamer), 4.85 (d, *J* = 4.4 Hz, 1H per rotamer), 4.50/4.49 (d, *overlapping peaks*, *J* = 7.3/7.2 Hz, 1H per rotamer), 4.12 – 3.97 (m, 1H per rotamer), 3.32 (s, 3H), 3.31 (s, 3H), 2.97 (s, 3H), 2.96 (s, 3H), 2.84 (s, 3H), 2.71 (s, 3H), 1.36 (s, 3H per rotamer), 1.25 – 1.00 (apt.m, 9 X 3H per rotamer), 0.81 (t, *J* = 7.4 Hz, 3H per rotamer).

^{13}C NMR (101 MHz, CD_3CN) δ 221.8, 177.2, 164.3, 103.2, 96.9, 81.2, 79.3, 78.8, 78.7, 78.6, 77.4, 75.1, 73.7 (2x), 70.9, 70.1, 68.3, 66.6, 59.9, 51.0, 50.9, 49.8, 46.0, 45.8, 40.2, 39.6, 38.3, 37.3, 35.5, 25.4, 21.9, 21.5, 20.4, 19.2, 18.1, 16.6, 16.2, 12.6, 11.0, 9.8 (2x).

HRMS (ESI) *m/z* calcd. for $C_{37}H_{65}NO_{14}$ $[M+Na]^+$ 784.4454, found 784.4469

***N*-((2*S*,3*R*,4*S*,6*R*)-2-(((3*R*,4*S*,5*S*,6*R*,7*R*,9*R*,11*S*,12*R*,13*S*,14*R*,*E*)-14-ethyl-7,12,13-trihydroxy-4-(((2*R*,4*R*,5*S*,6*S*)-5-hydroxy-4-methoxy-4,6-dimethyltetrahydro-2*H*-pyran-2-yl)oxy)-10-(((2-methoxyethoxy)methoxy)imino)-3,5,7,9,11,13-hexamethyl-2-oxooxacyclotetradecan-6-yl)oxy)-3-hydroxy-6-methyltetrahydro-2*H*-pyran-4-yl)-*N*-methylformamide (De(*N*-methyl)-*N*-formyl roxithromycin, 2t)**

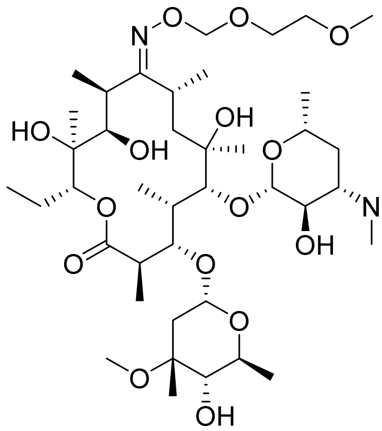

Prepared according to **General Procedure B** with roxithromycin (12 mM, 0.30 mM) as starting material. After 2 passes through the flow photoreactor, exactly 2.5 mL of crude reaction mixture was collected for yield determination (24% ^{1}H NMR yield) and another 2.5 mL of crude reaction mixture was collected and concentrated *in vacuo* to approximately half its volume. Then the mixture was filtered through a syringe filter before injecting to preparative HPLC for purification which afforded **2t** as white amorphous solid and as 0.43:1 mixture of rotamers (3.8 mg, 15%); data are consistent with the literature.[22]

^{1}H NMR (400 MHz, CD_3CN) rotamer 1 / rotamer 2: δ 8.03/8.01 (s, 1H per rotamer), 5.15 (d, *J* = 7.6 Hz, 2 X 1H per rotamer), 5.10 (d, *J* = 7.6 Hz, 2 X 1H per rotamer), 5.04 (dd, *J* = 10.9, 2.3 Hz, 1H per rotamer), 4.85 (d, *J* = 4.6 Hz, 1H per rotamer), 4.50 (d, *J* = 7.3 Hz, 1H per rotamer), 4.08 – 3.99 (m, 1H per rotamer), 3.84 (d, *J* = 9.3 Hz 1H per rotamer), 3.71 – 3.65 (m, 2H per rotamer), 3.50 – 3.46 (m, 2H per rotamer), 3.33 (s, 3H per rotamer), 3.31 (s, 3H), 3.30 (s, 3H), 2.84 (s, 3H), 2.71 (s, 3H), 1.44 (s, 3H per rotamer), 1.21 (apt.m, 2 X 3H per rotamer), 1.14 (apt.m, 5 x 3H per rotamer), 1.04 (d, *J* = 5.3 Hz, 3H per rotamer), 1.03 (d, *J* = 6.0 Hz, 3H per rotamer), 0.81 (t, *J* = 7.4 Hz, 3H per rotamer).

^{13}C NMR (101 MHz, CD_3CN) δ 176.3, 173.5, 164.4, 164.2, 103.6, 103.3, 98.1, 97.0 (2x), 84.8, 84.7, 80.3, 78.9, 78.8, 77.6, 75.6, 75.1, 73.8, 73.7, 72.6, 71.3, 71.0, 68.8, 68.7, 68.5, 68.4, 66.3, 66.2, 59.9, 59.1, 53.5, 49.8, 45.6 (2x), 40.0, 38.4, 37.3, 36.0, 35.7, 35.6, 34.2, 30.6, 27.8, 26.9, 25.4, 22.1, 21.5 (2x), 21.4, 19.2, 19.1, 18.9, 16.9, 16.4, 15.2, 11.1, 10.0(2x), 9.7.

HRMS (ESI) *m/z* calcd. for $C_{41}H_{74}N_2O_{16}$ $[M+Na]^+$ 873.4936, found 873.4947

8. UV-vis Absorption, Steady-State and Time-Resolved Emission Investigations

UV-Vis Spectroscopy of Dicyanoanthracenes

UV-visible absorption spectra were measured using a PerkinElmer Lambda 25 UV/VIS spectrophotometer. Solutions (1 x 10^{-5} M in MeCN) of **DCA** and **DCAS** (**9b**) were employed in direct comparisons, and samples were measured without degassing (Figure S11).

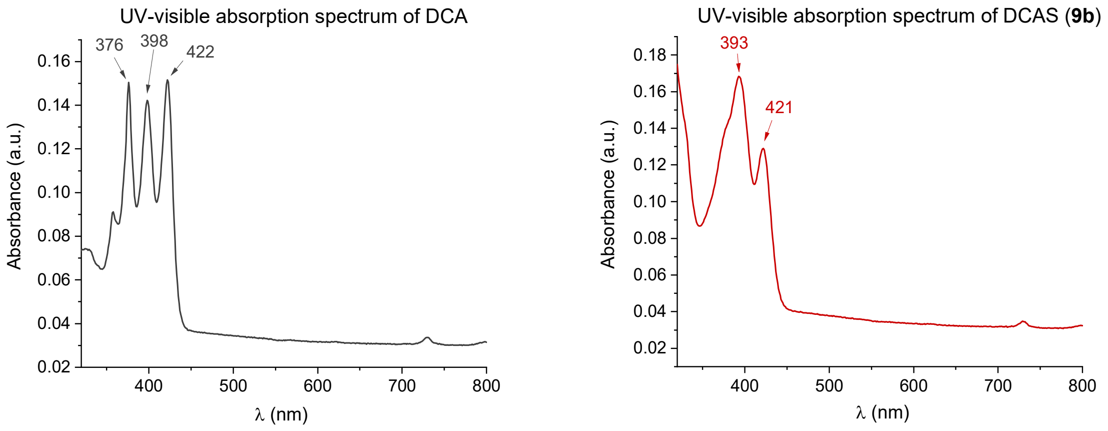

Figure S11. UV-visible absorption spectra of **DCA** and **DCAS** (**9b**).

For solutions of **9a** and **9c** (1 x 10^{-5} M in MeCN), UV-visible absorption spectra were measured using a Merck Pharo 100 Spectroquant spectrophotometer. The spectrum of **9b** measured by this spectrometer is included for direct comparison, the highest absorbance intensity of **9b** correlates with its solubility being the highest of the derivatives (Figure S12).

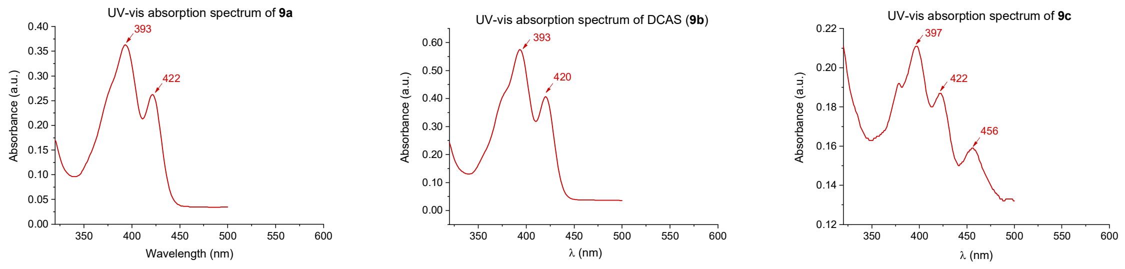

Figure S12. UV-visible absorption spectra of **9a**, **9b** and **9c**.

UV-Vis Spectroscopy of DCAS Radical Anion (DCAS•⁻)

Electrochemical generation

The reduction of **DCAS** (2.5 mM in 0.1 M $^{n}Bu_4N{\cdot}PF_6$) to **DCAS•⁻** was performed according to a literature procedure[23] without any amine substrate. A purple colour was observed in the cathodic half chamber of the divided cell. After 1 h, the contents of the cathodic chamber were taken for UV-vis analysis (Figure S13, black spectra).

Photochemical generation

Online irradiation / UV-vis measurements were performed on an Agilent 8453 spectrometer using an ISMATEC ISM930C dosing pump to continuously pump the catholyte (1 mL/min) through a quartz flow cell (pathlength 1.0 cm). In a 5 mL snap vial, **DCAS** (0.005 mmol, 1.0 equiv.), and DIPEA (0.1 mmol, 20 equiv.) were added. 25 µL of this mixture were added to a quartz cuvette which was made up to 2.0 mL total volume with dry MeCN. A 385 nm LED was used for online irradiation (Figure S13 and S14, left).

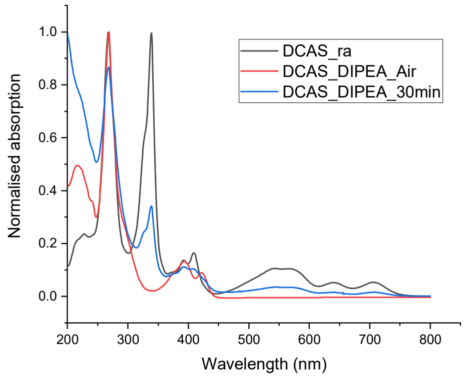

Figure S13. superimposed UV-vis spectra of electrochemically generated, photochemically generated (blue, after 30 mins of irradiation) **DCAS•⁻** and **DCAS** + DIPEA after exposure to air.

Kinetic plots of the online UV-vis experiments showed an increased in the bands at λ_{max} = 350 nm, and λ_{max} = 550 nm (Figure S14, right) over time. A sample spectrum was taken for Figure 6 in the manuscript.

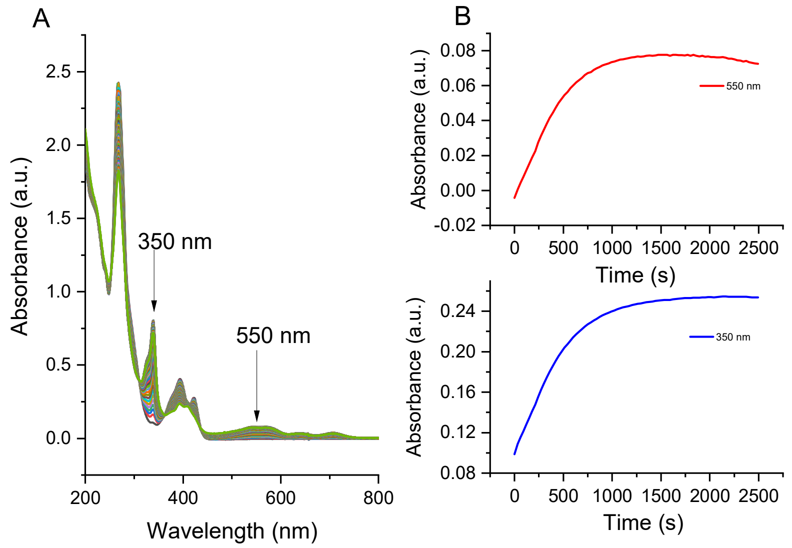

Figure S14. Left: UV-vis absorption spectra during online irradiation from t = 0 min (black) to t = 30 min (green). Right: Kinetic plots showing the increased absorbance of the DCAS radical anion peaks at 350 nm, and 550 nm over 30 min of irradiation.

TD-DFT calculation for the UV-vis of DCAS•⁻

The procedure for time dependent density functional theory (TD-DFT) calculations is outlined in Section "13. Density functional theory computational details and Cartesian coordinates." It predicted peaks at λ_{max} = 547 nm and 350nm for **DCAS•⁻** which are consistent with the new peaks observed from the online UV-vis experiment (Figure S15).

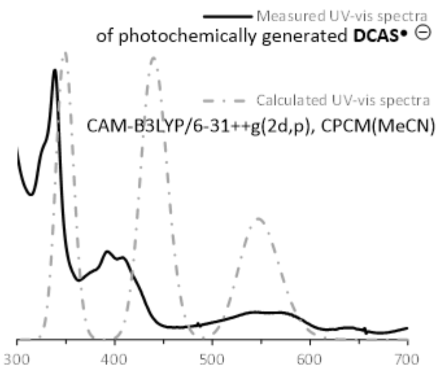

Figure S15. Superimposed and normalized UV-vis spectra of **DCAS•⁻** obtained by the online irradiation / UV-vis measurement and by a TD-DFT calculation using CAM-B3LYP (right) /6-31++G(d,p), CPCM (MeCN) level of theory.

Steady-State Luminescence Spectroscopy

Unless otherwise stated, steady-state luminescence measurements were performed on a Horiba® Scientific FluoroMax-4 instrument, which comprised an USHIO S150MO xenon short arc lamp as an excitation source, 200 - 900 nm double grating excitation and emission monochromators and a R928 Hamamatsu photomultiplier tube. Slit widths for excitation and emission were set at 5 nm. A signal detector (S1) was enabled to measure the intensity signal (counts s^{-1}) and a reference detector (R1) was enabled to compensate for light source fluctuations (μA). Therefore, 'Counts' refers to S1/R1 with the unit s^{-1} / μA but is given arbitrary units (a.u.) for simplicity. FluoroEssence V3.9 software was used for data acquisition and processing. Sample preparation was done with rigorous exclusion of O_2. For **DCA**, data were acquired on a Cary Eclipse Fluorescence spectrometer (courtesy of Prof. Duncan Graham's group, University of Strathclyde), where the excitation monochromator was set to 405 nm and emission was set to measure 410 - 600 nm. Slit widths for excitation and emission were set at 5 nm. Sample preparation was done by Ar bubbling (5 min). Emission spectra for ^{1}**DCAS*** and ^{1}**DCA*** are shown in Figure S16.

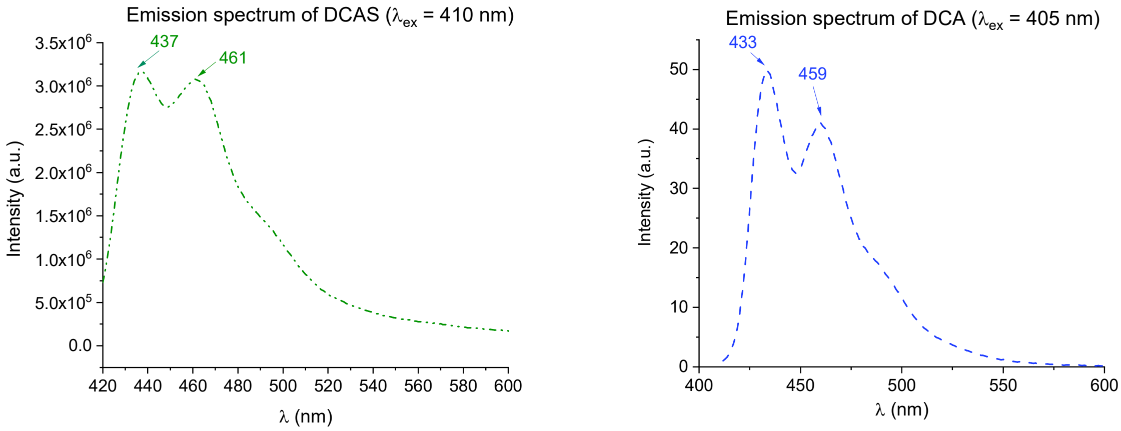

Figure S16. Fluorescence spectra of ¹**DCAS*** and ¹**DCA***.

The Excitation-Emission Matrix was collected for **DCAS** (Figure S17). The emission range was set from 415-600 nm, with a 5 nm increment and 5 nm slits. The excitation range was set from 345-500 nm, with a 5 nm increment and 5 nm slits. An increase in emission intensity resulted from excitation at 395 nm *vs* 420 nm, corroborating a higher excited state population of ¹**DCAS*** which accords with the more intense UV-vis absorption band at λ_{max} = 393 nm compared to λ_{max} = 421 nm and accords with the higher preparative yield using 395 nm LEDs.

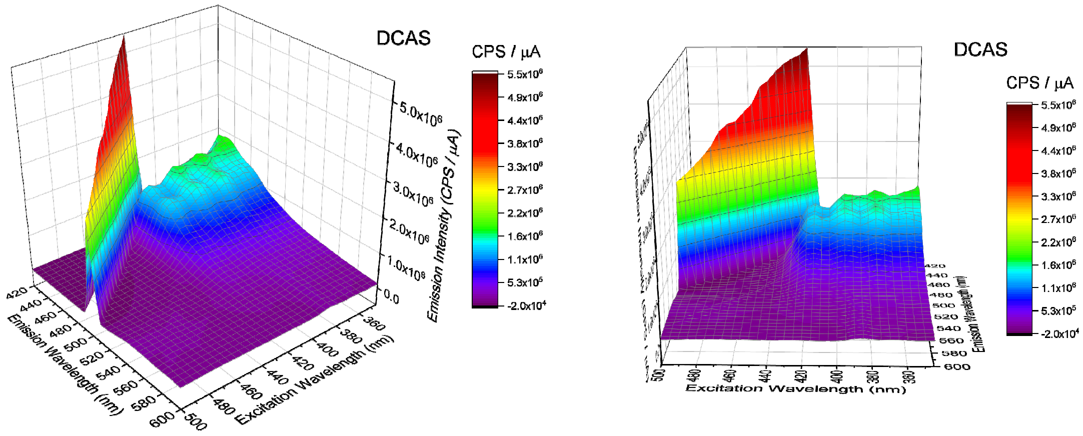

Figure S17. Excitation-Emission Matrix for **DCAS**.

Fluorescence quenching experiments and Stern-Volmer analysis

To prepare samples at different [O_2], samples prepared under N_2 were bubbled with a balloon of compressed air or pure O_2, for 5 min and immediately measured. The concentration of dissolved O_2 in O_2-saturated MeCN at rt is known at [8.1 mM]; leading to a [1.7 mM] concentration of dissolved O_2 in air-saturated MeCN, assuming a 21% O_2 content in air and assuming similar solubilization rates of O_2 and N_2 in MeCN. A bubbling time of 5 min was 'optimal' since the lifetime was found to stabilize after 3-5 min, while extended bubbling (15 min) led to notable solvent evaporation and decreasing temperature.

As validation of this method, the rate constant obtained for quenching of $*[Ru(bpy)_3]^{2+}$ with O_2 (k_q = 2.84 x 10^9 M^{-1} s^{-1}) was determined (Figure S18) and found to be in excellent agreement with the literature value (k_q = 2.7 x 10^9 M^{-1} s^{-1}).[24]

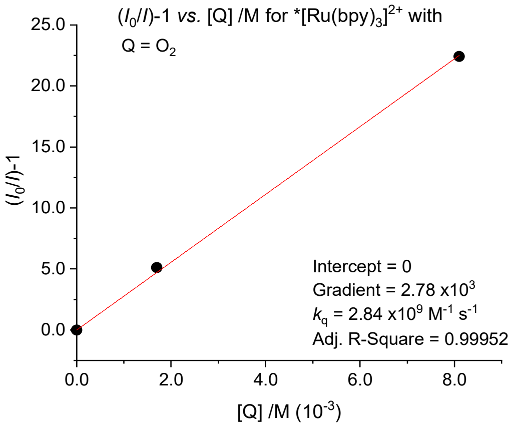

Figure S18. Stern-Volmer quenching of $^3[Ru(bpy)_3{}^{2+}]^*$ by O_2 as method validation.

For DCA and DCAS, the integration time was set to 0.1 s and the slid widths were 5 nm for both excitation and emission. For DCAS, data were acquired using the aforementioned spectrometer, where the excitation monochromator was set to 405 nm and emission was set to measure 420 - 550 nm (Figure S19).

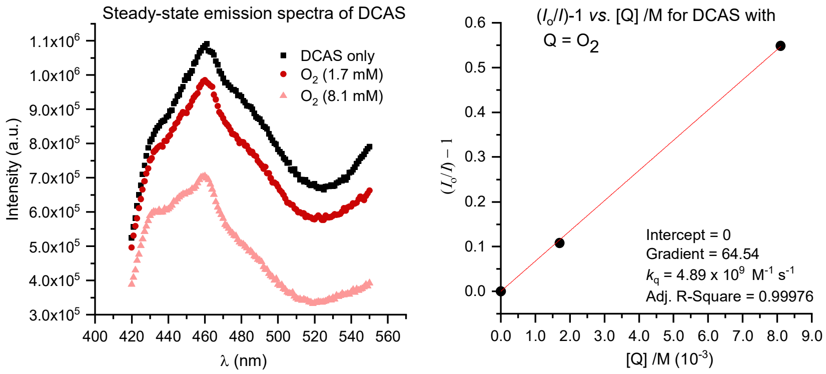

Figure S19. Stern-Volmer quenching of ^{1}**DCAS*** by O_2.

For Stern-Volmer quenching experiments using Tropine (Figure S20) and DABCO (Figure S21) as quenchers, sample preparation was done under an N_2 atmosphere MBRAUN UNIlab glovebox (courtesy of Prof. Dr. Robert Wolf's group).

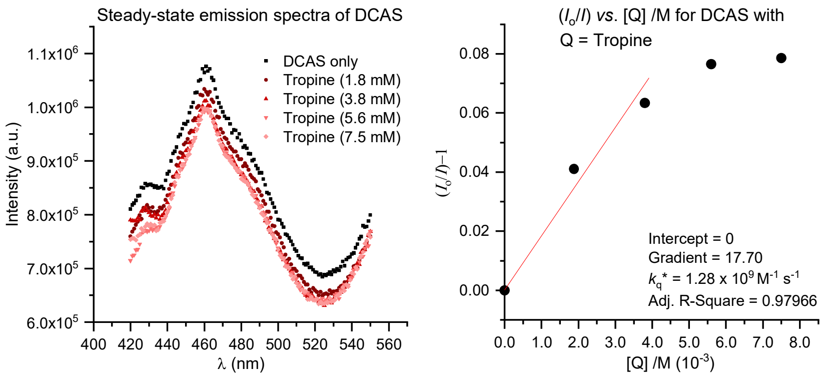

Figure S20. Stern-Volmer quenching of [1]**DCAS*** by tropine **1b**.

The quenching of [1]**DCAS*** by tropine displayed non-linear behaviour (Figure S21), whose origin is currently unclear. The rate constant was determined from a best fit line of the intercept and first two datapoints, representing the 'maximum possible' effective rate constant (k_q*) in order to make comparisons with other quenchers.

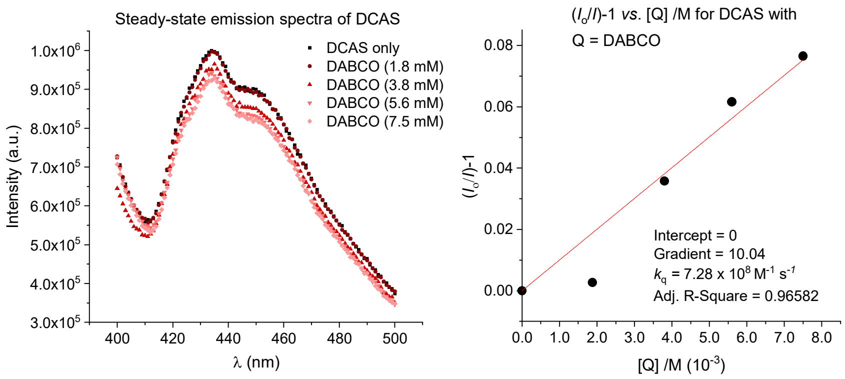

Figure S21. Stern-Volmer quenching of [1]**DCAS*** by DABCO.

In the quenching of [1]**DCAS*** with dextromethorphan **1a** (Figure S22), the peak wavelength for data collection was set at 460 nm and monitored over time. The excitation monochromator was set to 405 nm and emission was set to measure 460 nm. Slit widths were maintained at 5 nm for both excitation and emission. The interval time between data collection was 1 s. As can be seen, the peak intensity stabilized (5-6 minutes after degassing with Ar bubbling). The rate of peak intensity decrease over time before this point was identical for each sample no matter the quencher concentration, confirming the reliability of comparisons made *via* this method when samples are compared after the same degassing time.

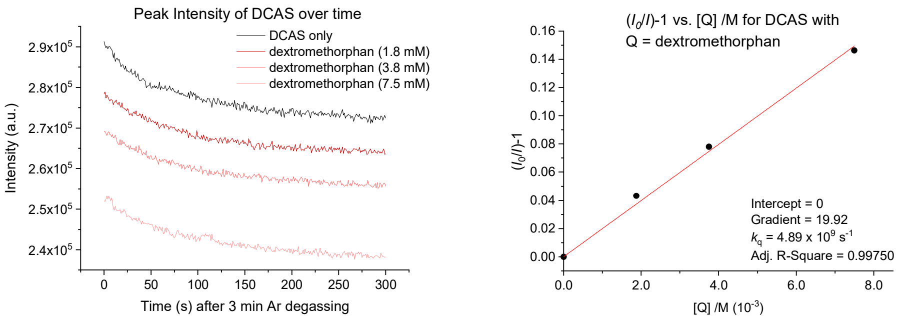

Figure S22. Stern-Volmer quenching of [1]**DCAS*** by dextromethorphan **1a**.

For **DCA**'s quenching with dextromethorphan **1a** (Figure S23) and *N*-methyl-1,2,3,4-THIQ (Figure S24) (synthesized according to literature proceedures[25]), data were acquired on a Cary Eclipse Fluorescence spectrometer (courtesy of Prof. Duncan Graham's group, University of Strathclyde), where the excitation monochromator was set to 405 nm and emission was set to measure 410 - 600 nm.

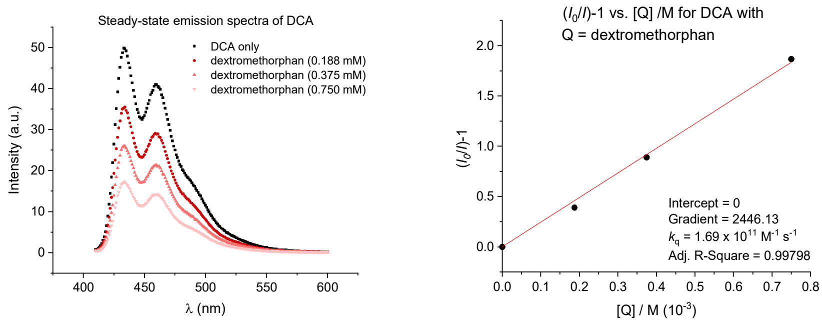

Figure S23. Stern-Volmer quenching of [1]**DCA*** by dextromethorphan **1a**.

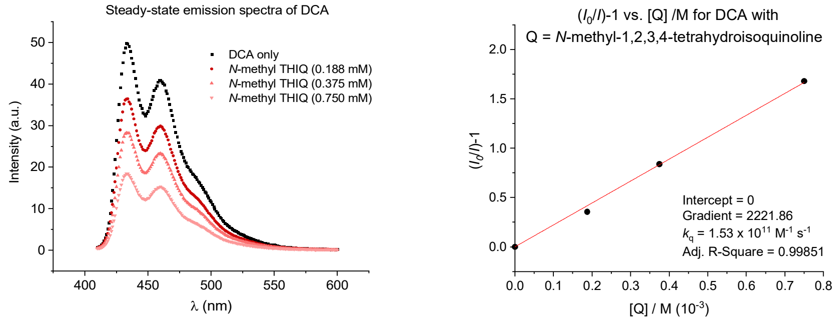

Figure S24. Stern-Volmer quenching of [1]**DCA*** by *N*-methyl THIQ.

Time-resolved Luminescence Measurements

Time-resolved luminescence measurements and lifetime measurements were performed using the time-correlated single photon counting technique (TCSPC) on a HORIBA Jobin Yvon FluoroCube. For **DCA** and **DCAS**, excitation was performed using a Horiba Scientific DeltaDiode laser at 375 nm (pulsed excitation with a typical pulse width of 80 ps and maximum repetition rate of 100 MHz), monitoring

fluorescence emission at 460 nm and collecting data in the 200 ns (0.052 ns/channel) time domain for [1]**DCA*** or 100 ns (0.026 ns/channel) time domain for [1]**DCAS***. Low absorption was maintained at the excitation wavelength (*A* = 0.1) and lifetimes were measured at the magic angle (54.7°). Sample degassing was done using Ar bubbling for 5 min.

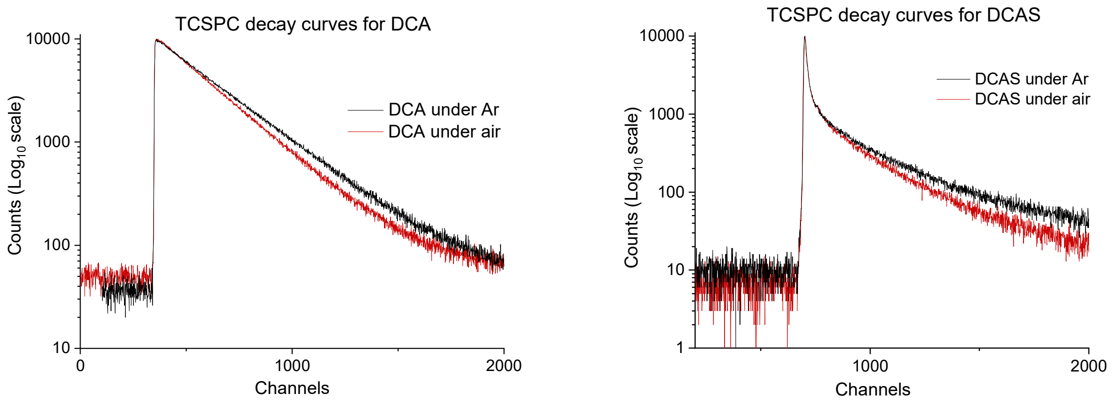

Figure S25. TCSPC decay curves for [1]**DCA*** and [1]**DCAS*** in presence and absence of air.

[1]**DCA*** was found to exhibit monoexponential decay behaviour, consistent with that reported in the literature for [1]**DCA*** in MeCN and other solvents (Figure S25).[26] [1]**DCAS*** however exhibited multiexponential decay behaviour (the instrument response factor can be excluded). A triexponential fit was most suitable to fit the whole range following excitation pulse; while the latter part of the decay (ignoring the initial decay of <1 ns) could be fitted more precisely with a biexponential decay. The lifetime with the greatest contribution to the overall decay of [1]**DCAS*** (τ_2) was taken for comparisons of [1]**DCA*** and [1]**DCAS***. As can be seen in Table S5, both τ_1 and τ_2 decreased in the presence of air regardless of fitting parameters.

Since the decay profiles of [1]**DCAS*** under air and Ar both show multiexponential decay, the multiexponential decay is unrelated to presence of O_2. While the origin of this effect is not yet clear, one possibility is that the sulfonamide chains lead to a certain degree of static quenching in [1]**DCAS*** that occurs prior to any bimolecular quenching with O_2. The timescales for vibrational, rotational relaxations and internal conversion are too short to detect on a nanosecond time domain. Since the initial sub-nanosecond decay is close to the instrument response factor (IRF), it is also possible that processes occur on a faster timescale that cannot be resolved at this time domain. However, when fitting is started at a channel number after the IRF, decay fitting is still biexponential.

Overall, this points to **DCAS** exhibiting a different kind of aggregation in solution compared to the known π-stacking aggregation of **DCA**.

Table S5. Lifetimes of [1]**DCA*** and [1]**DCAS*** in the presence and absence of air.

Entry	Excited state	Sample Preparation	Time domain (ns/channel)	τ (ns)		Literature τ (ns)
1	[1]**DCA***	Ar bubbling	0.052	14.5 (χ^2 = 1.16)		14.9[26]
2	[1]**DCA***	Equilibrated in air	0.052	12.7 (χ^2 = 1.05)		12.6[26]
3	[1]**DCA***	O_2 bubbling	-	-		7.6[26]
3	[1]**DCAS***	Ar bubbling	0.052	Triexp. fit:<br>τ_1 2.9 (38%)<br>τ_2 10.1 (41%)<br>τ_3 0.3 (21%)<br>χ^2 = 1.30	-	-
4	[1]**DCAS***	Ar bubbling	0.026	Triexp. fit:<br>τ_1 4.1 (34%)<br>**τ_2 13.7** (38%)<br>τ_3 0.3 (27%)<br>χ^2 = 1.20	Biexp. fit:<br>τ_1 4.3 (37%)<br>**τ_2 13.4** (63%)<br>χ^2 = 1.06	-
5	[1]**DCAS***	Equilibrated in air	0.026	τ_1 2.9 (30%)<br>**τ_2 9.1** (42%)<br>τ_3 0.2 (28%)<br>χ^2 = 1.15	τ_1 2.9 (33%)<br>**τ_2 9.2** (67%)<br>χ^2 = 1.11	-

9. Calculation of Dicyanoanthracene Derivatives' Physical Properties and Other Mechanistic Investigations

Solubilities of Dicyanoanthracenes

The solubilities of dicyanoanthracene derivatives were gauged using the calculated logP (octanol/water partition coefficient, a measure of lipophilicity) and total polar molecular surface area (TPSA). A low logP and high TPSA indicates a solubility in polar solvents. In Table S6, **DCAS** (**9b**) has the lowest logP and highest TPSA; while **DCA** has the opposite indicating an enhanced solubility upon introduction of the sulfonamide substituents at the dicyanoanthracene core. The actual solubilities for **DCAS** (**9b**) and **DCA** then measured in triplicates by dissolving 20 μmol of dicyanoanthracenes in 5 mL Acetonitrile; then adding 0.1 mL in increments. At par with the calculated parameters, **DCAS** has a higher solubility in acetonitrile (3.1 μM ± 0.2 μM) than **DCA** (1.5 μM ± 0.02 μM)

Table S6. Summary of calculated Physical properties of dicyanoanthracene derivatives using Molinspiration.

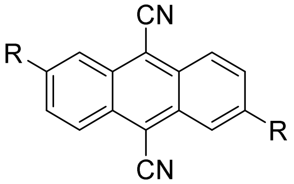

Compound	R group	smiles	logP[a] (solubility)[c]	TPSA[b]
**DCA**	H	N#Cc2c1ccccc1c(C#N)c3cccc c23,3.67,47.58,18,228.25,2,0, 0,0,205.75	3.67 (1.9 ± 0.1)[c]	47.58
**9a**	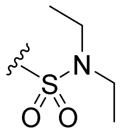	CCN(CC)S(=O)(=O)c3ccc2c(C #N)c1cc(S(=O)(=O)N(CC)CC) ccc1c(C#N)c2c3,3.75,122.34,3 4,498.63,8,0,0,8,427.63	3.75	122.34
**9b**	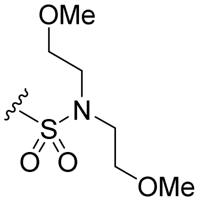	CCN(CC)S(=O)(=O)c3ccc2c(C #N)c1cc(S(=O)(=O)N(CC)CC) ccc1c(C#N)c2c3,3.75,122.34,3 4,498.63,8,0,0,8,427.63	2.19 (0.34 ± 6×10^{-3})[c]	159.28
**9c**	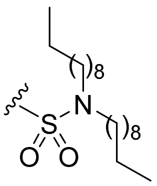	CCN(CC)S(=O)(=O)c3ccc2c(C #N)c1cc(S(=O)(=O)N(CC)CC) ccc1c(C#N)c2c3,3.75,122.34,3 4,498.63,8,0,0,8,427.63	10.57	122.34

[a]calculated octanol-water partition coefficient, [b]total polar molecular surface area, [c] measured experimentally, in mg mL^{-1}.

Singlet Oxygen Trapping Experiment Using α-Terpine

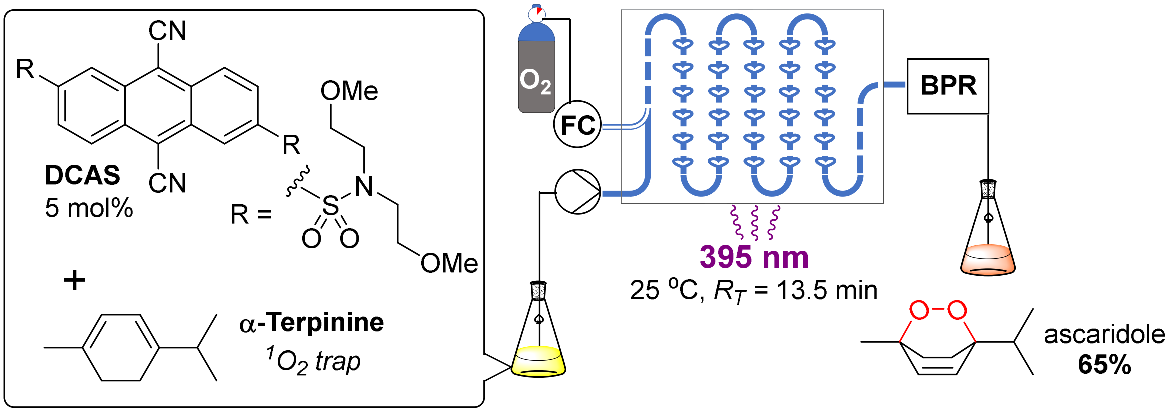

Following **General Procedure B** but α-terpinine (10.2 mM, 0.255 mmol) was used as a substrate under room temperature (25 °C) conditions. After 1 pass, 2.5 mL of the sample was collected and carefully concentrated *in vacuo*. The ^{1}H NMR spectrum of the crude reaction mixture was immediately obtained to prevent decomposition (^{1}H NMR yield = 65%):

^{1}H NMR (300 MHz, $CDCl_3$) δ 6.50 (d, *J* = 8.5 Hz, 1H), 6.41 (d, *J* = 8.5 Hz, 1H), 2.06 – 1.99 (m, 2H), 1.99 – 1.85 (m, 1H), 1.52 (d, *J* = 9.6 Hz, 2H), 1.00 (d, *J* = 6.9 Hz, 6H);

The spectrum was consistent with the literature for ascaridole.[27]

Effect of DABCO as a $^1O_2$ Physical Quencher on the Yield and Relative Formation Rate of 2b

DABCO is known in the literature as a singlet oxygen quencher.[28] Four sets of experiments were performed and for each, **General Procedure B** was followed using Tropine (12 mM, 0.30 mmol) as amine substrate but with an increasing loading of DABCO additive (Table S7). The relative rate of formylation was calculated (see formula below)[28] and plotted against [DABCO]. A linear fit with R^2 = 0.977 was obtained, indicating that singlet oxygen was a crucial reagent of the reaction (Figure S26).[18,28]

To confirm that the decrease in formation rate of **2b** was due to physical quenching of $^1O_2$ by DABCO and not by reductive quenching of ^{1}DCAS* (E^p_{ox} = +2.31 V *vs* SCE) by DABCO (E^p_{ox} = +0.68 V *vs* SCE)[29] given the very favorable difference in redox potentials, steady-state fluorescence experiments were carried out (*vide supra*) comparing **1b**, DABCO and O_2 as quenchers at different concentrations. Comparison of the Stern-Volmer plots revealed that kinetic preference for quenching of ^{1}DCAS* followed the order: O_2 >> 1b > DABCO.

Table S7. Yield of and inverse relative rate for the formation **2b** under increasing [DABCO].

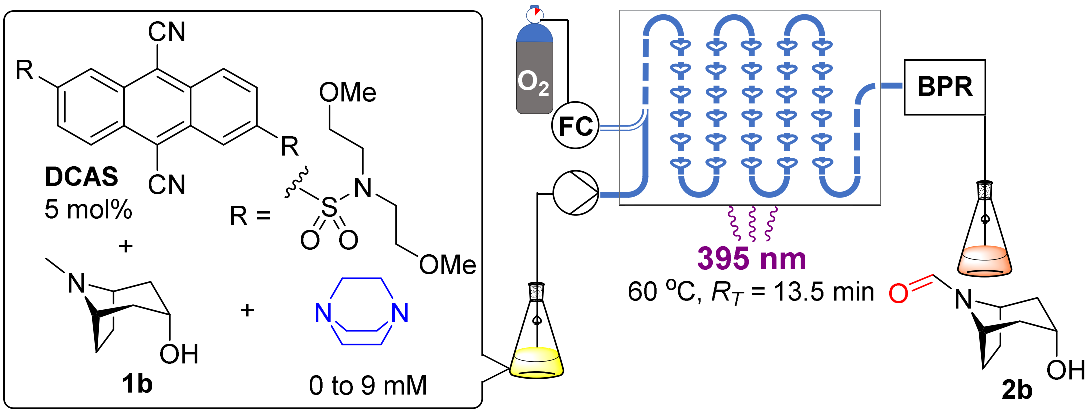

[DABCO] (mM)	^{1}H NMR Yield of **2b** (%)	1/relative rate[a]
0	49	1
3	42	1.17
6	32	1.53
9	29	1.70

[a][product]^o/[product] = (% ^{1}H NMR Yield)^o/ (% ^{1}H NMR Yield) = relative rate of formylation

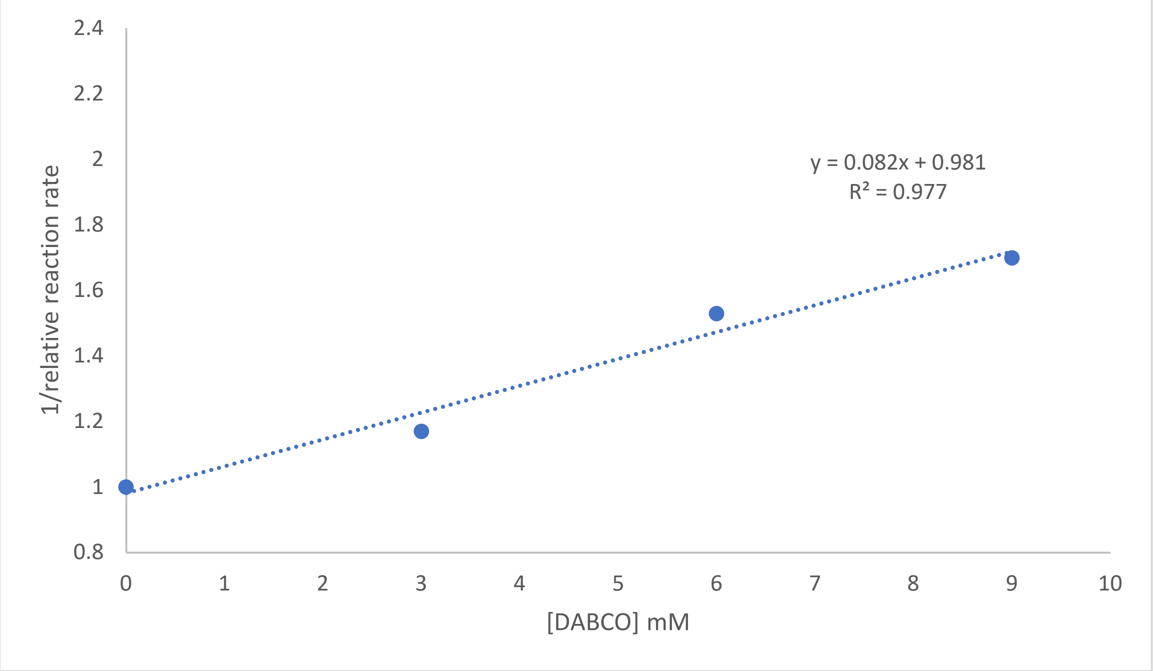

Figure S26. Linear regression plot of results in Table S7.

Isotope (O_2^{18}) Labeling Experiment.

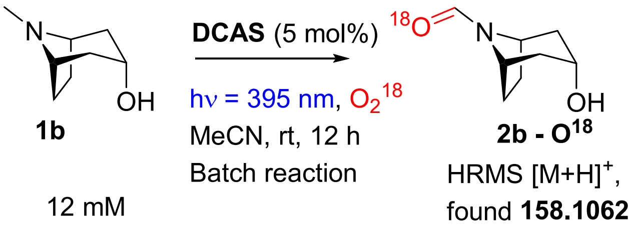

The O_2^{18} enriched gas was generated by electrolysis of O^{18} – labeled water (H_2O^{18}) using Solar Hydrogen™ electrolysis kit by Horizon Educational, Horizon Fuel Cell Europe s.r.o. (Figure S27,A). In a 5 mL high pressure compatible reaction vial equipped with a magnetic stir bar, 2.5 mL reaction solution as added prepared similar to **General Proceedure B** using Tropine (12 mM, 0.03 mmol), crimped sealed with septum, and freeze pump thaw cycle was performed three times. While still on vacuum, 20 mL of O_2^{18} enriched gas was injected to the reaction solution. Then another 10 to 15 mL of gas was added with force till the syringe resisted further compression. The reaction solution was stirred under irradiation (395 nm, LED light) for 12 hours at rt. Then, the crude product mixture was filtered using a syringe filter and submitted for HRMS analysis. The mass spectrum of $[M+H]^+$ is shown below (Figure S27,B).

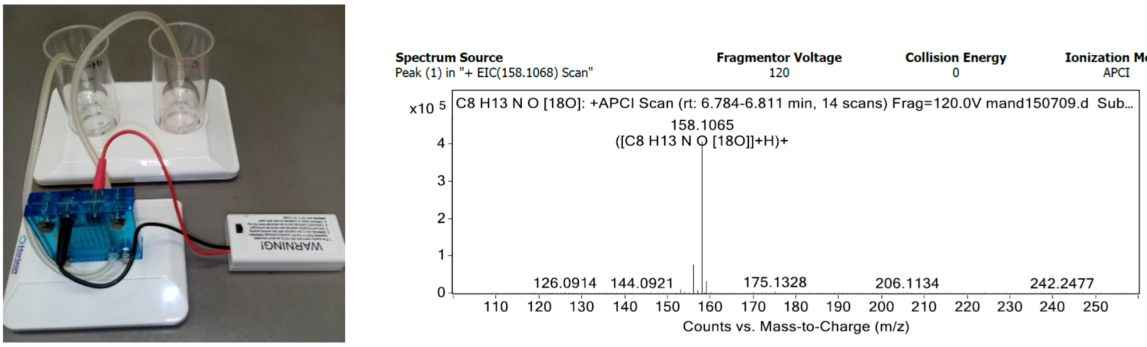

Figure S27. A) Electrochemical cell used to generate O_2^{18} from H_2O^{18}. **B**) HRMS Spectrum of **2b**-O^{18}.

Complete Relative Transmission Spectra of Catalyst Solutions.

The data in Figure S28 were recorded using the Vapourtec UV-150's fitted fiber-optic spectrometer, which detects the intensity and wavelength of light transmitted through the fluoropolymer reactor coil. Measurements therefore reflect the absorption of 420 nm light by the reactor coil contents.

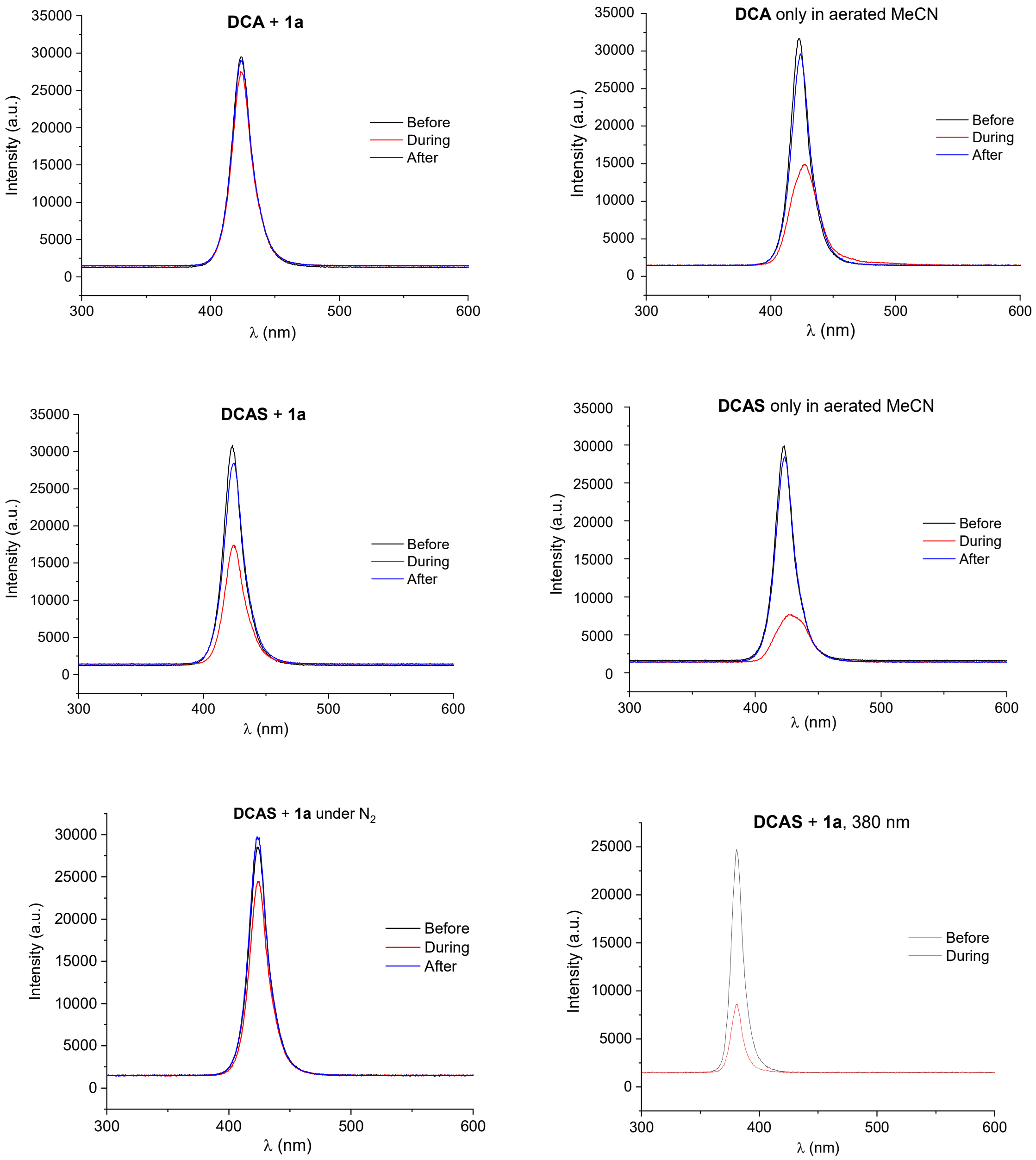

Figure S28. Relative transmission of LED light through the tubular reactor coil of different reaction mixtures.

10. Copies of NMR Spectra

Catalyst Syntheses:

^{1}H NMR of 8a

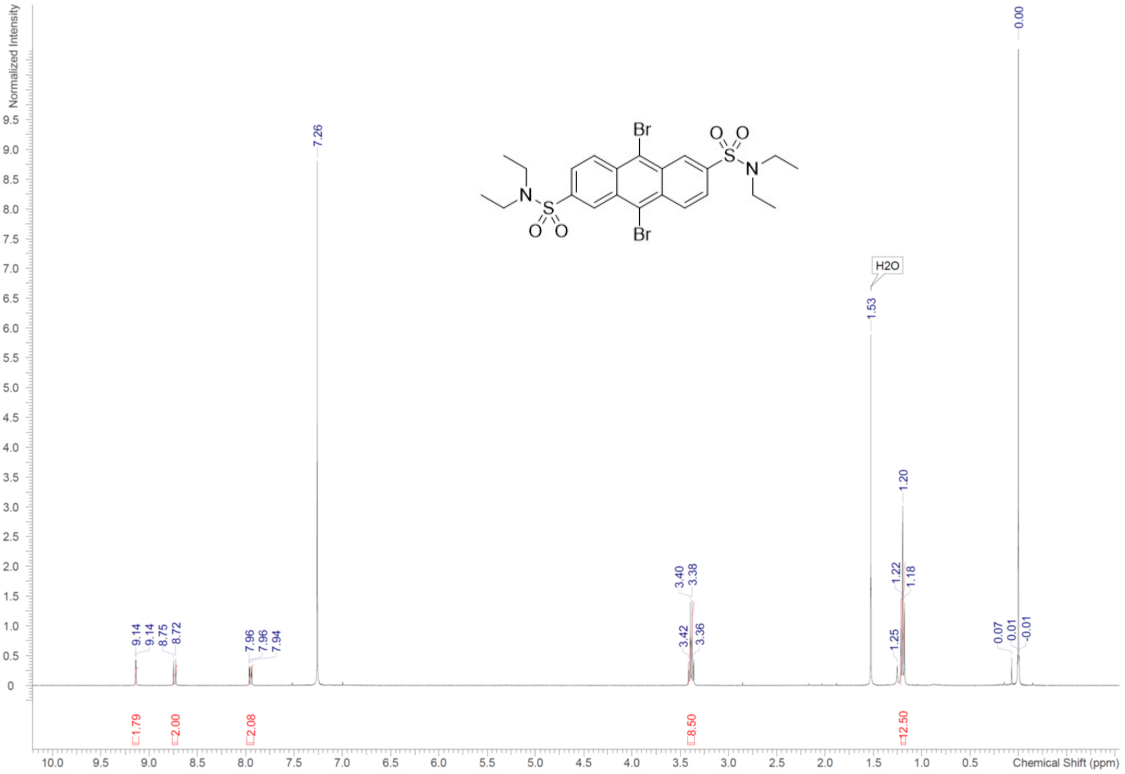

^{13}C NMR of 8a

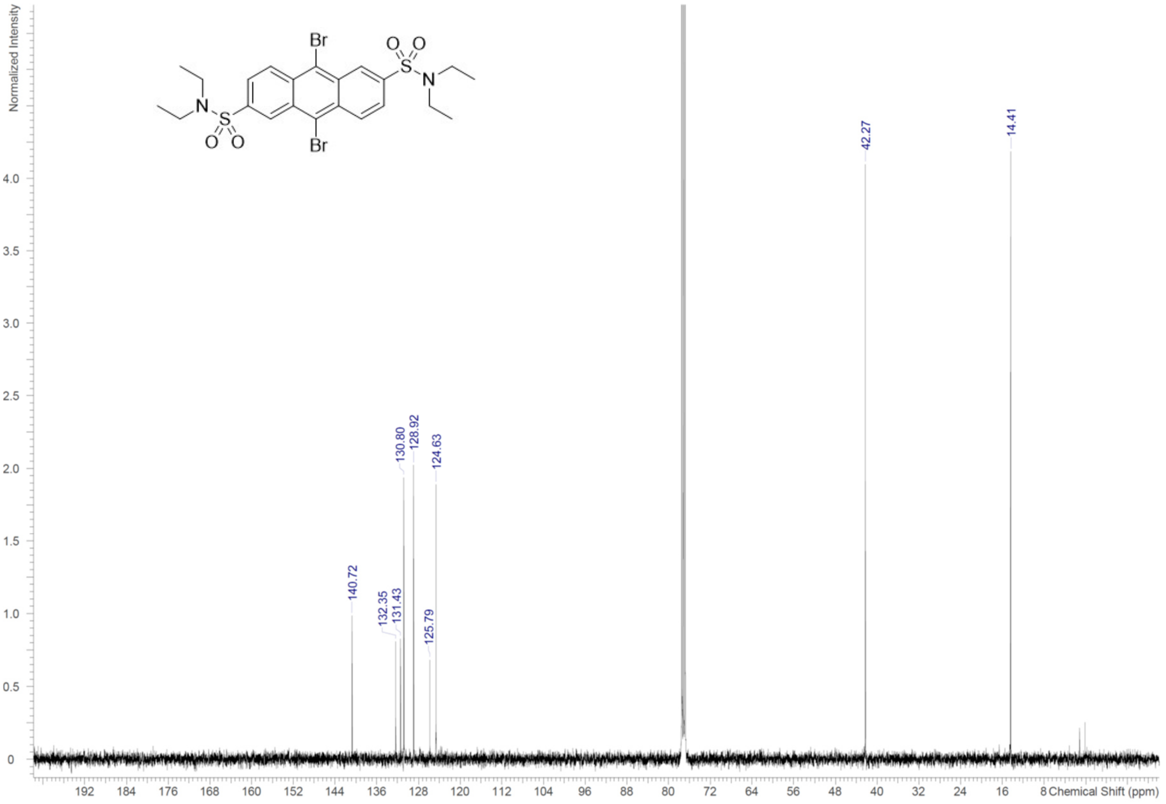

^{1}H NMR of 8b

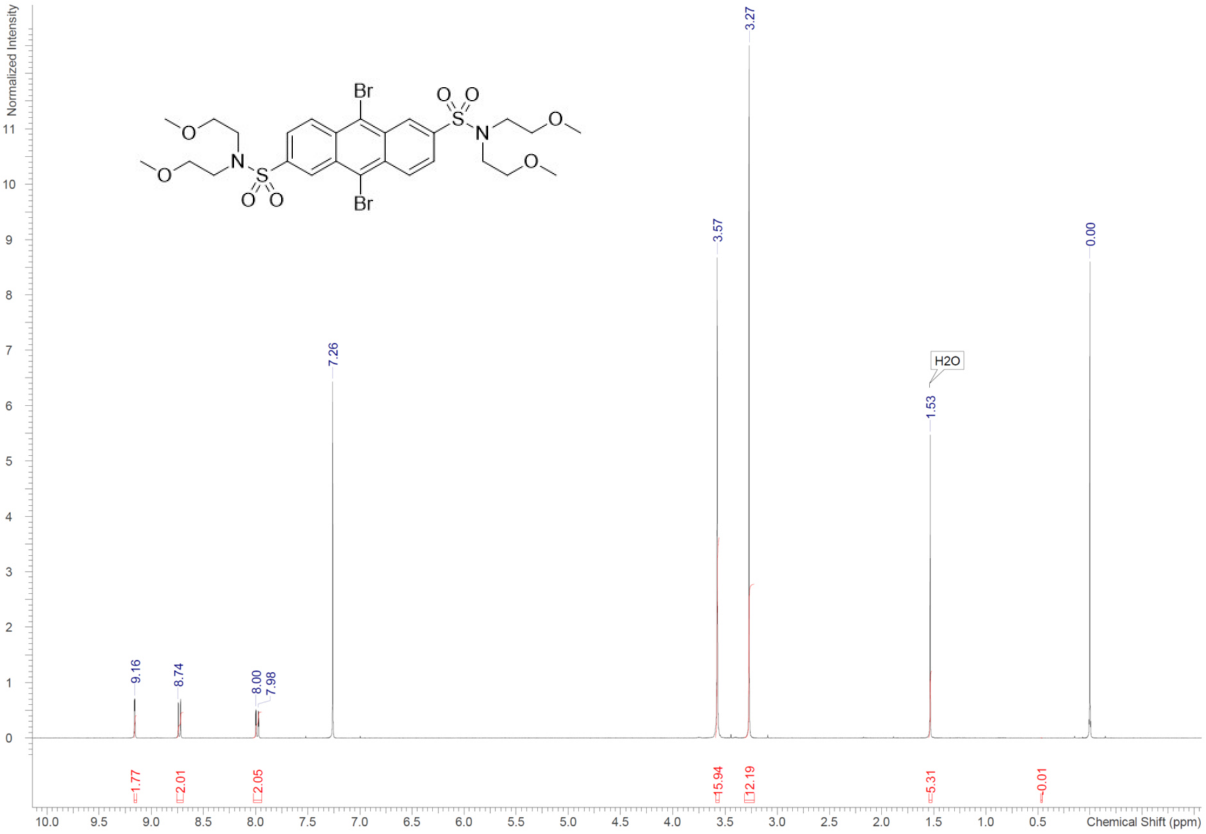

^{13}C NMR of 8b

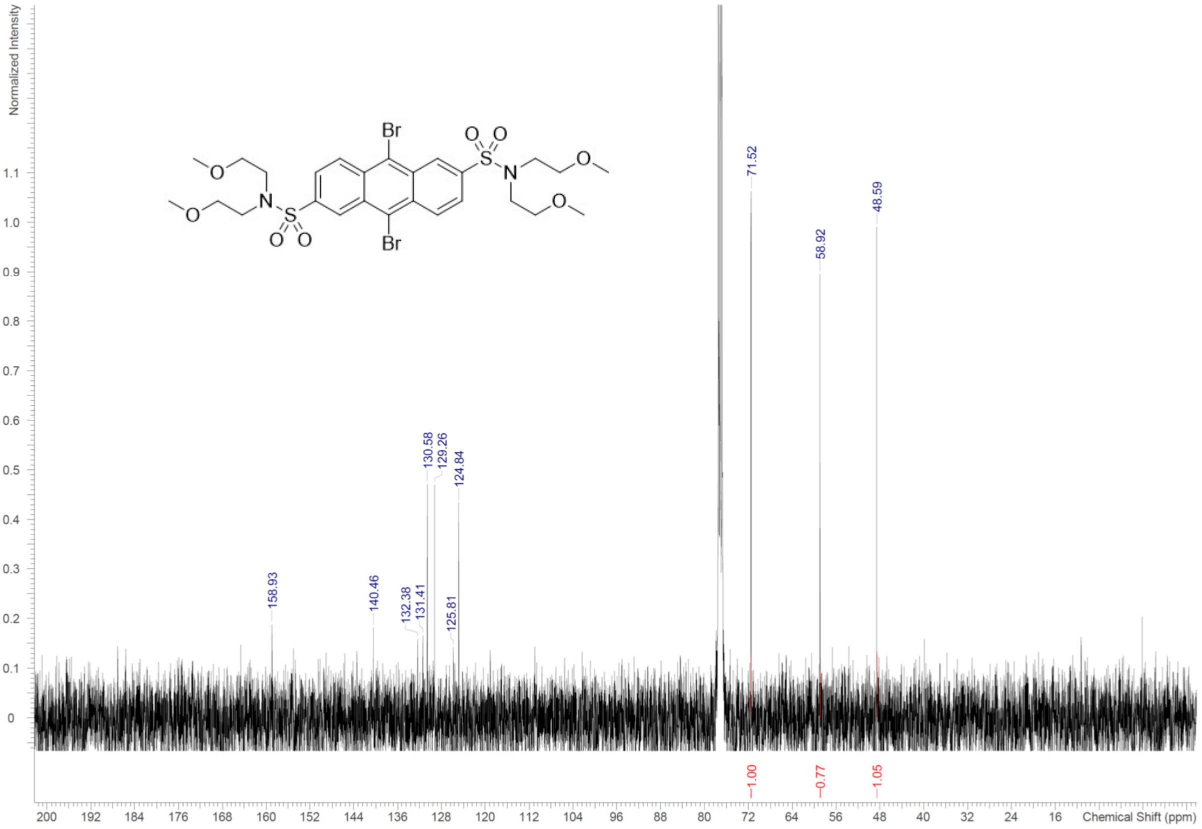

^{1}H NMR of 8c

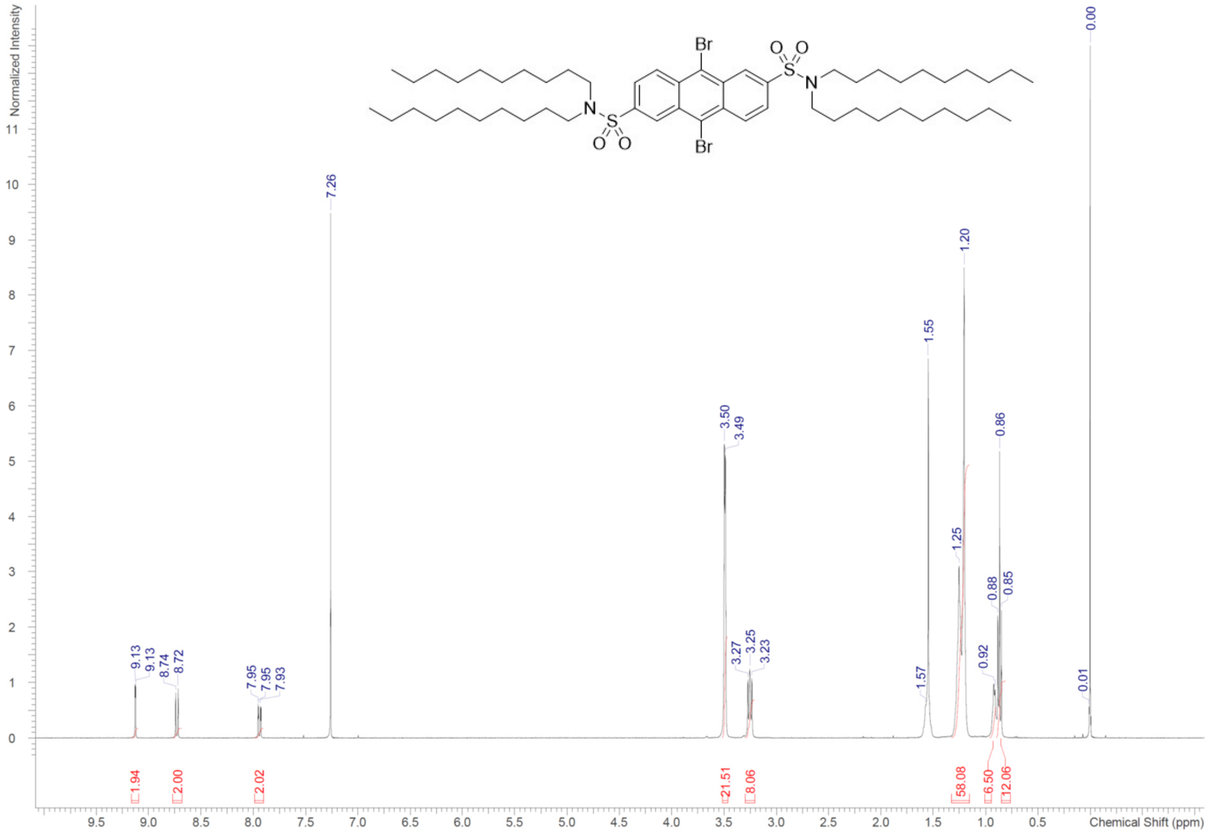

^{13}C NMR of 8c

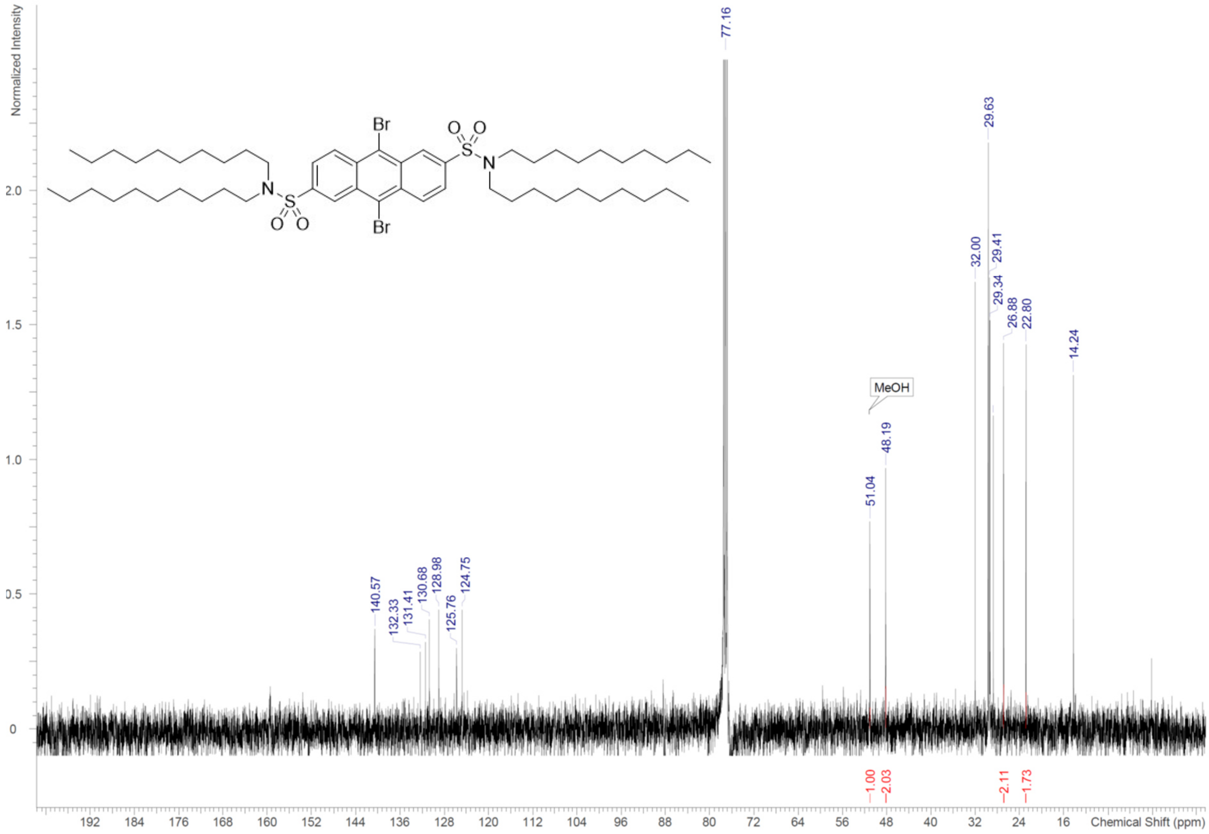

^{1}H NMR of 9a

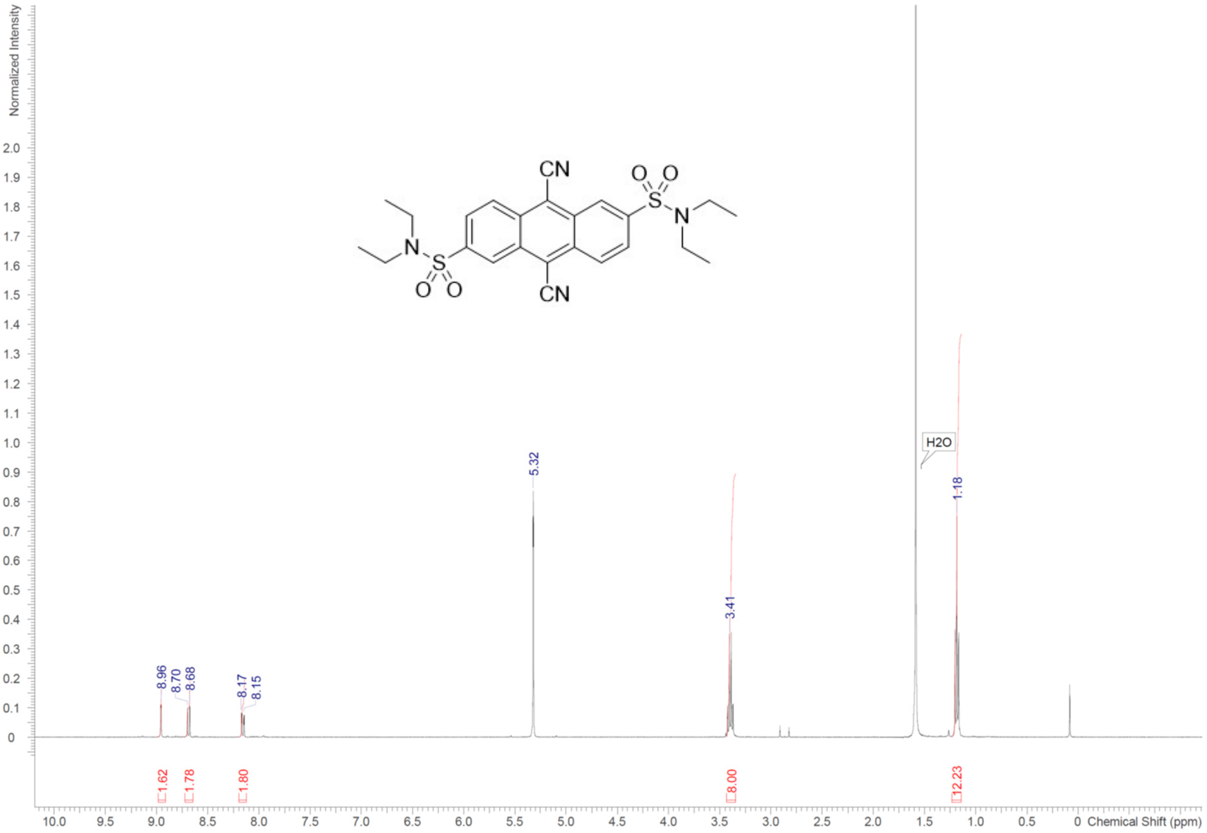

^{13}C NMR of 9a

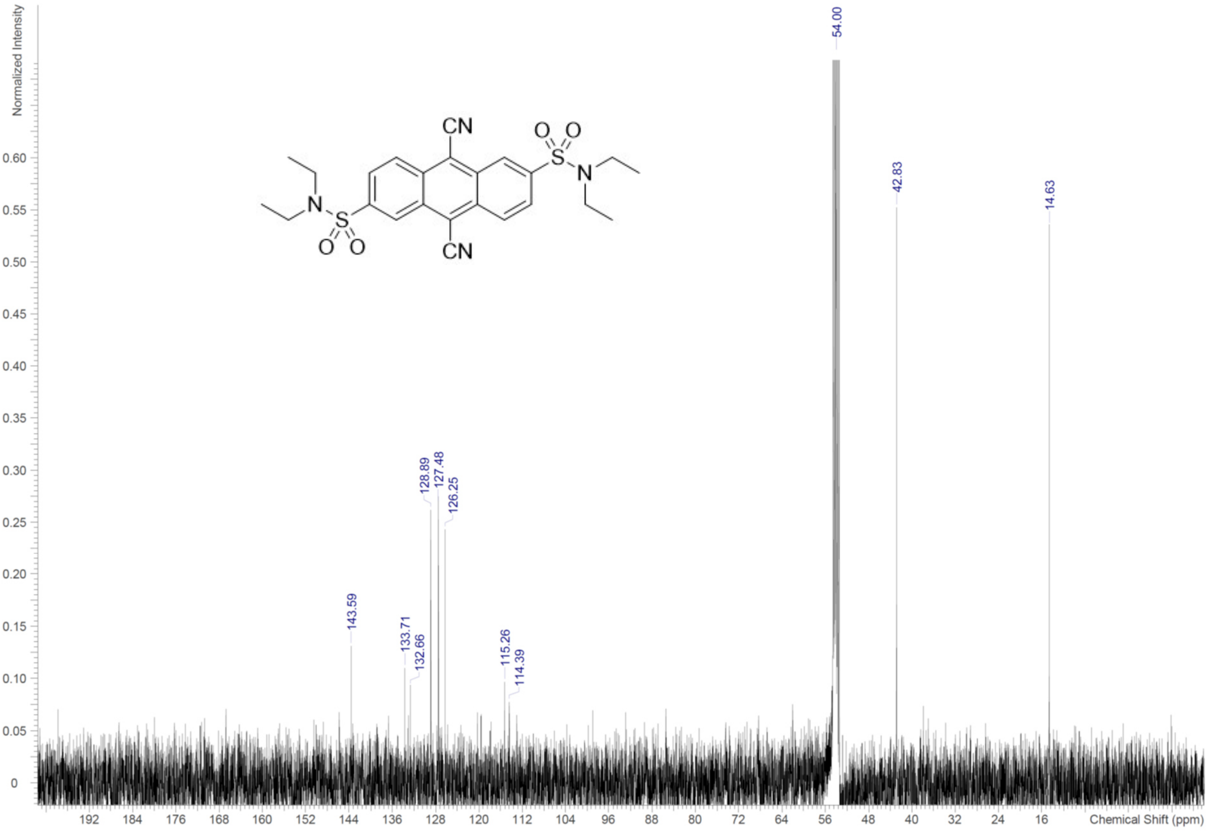

^{1}H NMR of 9b

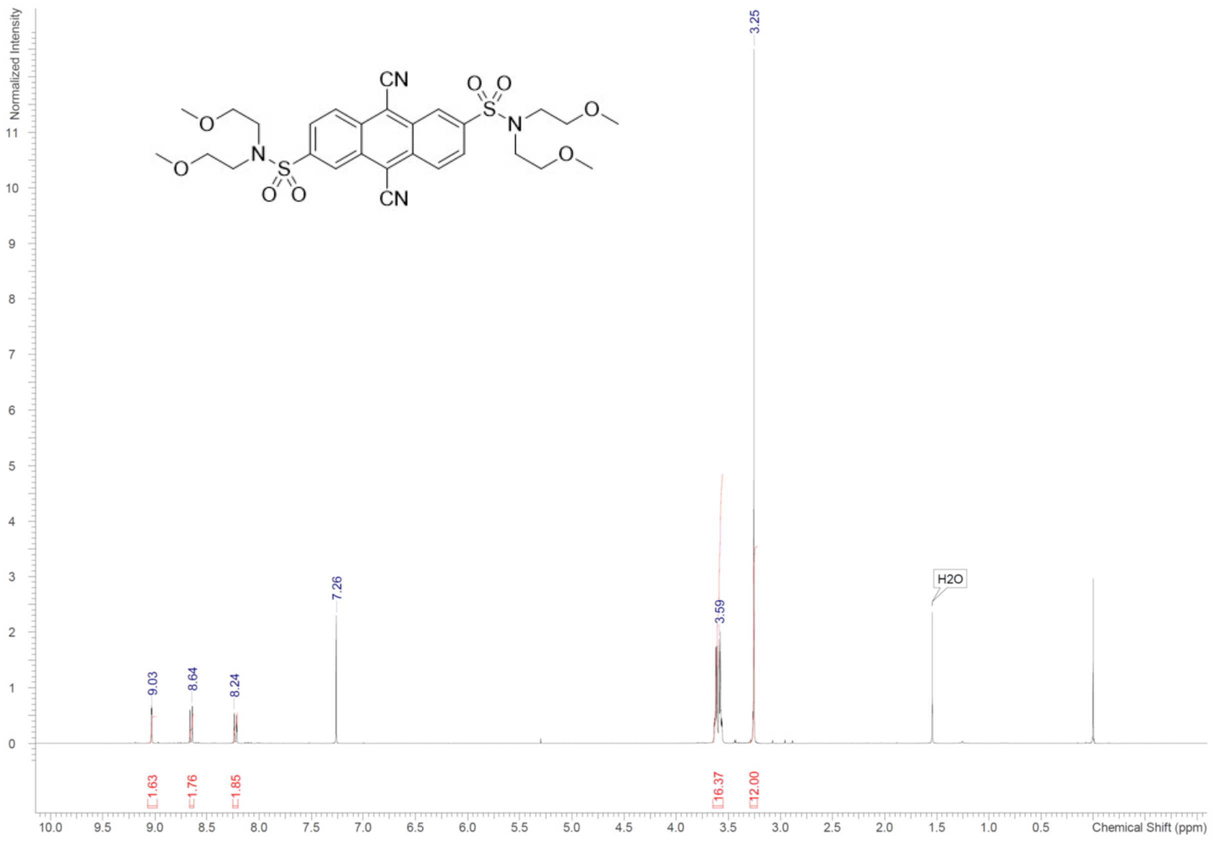

^{13}C NMR of 9b

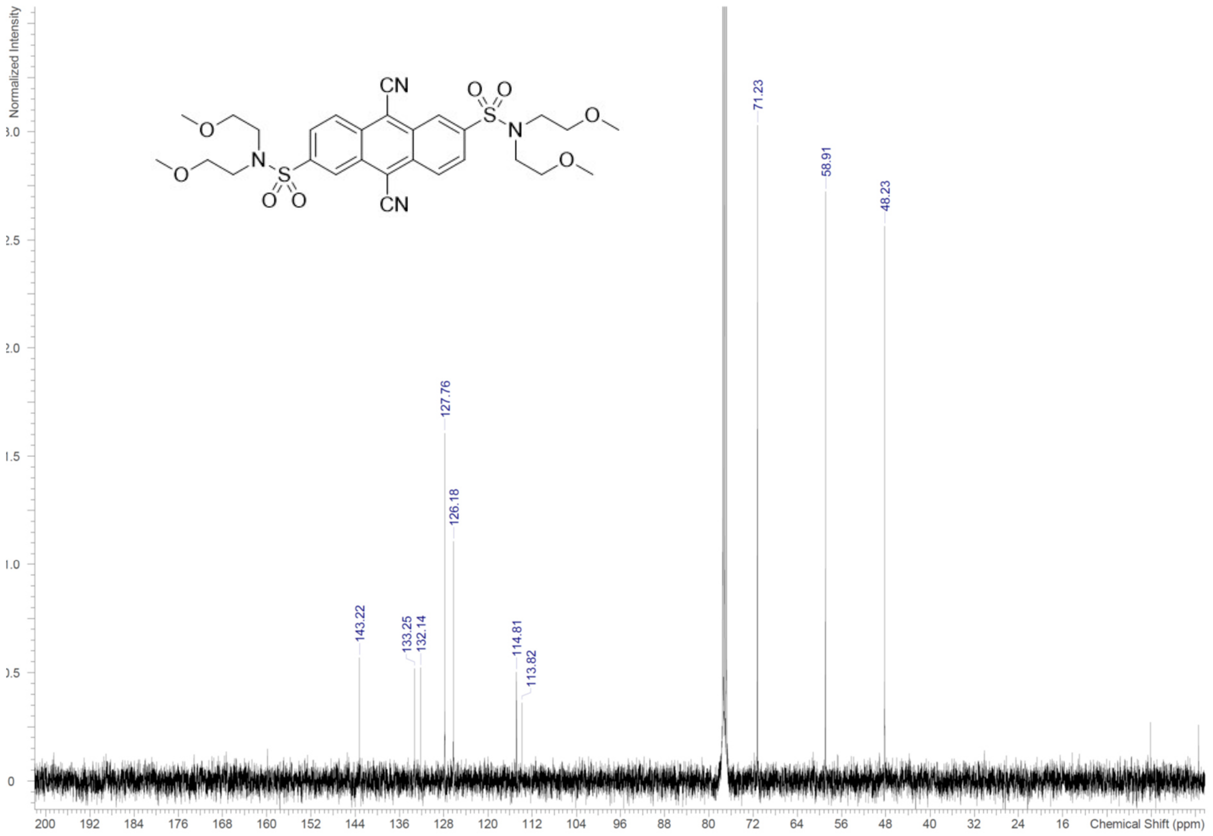

^{1}H NMR of 9c

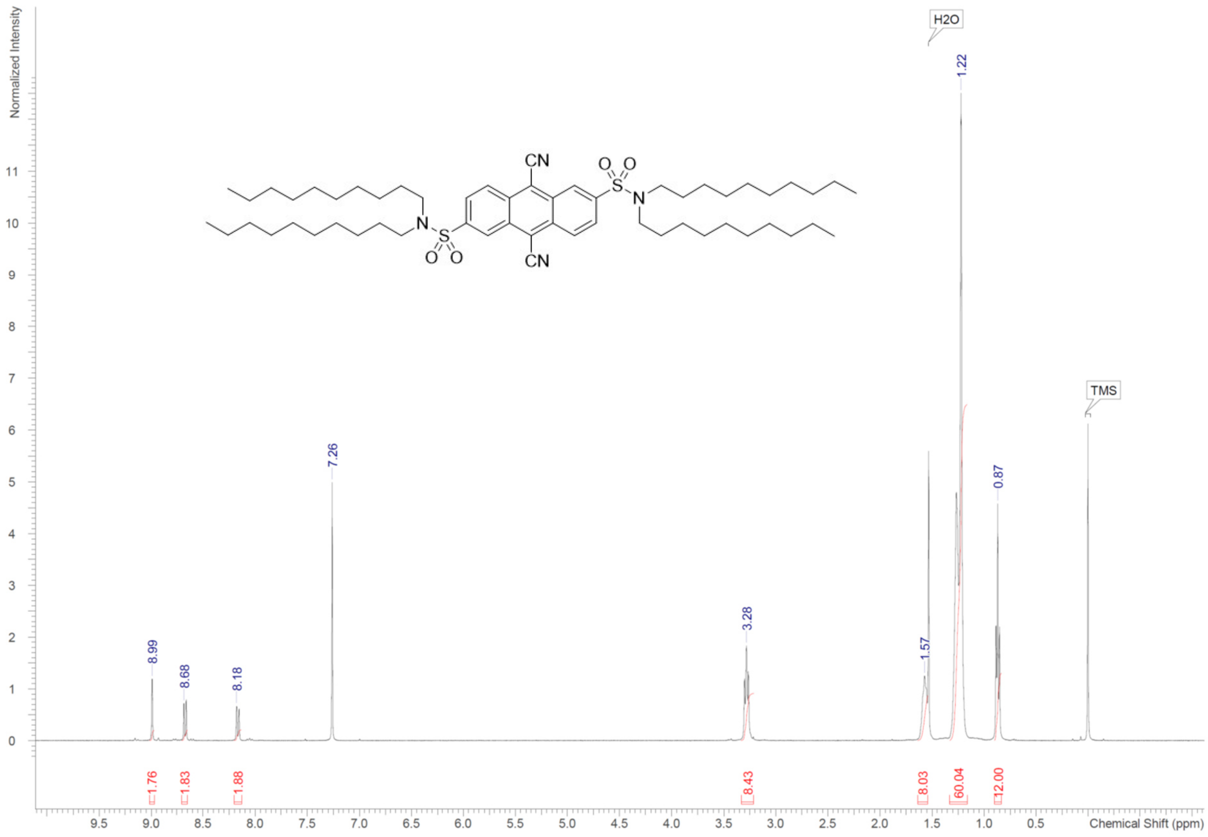

^{13}C NMR of 9c

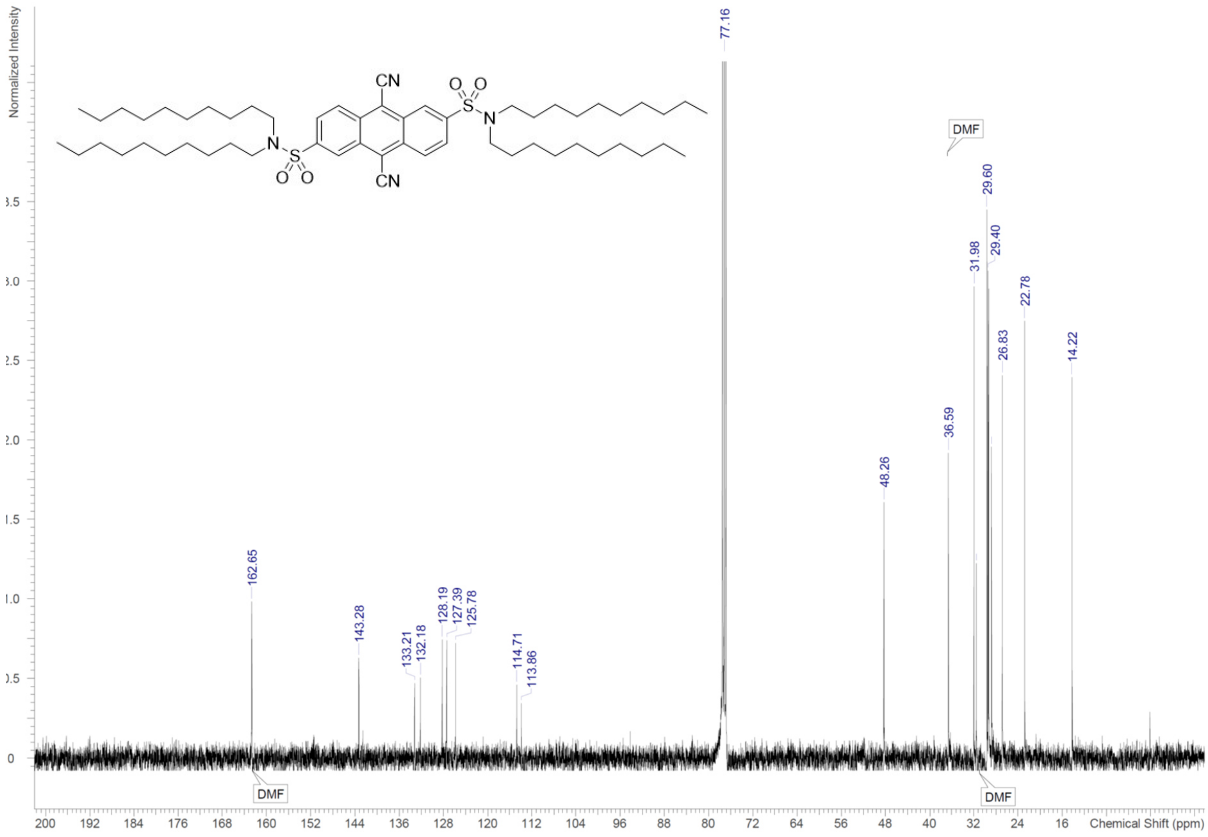

Starting Material Trialkylamines:

^{1}H NMR of (4bS,9S)-3-methoxy-11-methyl-6,7,8,8a,9,10-hexahydro-5H-9,4b-(epiminoethano)phenanthrene (Dextromethorphan free base, 1a)

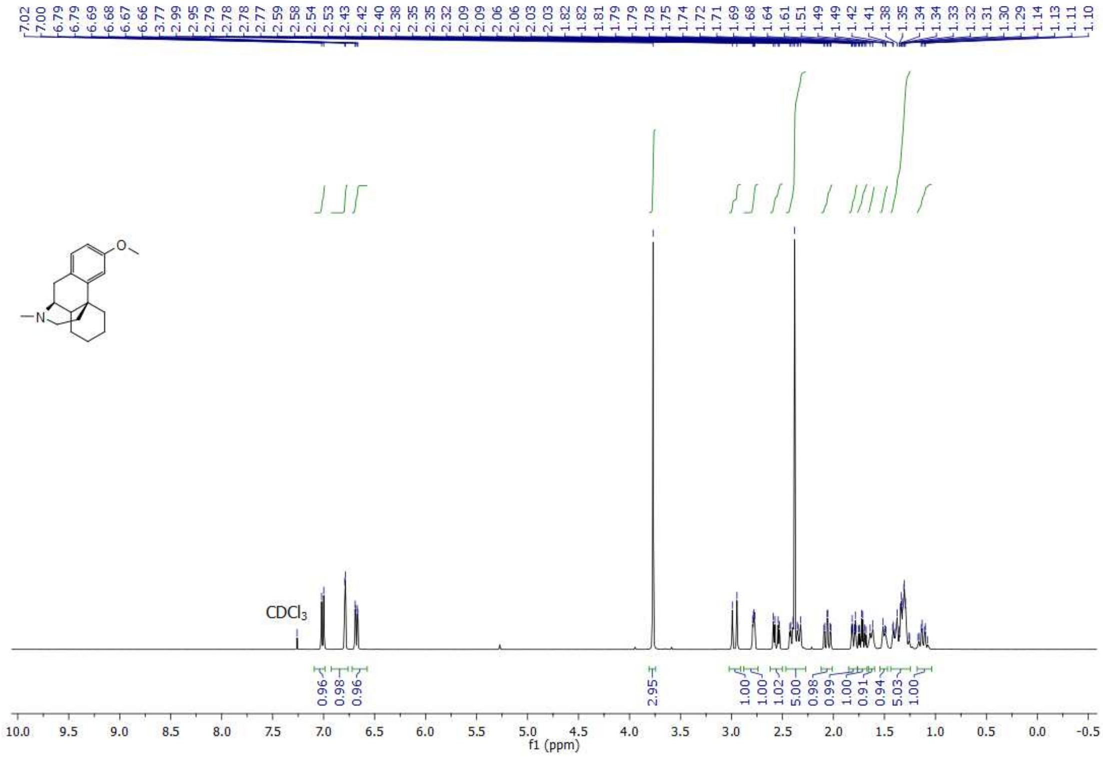

^{13}C NMR of 1a(4bS,9S)-3-methoxy-11-methyl-6,7,8,8a,9,10-hexahydro-5H-9,4b-(epiminoethano)phenanthrene (Dextromethorphan free base, 1a)

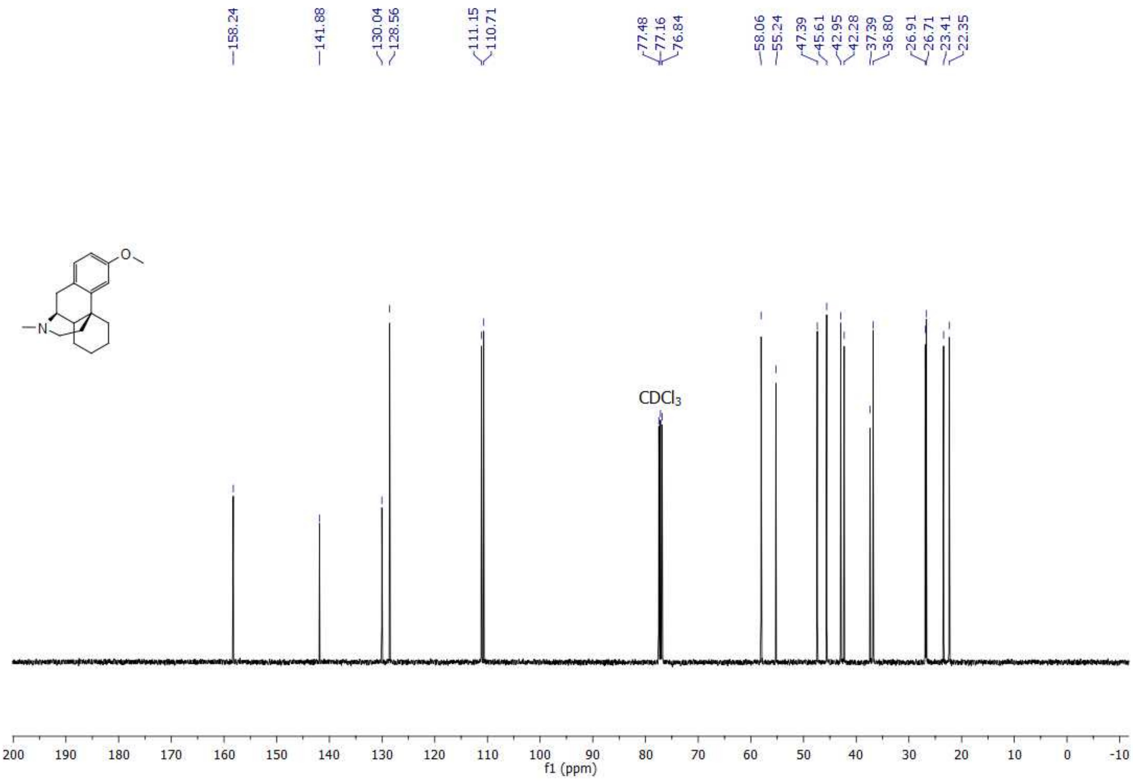

^{1}H NMR of (1R,3r,5S)-8-methyl-8-azabicyclo[3.2.1]octan-3-yl (S)-3-hydroxy-2-phenylpropanoate (Atropine free base, 1d)

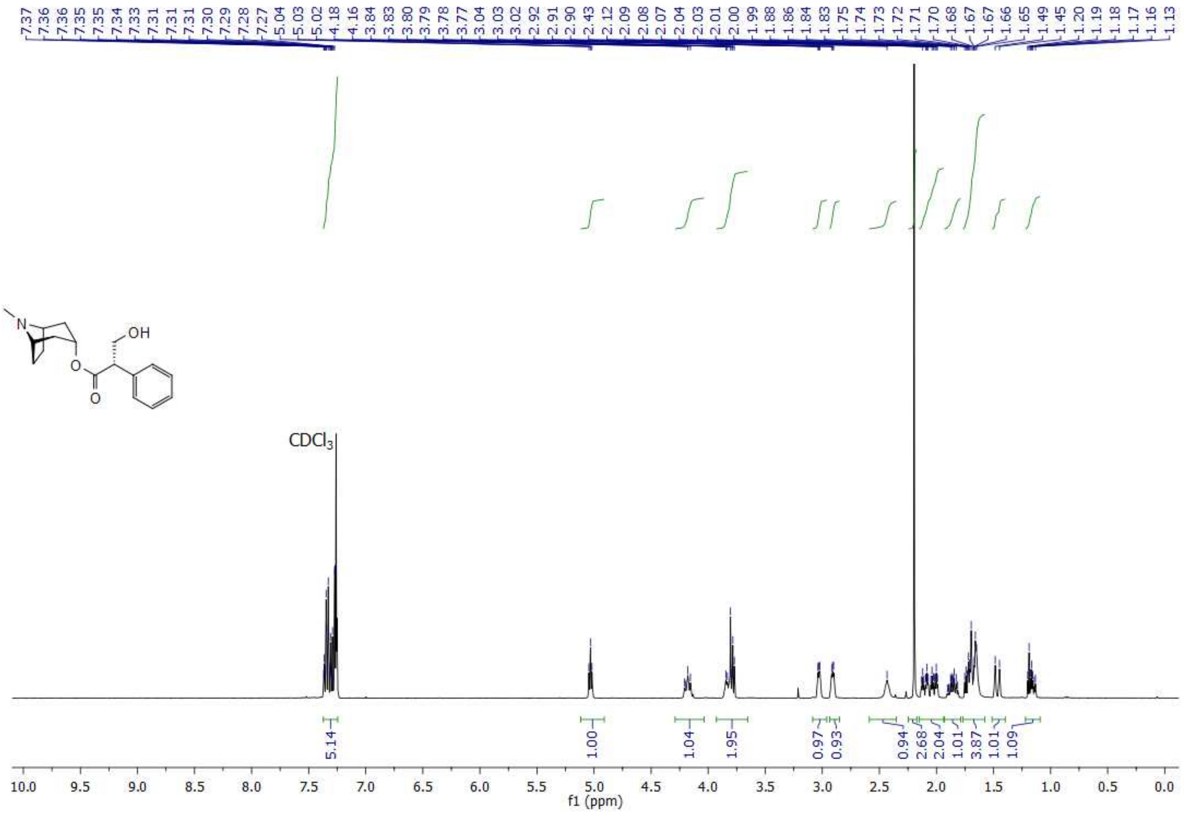

^{13}C NMR of (1R,3r,5S)-8-methyl-8-azabicyclo[3.2.1]octan-3-yl (S)-3-hydroxy-2-phenylpropanoate (Atropine free base, 1d)

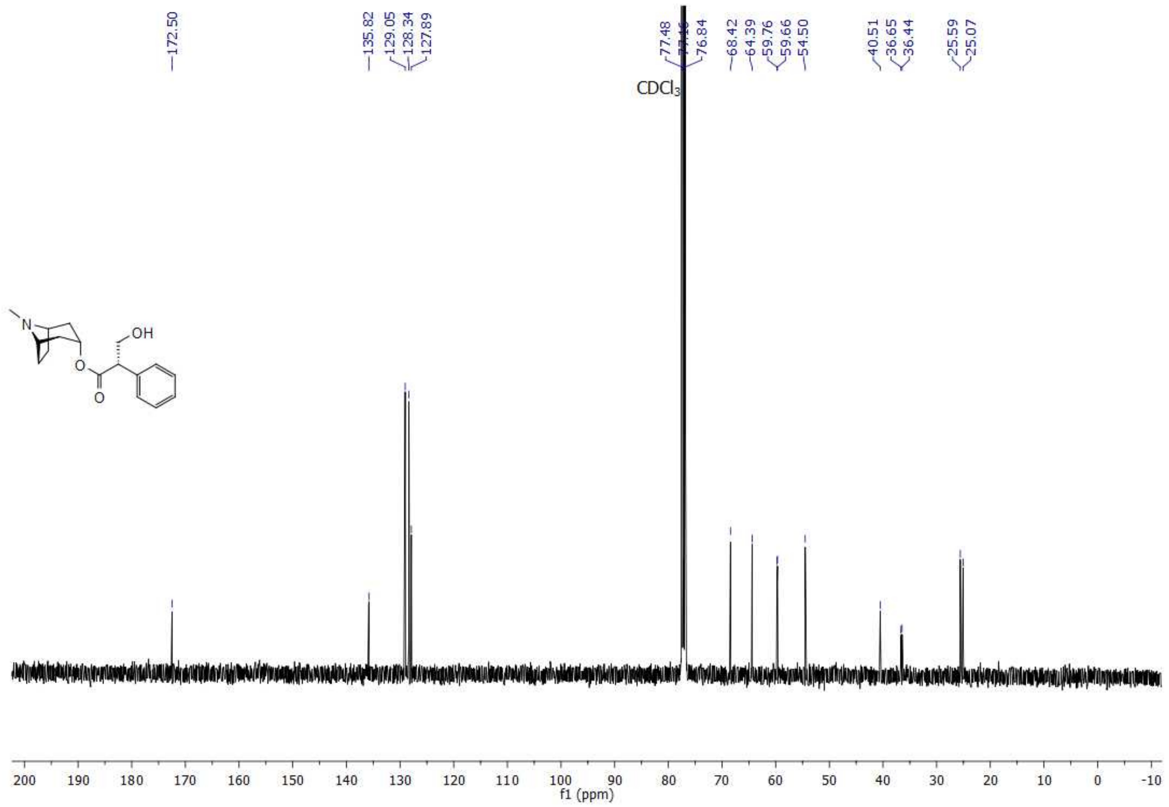

^{1}H NMR of (1R,2R,4S,5S,7s)-9-methyl-3-oxa-9-azatricyclo[3.3.1.02,4]nonan-7-yl (S)-3-hydroxy-2-phenylpropanoate (Scopolamine free base, 1e)

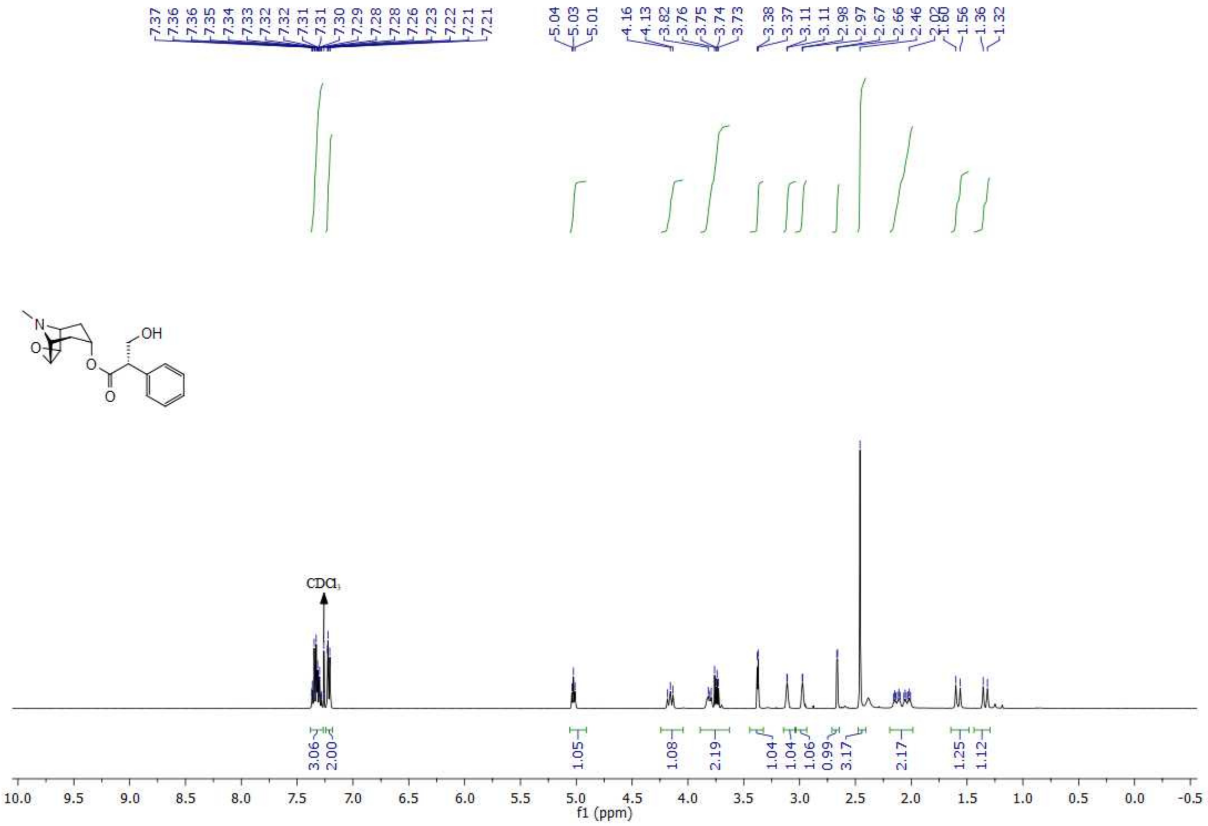

^{13}C NMR of (1R,2R,4S,5S,7s)-9-methyl-3-oxa-9-azatricyclo[3.3.1.02,4]nonan-7-yl (S)-3-hydroxy-2-phenylpropanoate (Scopolamine free base, 1e)

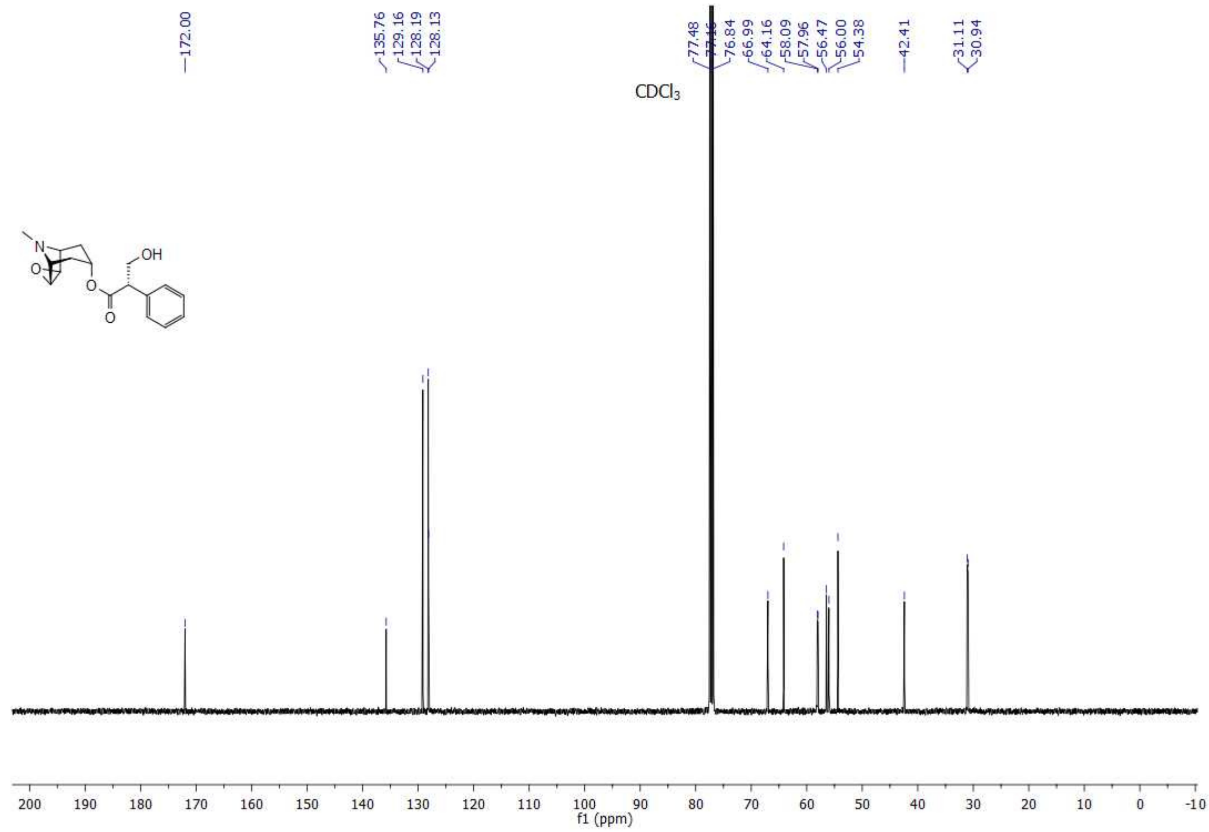

^{1}H NMR of (1R,3r,5S)-8-methyl-3-((methyldiphenylsilyl)oxy)-8-azabicyclo[3.2.1]octane (1f)

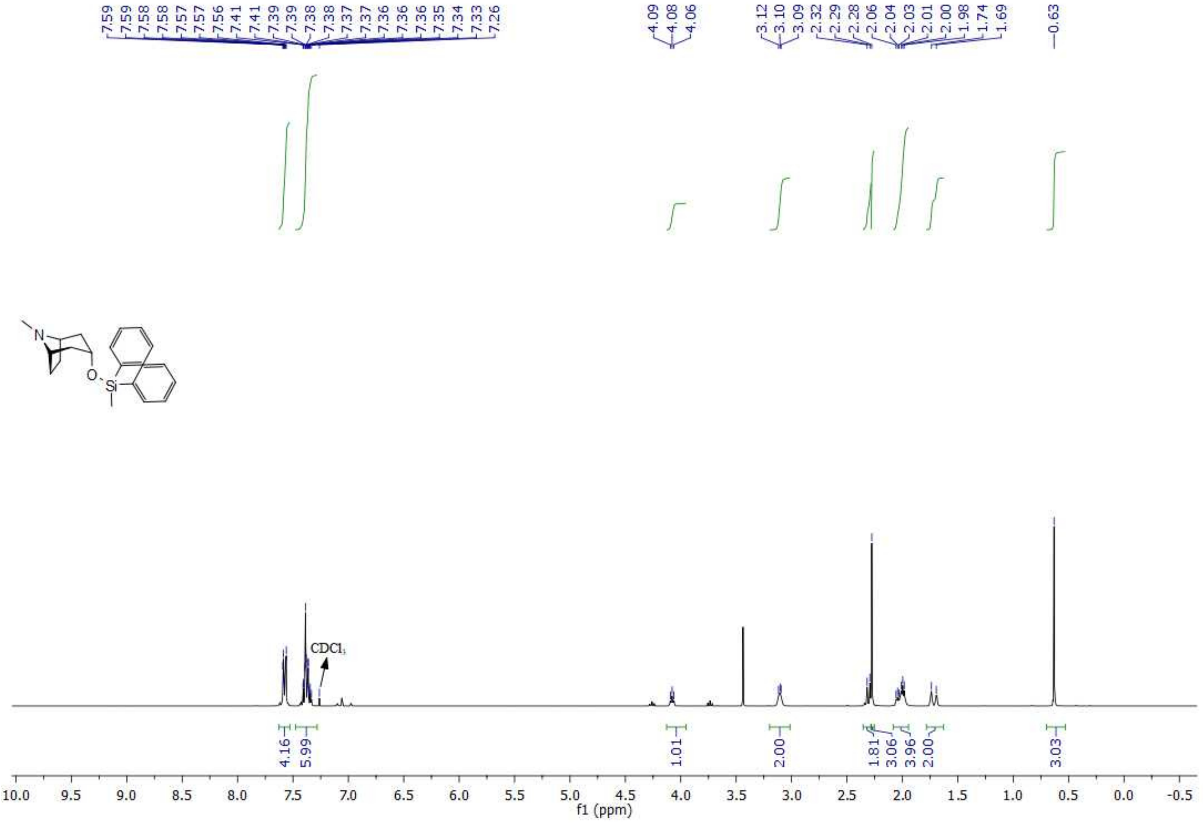

^{13}C NMR of (1R,3r,5S)-8-methyl-3-((methyldiphenylsilyl)oxy)-8-azabicyclo[3.2.1]octane (1f)

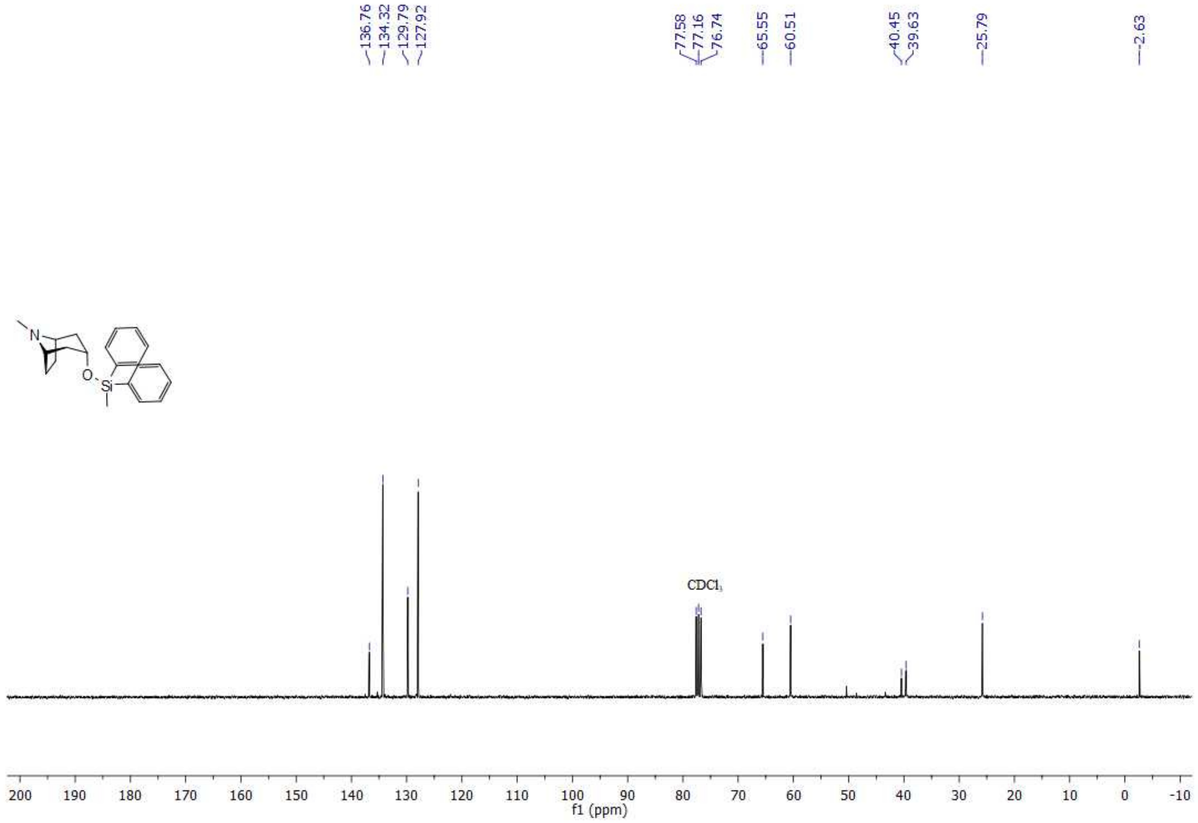

^{1}H NMR of (1R,3r,5S)-8-methyl-8-azabicyclo[3.2.1]octan-3-yl benzoate (1g)

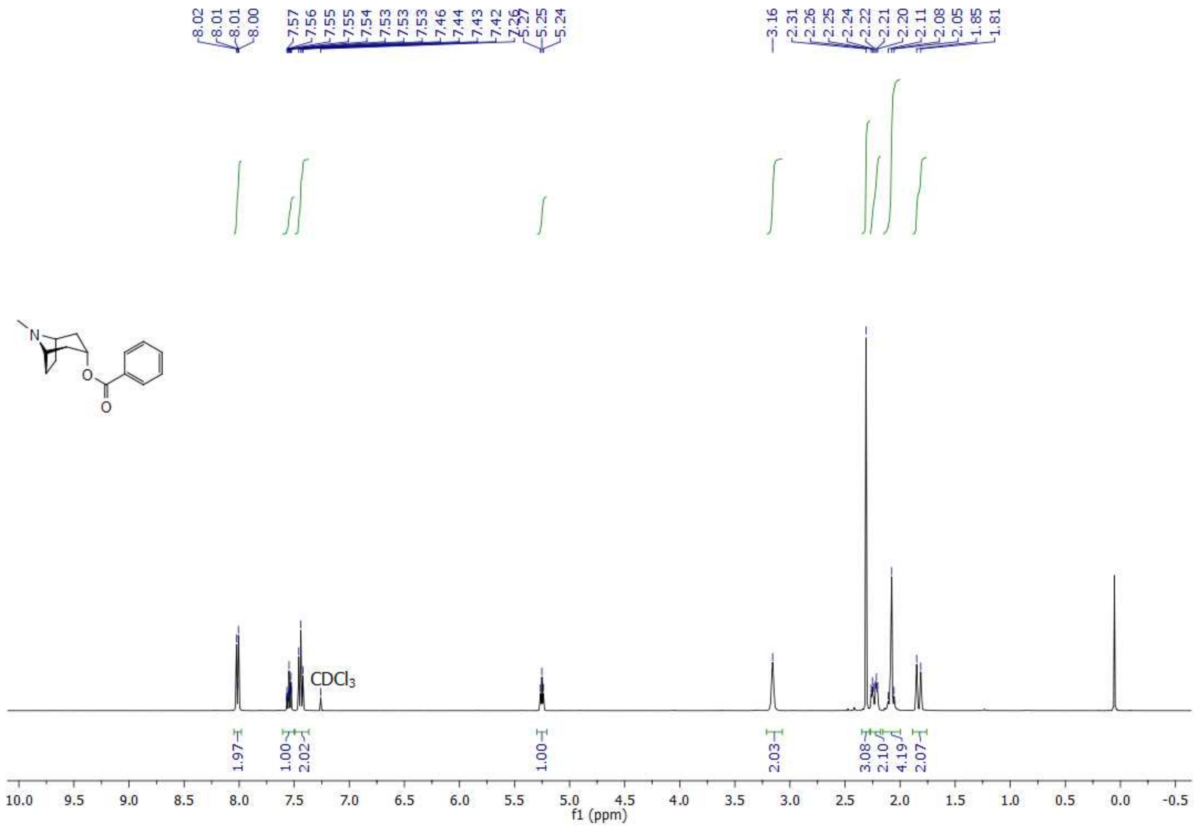

^{13}C NMR of (1R,3r,5S)-8-methyl-8-azabicyclo[3.2.1]octan-3-yl benzoate (1g)

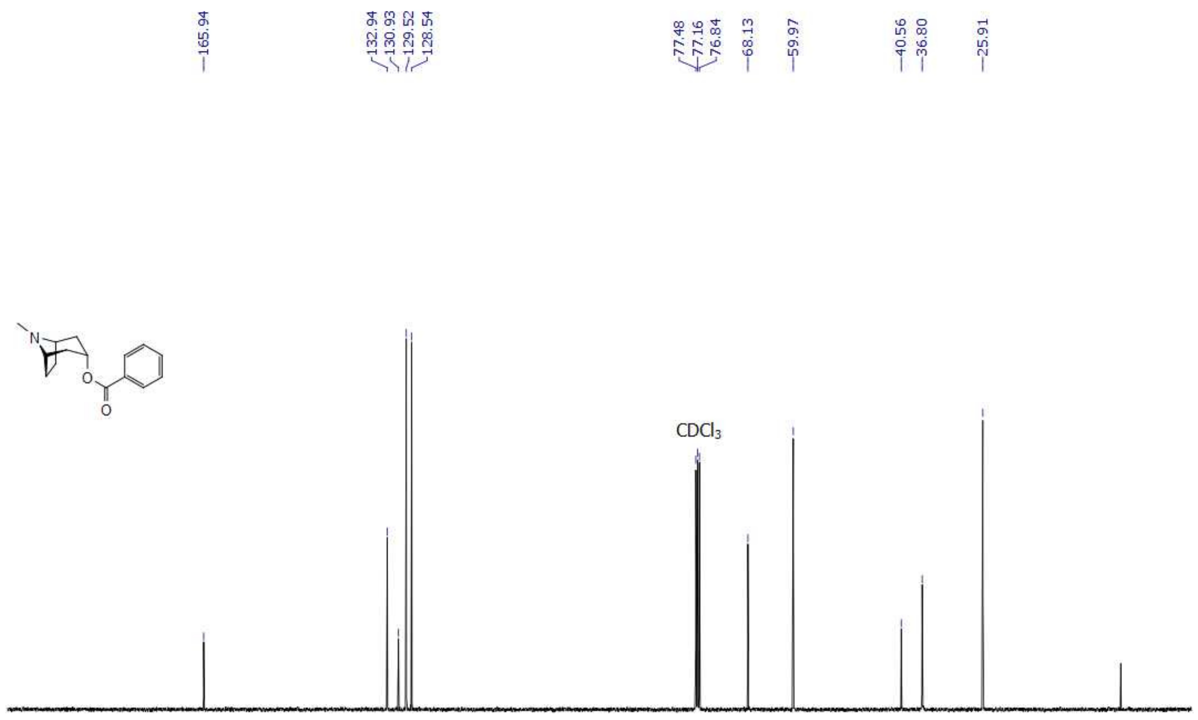

^{1}H NMR of (1R,3r,5S)-8-methyl-8-azabicyclo[3.2.1]octan-3-yl 4-(trifluoromethyl)benzoate (1h)

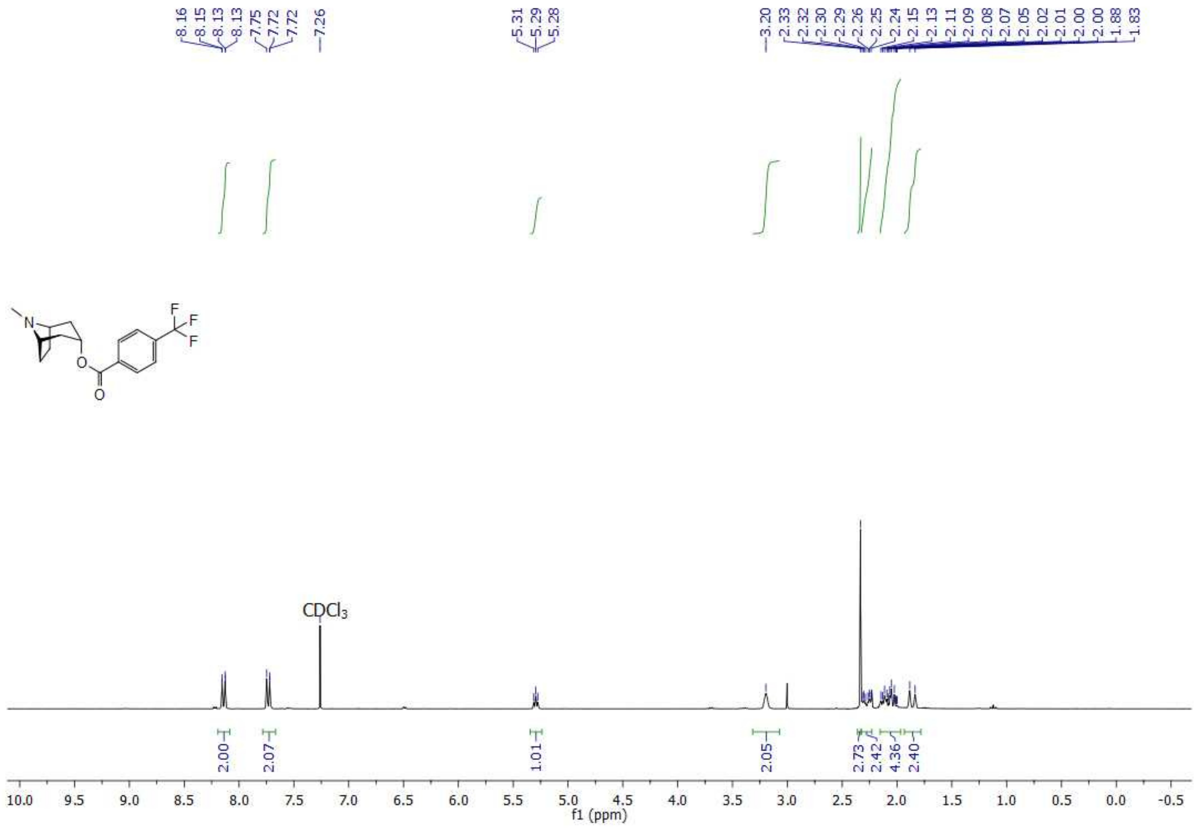

^{13}C NMR of (1R,3r,5S)-8-methyl-8-azabicyclo[3.2.1]octan-3-yl 4-(trifluoromethyl)benzoate (1h)

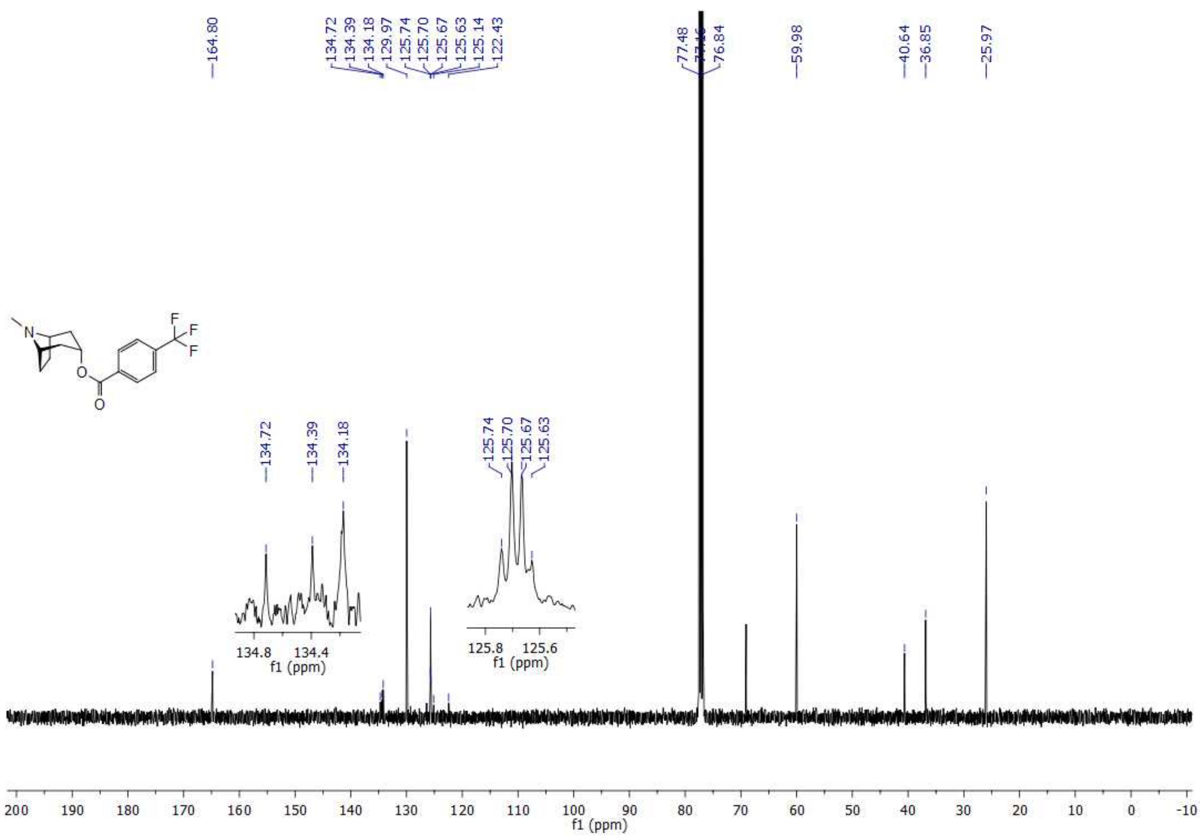

^{19}F NMR of 1h

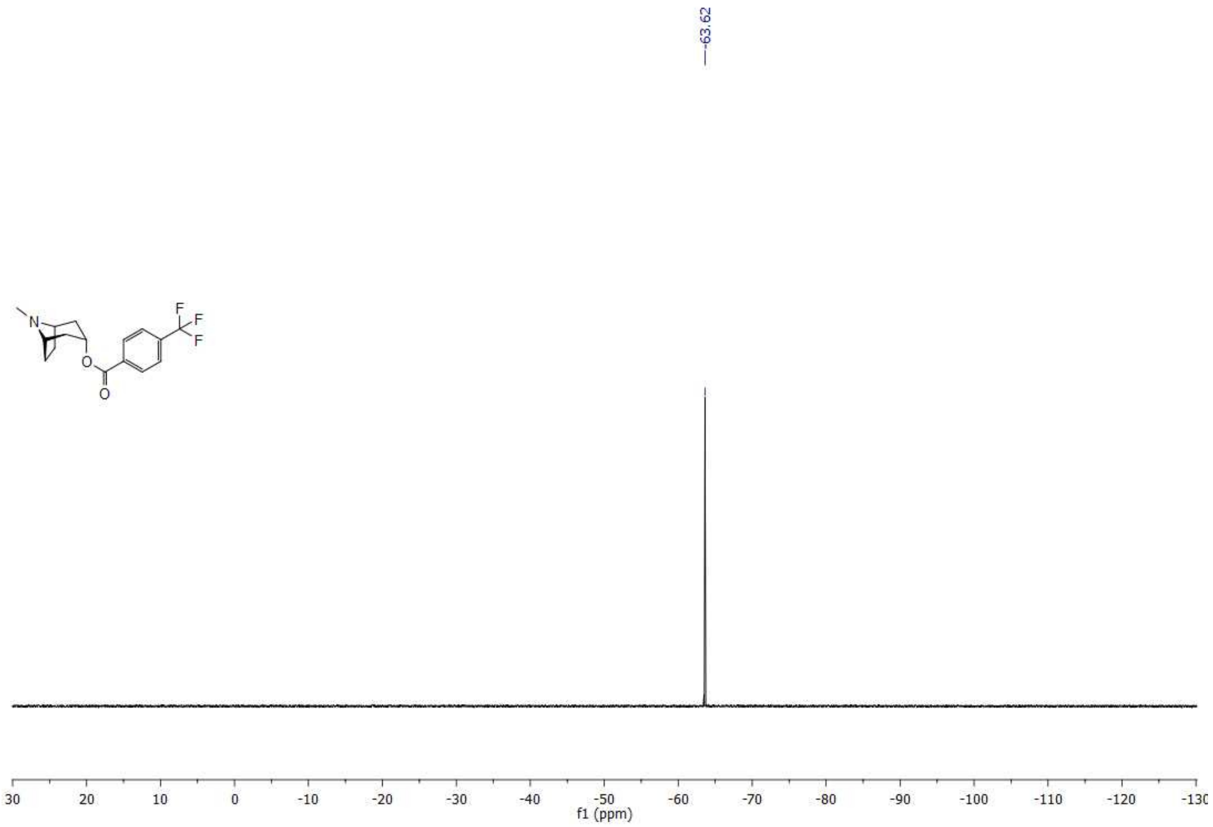

^{1}H NMR of (1R,3r,5S)-8-methyl-8-azabicyclo[3.2.1]octan-3-yl 3,4-dimethoxybenzoate (Convolvamine, 1i)

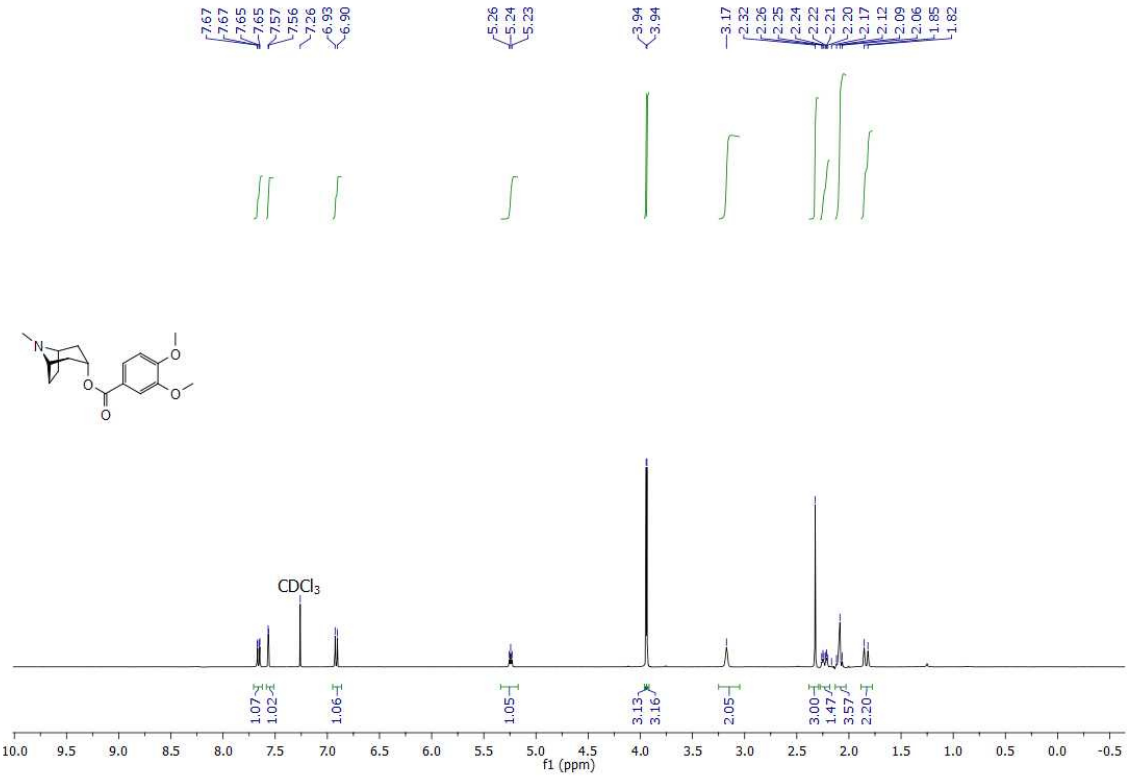

^{13}C NMR of (1R,3r,5S)-8-methyl-8-azabicyclo[3.2.1]octan-3-yl 3,4-dimethoxybenzoate (Convolvamine, 1i)

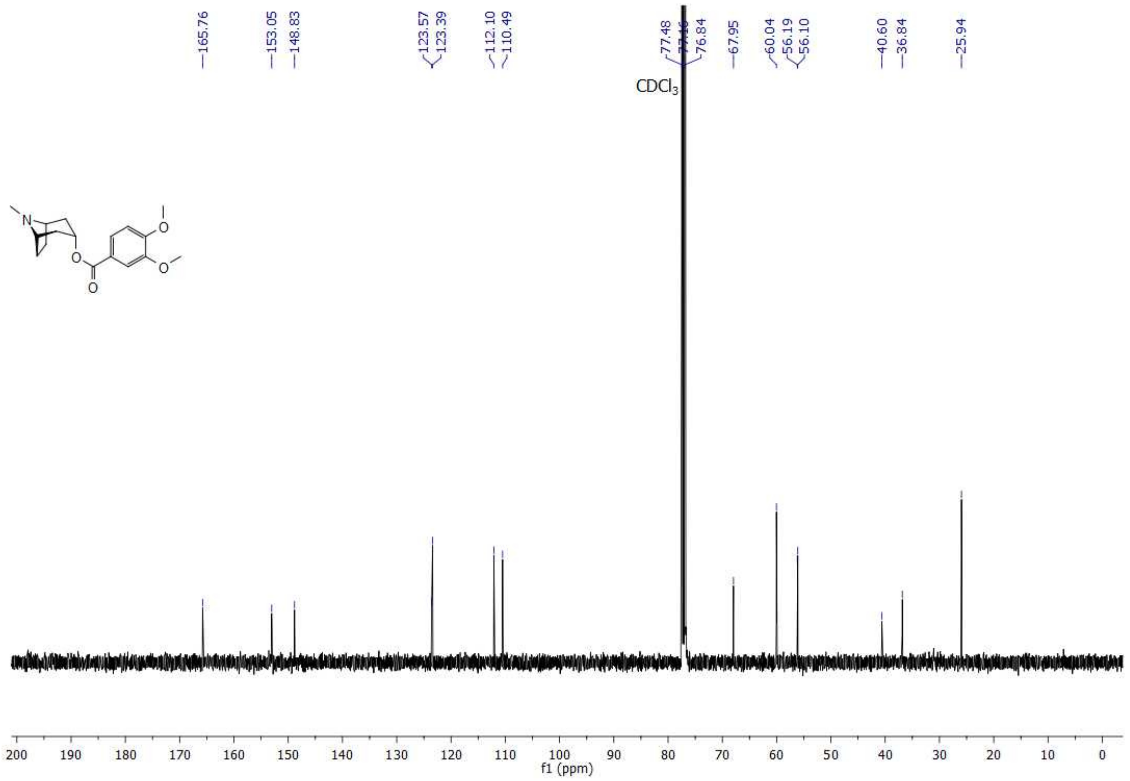

^{1}H NMR of 1-methyl-4-(phenylsulfonyl)piperazine (1j)

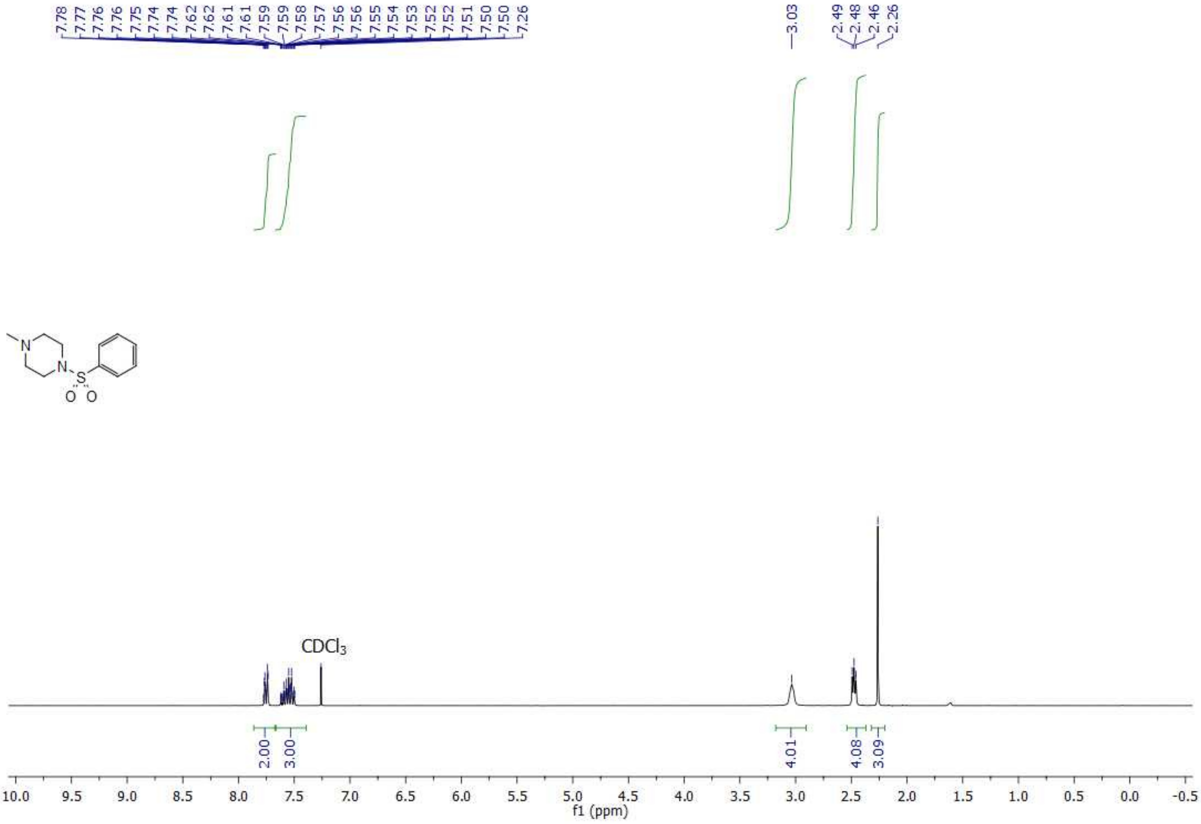

^{13}C NMR of 1-methyl-4-(phenylsulfonyl)piperazine (1j)

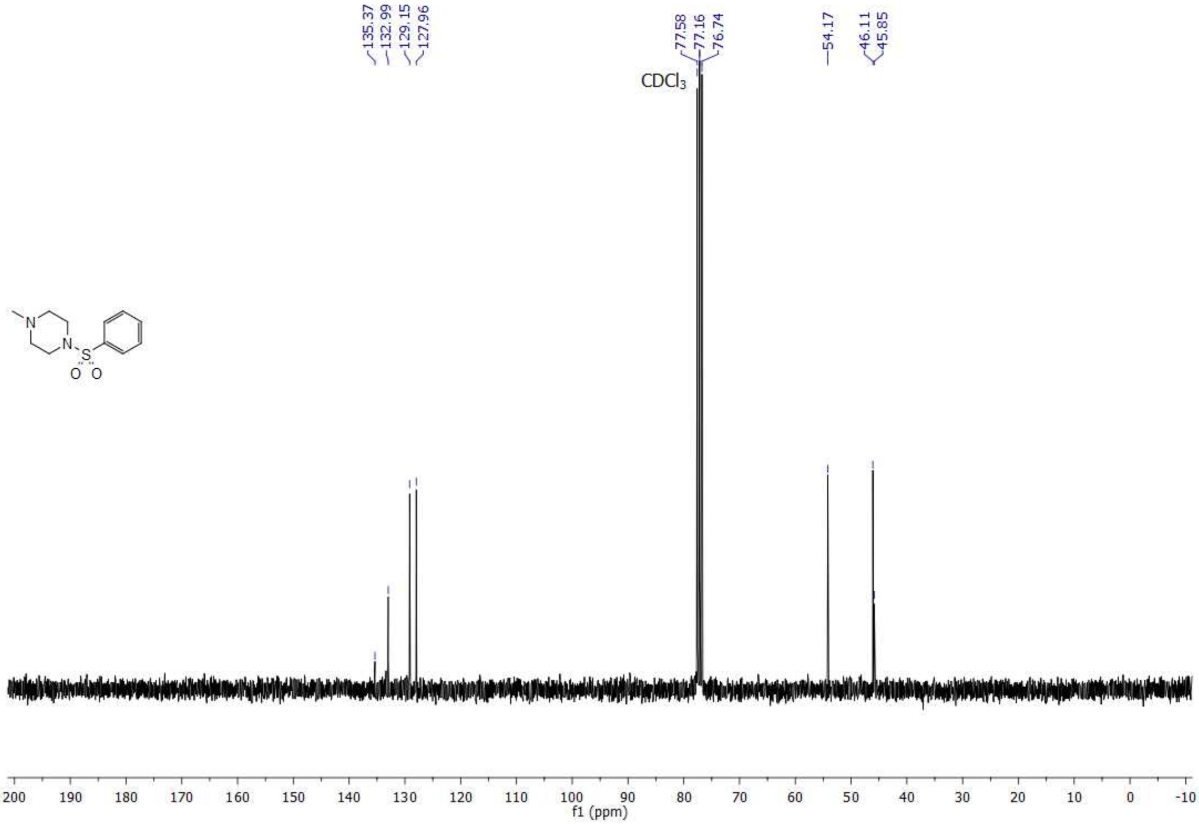

^{1}H NMR of 1-methyl-4-tosylpiperazine (1k)

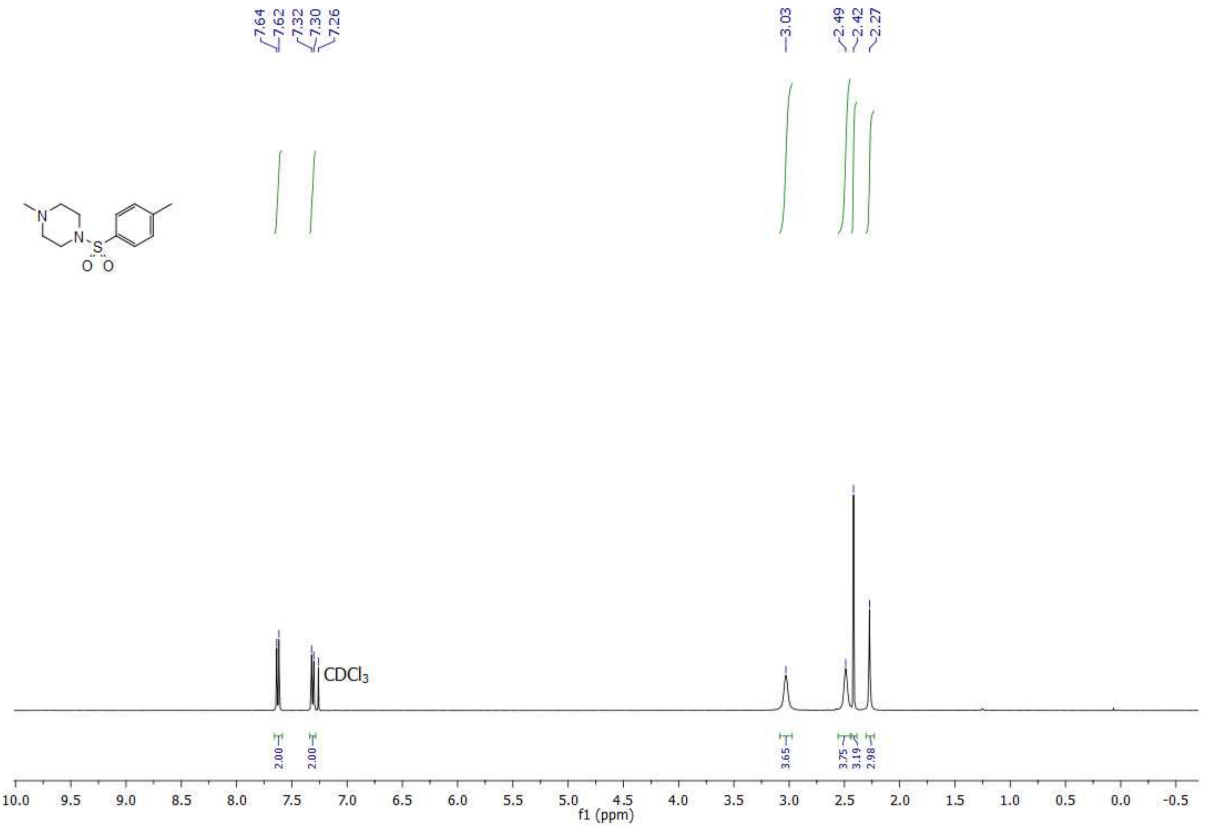

^{13}C NMR of 1-methyl-4-tosylpiperazine (1k)

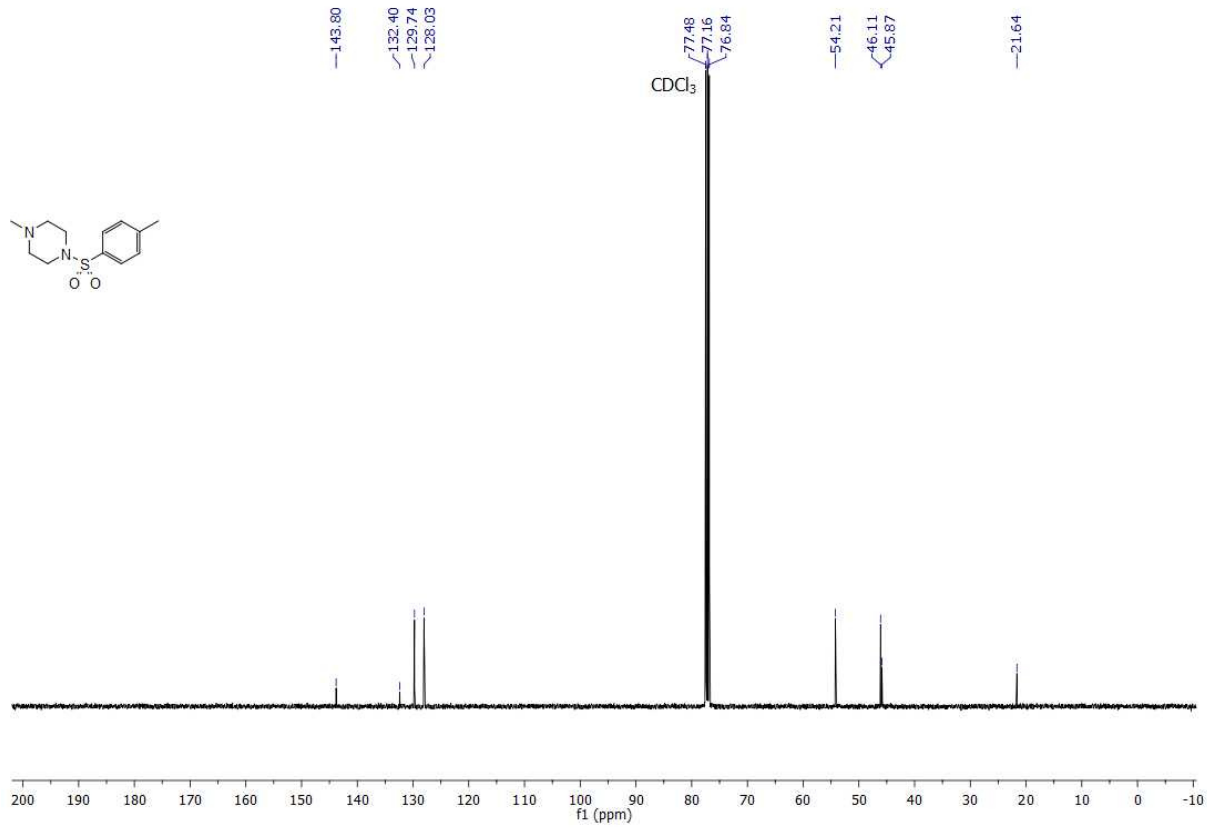

^{1}H NMR of 1-methyl-4-((4-(trifluoromethyl)phenyl)sulfonyl)piperazine (1l)

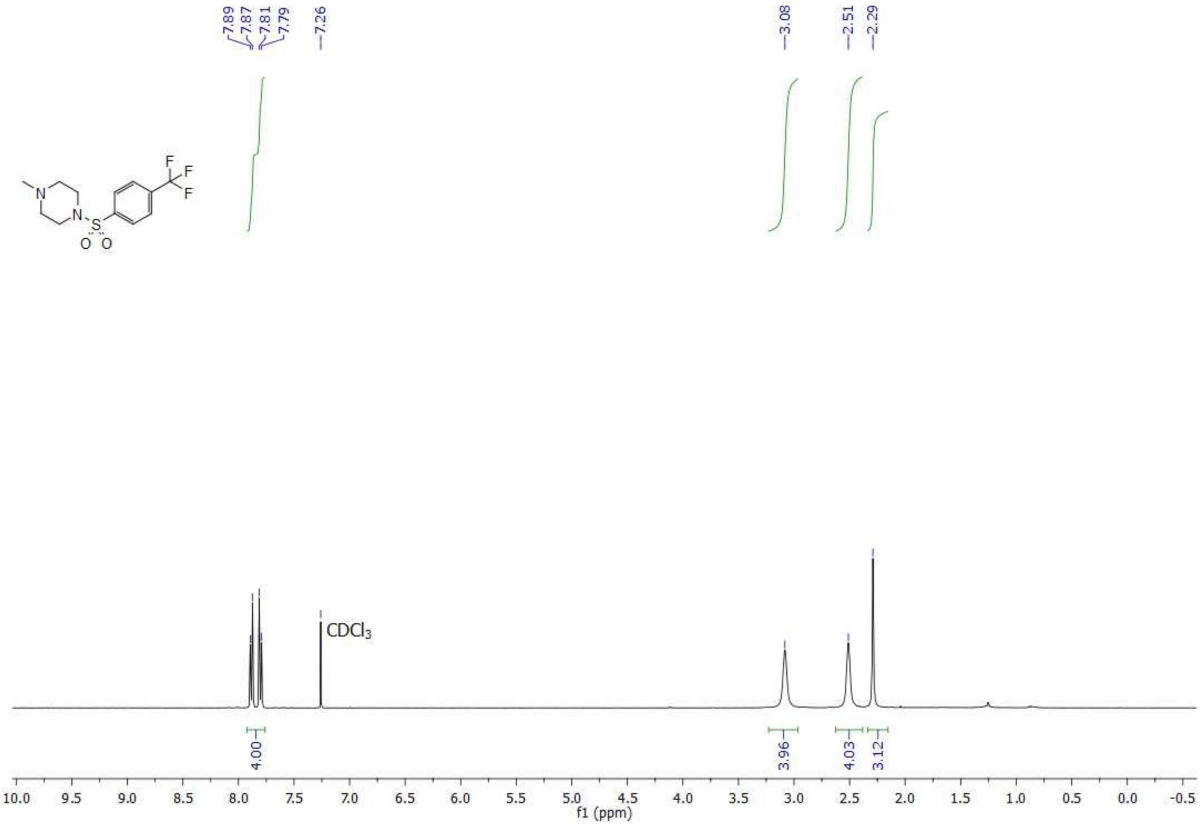

^{13}C NMR 1-methyl-4-((4-(trifluoromethyl)phenyl)sulfonyl)piperazine (1l)

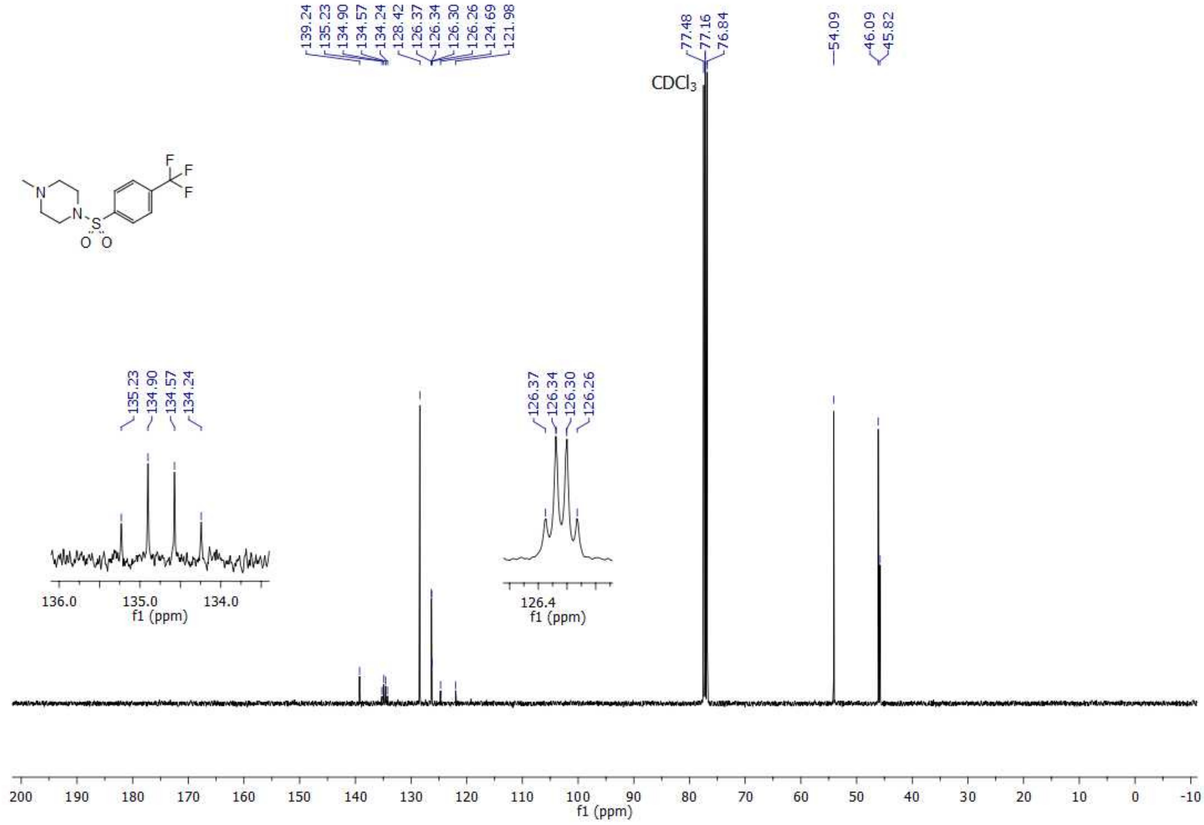

^{19}F NMR of 1-methyl-4-((4-(trifluoromethyl)phenyl)sulfonyl)piperazine (1l)

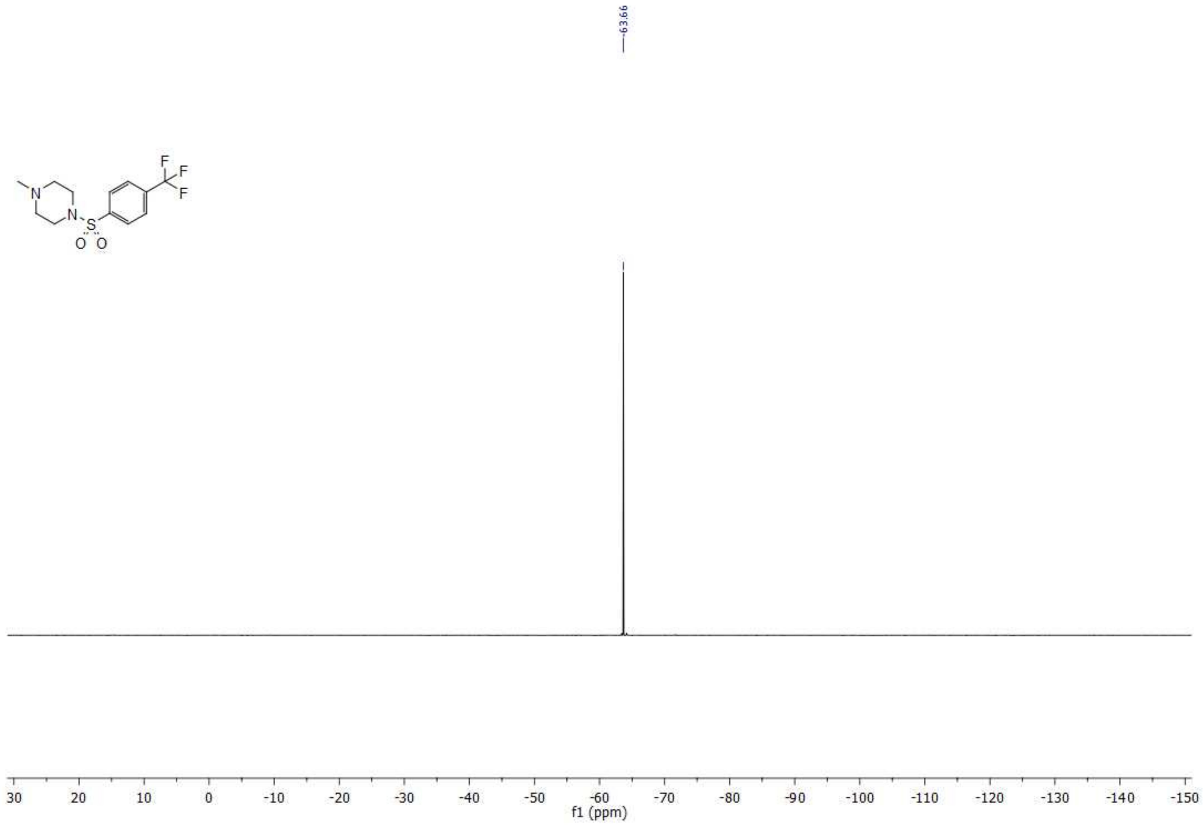

^{1}H NMR of 1-((4-bromophenyl)sulfonyl)-4-methylpiperazine (1m)

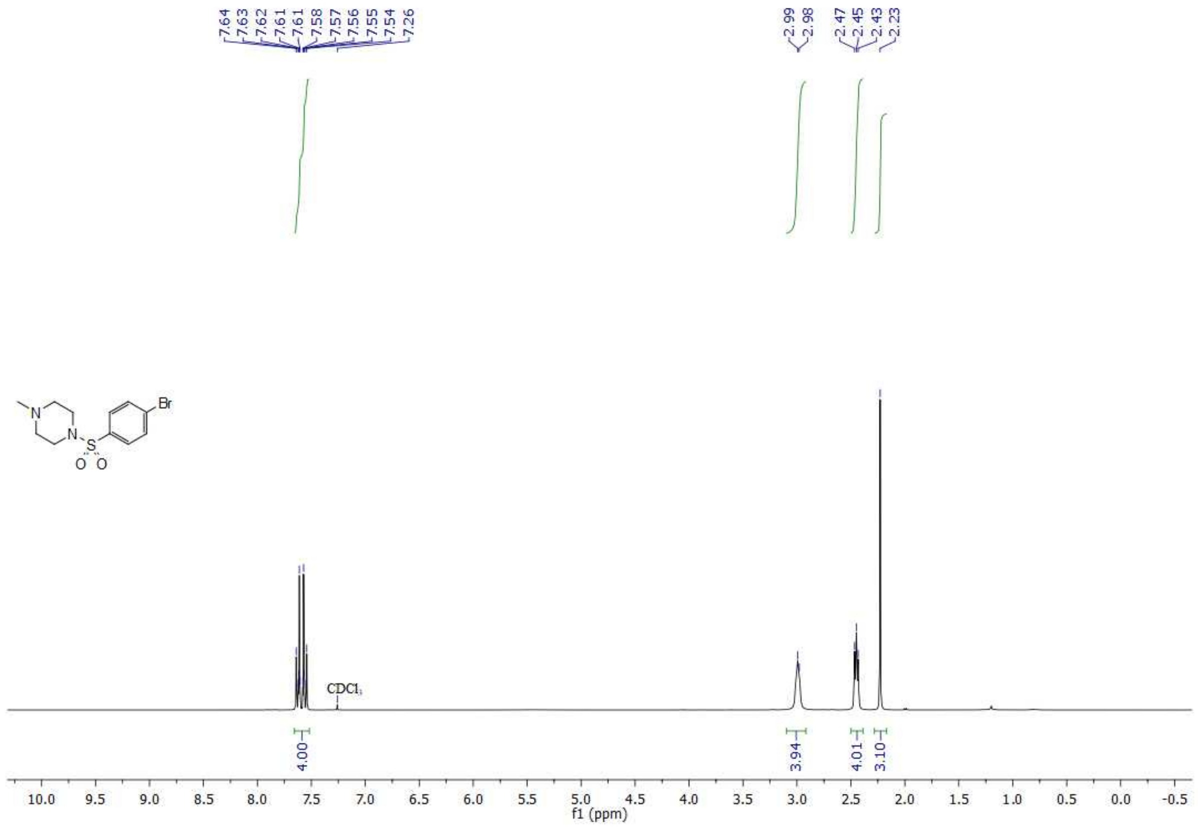

^{13}C NMR 1-((4-bromophenyl)sulfonyl)-4-methylpiperazine (1m)

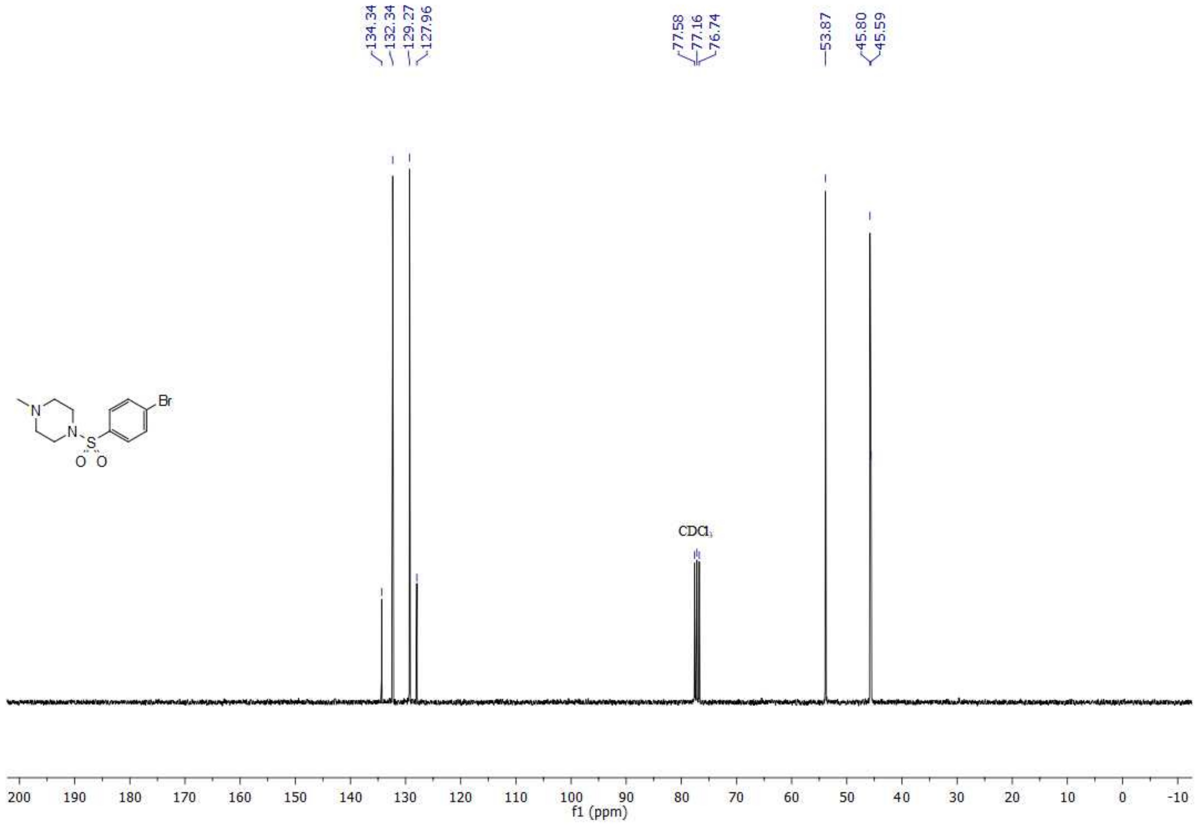

^{1}H NMR of (1S,4S)-2-methyl-5-((4-(trifluoromethyl)phenyl)sulfonyl)-2,5-diazabicyclo[2.2.1]heptane (1n)

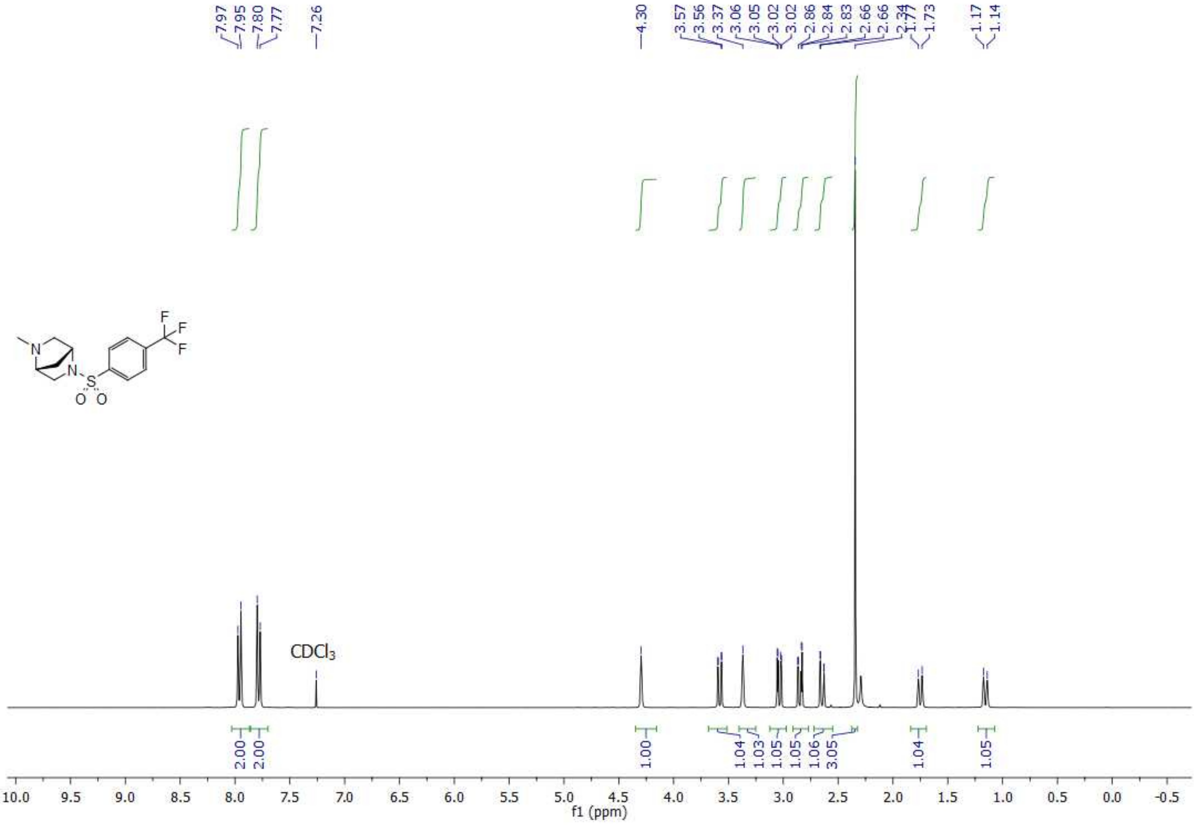

^{13}C NMR of (1S,4S)-2-methyl-5-((4-(trifluoromethyl)phenyl)sulfonyl)-2,5-diazabicyclo[2.2.1]heptane (1n)

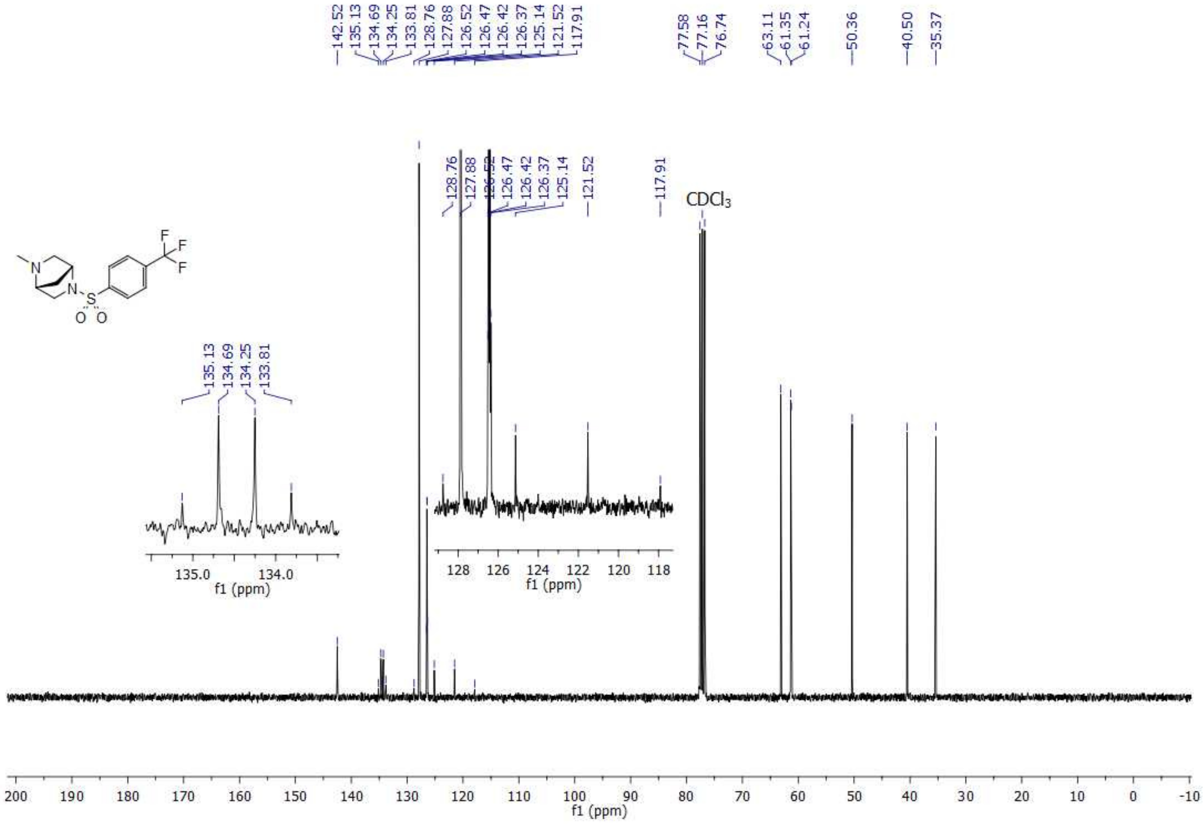

^{19}F NMR of (1S,4S)-2-methyl-5-((4-(trifluoromethyl)phenyl)sulfonyl)-2,5-diazabicyclo[2.2.1]heptane (1n)

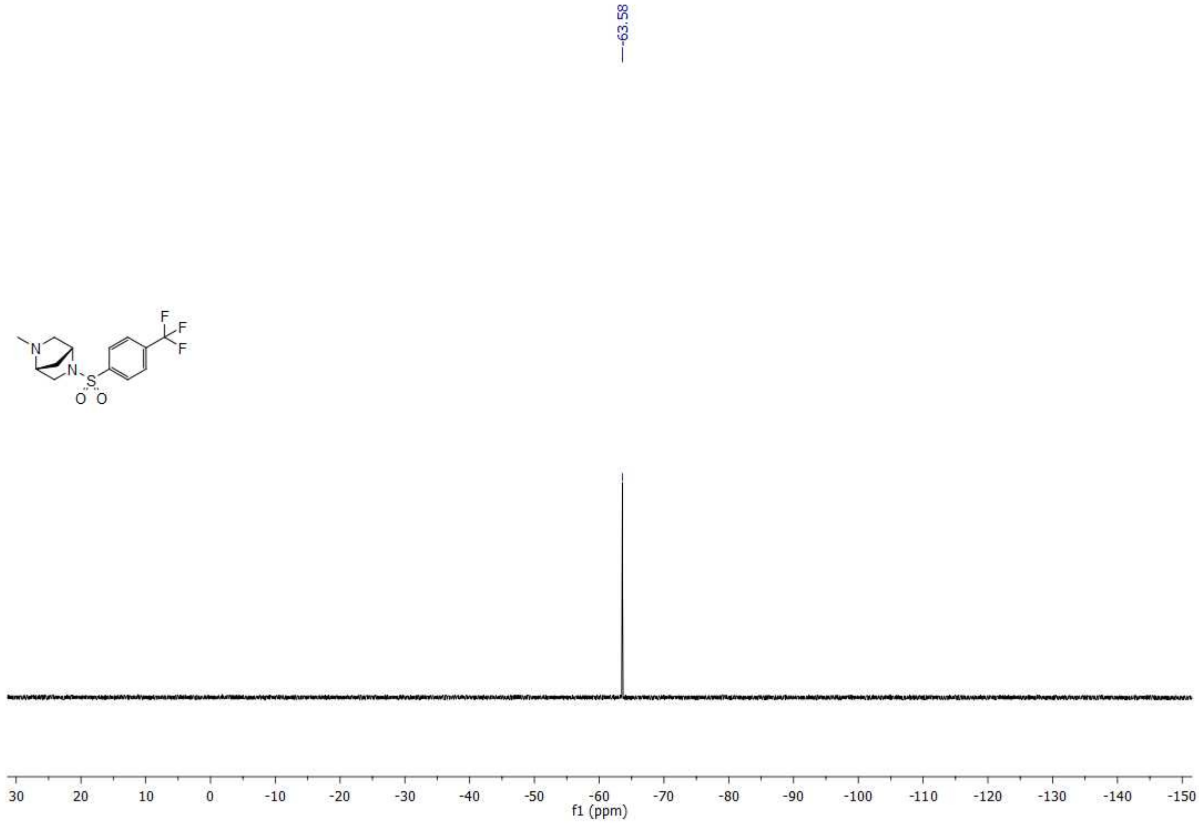

^{1}H NMR of (4-methylpiperazin-1-yl)(phenyl)methanone (1o)

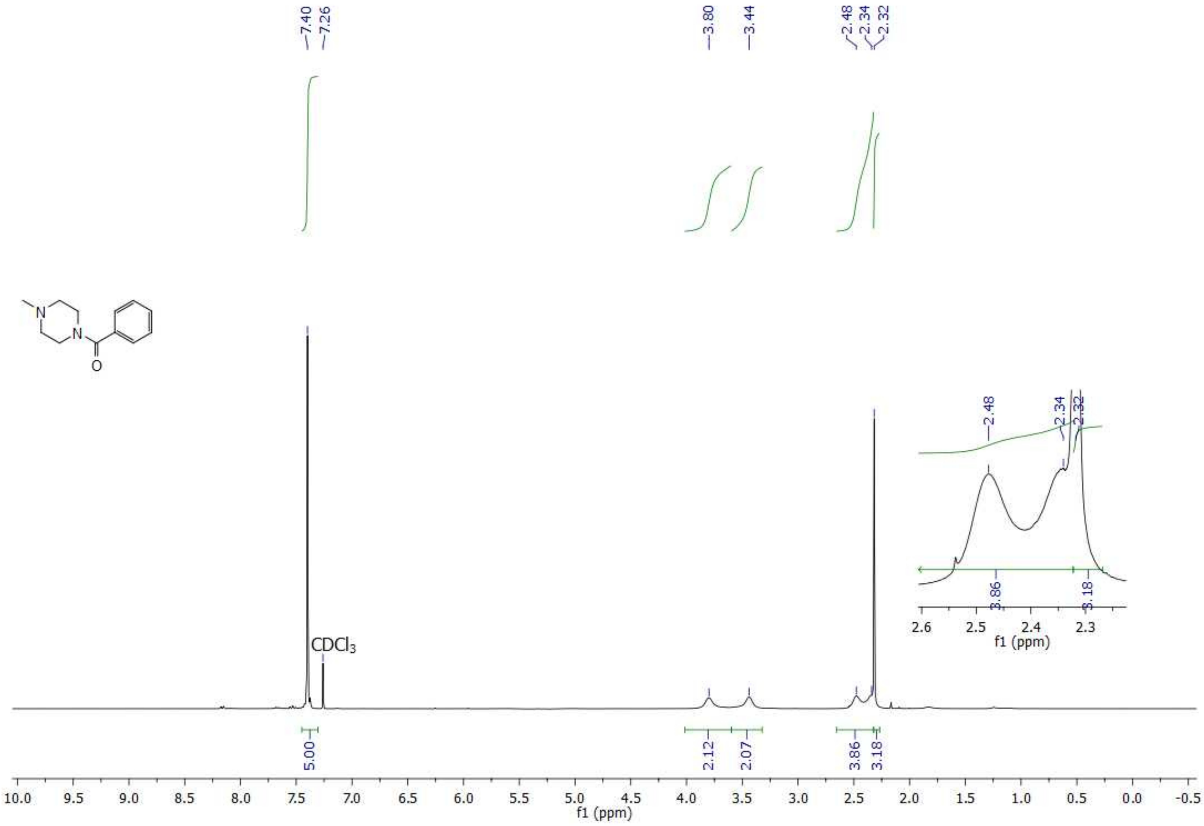

^{13}C NMR of (4-methylpiperazin-1-yl)(phenyl)methanone (1o)

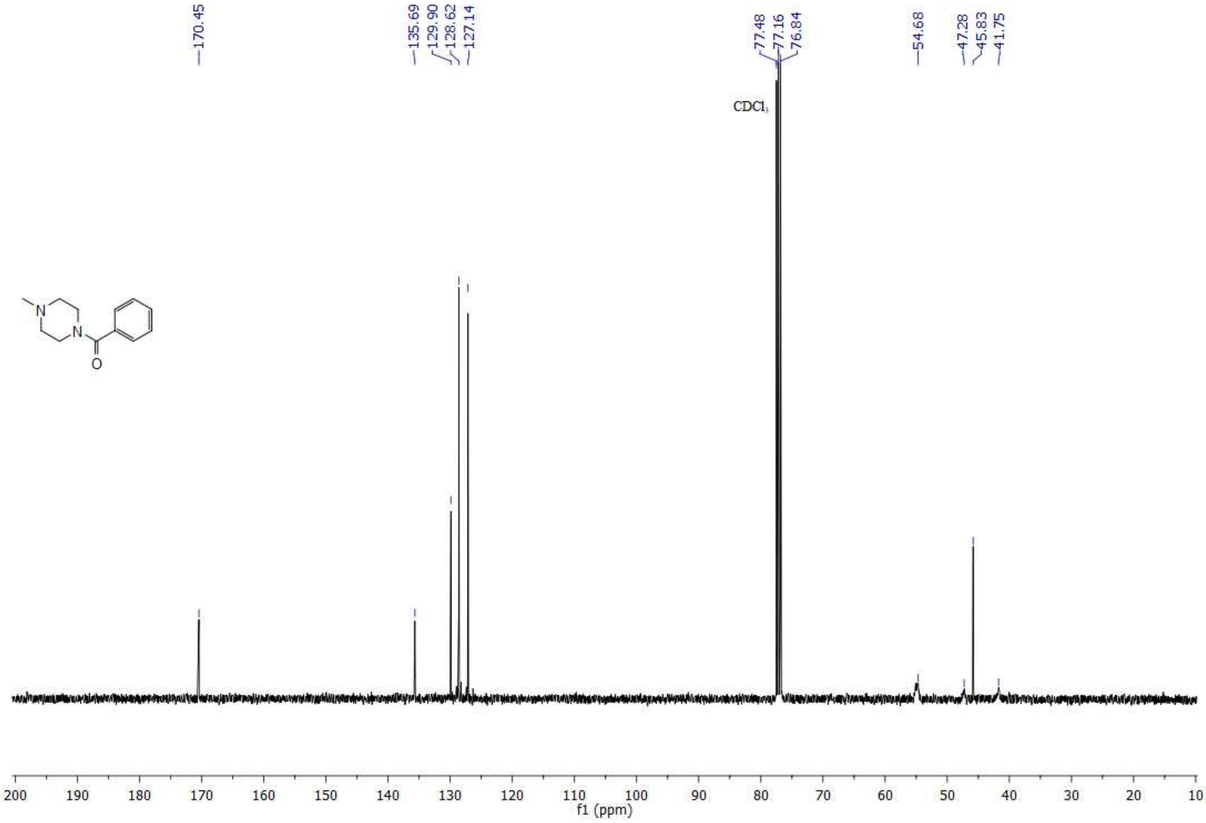

^{1}H NMR of (4b*S*,9*S*)-3-methoxy-6,7,8,8a,9,10-hexahydro-5*H*-9,4b-(epiminoethano)phenanthrene-11-carbaldehyde (2a)

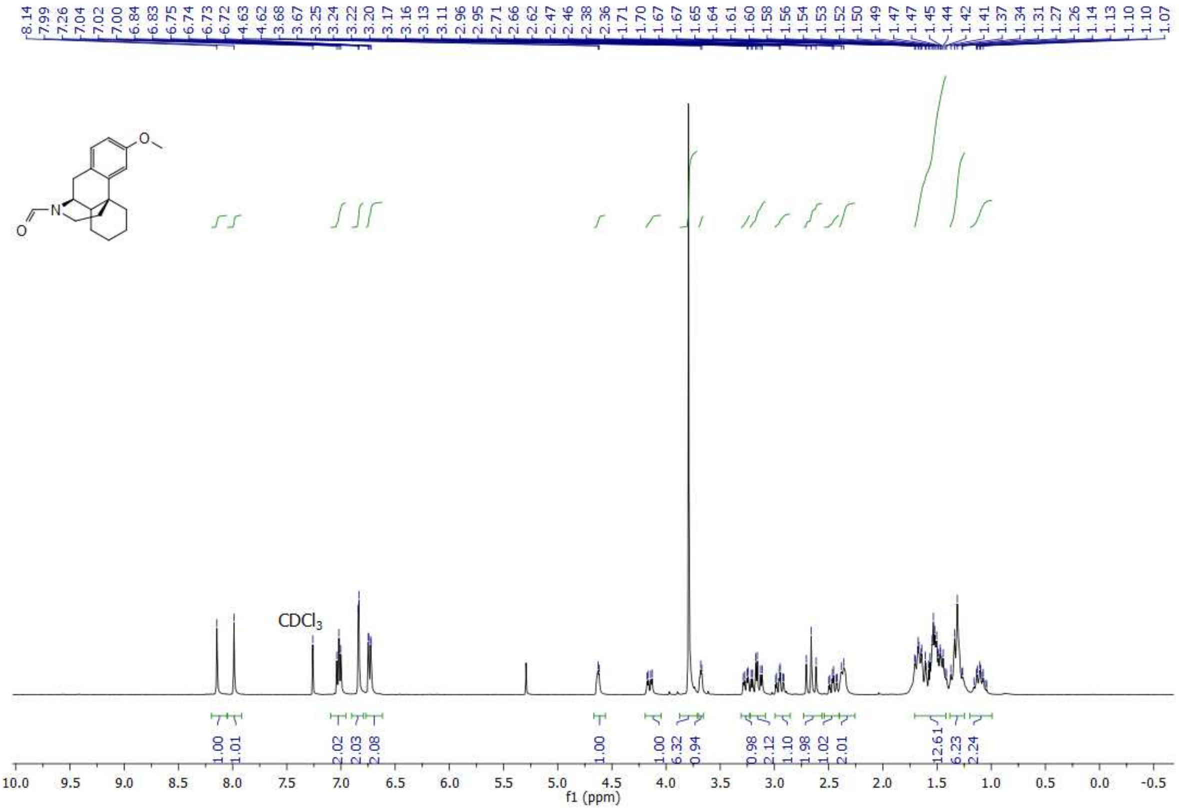

^{13}C NMR of (4b*S*,9*S*)-3-methoxy-6,7,8,8a,9,10-hexahydro-5*H*-9,4b-(epiminoethano)phenanthrene-11-carbaldehyde (2a)

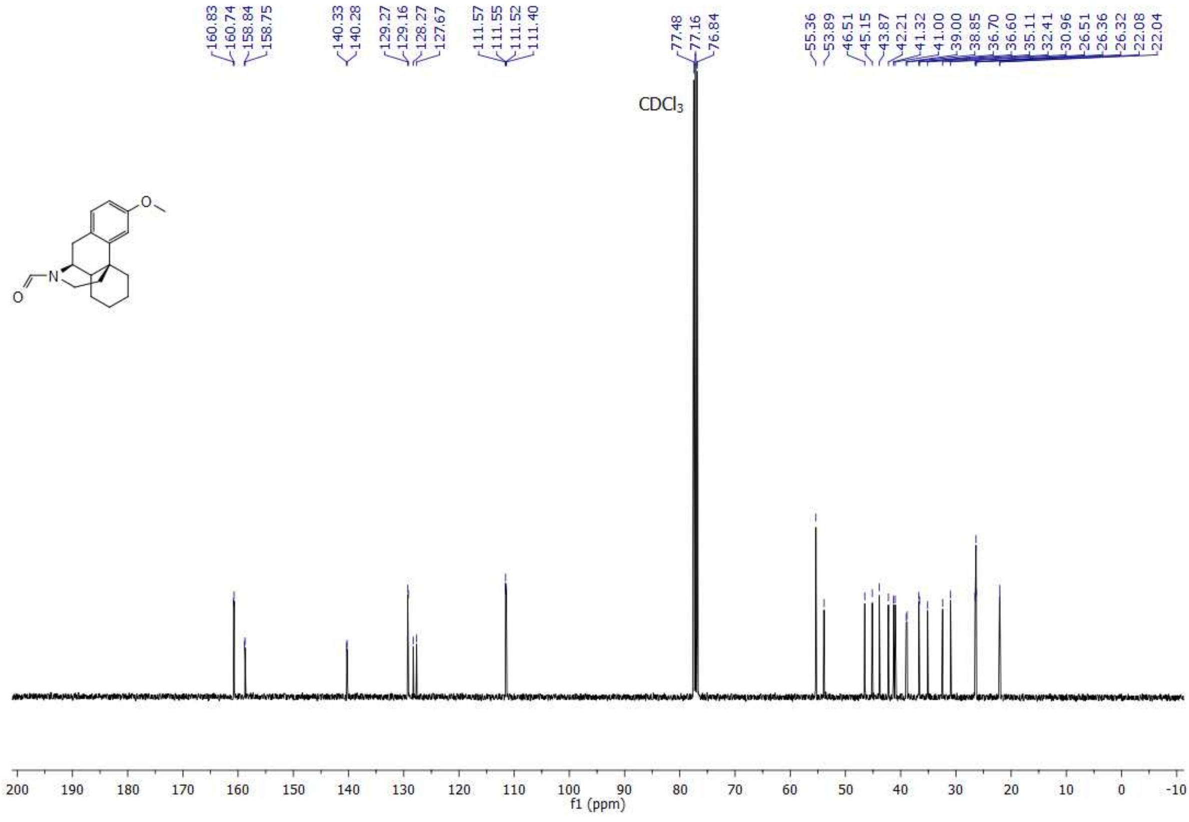

^{1}H NMR of (1R,3r,5S)-3-hydroxy-8-azabicyclo[3.2.1]octane-8-carbaldehyde (2b)

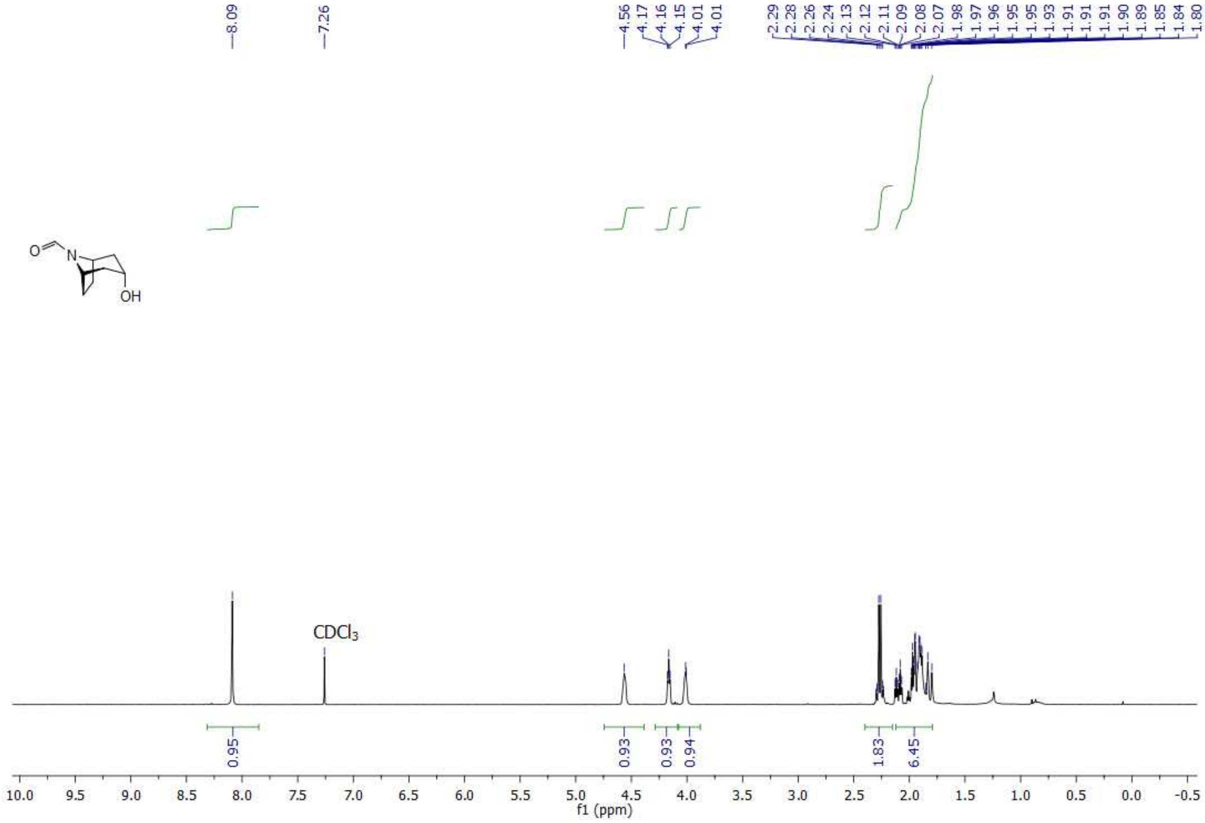

^{13}C NMR of (1R,3r,5S)-3-hydroxy-8-azabicyclo[3.2.1]octane-8-carbaldehyde (2b)

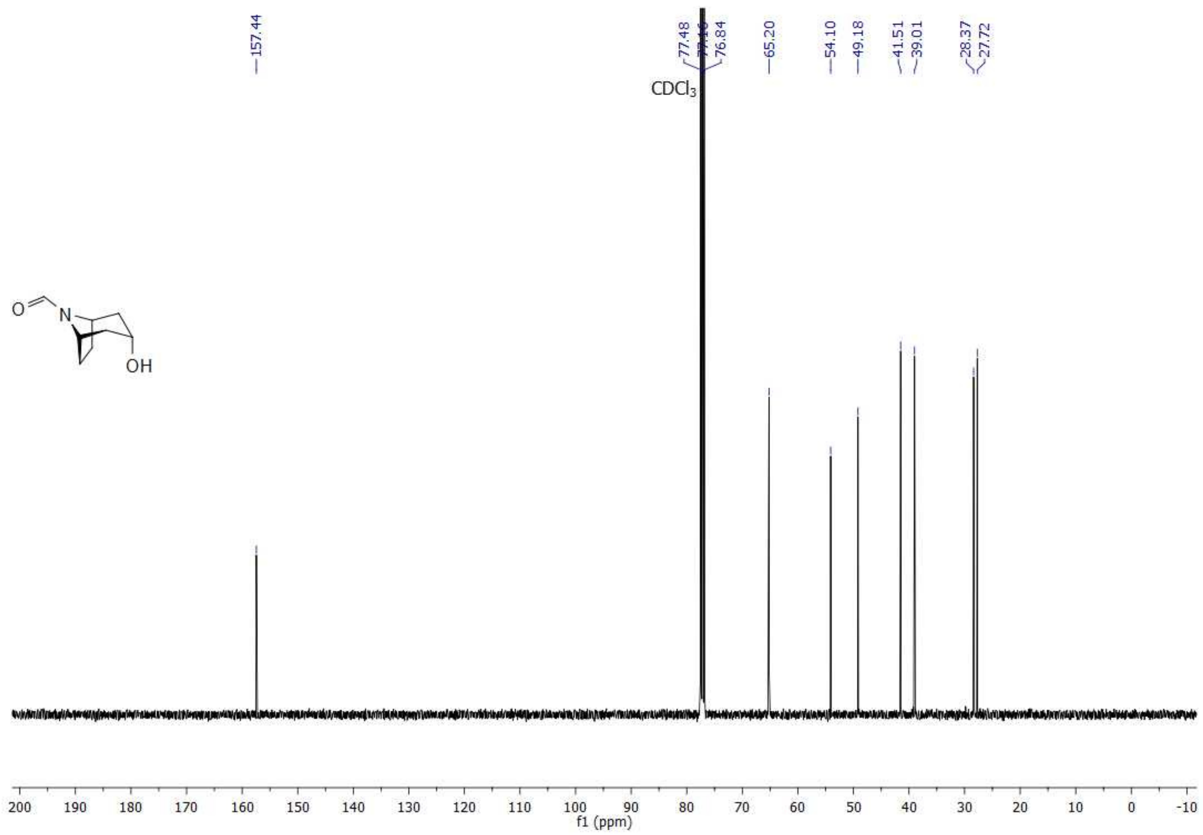

^{1}H NMR of (1R,5S)-8-azabicyclo[3.2.1]octane-8-carbaldehyde (2c)

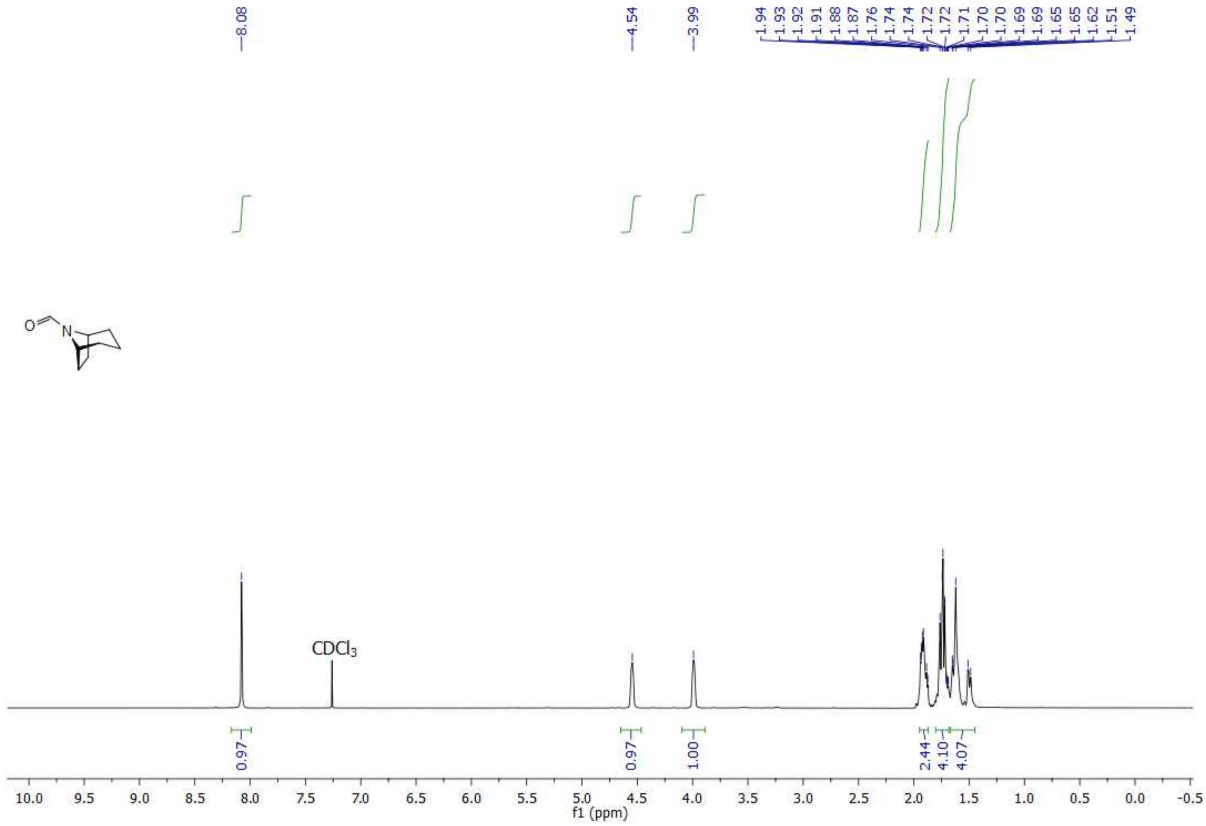

^{13}C NMR of (1R,5S)-8-azabicyclo[3.2.1]octane-8-carbaldehyde (2c)

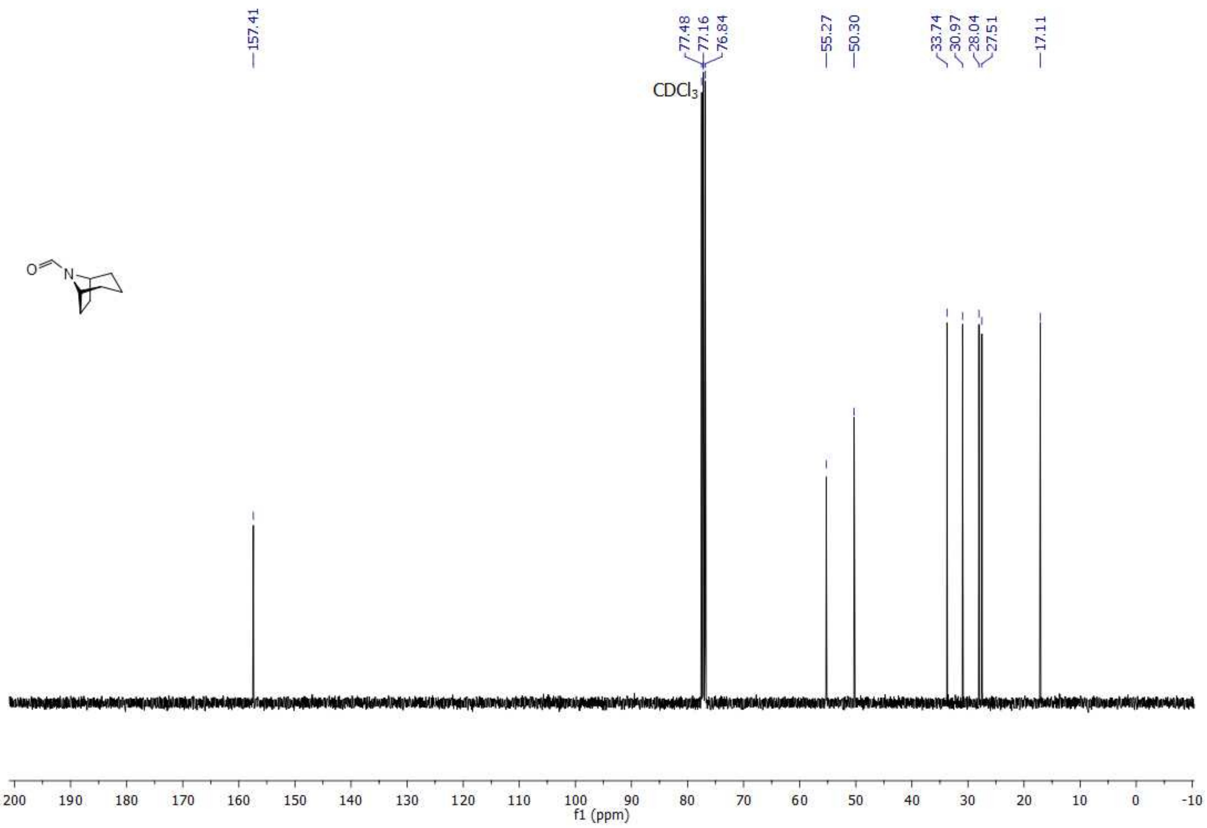

^{1}H NMR of (1R,3r,5S)-8-formyl-8-azabicyclo[3.2.1]octan-3-yl (S)-3-hydroxy-2-phenylpropanoate (2d)

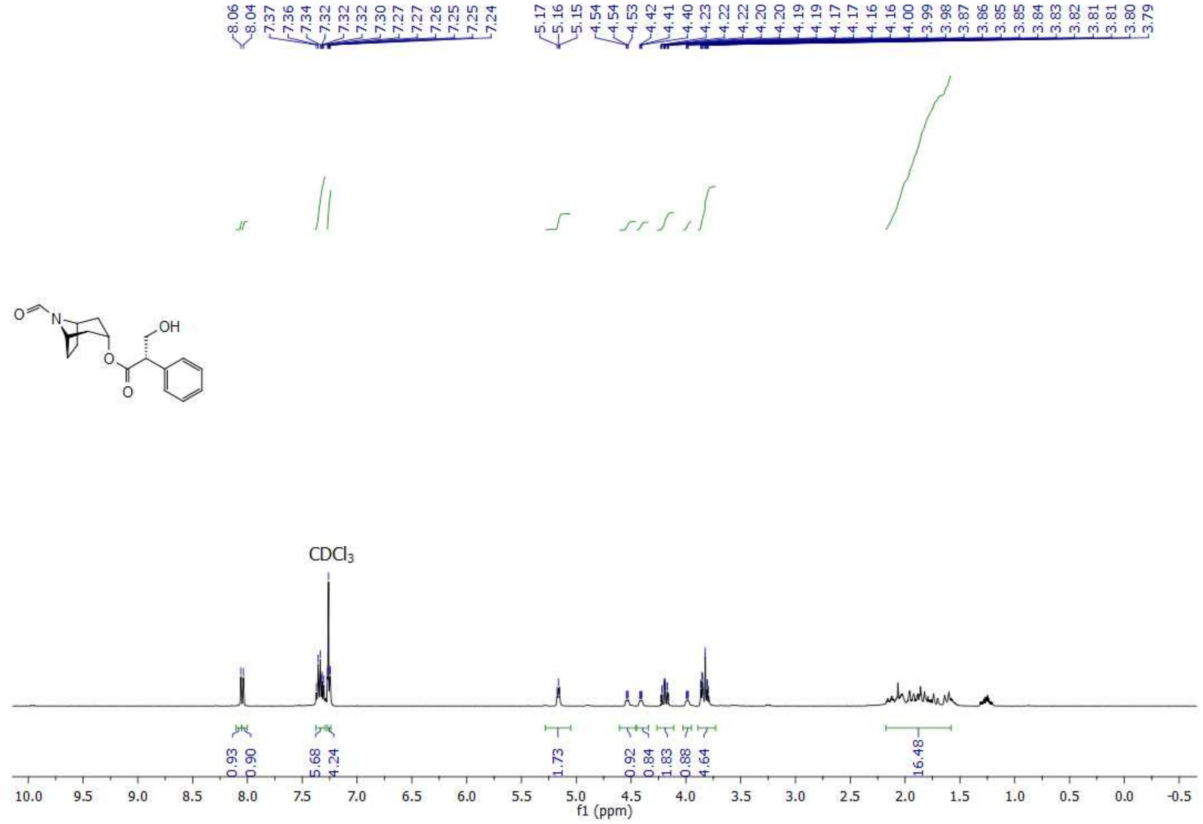

^{13}C NMR of ^{1}H NMR of (1R,3r,5S)-8-formyl-8-azabicyclo[3.2.1]octan-3-yl (S)-3-hydroxy-2-phenylpropanoate (2d)

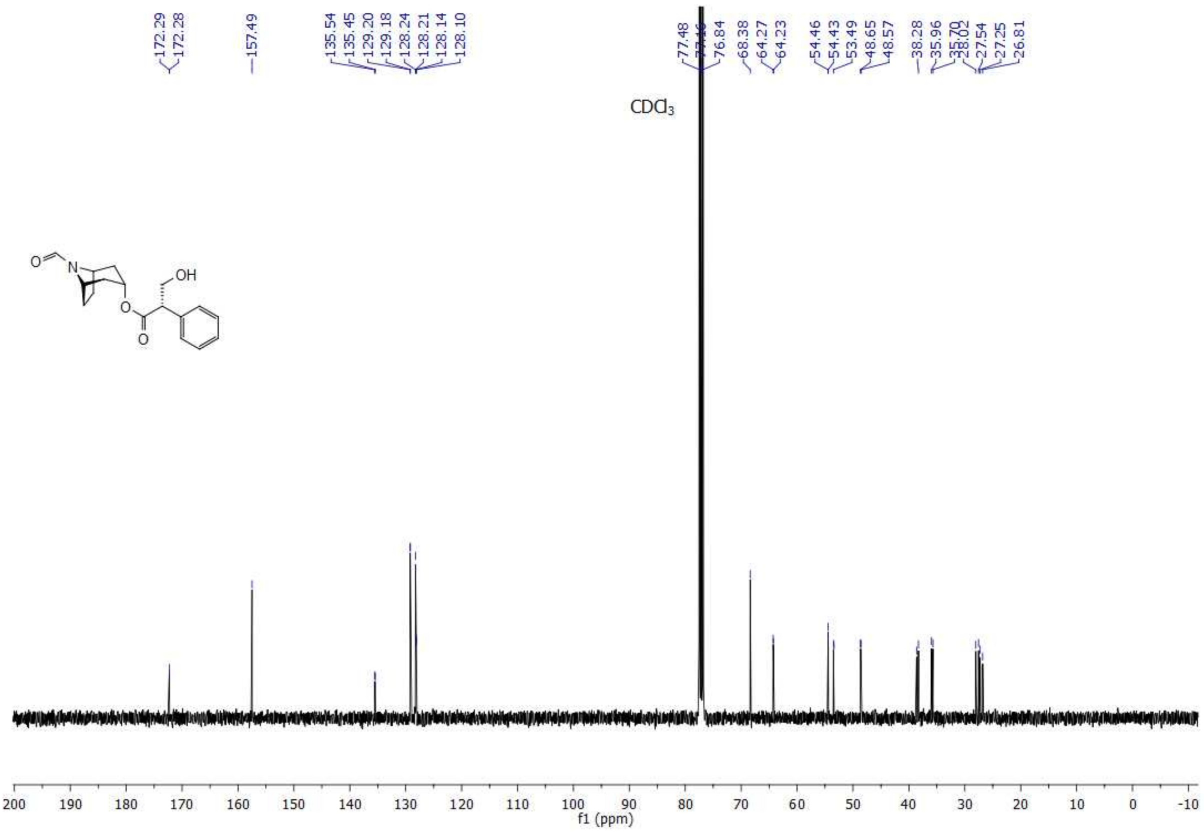

^{1}H NMR of (1R,2R,4S,5S,7s)-9-formyl-3-oxa-9-azatricyclo[3.3.1.02,4]nonan-7-yl (S)-3-hydroxy-2-phenylpropanoate (2e)

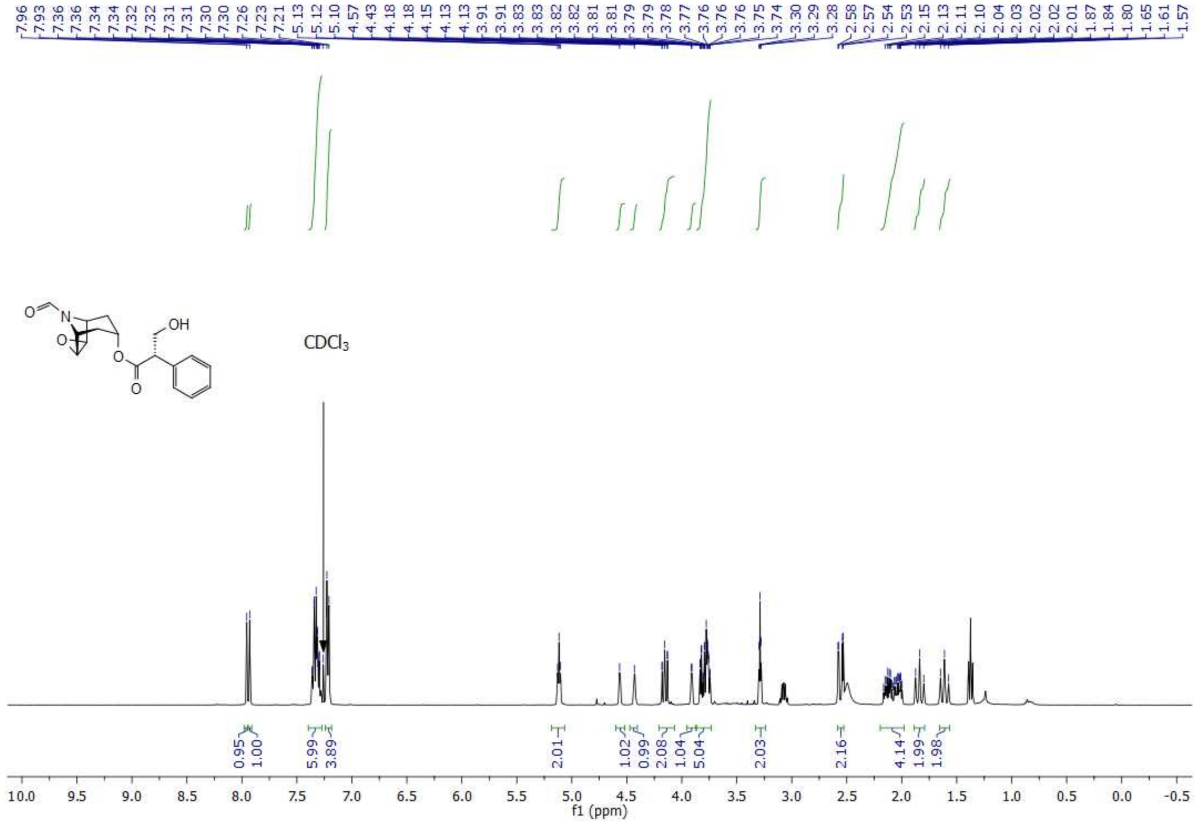

^{13}C NMR of (1R,2R,4S,5S,7s)-9-formyl-3-oxa-9-azatricyclo[3.3.1.02,4]nonan-7-yl (S)-3-hydroxy-2-phenylpropanoate (2e)

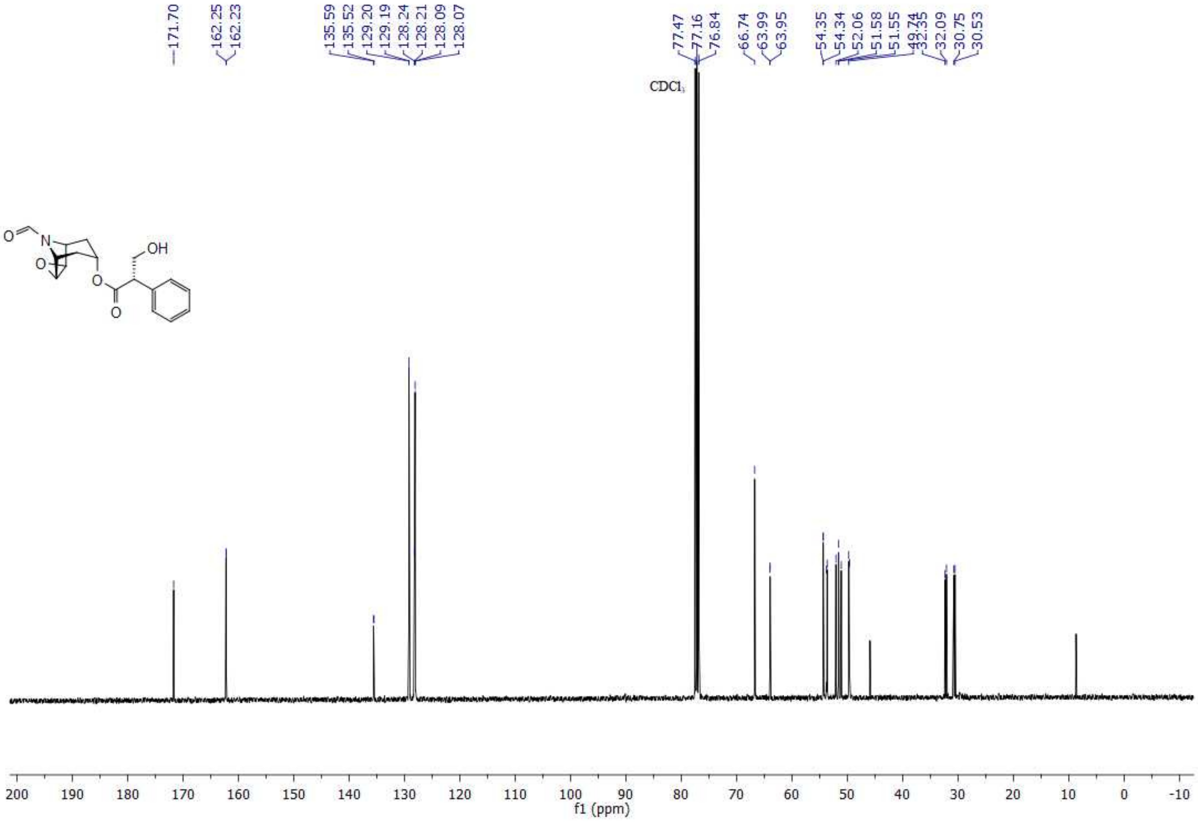

^{1}H NMR of (1R,3r,5S)-3-((methyldiphenylsilyl)oxy)-8-azabicyclo[3.2.1]octane-8-carbaldehyde (2f)

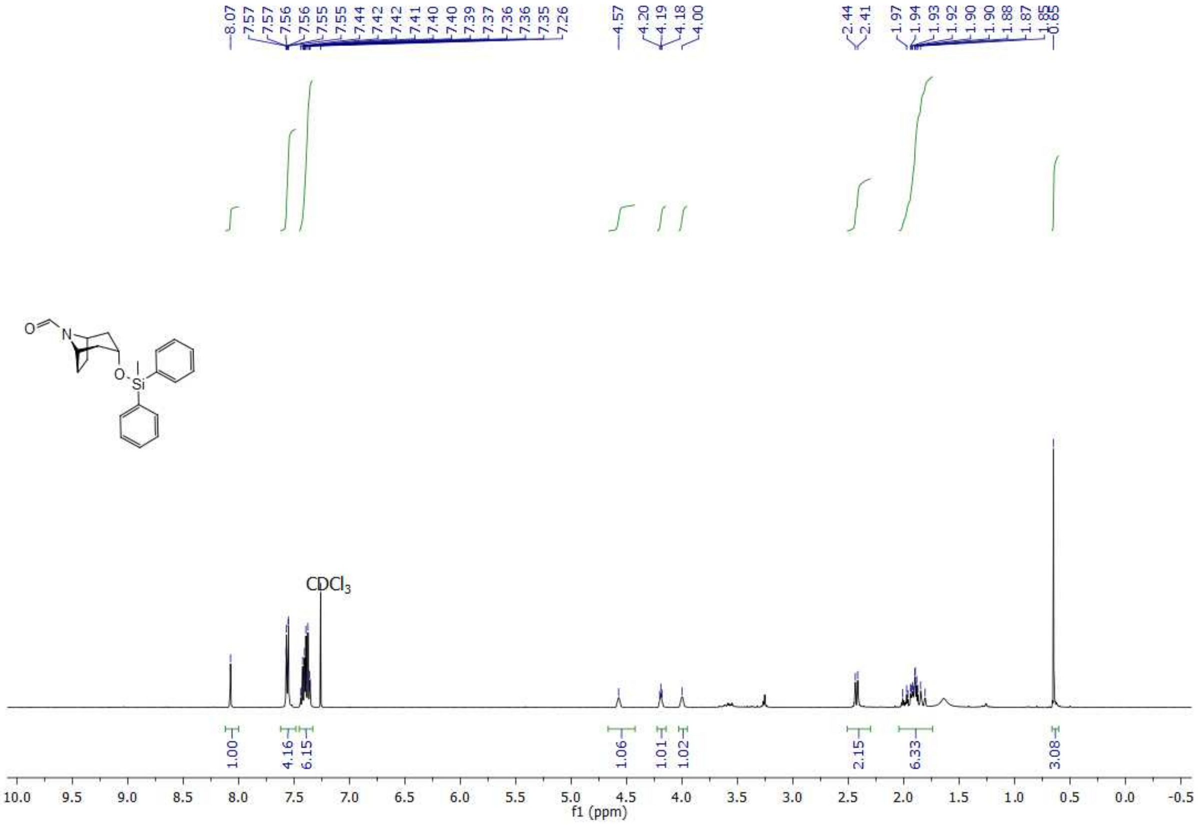

^{13}C NMR of (1R,3r,5S)-3-((methyldiphenylsilyl)oxy)-8-azabicyclo[3.2.1]octane-8-carbaldehyde (2f)

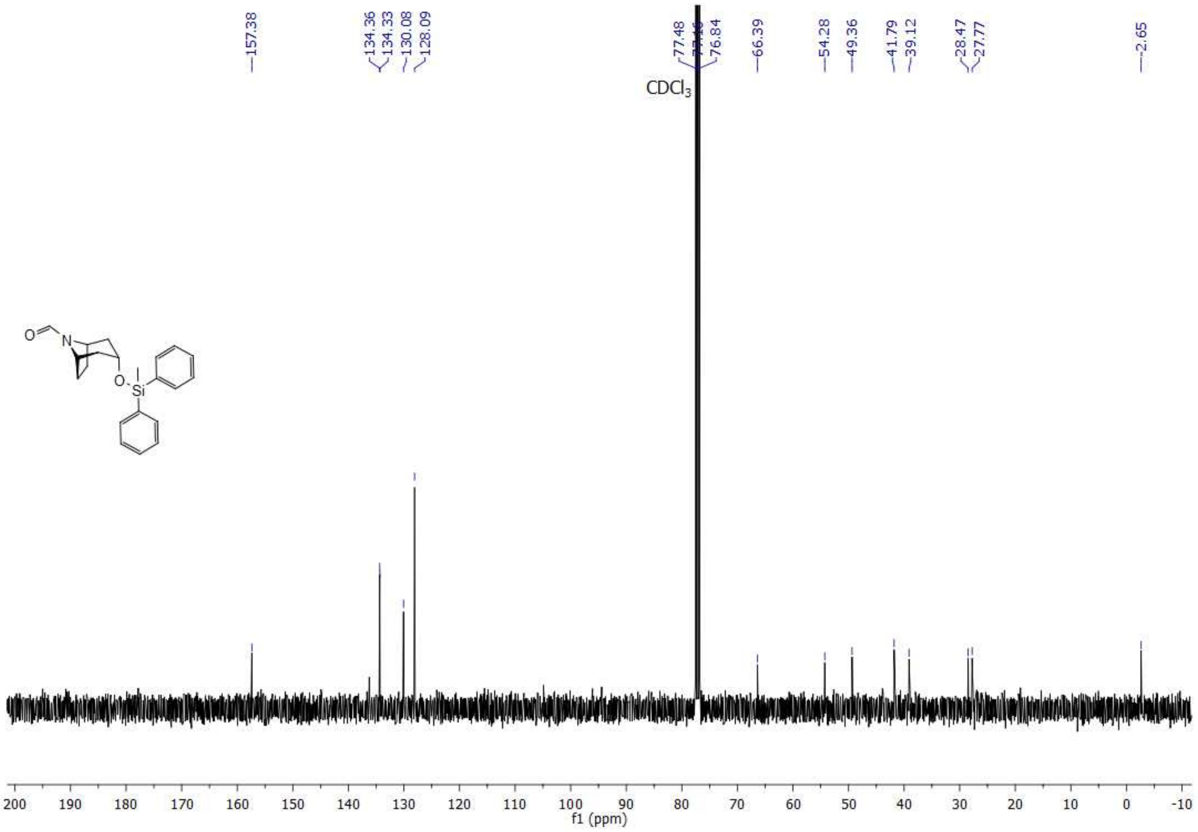

¹H NMR of (1R,3r,5S)-8-formyl-8-azabicyclo[3.2.1]octan-3-yl benzoate (2g)

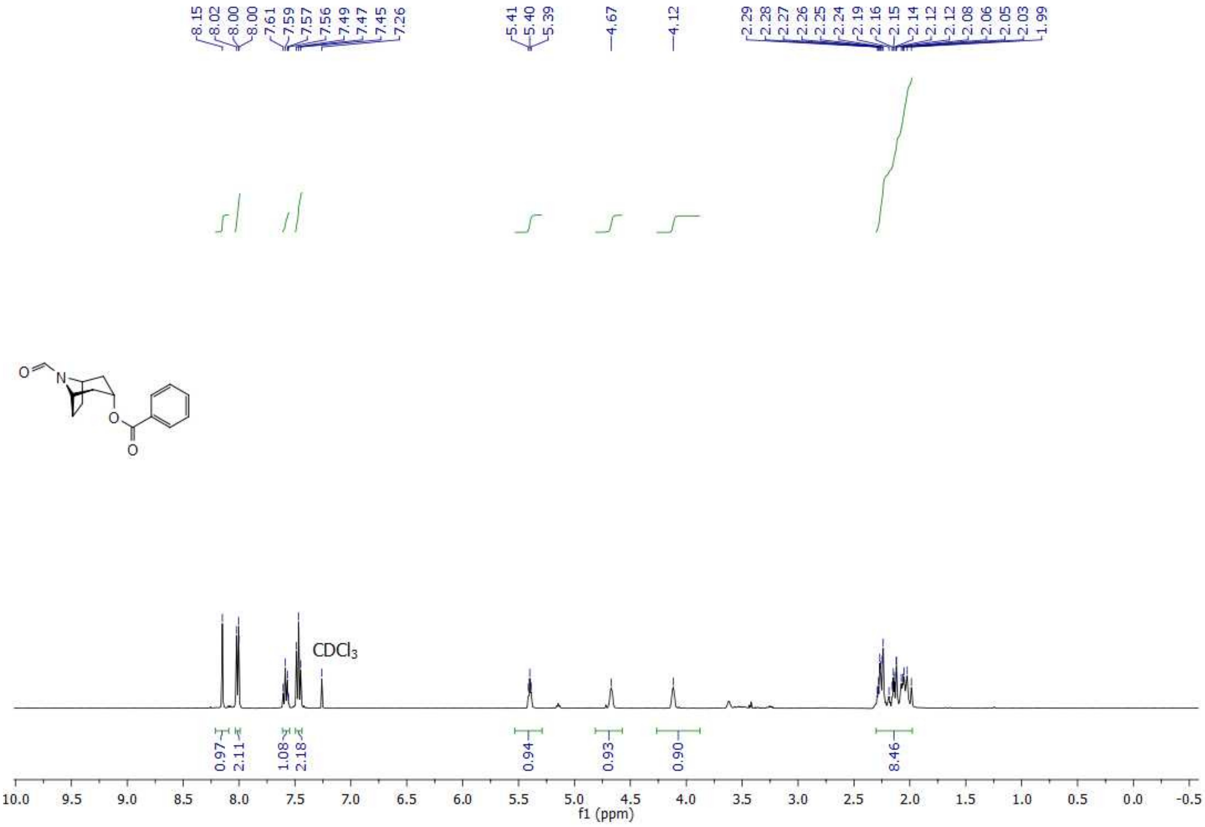

¹³C NMR of (1R,3r,5S)-8-formyl-8-azabicyclo[3.2.1]octan-3-yl benzoate (2g)

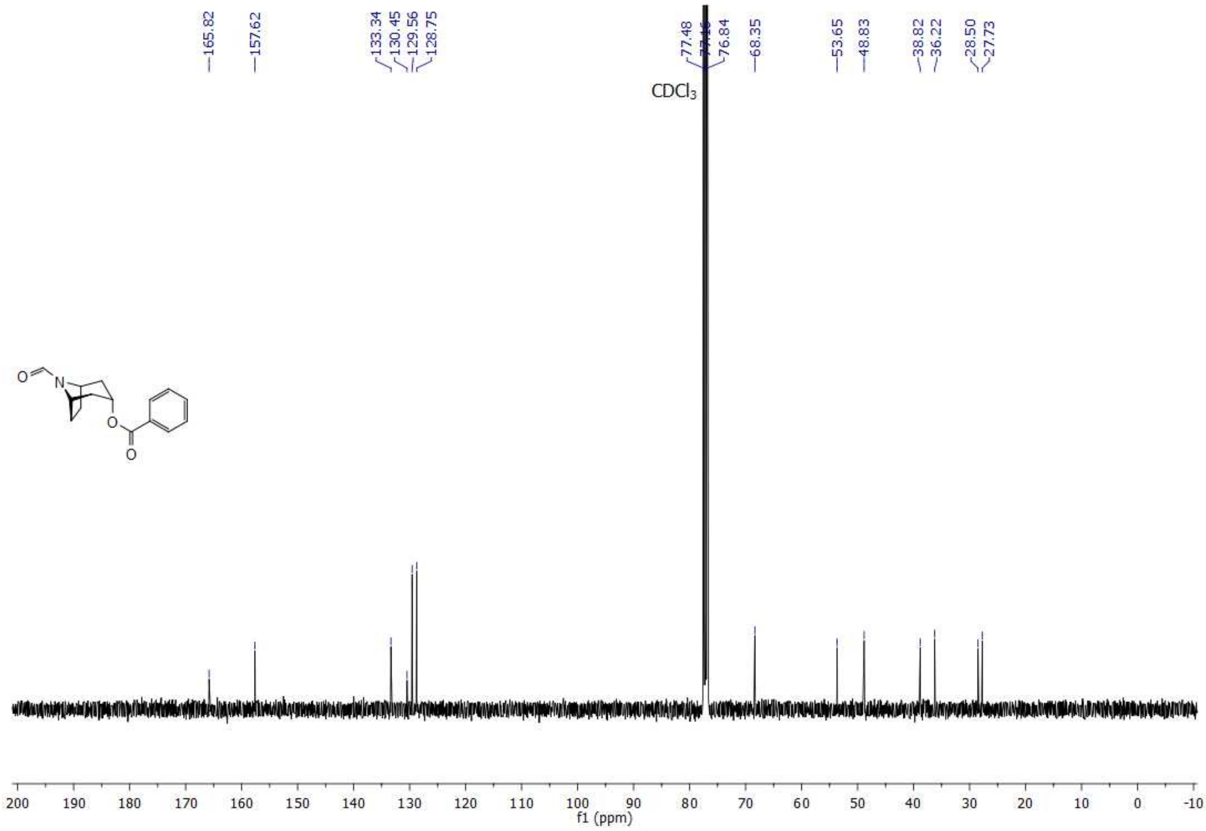

^{1}H NMR of (1R,3r,5S)-8-formyl-8-azabicyclo[3.2.1]octan-3-yl 4-(trifluoromethyl)benzoate (2h)

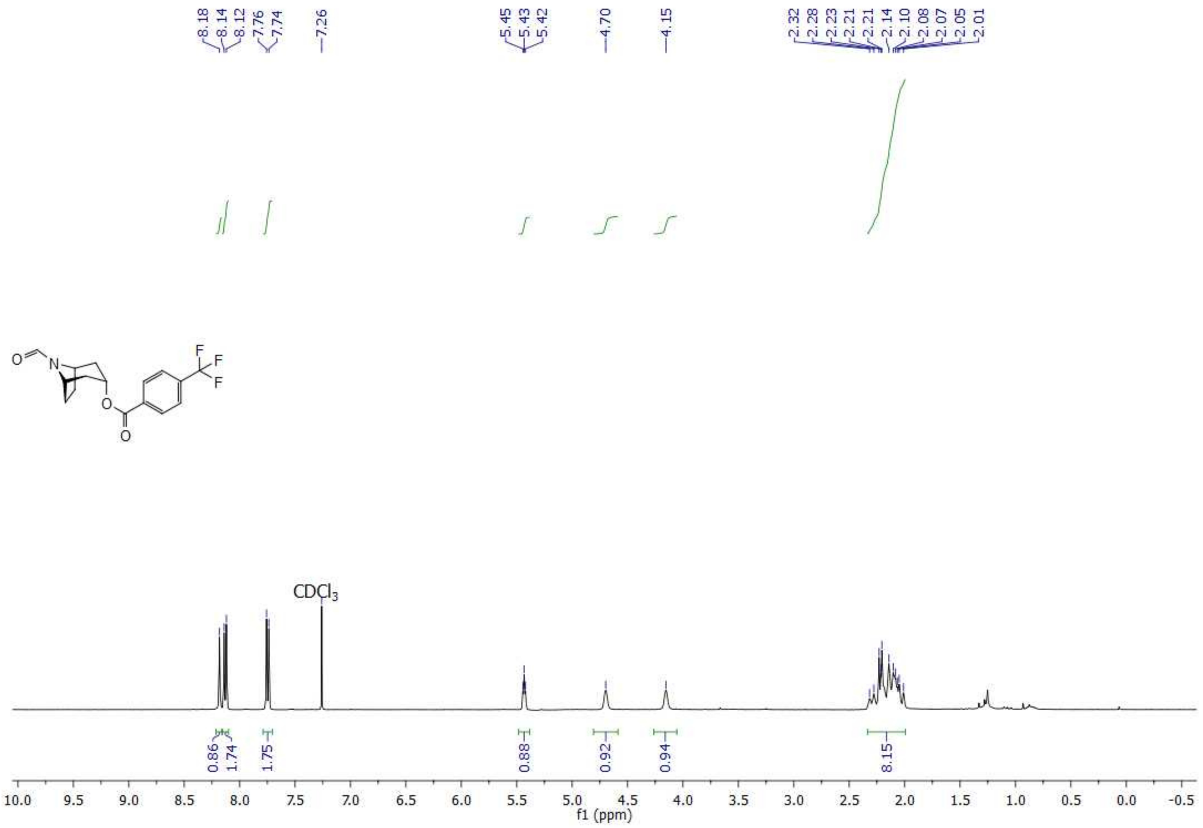

^{13}C NMR of (1R,3r,5S)-8-formyl-8-azabicyclo[3.2.1]octan-3-yl 4-(trifluoromethyl)benzoate (2h)

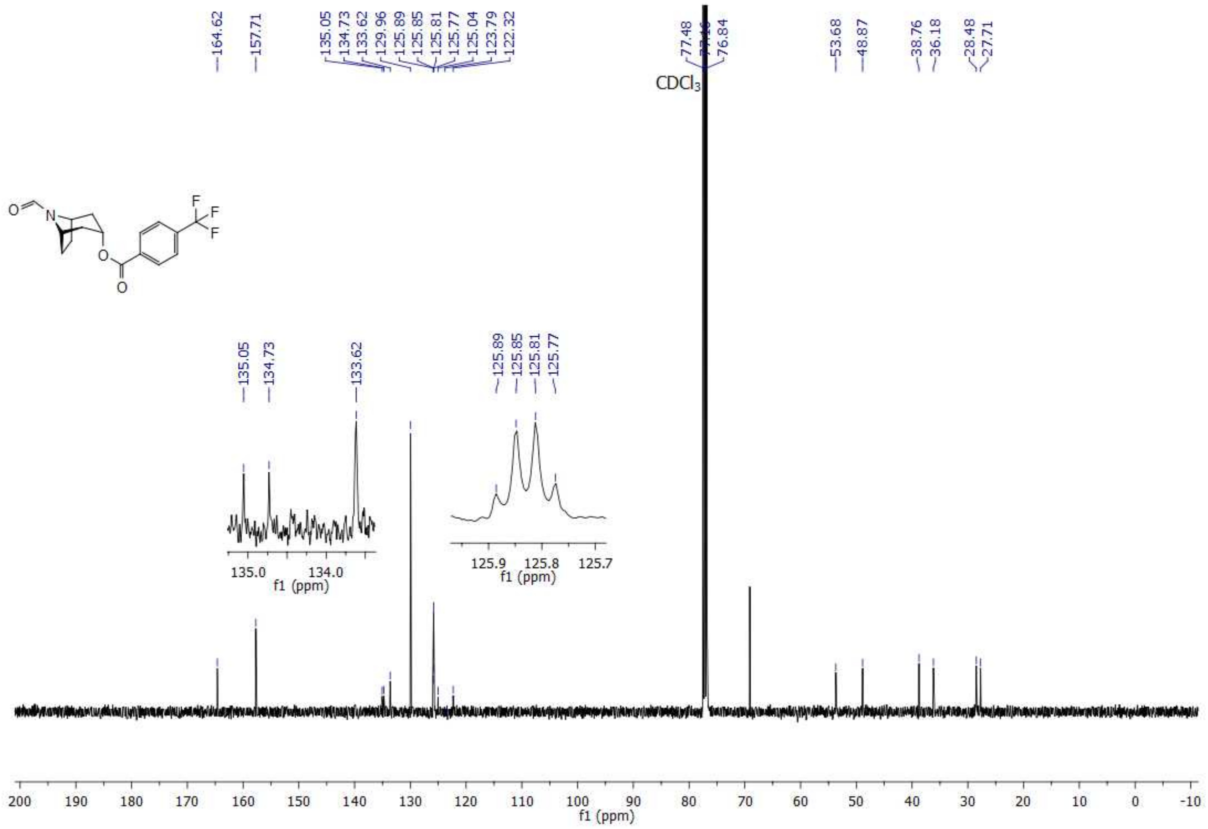

^{19}F NMR of 2h

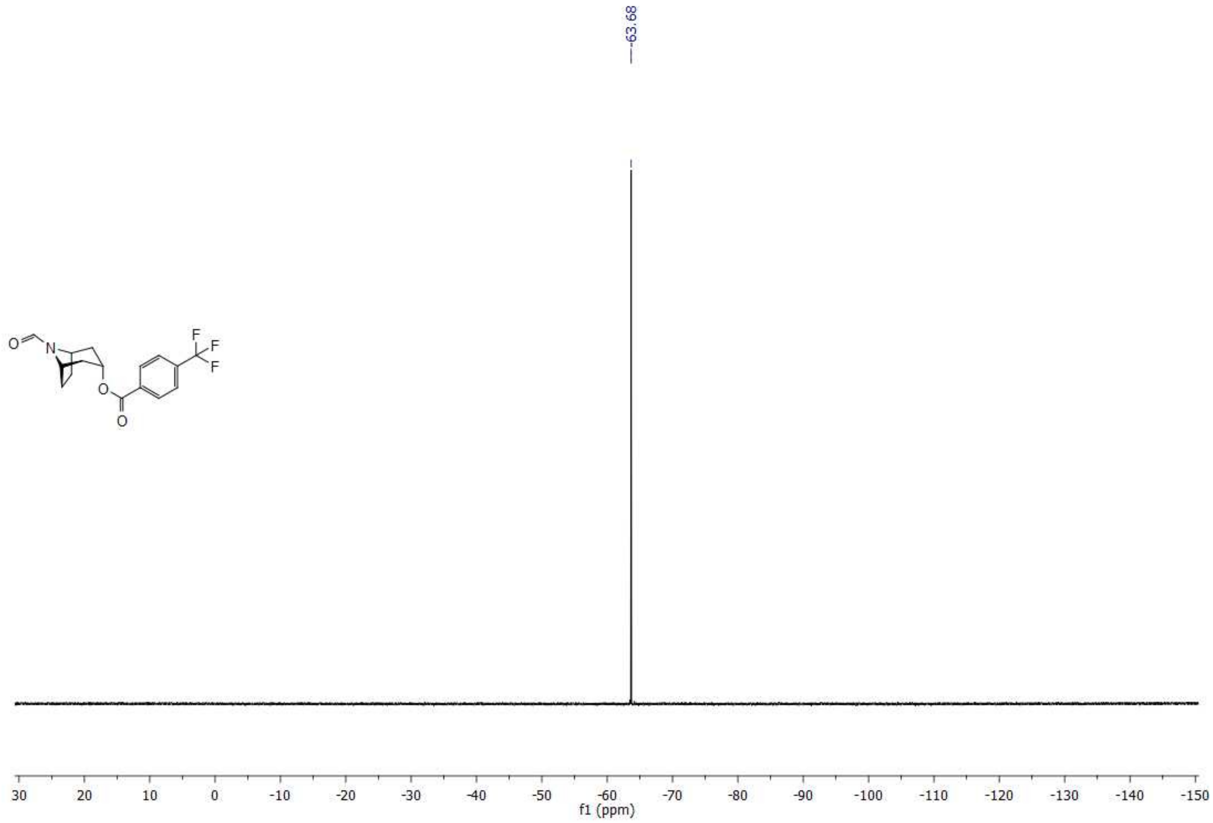

-63.68
30 20 10 0 -10 -20 -30 -40 -50 -60 -70 -80 -90 -100 -110 -120 -130 -140 -150
f1 (ppm)

^{1}H NMR of (1R,3r,5S)-8-formyl-8-azabicyclo[3.2.1]octan-3-yl 3,4-dimethoxybenzoate (2i)

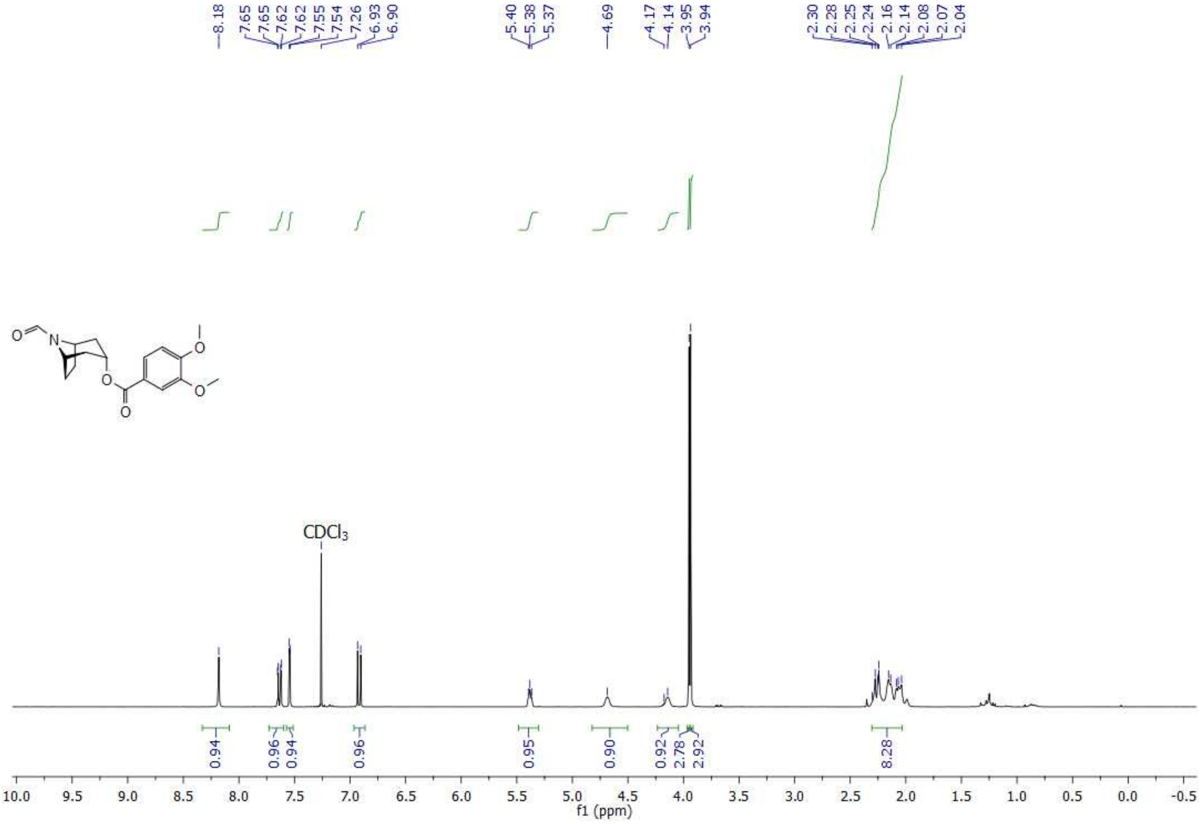

^{13}C NMR of (1R,3r,5S)-8-formyl-8-azabicyclo[3.2.1]octan-3-yl 3,4-dimethoxybenzoate (2i)

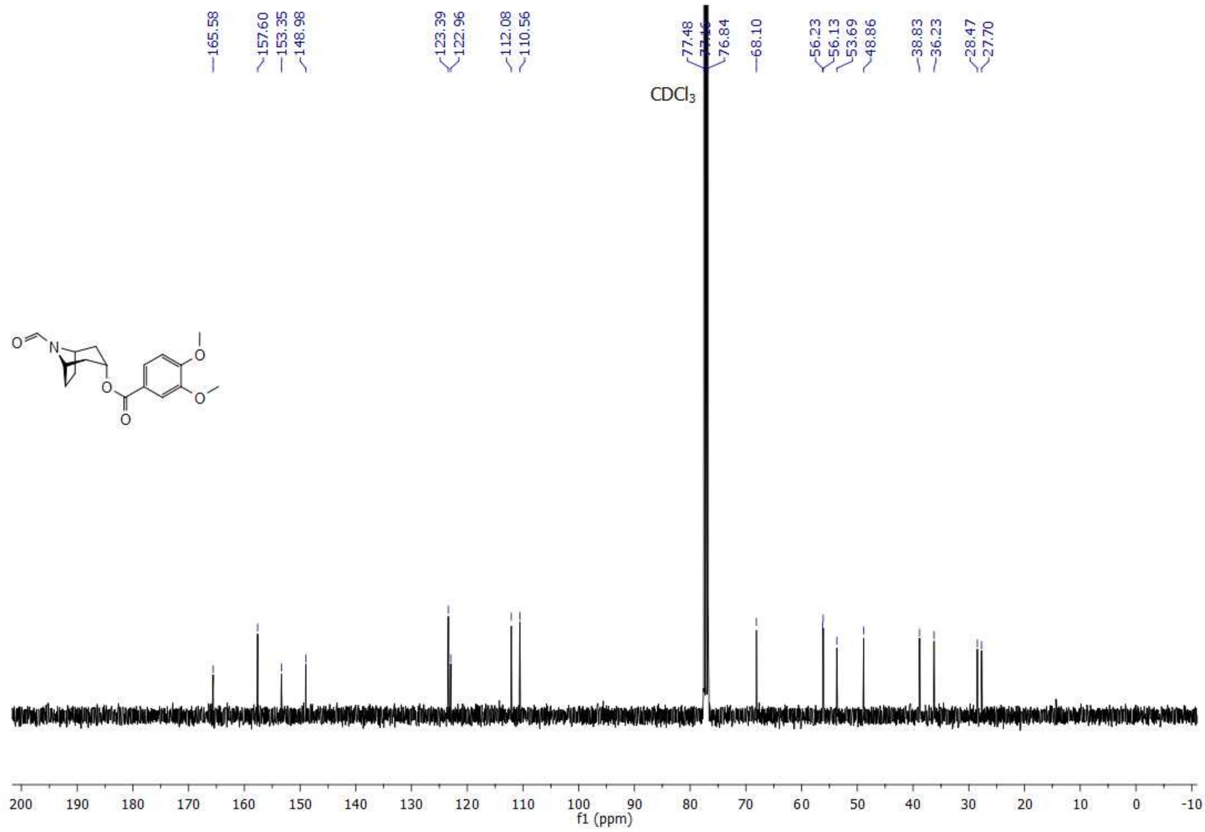

^{1}H NMR of 4-(phenylsulfonyl)piperazine-1-carbaldehyde (2j)

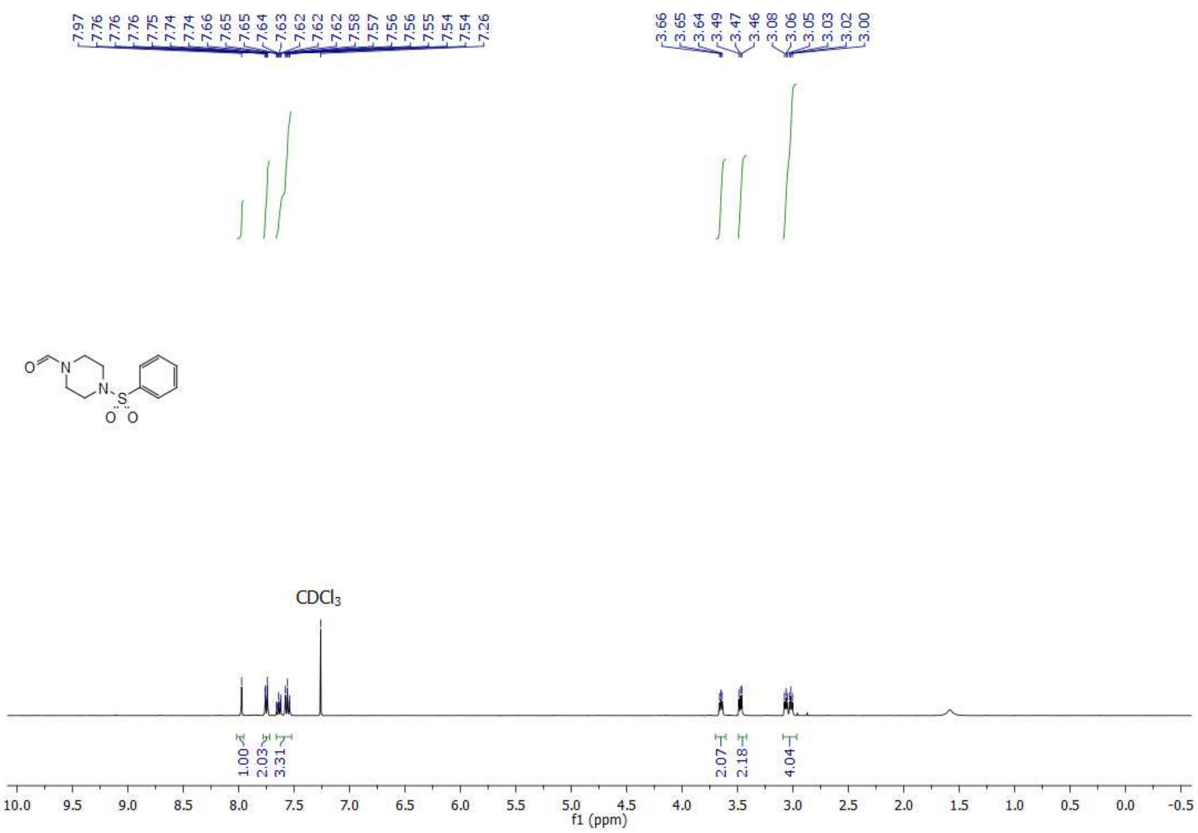

^{13}C NMR of 4-(phenylsulfonyl)piperazine-1-carbaldehyde (2j)

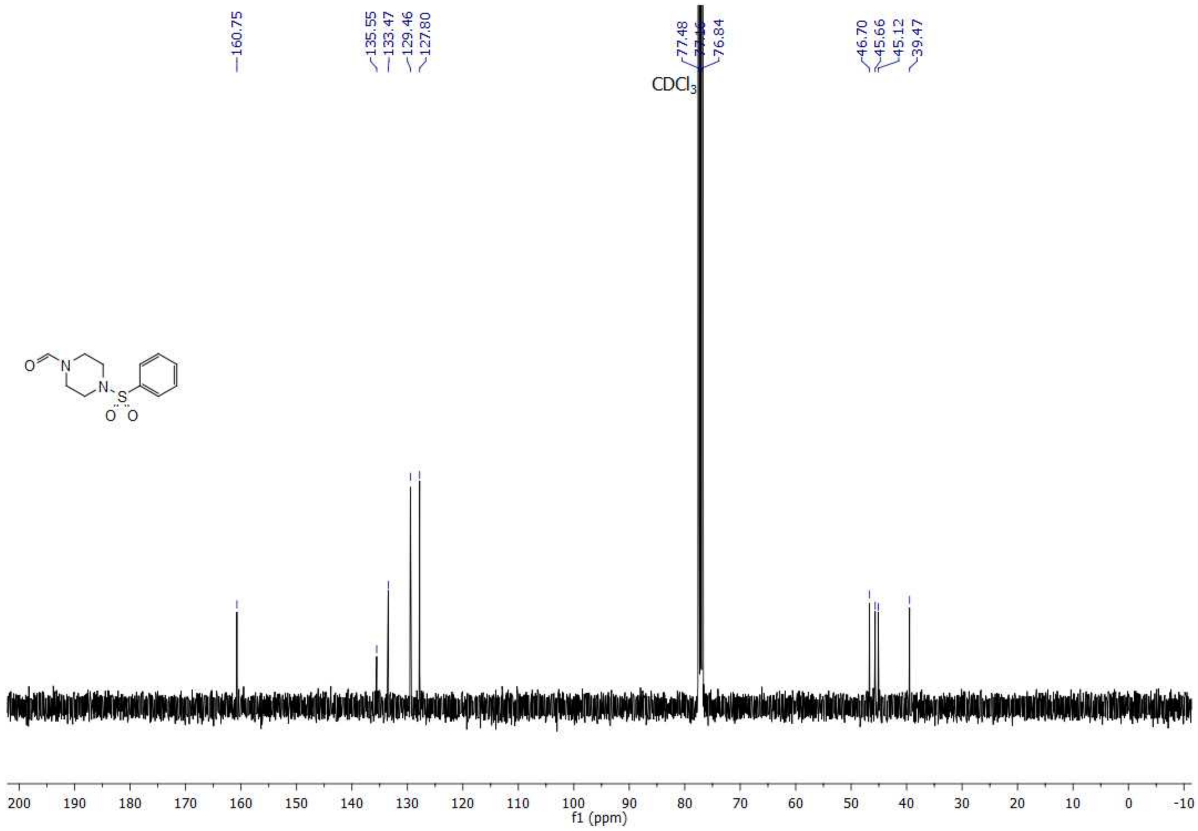

^{1}H NMR of 4-tosylpiperazine-1-carbaldehyde (2k)

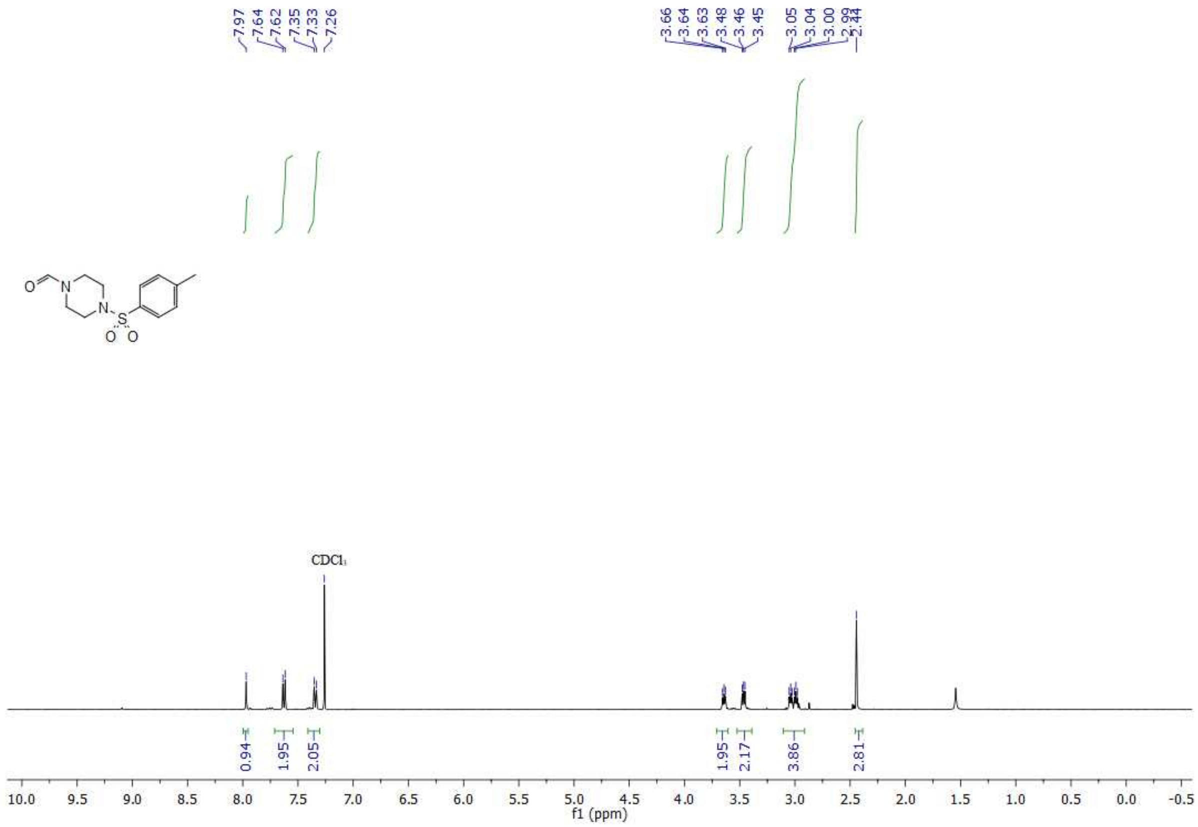

^{13}C NMR of 4-tosylpiperazine-1-carbaldehyde (2k)

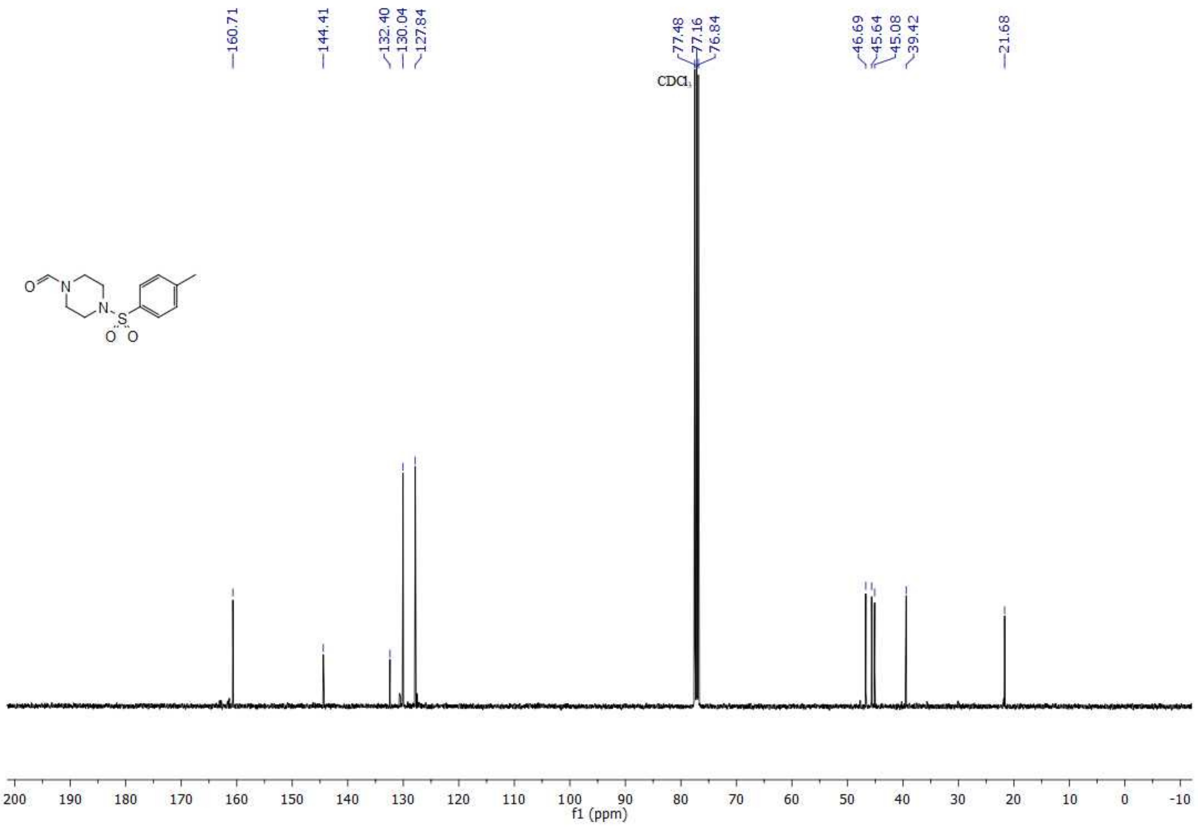

^{1}H NMR 4-((4-(trifluoromethyl)phenyl)sulfonyl)piperazine-1-carbaldehyde (2l)

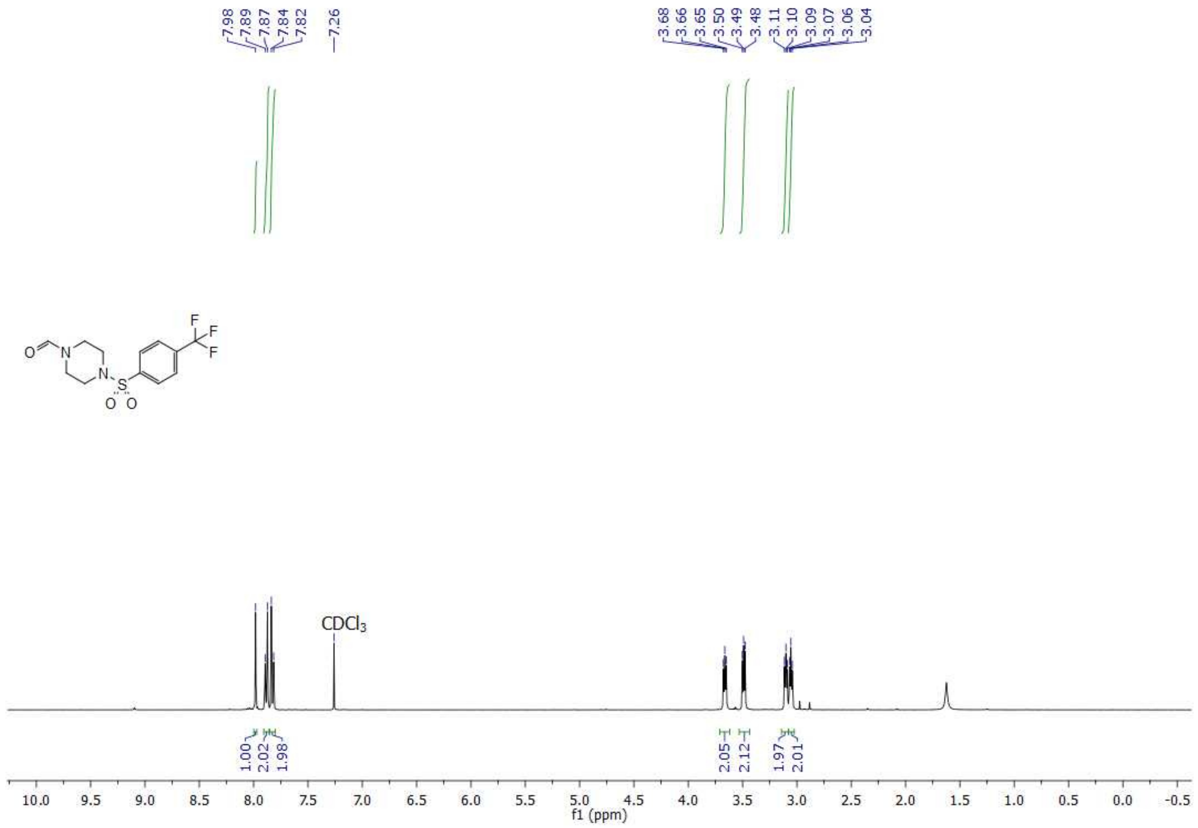

^{13}C NMR of 4-((4-(trifluoromethyl)phenyl)sulfonyl)piperazine-1-carbaldehyde (2l)

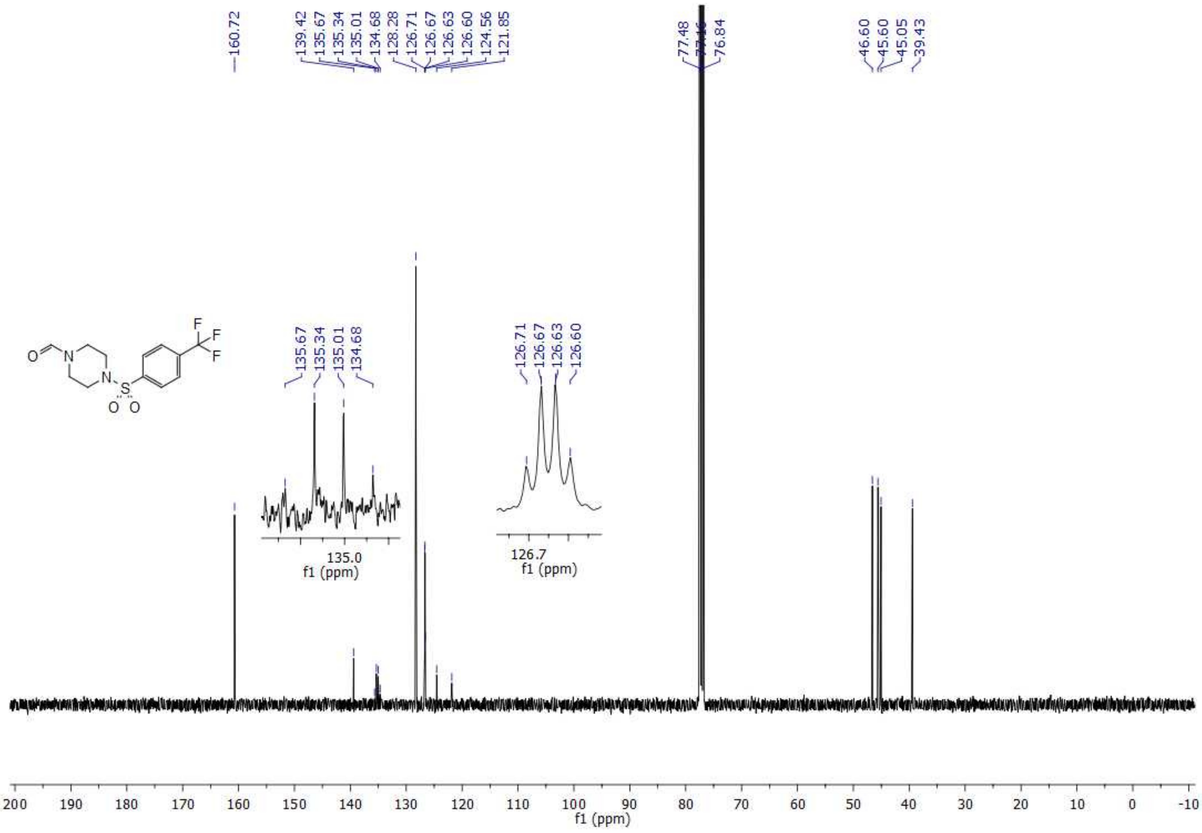

^{19}F NMR of 4-((4-(trifluoromethyl)phenyl)sulfonyl)piperazine-1-carbaldehyde (2l)

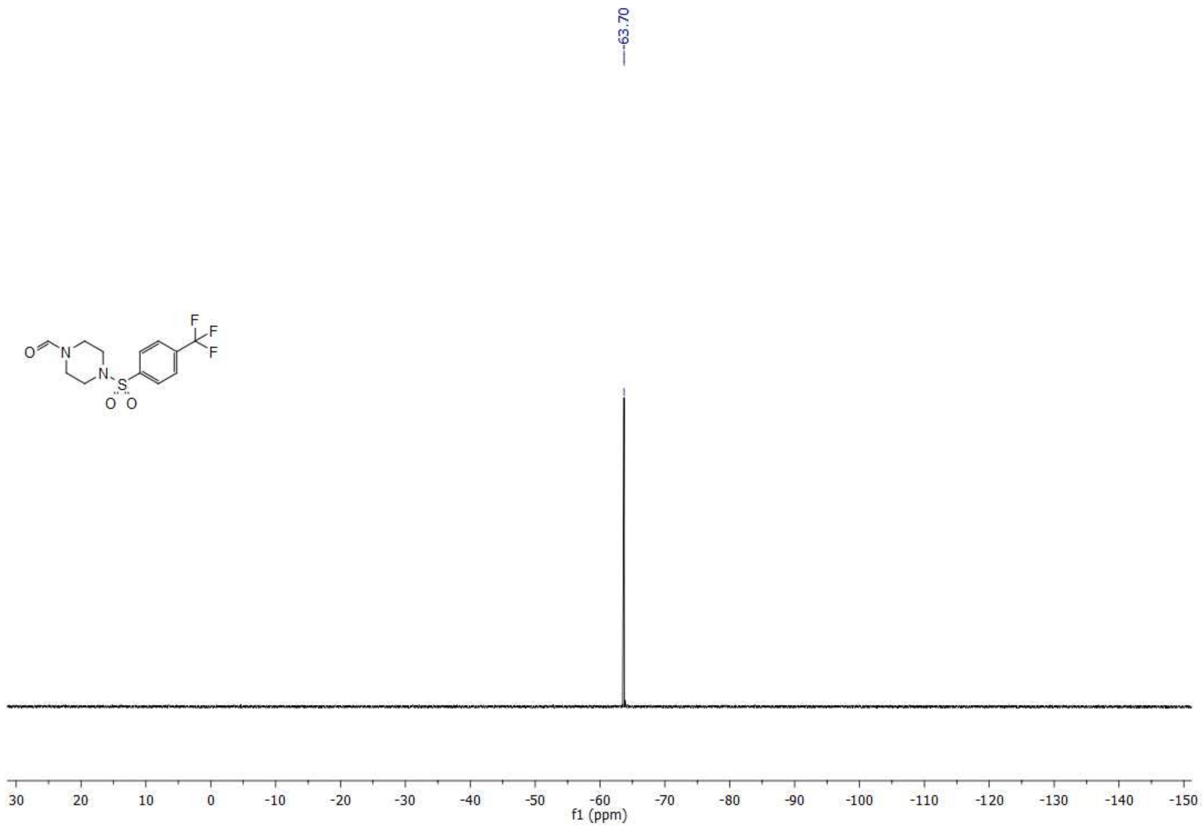

^{1}H NMR of 4-((4-bromophenyl)sulfonyl)piperazine-1-carbaldehyde (2m)

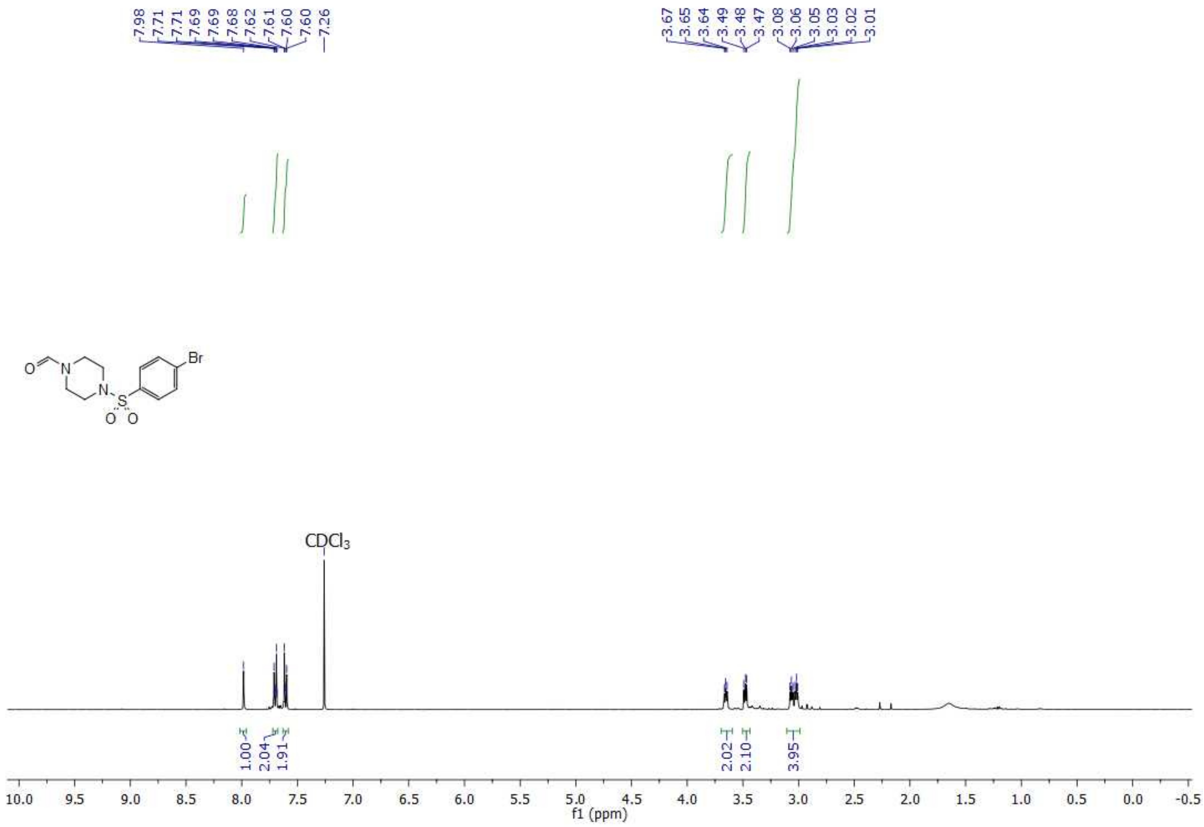

^{13}C NMR of 4-((4-bromophenyl)sulfonyl)piperazine-1-carbaldehyde (2m)

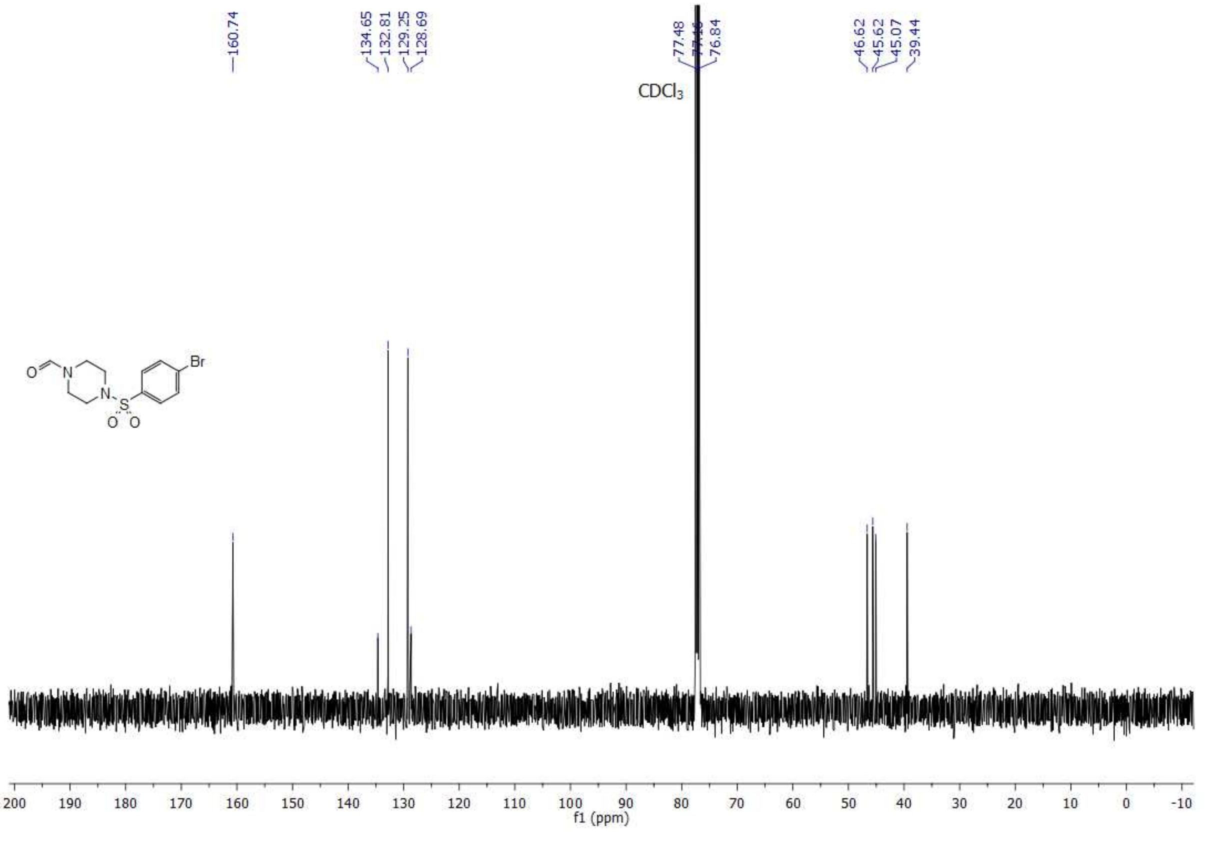

^{1}H NMR of (1S,4S)-5-((4-(trifluoromethyl)phenyl)sulfonyl)-2,5-diazabicyclo[2.2.1]heptane-2-carbaldehyde (2n)

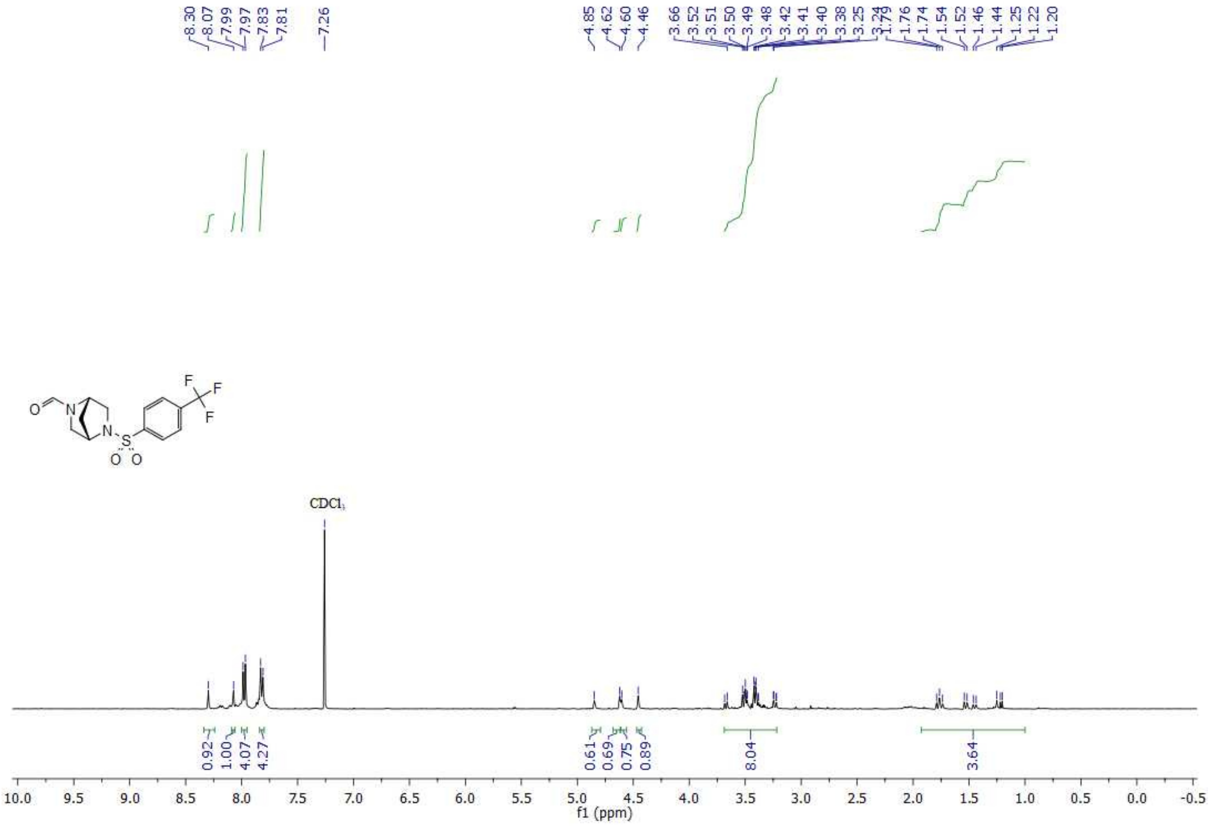

^{13}C NMR of of (1S,4S)-5-((4-(trifluoromethyl)phenyl)sulfonyl)-2,5-diazabicyclo[2.2.1]heptane-2-carbaldehyde (2n)

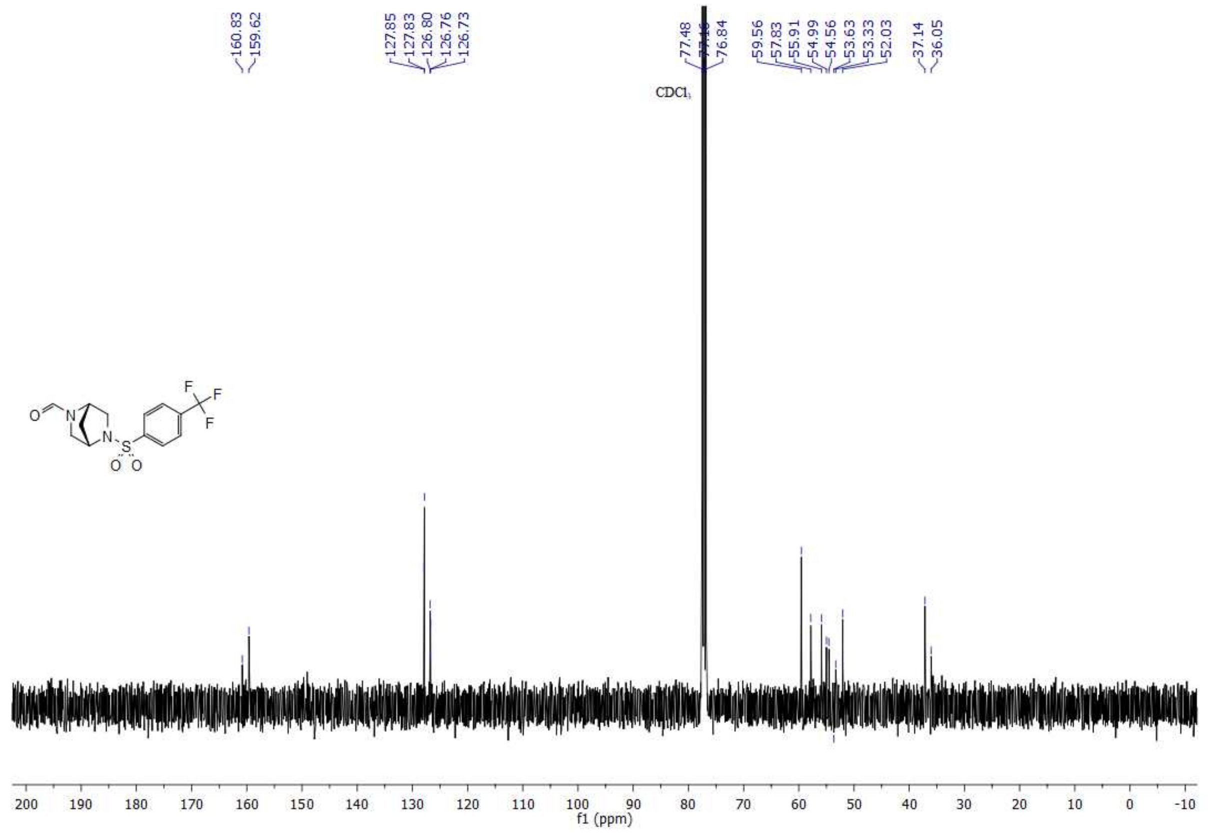

^{19}F NMR of of (1S,4S)-5-((4-(trifluoromethyl)phenyl)sulfonyl)-2,5-diazabicyclo[2.2.1]heptane-2-carbaldehyde (2n)

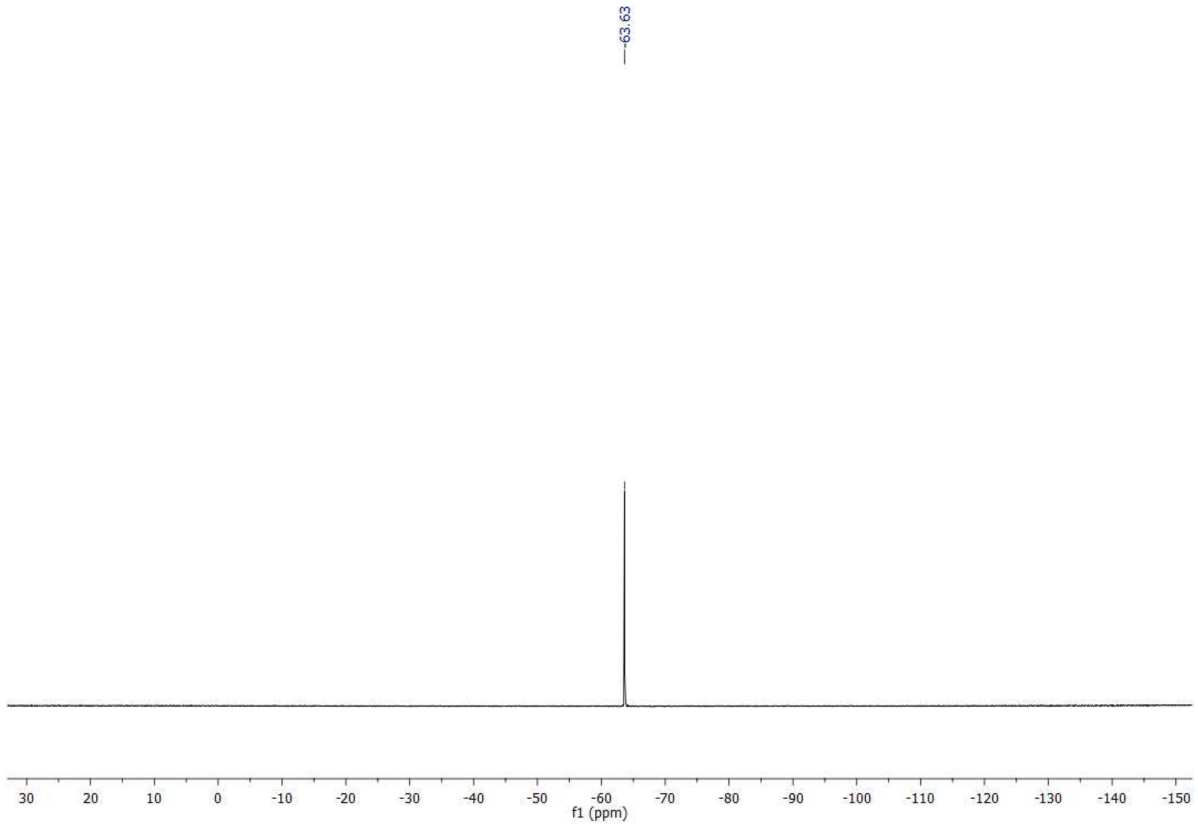

^{1}H NMR of 4-benzoylpiperazine-1-carbaldehyde (2o)

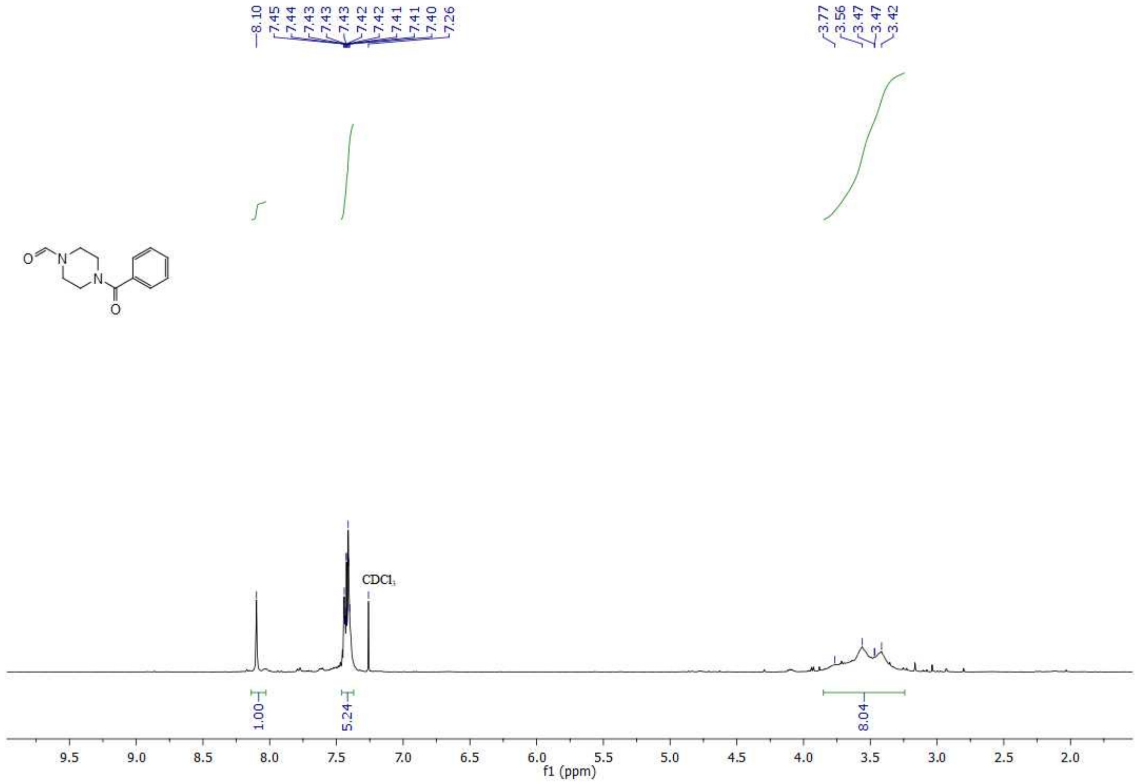

*The presence of rotamers at each and of the piperazine leads to multiple rotamers and line-broadening in the ^{1}H NMR. Purity was confirmed by inspection of the ^{13}C NMR spectrum (below).

^{13}C NMR of 4-benzoylpiperazine-1-carbaldehyde (2o)

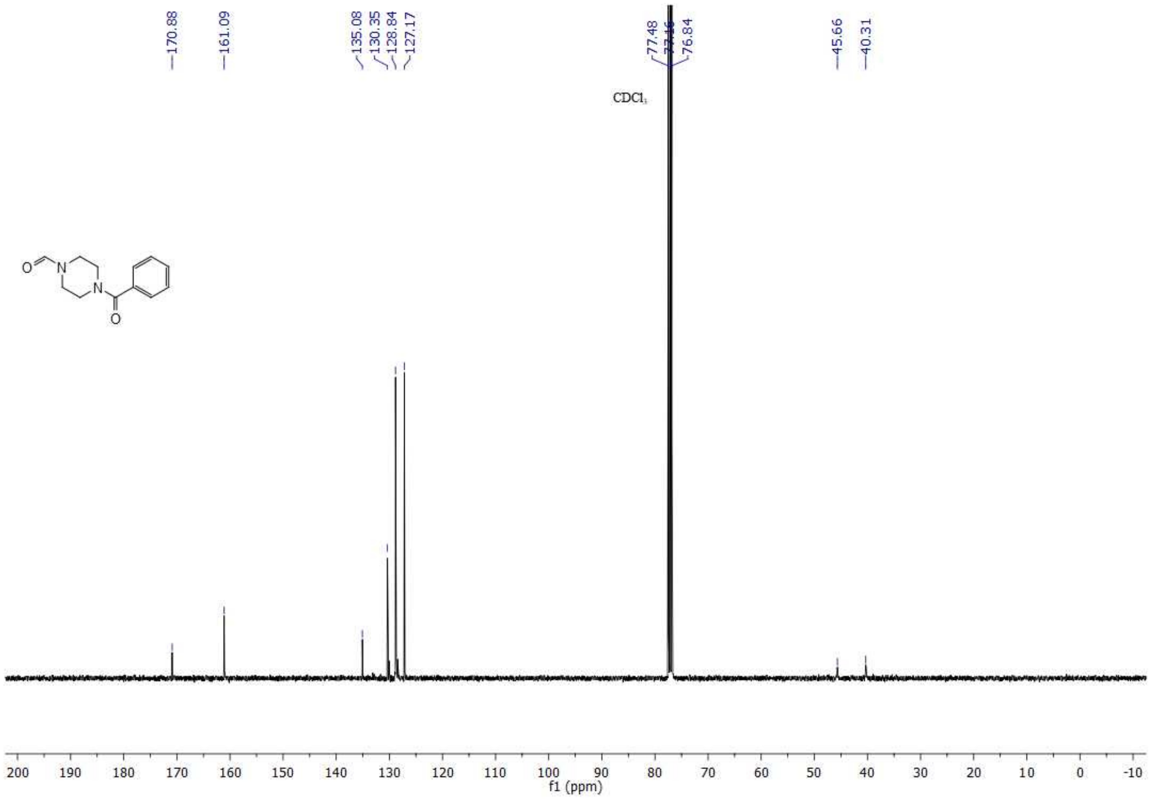

^{1}H NMR of 2o 4-benzoylpiperazine-1-carbaldehyde (2p)

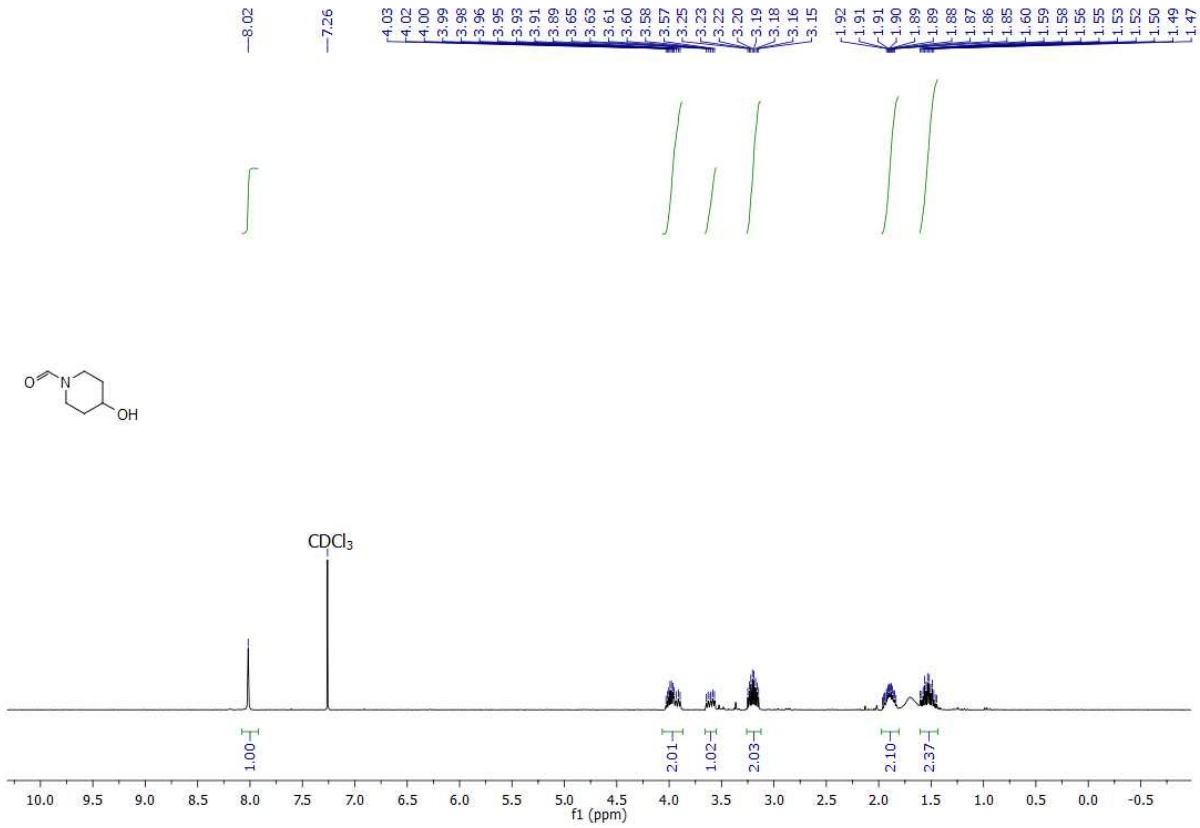

^{13}C NMR 4-benzoylpiperazine-1-carbaldehyde (2p)

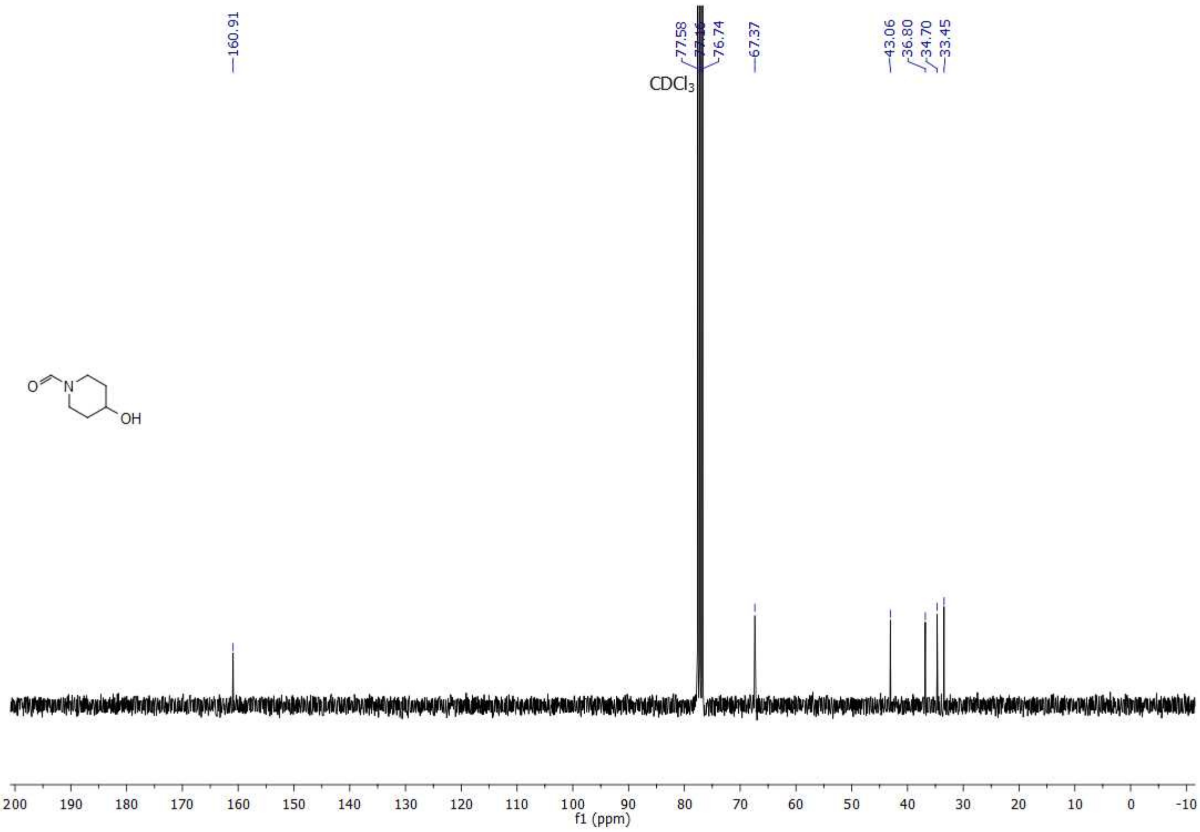

^{1}H NMR of De(*N*-methyl)-*N*-formyl erythromycin A (2r)*

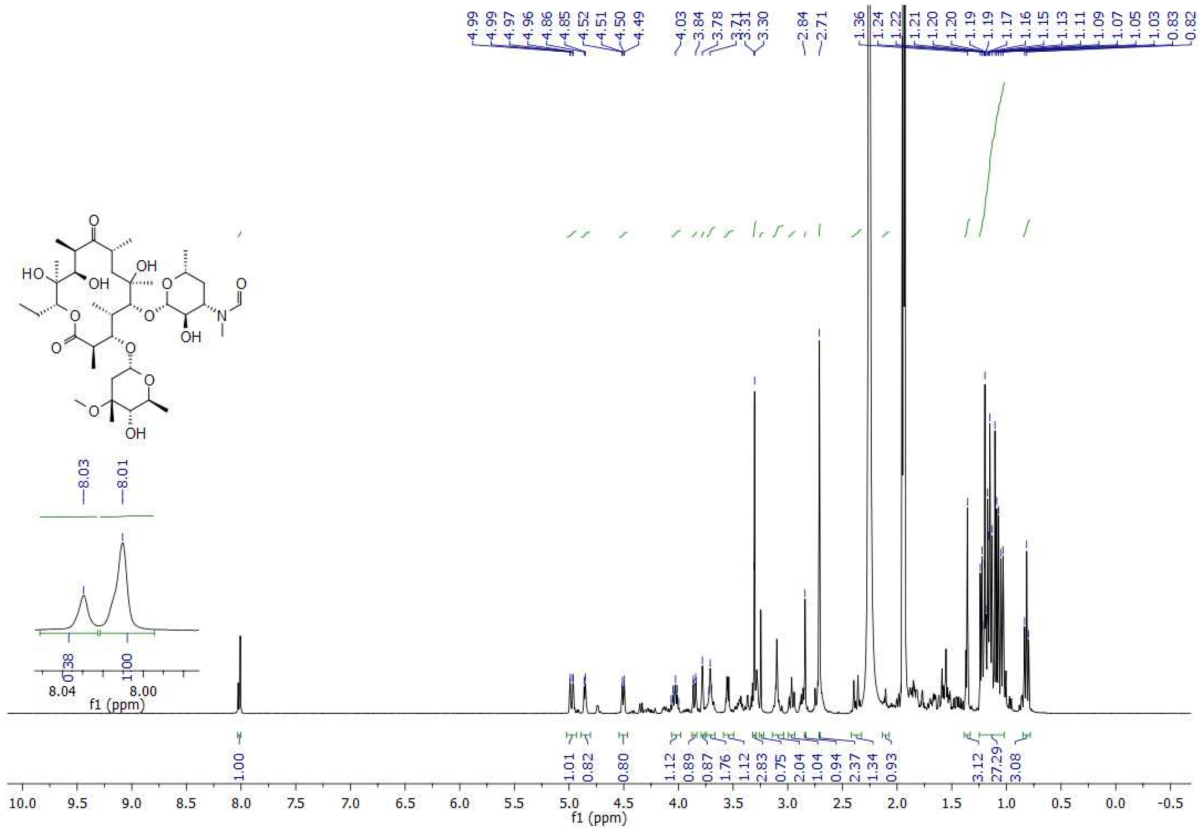

^{13}C NMR of De(*N*-methyl)-*N*-formyl erythromycin A (2r)

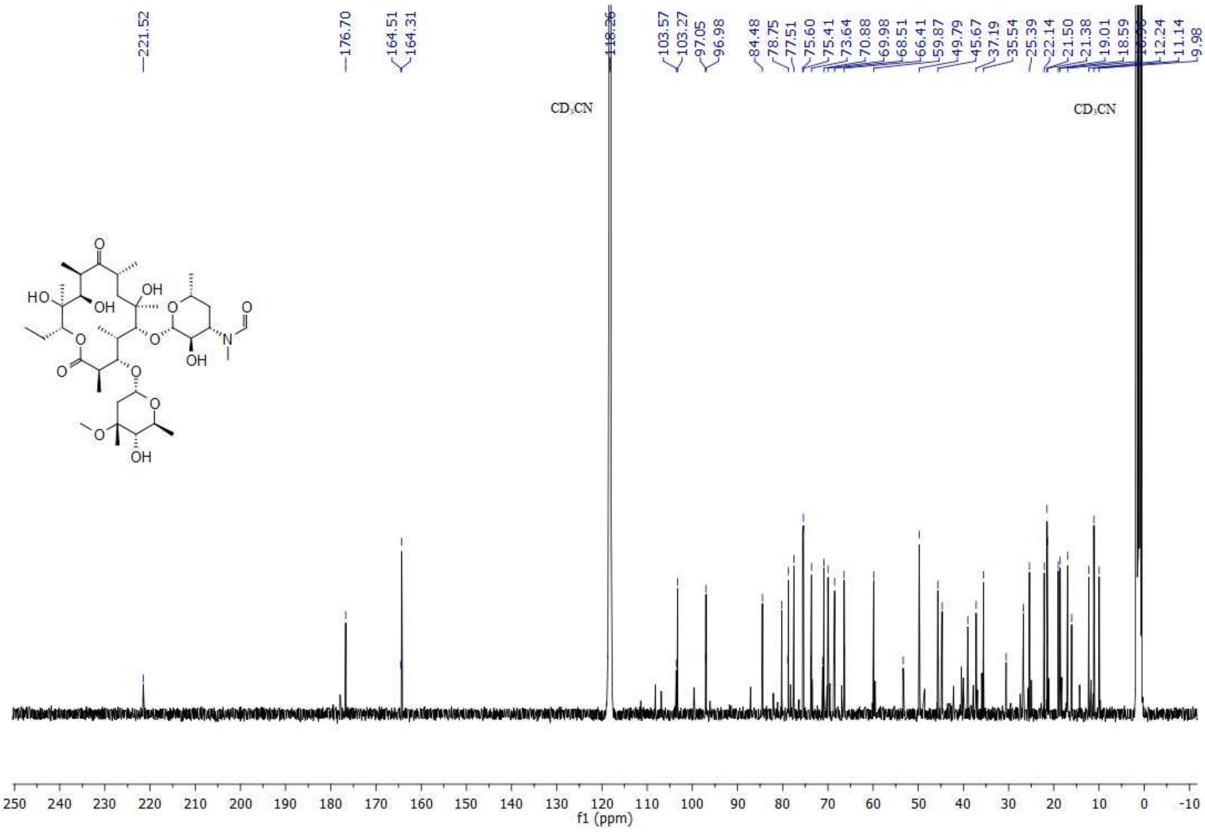

*We note that commercially-supplied mycins are mixtures of diastereomers. The ^{1}H NMR spectra of products reflect the purity of diastereomers from the starting materials. Only key peaks are integrated and highlighted, and a spectrum of erythromycin starting material is given below for comparison.

^{1}H NMR of commercial erythromycin (1r)

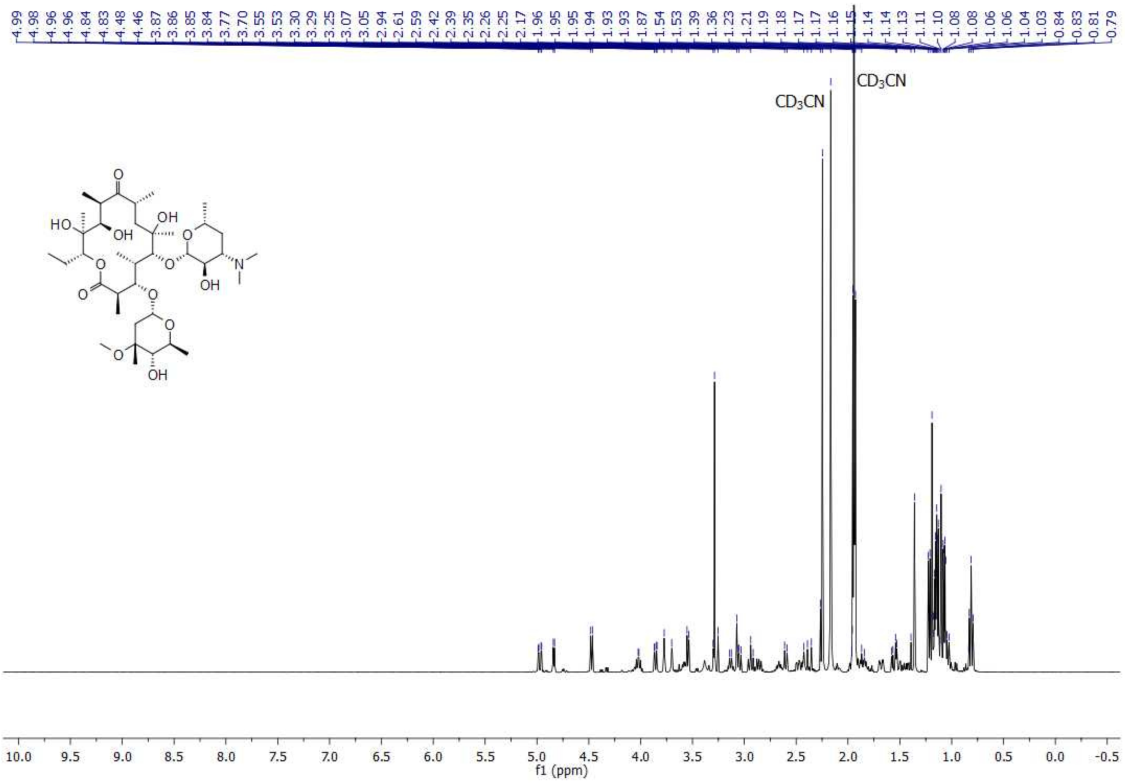

^{1}H NMR of commercial Clarithromycin (1s)

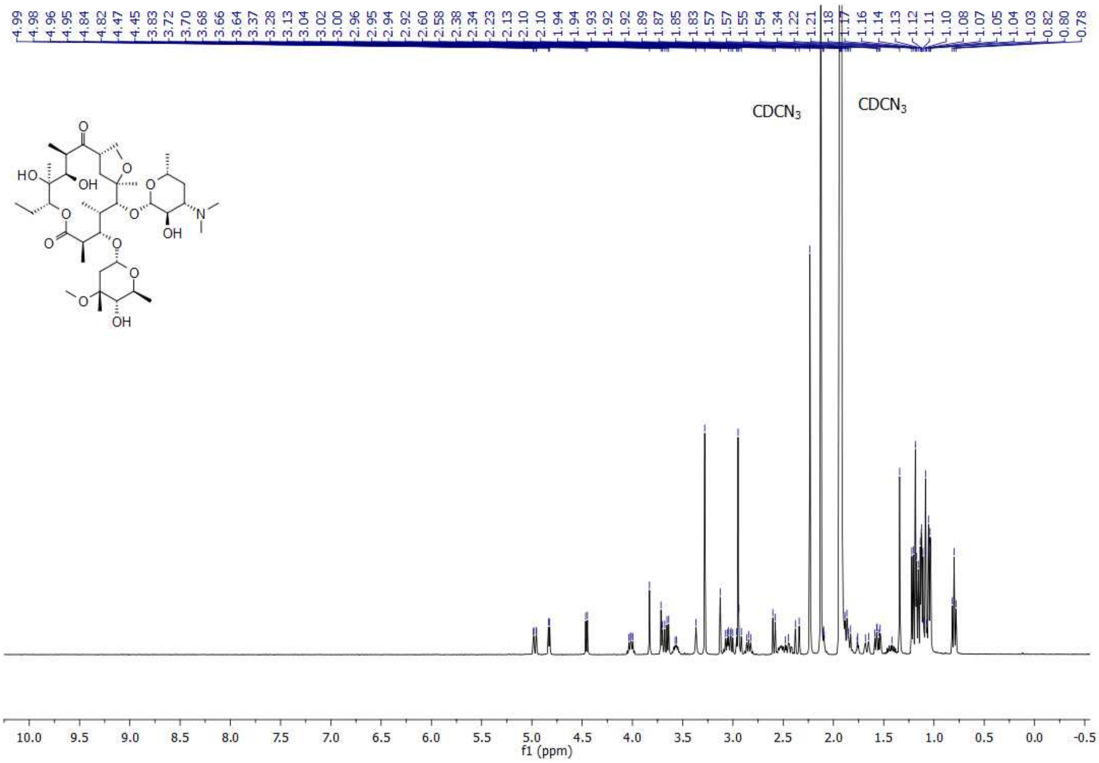

^{1}H NMR of commercial Roxithromycin (1t)

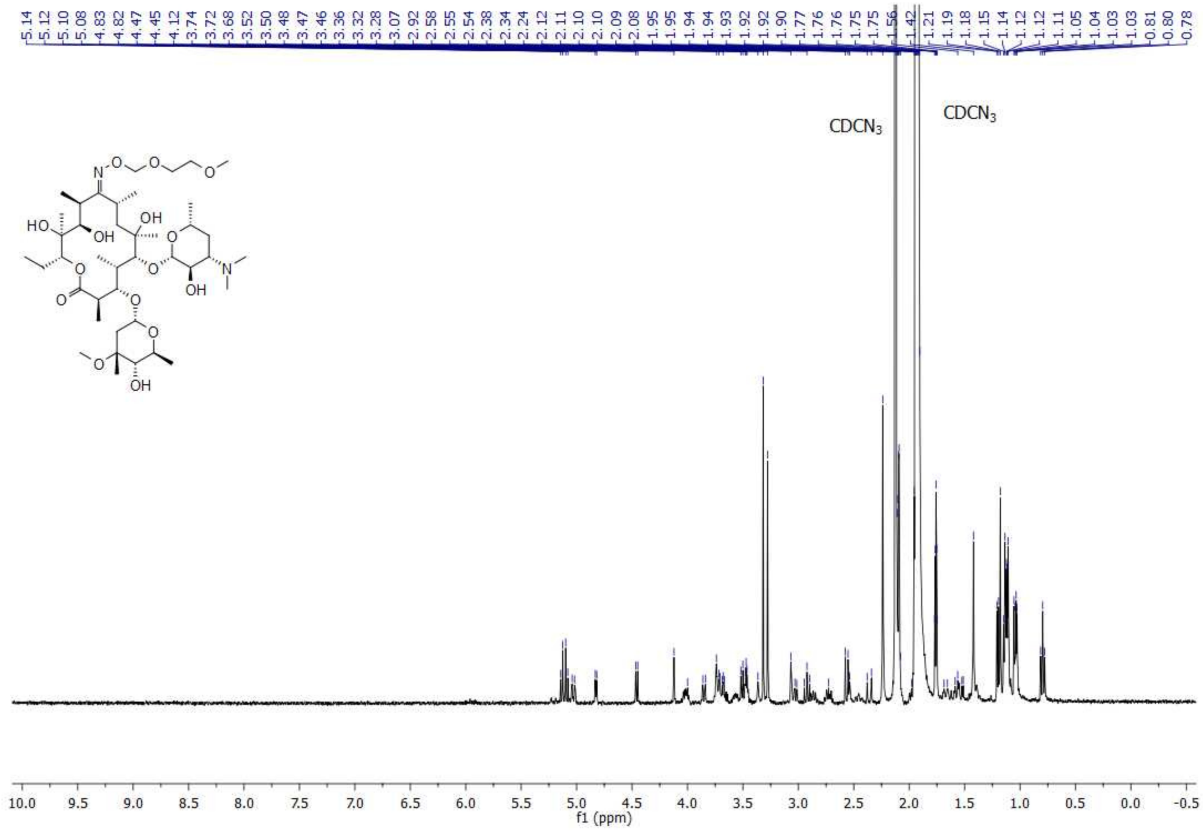

^{1}H NMR of De(*N*-methyl)-*N*-formyl clarithromycin (2s)

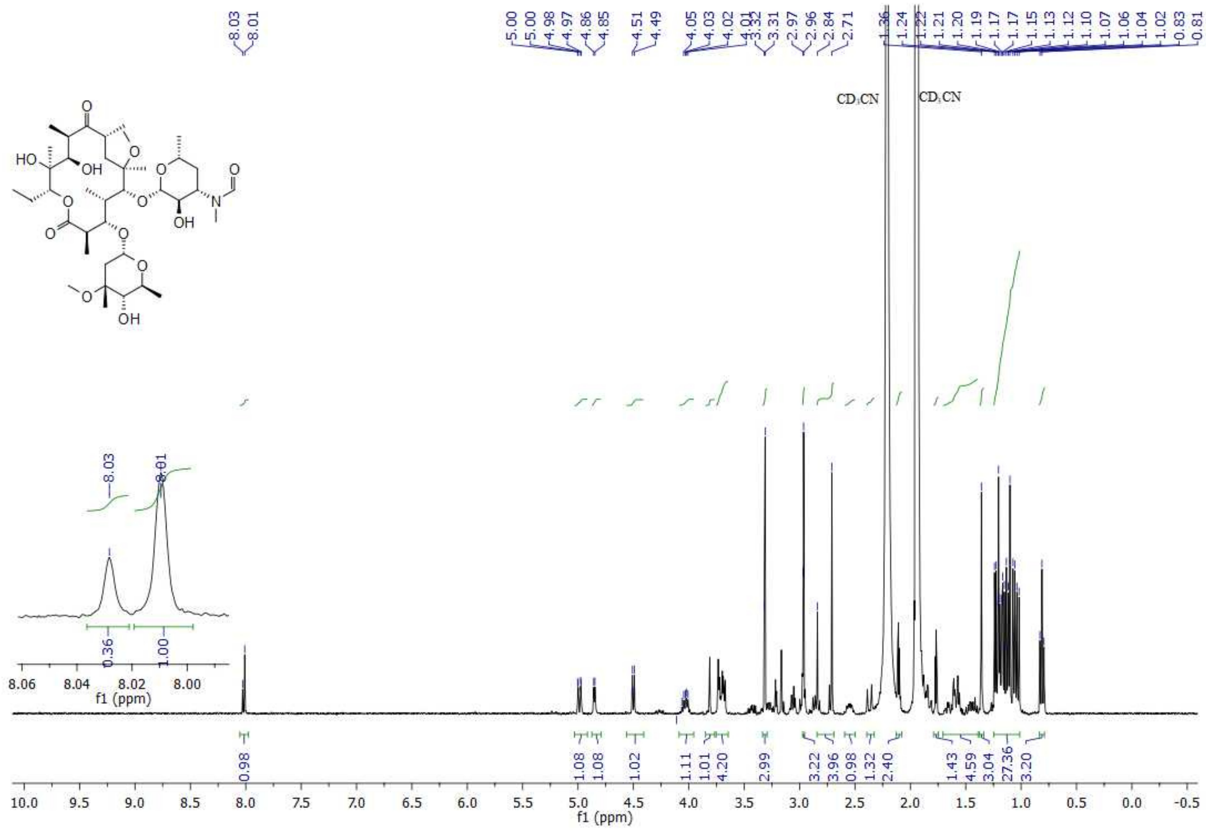

^{13}C NMR of De(*N*-methyl)-*N*-formyl clarithromycin (2s)

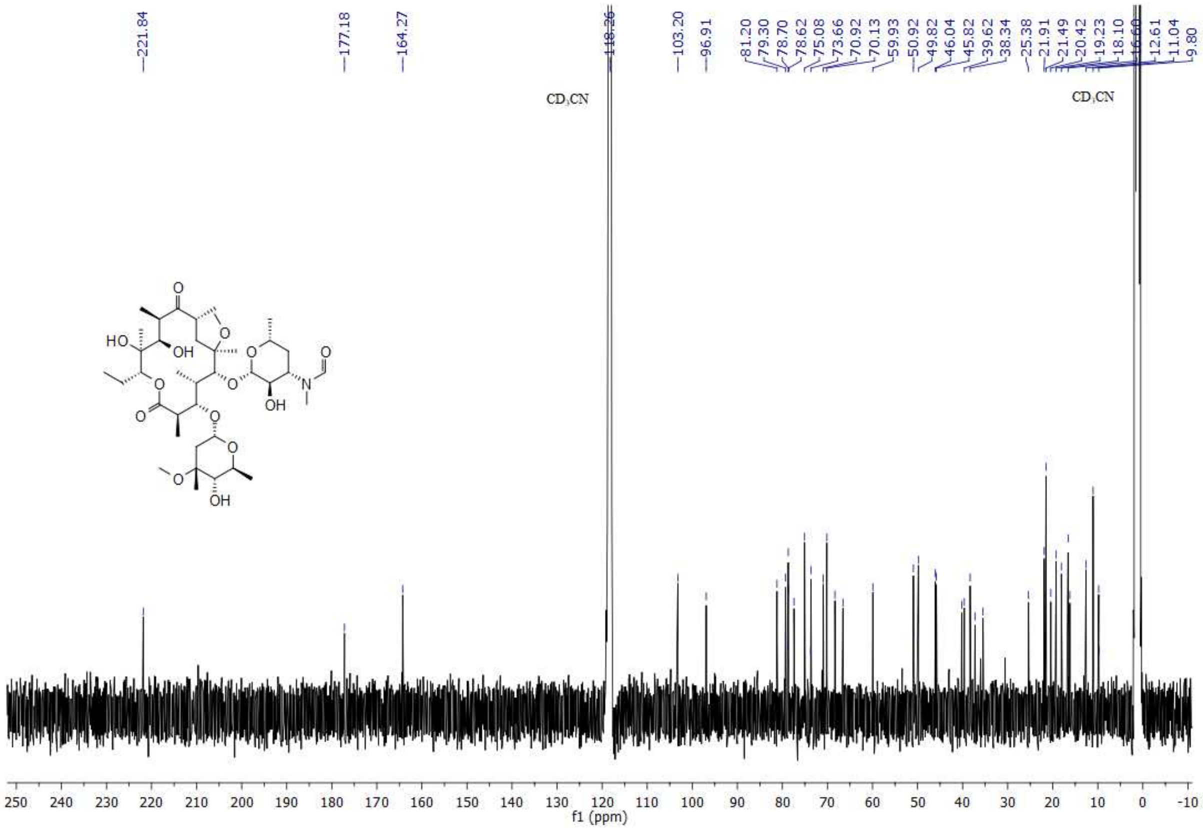

^{1}H NMR of De(*N*-methyl)-*N*-formyl roxithromycin, 2t

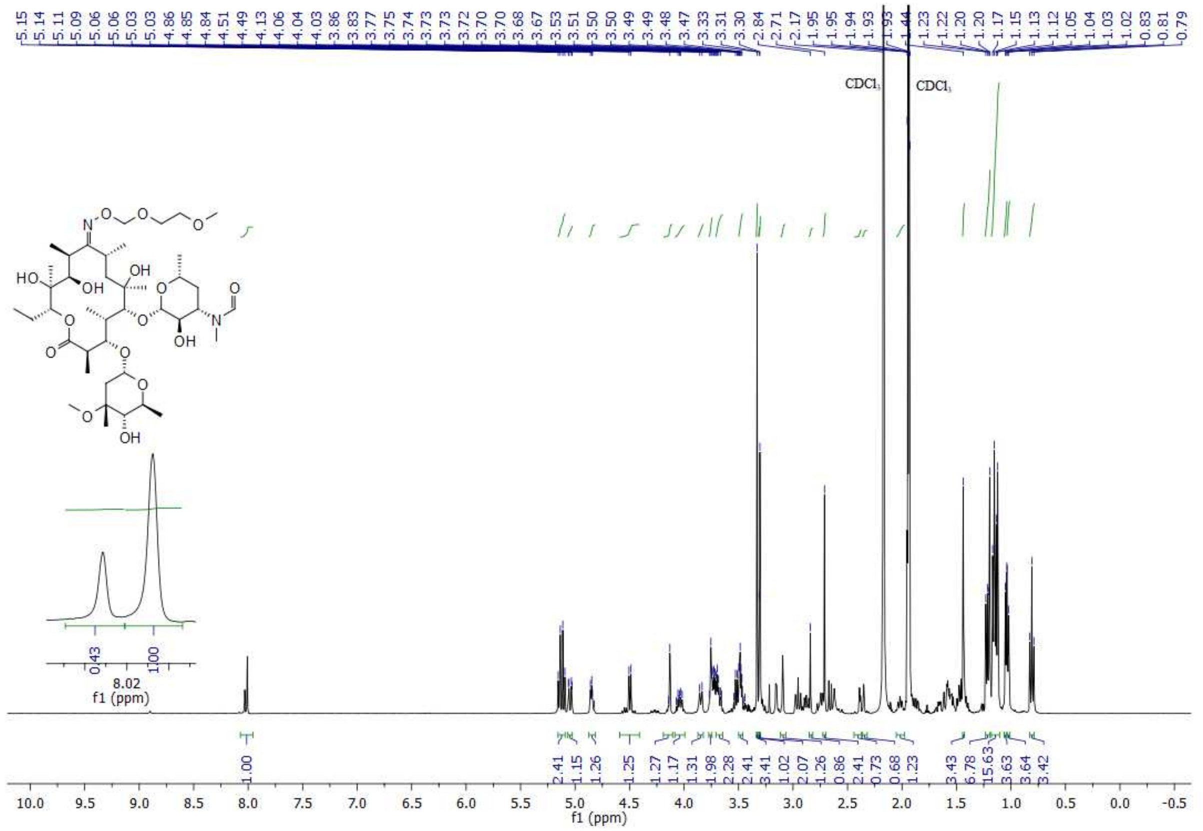

^{13}C NMR of De(*N*-methyl)-*N*-formyl roxithromycin, 2t

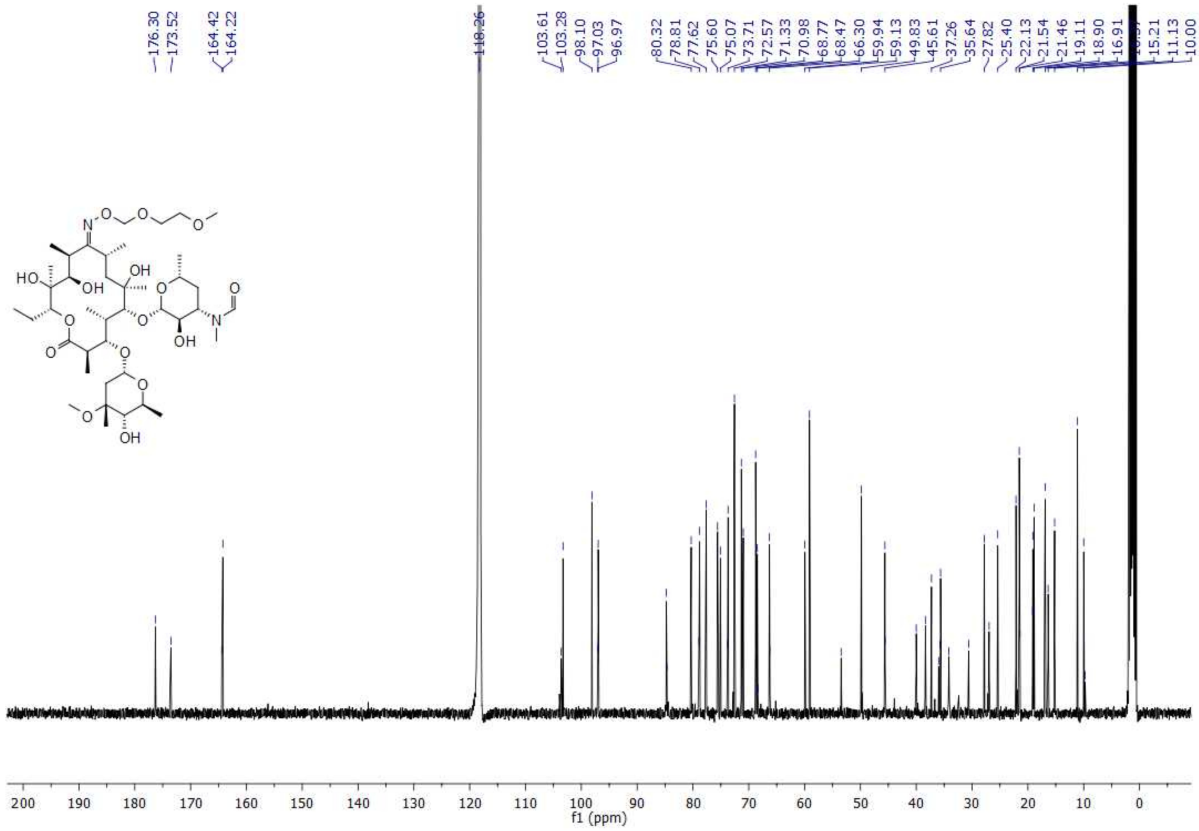

Crude ^{1}H NMR Spectra for Singlet Oxygen trapping (formation of Ascaridole)

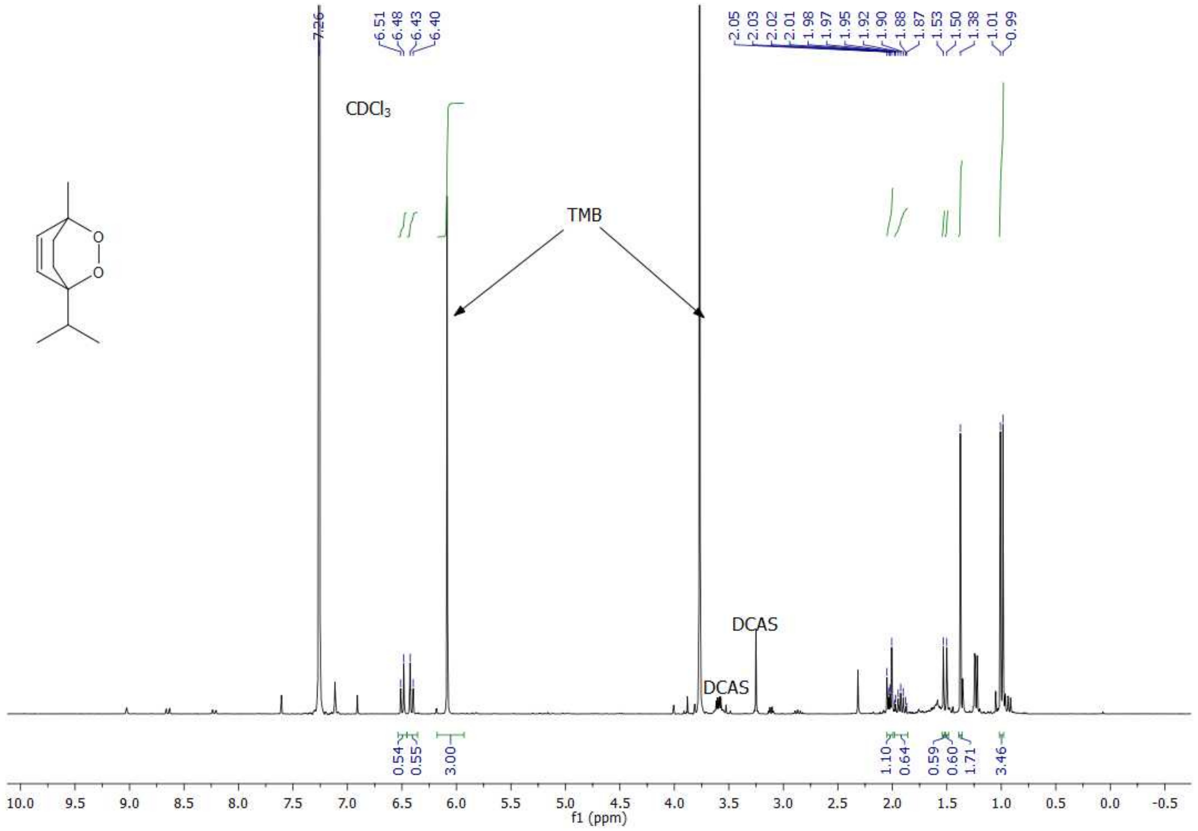

^{1}H NMR yield adjusted to $\%_{wt}$ purity of a-terpenine used ($\%_{wt}$85 purity) = 65%

11. Estimation of *Exo*- : *Endo*- Selectivity by ^{1}H NMR

To estimate the exocyclic (*N*-C$\underline{H}_3$) : endocyclic (*N*-C$\underline{H}_2$) selectivities of oxidation for piperazine-containing compounds, ^{1}H NMR yields of relevant species were obtained using 1 equiv. (with respect to starting material solution) of 1,3,5-trimethoxybenzene as an internal standard. For exocyclic (*N*-CH_3) oxidation products (*N*-Formyl), the ^{1}H NMR yield was determined as shown on Table S4 (*vide supra*). For endocyclic (*N*-C$\underline{H}_2$) oxidation products, integration of remaining *N*-C*H*$_3$ peaks detected in the ^{1}H NMR spectrum of the crude reaction mixture was used.

Using compound **1h** as a representative example, its crude reaction mixture's LC-MS chromatogram (Figure S29) revealed two relevant structures, with $[M+H]^+$ of 271.1115 (R_t = 1.308 min) and 269.0596 (overlapping with an unknown compound with *m*/*z*: 257.0959 at R_t = 1.565-1.676 min) consistent with structures of **2h''** and **2h'** respectively (**2h** is at R_t = 1.830 min). Heavier components (with *m*/*z* > 280) were observed which derive from over oxidized products and cannot be accounted for in the estimation of *exo*- : *endo*- oxidation selectivity.

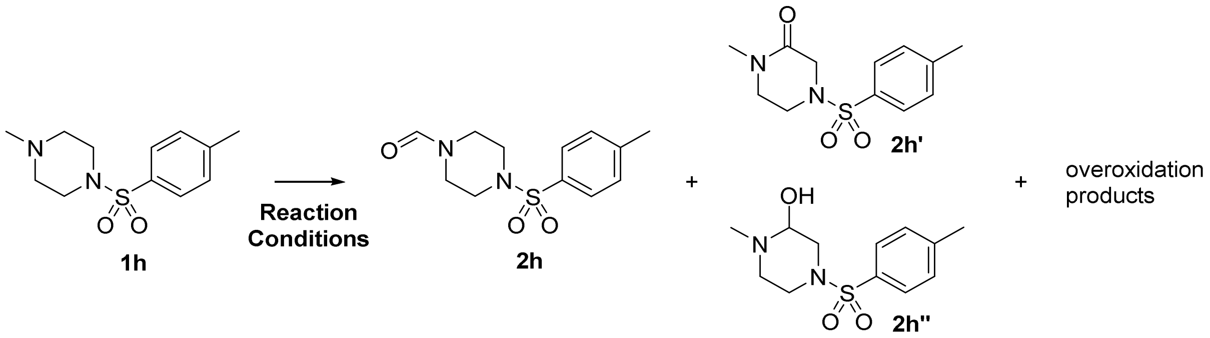

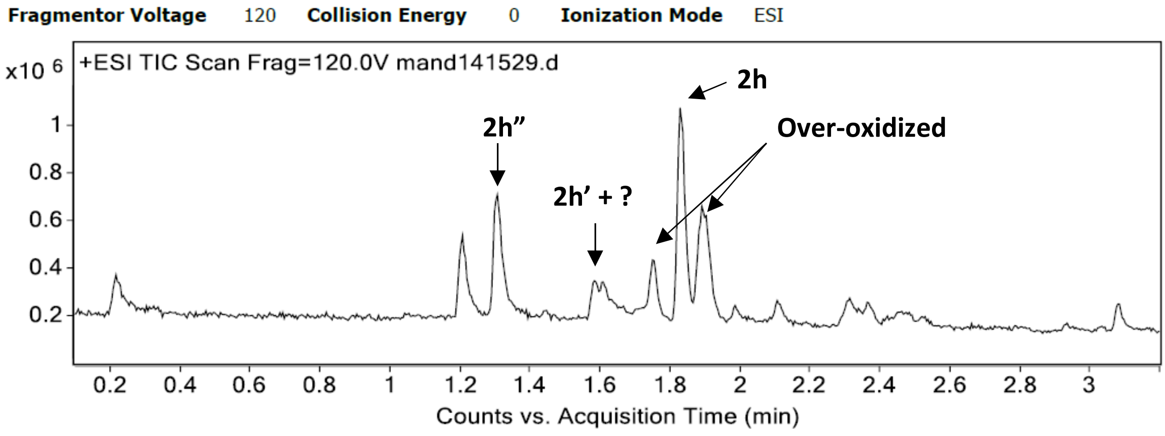

Figure S29. LC-MS profile of the crude reaction mixture of **1h** as a representative for selectivity calculation

In the ^{1}H NMR spectrum (Figure S30), three singlet peaks (2.96, 2.90, and 2.87 ppm) above 2.27 ppm (*N*-C$\underline{H}_3$ of **1h**) were observed which is consistent with the *N*-C$\underline{H}_3$ peaks of the endocyclic oxidation products (over-oxidized products would render the electronic environment of the neighboring H more deshielded, thus a downfield shift). These peaks (ca. 2.9 ppm) are also consistent with the structurally-related known *N*-C$\underline{H}_3$ signal of 1-methylpiperidin-2-one (2.86 ppm).[30]

Since it is yet unknown if overoxidized products form i) *via* exocyclic *N*-CH_3 oxidation followed by endocyclic *N*-CH_2 oxidation, **or** i) *via* endocyclic *N*-CH_2 oxidation followed by exocyclic *N*-CH_3 oxidation, it is not possible to include overoxidized products in the determination of selectivity. Therefore, we report selectivity as the 'maximum possible selectivity' by taking the ratio **2h** : (**2h'** + **2h''**). Selectivities for **2m, 2n,** and **2o**, were not obtained due to the intractable profiles of their crude ^{1}H NMRs.

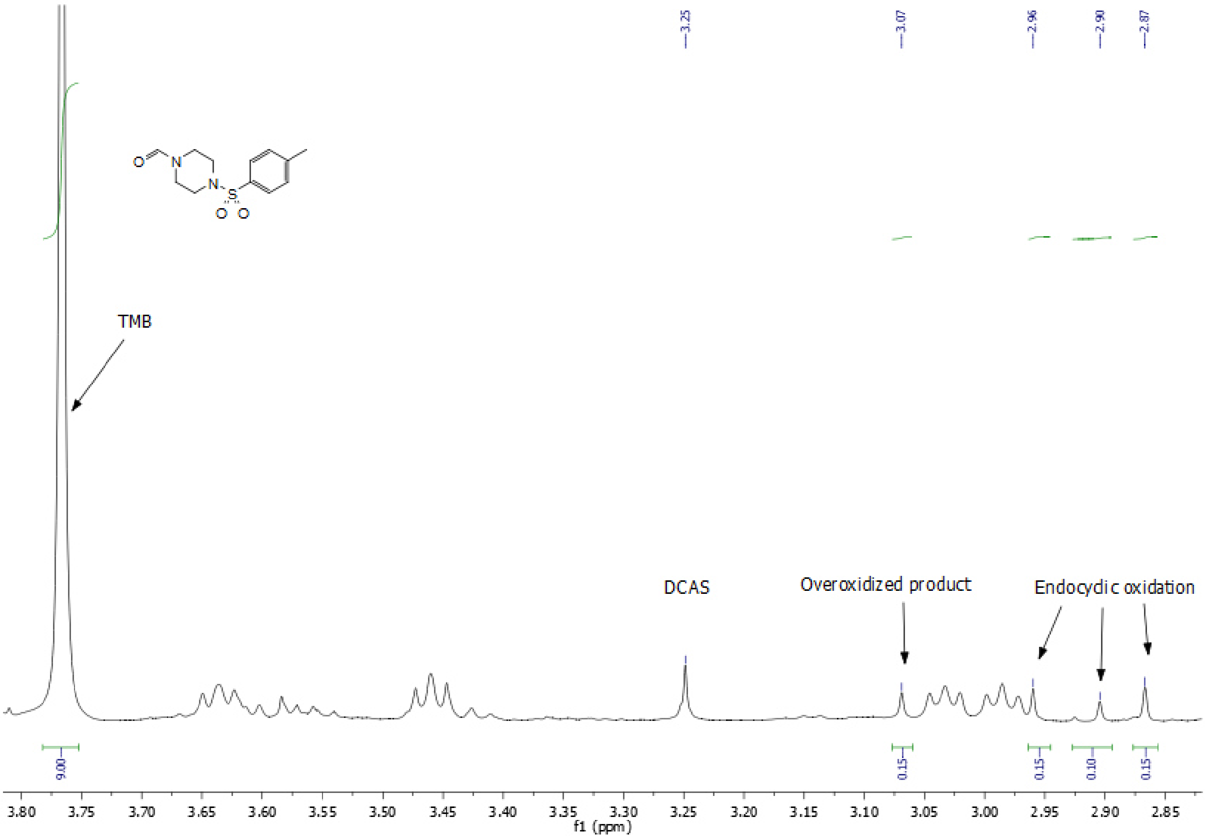

Figure S30. ^{1}H NMR spectra of the crude reaction mixture of **1h.** 1,3,5-trimethoxybenzene (1 equiv.) was used as a ^{1}H NMR internal standard. Its integration at 3.77 ppm (OCH_3) was normalized to 9 units. (**2h'** + **2h''**) = (total integration of singlets from 2.96 → 2.87) / 3.

12. X-Ray Cystallography

Single crystal XRD data were recorded for a suitable crystal of **DCAS** (dimensions = 0.10 × 0.05 × 0.01 mm^3). The crystal was mounted on a MITIGEN holder with inert oil on a XtaLAB Synergy R, DW system, HyPix-Arc 150 diffractometer using Cu-Kα radiation (λ= 1.54184 Å). The crystal was kept at a steady T = 100.01(10) K during data collection. Empirical multi-scan[30] and analytical absorption corrections[31] were applied to the data. Structures were solved using SHELXT[32] using dual methods and Olex2 as the graphical interface.[33] and least-squares refinements on F2 were carried out using SHELXL.[32,34]

All non-hydrogen atoms were refined anisotropically. Hydrogen atom positions were calculated geometrically and refined using the riding model. Most hydrogen atom positions were calculated geometrically and refined using the riding model, but some hydrogen atoms were refined freely. To visualize the crystals in this study, the program Diamond[35] of Crystal Impact was used and all displacement ellipsoids are shown with a probability factor of 50%.

The crystallographic data for **DCAS** are submitted to The Cambridge Crystallographic Data Centre (Deposition Number 2109522).

Table S8. Crystallographic data and structure refinement for **DCAS**

**Compound**	**DCAS**
**Formula**	$C_{28}H_{34}N_4O_8S_2$
**$r_{calc.}$/(g cm^{-3})**	1.377
**μ/mm-1**	2.091
**Formula Weight**	618.71
**Colour**	clear light yellow
**Shape**	needle-shaped
**Size/mm^3**	0.10×0.05×0.01
**T/K**	123.01(10)
**Crystal System**	monoclinic
**Space Group**	C_2/c
**a/Å**	26.7306(7)
**b/Å**	5.04920(10)
**c/Å**	24.8887(5)
**α/°**	90
**β/°**	117.317(3)
**γ/°**	90
**V/Å^3**	2984.57(14)
**Z**	4
**Z'**	0.5
**Wavelength/Å**	1.54184
**Radiation type**	Cu Kα
**Θmin/°**	3.722
**Θmax/°**	73.171
**Measured Refl's.**	25114
**Indep't Refl's**	2930
**Refl's I≥2 σ(I)**	2584
**R_{int}**	0.0354
**Parameters**	221
**Restraints**	46
**Largest Peak**	0.811
**Deepest Hole**	-0.393
**GooF**	1.055
**wR_2 (all data)**	0.1216
**wR_2**	0.1179
**R_1 (all data)**	0.0509
**R_1**	0.0448

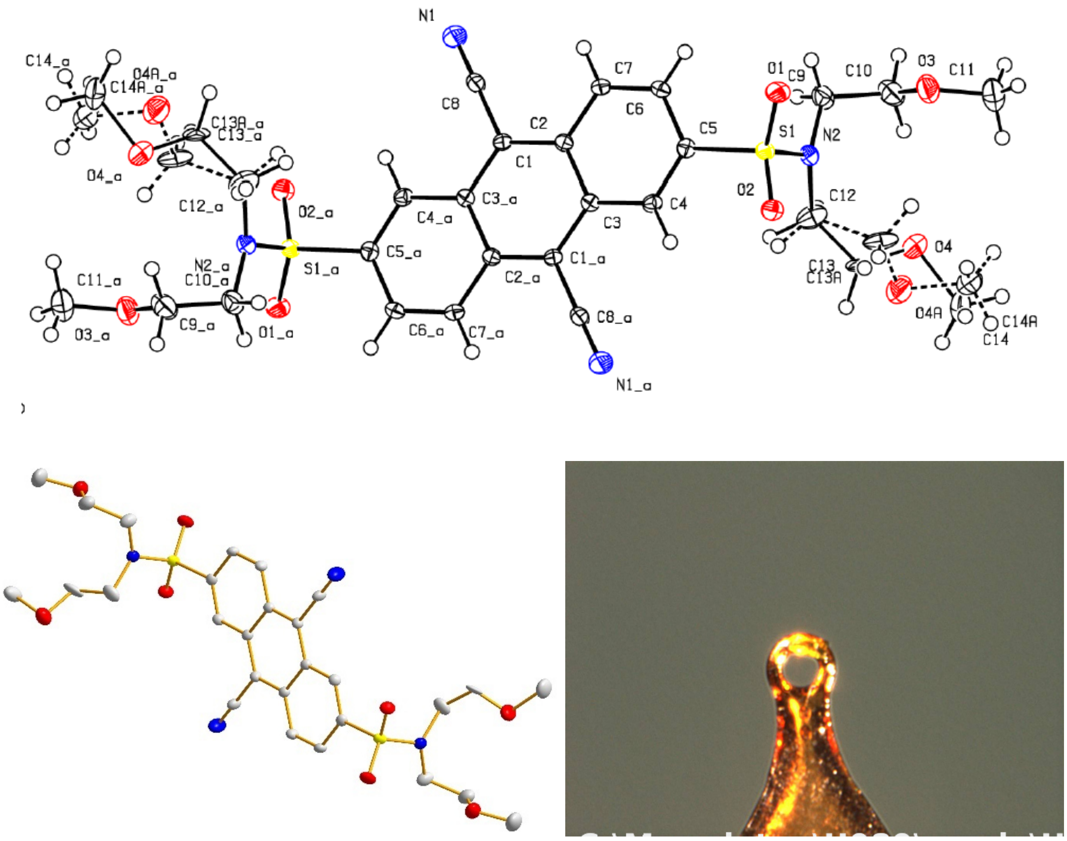

Figure S31. Solid-state molecular structure of **DCAS** including the numbering scheme (top). Visualized using Diamond 4 with H atoms are omitted for clarity (bottom left). Thermal ellipsoids are set at the 50% probability level. C atoms shown in grey, N atoms in blue and O atoms in red S atoms in yellow. Picture of **DCAS** Crystal in the diffractometer (bottom right).

Table S9: Bond lengths for **DCAS**

Atom	Atom	Length/Å
S1	O2	1.4342(15)
S1	O1	1.4321(15)
S1	N2	1.6120(19)
S1	C5	1.772(2)
O3	C11	1.423(3)
O3	C10	1.407(3)
N2	C9	1.479(3)
N2	C12	1.471(3)
N1	C8	1.141(3)
C5	C4	1.360(3)
C5	C6	1.425(3)
C4	C3	1.421(3)
C3	C1[1]	1.411(3)
C3	C2	1.433(3)
C1	C2	1.407(3)
C1	C8	1.444(3)
C6	C7	1.361(3)
C13	C12	1.476(5)
C13	O4	1.383(6)
C2	C7	1.424(3)

Atom	Atom	Length/Å
C10	C9	1.502(3)
C12	C13A	1.616(7)
O4	C14	1.607(6)
O4A	C14A	1.617(8)
O4A	C13A	1.399(7)

Table S10. Bond angles for **DCAS**

Atom	Atom	Atom	Angle/°
O2	S1	N2	107.28(10)
O2	S1	C5	106.74(9)
O1	S1	O2	120.25(10)
O1	S1	N2	107.84(10)
O1	S1	C5	106.96(9)
N2	S1	C5	107.13(10)
C10	O3	C11	111.2(2)
C9	N2	S1	119.26(15)
C12	N2	S1	117.36(15)
C12	N2	C9	118.25(19)
C4	C5	S1	119.36(16)
C4	C5	C6	121.84(19)
C6	C5	S1	118.76(15)
C5	C4	C3	120.00(19)
C4	C3	C2	118.88(18)
C1^{1}	C3	C4	122.36(18)
C1^{1}	C3	C2	118.76(19)
C3^{1}	C1	C8	118.49(18)
C2	C1	C3^{1}	122.60(18)
C2	C1	C8	118.90(18)
C7	C6	C5	119.23(18)
O4	C13	C12	106.1(4)
C1	C2	C3	118.63(18)
C1	C2	C7	122.51(18)
C7	C2	C3	118.85(18)
N1	C8	C1	179.1(3)
C6	C7	C2	121.19(19)
O3	C10	C9	109.4(2)
N2	C9	C10	111.6(2)
N2	C12	C13	121.4(3)
N2	C12	C13A	99.7(3)
C13	O4	C14	104.0(4)
C13A	O4A	C14A	87.9(5)
O4A	C13A	C12	108.1(5)

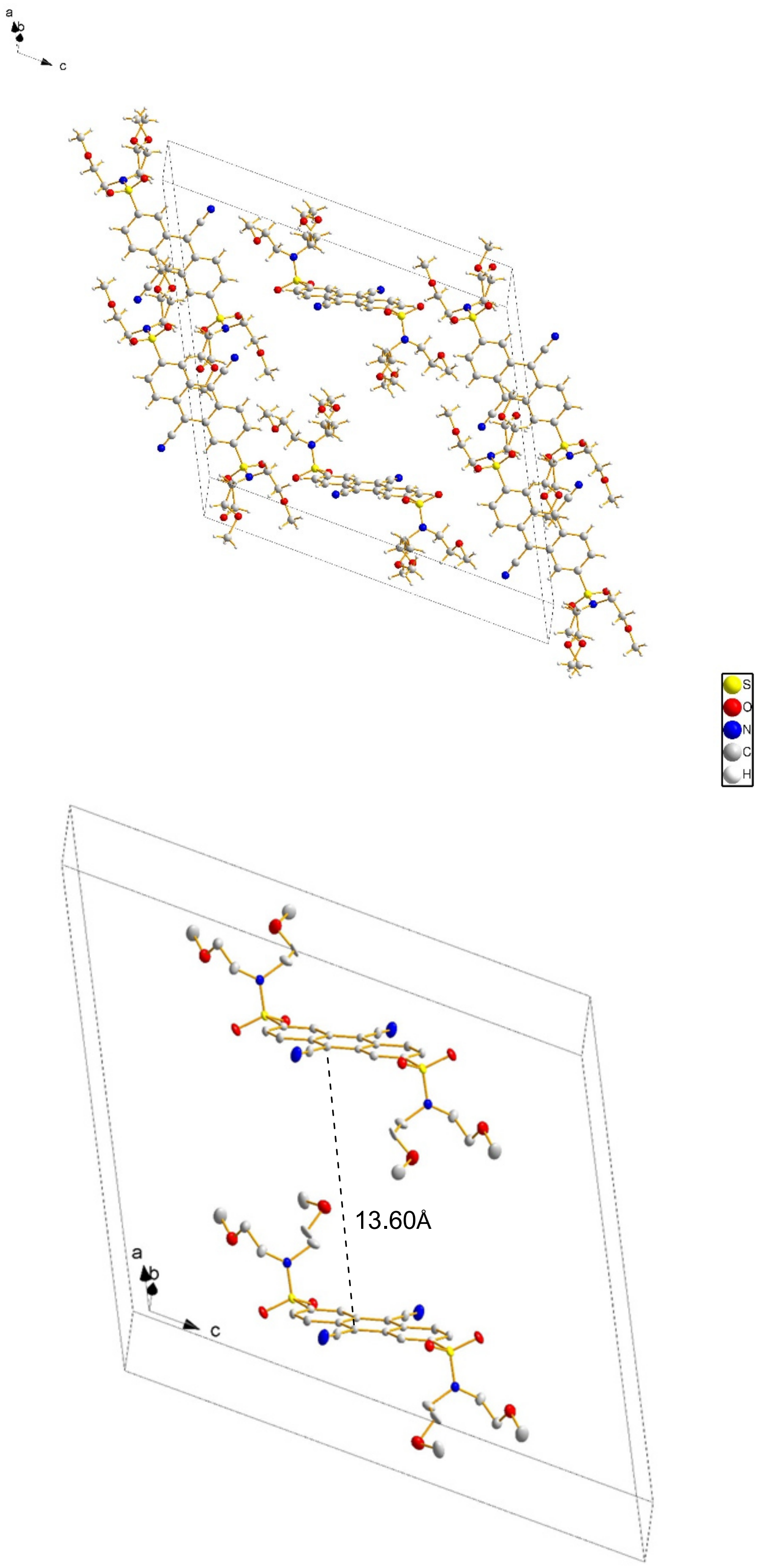

Figure S32. Unit cell of **DCAS** crystals showing all atoms (top). Distance between the dicyanoanthracene planes of **DCAS** molecules in a unit cell, all Hydrogens and other molecules of **DCAS** were omitted for clarity (bottom). Thermal ellipsoids are set at the 50% probability level. Visualized using Diamond 4 with H atoms in white, C atoms shown in grey, N atoms in blue and O atoms in red S atoms in yellow.

13. Density Functional Theory Computational Details and Cartesian Coordinates

Computations for the free energies were performed using Density Functional Theory (DFT)[36] using the Gaussian09 software package.[37] Geometry optimizations and frequency calculations were carried out using the CAM-B3LYP[38] hybrid-functional or the ωB97X-D[39] functional with a 6-31++g(2d,p)[40] basis set. Solvation was modelled implicitly using the Conductor-like Polarisable Continuum Model (CPCM)[41] in acetonitrile. These functionals were recommended for excited state calculations[42] and were used in the literature for calculations of kinetic and thermodynamic parameters of related radical systems.[23,43] For excited states, vertical excitation energy was calculated using Time Dependent-Density Functional Theory (TD-DFT)[44] with Tamm-Dancoff Approximation[45] (code: "tda=(nstates=6)"). The Free energy barrier for photoinduced SETs ([$\Delta G^{\ddagger}_{SET}$, kcal mol^{-1}) were calculated using Marcus theory (Equation S1):

$$\Delta G^{\neq}_{SET} = \frac{(\lambda + \Delta G^{o}_{SET})^2}{4\lambda}$$

Equation S1 Marcus theory equation. $\Delta G^{\neq}_{SET}$ = activation energy of SET, ΔG^{o}_{SET} = standard free energy of SET, λ = reorganization energy

The reorganization energy was estimated according to the literature[46] (Equation S2):

$$\lambda \approx \lambda_o = 332\left(\frac{1}{\varepsilon_{op}} - \frac{1}{\varepsilon}\right)\left(\frac{1}{2r_1} + \frac{1}{2r_2} - \frac{1}{R_{12}}\right) \qquad where: R_{12} = r_1 + r_2$$

Equation S2 Estimation of reorganization energy

Here, λ is the reorganization energy and is approximated as λ_o (outer sphere reorganization energy); ε is the static dielectric constant of the solvent (acetonitrile = 37.5);[47] ε_{op} is the optical dielectric constant of the solvent ($\varepsilon_{op} = \eta^2$, acetonitrile = 1.344^2 = 1.8066);[48] r_1 and r_2 are the calculated radius of the reacting species in Å (gaussian 09, DFT using the code "volume"). In the literature, the calculated (using the same functionals) free energy quenching of **^{1}DCA*** by an allyl radical *via* SET is intuitively very exergonic (-2.7 eV or -62.3 kcal mol^{-1}).[43] In another paper calculated energy barriers for SET of an excited photocatalyst with several aryl halides ranges from 0.9 to 29 kcal mol^{-1}.[49] Based on these and the mentioned experimental evidences in the results and discussion (*i.e.*, agreement with experimental UV-vis spectra, consistent hints from CV measurements and Stern-Volmer analysis), the chosen computational parameters were deemed sufficient and the calculated thermodynamic and kinetic data in this study was gauged to falls within, a sensible range.

Free energies for the SET and HAT elementary steps were calculated with Tropine **1b** as representative amine substrate (Figure 33). Cartesian coordinates of the optimized structures are shown.

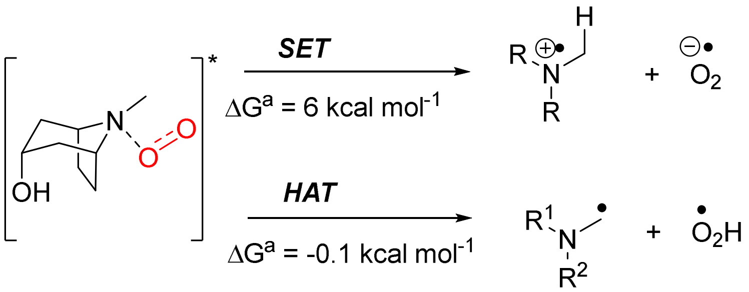

Figure 33. SET *vs* HAT Elemetary step of tropine – $^{1}O_2$ complex.

<u>**Cartesian Coordinates and Results from (TD)-DFT Computations**</u>

SET of between dicyanoanthracenes and 1b:

DCA

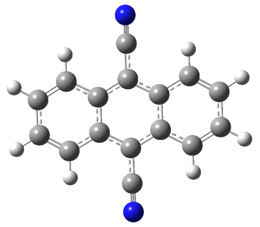				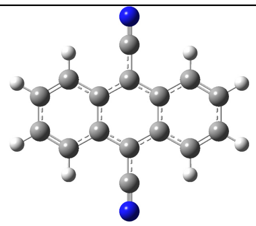			
functional: CAM-B3LYP				functional: ωB97X-D			
Charge = 0, Multiplicity = 1				Charge = 0, Multiplicity = 1			
C	0.00000000	0.00000000	0.00000000	C	2.48366600	1.40405400	0.00008500
C	0.00000000	0.00000000	1.36078207	C	3.65711100	0.71149400	0.00006000
C	2.41489543	0.00000000	1.42384007	C	2.48361800	-1.40414400	-0.00008100
C	2.45705848	-0.00007675	-0.00089419	C	1.23343400	-0.71518800	-0.00001600
C	3.66473828	-0.00023105	-0.71859461	C	-0.00001500	-1.39090600	0.00001600
C	3.70812048	-0.00027094	-2.12271632	C	-1.23340900	-0.71514700	0.00003900
C	4.93440468	-0.00058236	-2.84932884	C	-2.48363700	-1.40405900	0.00010000
C	4.93440078	-0.00041036	-4.21008652	C	-3.65706900	-0.71150400	0.00005300
C	2.51949111	0.00023965	-4.27317731	C	-2.48358900	1.40414800	-0.00008900
C	2.47730645	0.00003540	-2.84841631	C	-1.23338500	0.71519400	-0.00001400
C	1.26966648	0.00015369	-2.13075393	C	0.00003500	1.39090500	0.00000500
C	1.22626083	0.00003058	-0.72657871	C	1.23345900	0.71514300	0.00002800
C	0.03548521	0.00036259	-2.85842757	C	0.00008100	2.82526800	-0.00003300
N	-0.95838259	0.00059997	-3.44420096	N	-0.00001900	3.98131600	0.00000800
C	4.89890986	-0.00039794	0.00908957	C	-0.00002500	-2.82526800	-0.00000600
N	5.89253238	-0.00053031	0.59527903	N	-0.00027200	-3.98131600	-0.00003200
H	-0.93523055	-0.00003946	-0.54708871	H	2.48883100	2.48850700	0.00016100
H	-0.93876960	-0.00011018	1.90256044	H	4.60095900	1.24465300	0.00013000
H	3.34634174	0.00004867	1.97734726	H	2.48874400	-2.48859600	-0.00015800
H	5.86962298	-0.00099490	-2.30221948	H	-2.48878900	-2.48851200	0.00020800
H	5.87316942	-0.00068817	-4.75187125	H	-4.60092200	-1.24465300	0.00012200
H	1.58804009	0.00054098	-4.82668033	H	-2.48870800	2.48860100	-0.00016000
C	1.22417902	0.00004175	2.08256898	C	3.65708700	-0.71162500	-0.00005500
H	1.20454519	0.00016846	3.16628031	H	4.60091600	-1.24481600	-0.00012300
C	3.71019218	0.00007890	-4.93189204	C	-3.65704600	0.71163400	-0.00006800
H	3.72983569	0.00033618	-6.01560000	H	-4.60088100	1.24481400	-0.00015400

Sum of electronic and thermal Free Energies= -723.515683 Hartree<br><br>Recommended a0 for SCRF calculation = 4.87 angstrom<br><br>**TD-DFT S1 (^{1}DCA*) =** 3.2717 eV	Sum of electronic and thermal Free Energies= -723.660735 Hartree<br><br>Recommended a0 for SCRF calculation = 4.96 angstrom<br><br>**TD-DFT S1 (^{1}DCA*) =** 3.2918 eV

DCA radical anion

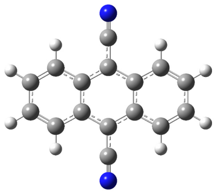	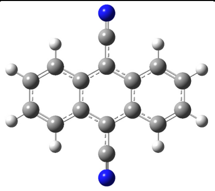
functional: CAM-B3LYP	functional: ωB97X-D
Charge = -1, Multiplicity = 2	Charge = -1, Multiplicity = 2

C	2.49172100	1.38695700	-0.00023900	C	2.49493100	-1.38909900	0.00003400
C	3.68770000	0.70111100	-0.00041600	C	3.69182300	-0.70162900	0.00004700
C	2.49187700	-1.38669000	-0.00006700	C	2.49479200	1.38936300	0.00002000
C	1.25214900	-0.71230800	0.00007800	C	1.25400200	0.71315700	0.00000200
C	0.00001700	-1.41056400	0.00020500	C	-0.00002400	1.41091700	-0.00000700
C	-1.25219800	-0.71242800	0.00011000	C	-1.25397100	0.71301800	0.00000100
C	-2.49185800	-1.38695800	0.00011900	C	-2.49482600	1.38909900	0.00001200
C	-3.68782800	-0.70111000	-0.00022300	C	-3.69171800	0.70163000	0.00003900
C	-2.49199400	1.38667600	-0.00037300	C	-2.49468700	-1.38936300	0.00005100
C	-1.25228100	0.71230800	-0.00004800	C	-1.25389900	-0.71315500	0.00001700
C	-0.00014800	1.41056100	0.00008500	C	0.00012400	-1.41091700	-0.00001500
C	1.25207400	0.71245900	-0.00002200	C	1.25407400	-0.71302000	0.00001200
C	-0.00023900	2.81811700	0.00036400	C	0.00021200	-2.82003300	-0.00006200
N	0.00009900	3.98146900	0.00078000	N	-0.00001400	-3.98596600	0.00025900
C	0.00004500	-2.81811900	0.00037100	C	-0.00008100	2.82003300	-0.00003300
N	0.00090600	-3.98147000	0.00030800	N	-0.00078200	3.98596600	-0.00050000
H	2.49579700	2.47184900	-0.00027800	H	2.50093000	-2.47495900	0.00004300
H	4.62489800	1.24720100	-0.00048100	H	4.62971800	-1.24704300	0.00006600
H	2.49605200	-2.47158400	0.00004700	H	2.50068300	2.47522200	0.00001800
H	-2.49590900	-2.47184600	0.00049300	H	-2.50082500	2.47496100	-0.00000100
H	-4.62500200	-1.24724900	-0.00036700	H	-4.62961300	1.24704300	0.00004500
H	-2.49621900	2.47156800	-0.00044300	H	-2.50057900	-2.47522500	0.00007200
C	3.68778100	-0.70070900	-0.00029600	C	3.69175200	0.70201300	0.00004000
H	4.62504300	-1.24669500	-0.00053200	H	4.62959300	1.24752000	0.00005300
C	-3.68791000	0.70070300	-0.00051700	C	-3.69164700	-0.70201300	0.00006000
H	-4.62514900	1.24672500	-0.00084000	H	-4.62948700	-1.24752100	0.00008800

Sum of electronic and thermal Free Energies= -723.647675 Hartree	Sum of electronic and thermal Free Energies= -723.792692 Hartree

DCAS

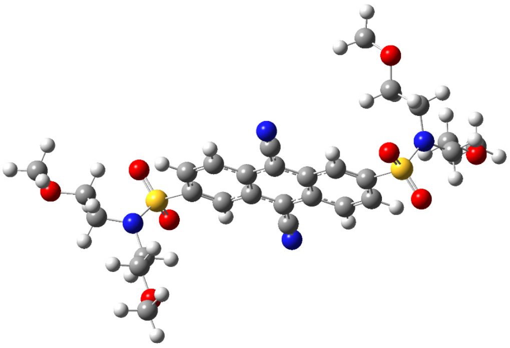				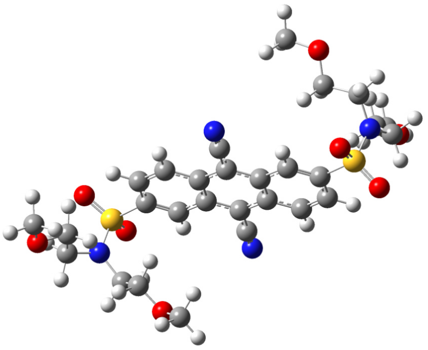			
functional: CAM-B3LYP’				functional: ωB97X-D			
Charge = 0, Multiplicity = 1				Charge = 0, Multiplicity = 1			
C	-2.07923400	1.56139000	0.08457900	C	-2.02819900	1.69061300	-0.67030300
C	-3.26030600	1.16777700	-0.45518200	C	-3.21611600	1.24282800	-1.15508300
C	-3.37876800	-0.13638700	-1.00785400	C	-3.43063200	-0.15342900	-1.31682200
C	-2.33918600	-1.00860700	-1.01415000	C	-2.46328200	-1.06402200	-1.02512900
C	-1.08633300	-0.62292800	-0.45459100	C	-1.20779400	-0.62448300	-0.51119900
C	0.01654200	-1.49116400	-0.43869700	C	-0.18388700	-1.52879500	-0.18130200
C	1.25149600	-1.11410600	0.11023000	C	1.05097000	-1.10181500	0.33400300
C	2.37325500	-1.99499200	0.13848100	C	2.09771600	-2.01550600	0.66393100
C	3.55405800	-1.60133100	0.67986400	C	3.28531900	-1.56601700	1.14971800
C	3.67997400	-0.29145400	1.21789000	C	3.48830600	-0.16992700	1.33493600
C	2.63850700	0.57919400	1.22295900	C	2.51297500	0.73558000	1.05966000
C	1.38499100	0.19278700	0.66656400	C	1.26282200	0.29566400	0.53615500
C	0.28094000	1.06031700	0.65210400	C	0.24087400	1.20033900	0.20211100
C	-0.95435800	0.68309000	0.10393200	C	-0.99075800	0.77385800	-0.32007200
S	-4.91568100	-0.60759500	-1.75893100	S	-5.03916600	-0.72591700	-1.81940700
N	-5.97690100	-0.54564400	-0.51301500	N	-5.91175600	-0.75601700	-0.44587500
O	-5.30527700	0.43384500	-2.68181600	O	-5.63183600	0.27518900	-2.67121900
O	-4.74651200	-1.95418900	-2.25632700	O	-4.85666900	-2.07280200	-2.30029800
C	-5.86558400	-1.60134100	0.49575900	C	-5.55338500	-1.72333600	0.58705500
C	-7.30589100	0.03117400	-0.76148500	C	-6.98773900	0.20200200	-0.19958700
C	-6.00424700	-1.03850100	1.90044500	C	-4.88202700	-1.08301800	1.79754000
C	-7.39063000	1.47943400	-0.31062800	C	-6.51204100	1.58216800	0.24126200
O	-8.70840400	1.91703600	-0.54118700	O	-7.63894800	2.28643400	0.69729900
O	-5.88233000	-2.11722600	2.79526500	O	-4.44228200	-2.12748500	2.62731000
C	-8.90932400	3.26418000	-0.16848000	C	-7.33599100	3.61857700	1.05129200
C	-6.01354300	-1.72319300	4.14533700	C	-3.77248800	-1.65981800	3.77788500
S	5.23568400	0.19912700	1.92797400	S	5.07577100	0.40354900	1.90256400

O	5.67778600	-0.92418100	2.71926400
N	6.28812100	0.39867300	0.69972000
O	5.02452000	1.48784900	2.54075300
C	6.49303400	1.73117700	0.12491300
C	7.18946100	-0.68325500	0.30129300
C	6.98345800	-1.12805400	-1.13895800
C	5.49317200	2.08928100	-0.96486000
O	7.88427100	-2.18268400	-1.37553500
O	5.87532000	3.34119300	-1.48135100
C	5.00322400	3.80565000	-2.49056900
C	7.82558900	-2.66441400	-2.70205800
C	0.42039000	2.37101900	1.21400000
N	0.53357800	3.42560300	1.66590800
C	-0.12308400	-2.80211500	-1.00031200
N	-0.23604400	-3.85650800	-1.45262900
H	-1.98124600	2.55394200	0.50673500
H	-4.10963400	1.83909200	-0.46962700
H	-2.46058600	-1.99365000	-1.44664700
H	2.27188600	-2.99264700	-0.27070700
H	4.39219900	-2.28574800	0.71774800
H	2.75701600	1.56467000	1.65530500
H	-6.62252700	-2.37381100	0.32145400
H	-4.88827700	-2.07894100	0.40714800
H	-7.56668500	-0.03750800	-1.81847100
H	-8.02990600	-0.57219100	-0.20885600
H	-5.22043900	-0.28979200	2.08485500
H	-6.97911400	-0.54751600	2.03389300
H	-7.13816600	1.56697800	0.75603600
H	-6.67898700	2.09317200	-0.88030300
H	-9.95020800	3.51055300	-0.38219800
H	-8.71576800	3.40939300	0.90248900
H	-8.25406100	3.93317100	-0.74167900
H	-5.90405700	-2.61871600	4.75822800
H	-5.23639700	-0.99844800	4.42100400
H	-6.99890000	-1.27579500	4.33040300
H	6.45708100	2.48108600	0.91532600
H	7.50236200	1.74139900	-0.29325100
H	8.22193300	-0.34391000	0.43421100
H	7.03778900	-1.53017000	0.96840000
H	5.94737400	-1.46232200	-1.29570900
H	7.18064400	-0.30348900	-1.83890300
H	5.49092500	1.33011500	-1.75963900
H	4.47566800	2.14488800	-0.55329900
H	5.37812900	4.77366300	-2.82539900
H	4.98098400	3.11223300	-3.34147800
H	3.98292800	3.92668900	-2.10375400
H	8.55304300	-3.47258700	-2.78767100
H	6.82535600	-3.05149300	-2.93636300
H	8.07768100	-1.87260900	-3.41953300

Sum of electronic and thermal Free Energies= -2703.200082 Hartree

Recommended a0 for SCRF calculation = 6.75 angstrom

O	5.54721400	-0.59401000	2.83049400
N	6.05975000	0.40344600	0.60651400
O	4.91939700	1.77621200	2.31217400
C	6.25796300	1.62645000	-0.17305800
C	6.63550800	-0.84904300	0.12312400
C	6.01677800	-1.33442600	-1.18345300
C	5.08029000	2.00122200	-1.06639000
O	6.58706800	-2.58503600	-1.47163800
O	5.49512800	3.07516300	-1.87110800
C	4.46302600	3.55299800	-2.70666500
C	6.10183100	-3.13352600	-2.67837800
C	0.46872200	2.60327100	0.39331400
N	0.65749400	3.73281500	0.54589000
C	-0.40859000	-2.93162400	-0.37646100
N	-0.59253800	-4.06143400	-0.53302800
H	-1.86072400	2.75424200	-0.54806400
H	-3.99401600	1.94298900	-1.43166500
H	-2.64377500	-2.12183300	-1.17630900
H	1.93863800	-3.07833500	0.52476800
H	4.07111400	-2.26535500	1.40856300
H	2.68168300	1.79106300	1.23817400
H	-6.45725700	-2.25798300	0.89483100
H	-4.87656200	-2.46379000	0.15610800
H	-7.61380200	0.29168300	-1.08842300
H	-7.60397200	-0.23055700	0.59357100
H	-4.03312700	-0.45984600	1.47592100
H	-5.58758400	-0.43684300	2.34171100
H	-5.75692600	1.50014500	1.03805500
H	-6.05408500	2.11187300	-0.60610700
H	-8.25800900	4.07783300	1.41073300
H	-6.58015500	3.65228200	1.84792900
H	-6.96344300	4.18156000	0.18455900
H	-3.47143600	-2.53463200	4.35626100
H	-2.87881500	-1.08145600	3.50461100
H	-4.43065400	-1.02894300	4.39093700
H	6.48875700	2.45368100	0.49967900
H	7.13942300	1.45219100	-0.79584000
H	7.71189200	-0.70191400	-0.01121000
H	6.50794300	-1.61228600	0.89221200
H	4.92367600	-1.42093400	-1.08201900
H	6.22480500	-0.62577900	-1.99958800
H	4.77067000	1.14649800	-1.68696400
H	4.21670000	2.30141500	-0.45614700
H	4.86962600	4.38685900	-3.28076400
H	4.12028600	2.77044700	-3.39749300
H	3.60650300	3.90413100	-2.11462800
H	6.59114200	-4.09868800	-2.81707600
H	5.01415600	-3.28186900	-2.63310700
H	6.33741600	-2.48168800	-3.53086100

Sum of electronic and thermal Free Energies= -2703.476834 Hartree

Recommended a0 for SCRF calculation = 6.63 angstrom

TD-DFT S1 ([1]DCAS *) = 3.3469 eV	TD-DFT S1 ([1]DCAS*)' = 3.3320 eV

DCAS radical anion

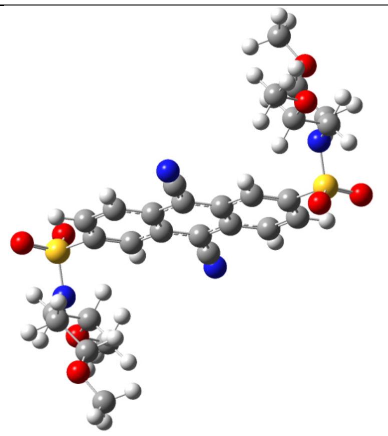

functional: CAM-B3LYP

Charge = -1, Multiplicity = 2

C	1.28998300	2.26694800	1.15552000
C	2.61860400	2.22378300	1.50715700
C	3.34128500	1.04551000	1.29005600
C	2.74112600	-0.06770900	0.73246500
C	1.38733700	-0.04501800	0.36697200
C	0.73590800	-1.18198100	-0.21425000
C	-0.63751700	-1.15164900	-0.58243000
C	-1.28993800	-2.26625900	-1.15577300
C	-2.61855500	-2.22308700	-1.50742500
C	-3.34123600	-1.04481100	-1.29033500
C	-2.74107800	0.06840300	-0.73273300
C	-1.38729100	0.04570900	-0.36723300
C	-0.73585900	1.18267600	0.21397900
C	0.63756400	1.15234000	0.58217100
S	5.02712000	0.94992500	1.78533700
N	5.82907800	0.62548600	0.38229300
O	5.39492800	2.24651100	2.32164100
O	5.23350000	-0.20921700	2.62989900
C	7.02828500	-0.21747800	0.47267100
C	5.83093100	1.69585400	-0.61465500
C	6.73463800	-1.65267100	0.07074900
C	5.62888000	1.13958900	-2.01373700
O	5.65494500	2.22596400	-2.90872500
O	7.94024900	-2.37648300	0.15712500

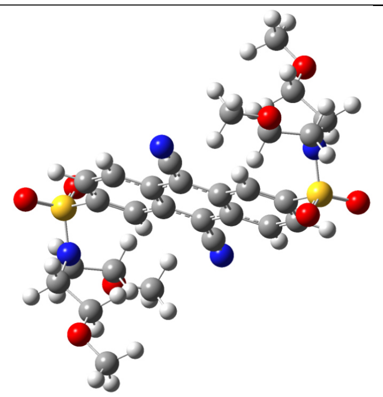

functional: ωB97X-D

Charge = -1, Multiplicity = 2

C	-2.02819900	1.69061300	-0.67030300
C	-3.21611600	1.24282800	-1.15508300
C	-3.43063200	-0.15342900	-1.31682200
C	-2.46328200	-1.06402200	-1.02512900
C	-1.20779400	-0.62448300	-0.51119900
C	-0.18388700	-1.52879500	-0.18130200
C	1.05097000	-1.10181500	0.33400300
C	2.09771600	-2.01550600	0.66393100
C	3.28531900	-1.56601700	1.14971800
C	3.48830600	-0.16992700	1.33493600
C	2.51297500	0.73558000	1.05966000
C	1.26282200	0.29566400	0.53615500
C	0.24087400	1.20033900	0.20211100
C	-0.99075800	0.77385800	-0.32007200
S	-5.03916600	-0.72591700	-1.81940700
N	-5.91175600	-0.75601700	-0.44587500
O	-5.63183600	0.27518900	-2.67121900
O	-4.85666900	-2.07280200	-2.30029800
C	-5.55338500	-1.72333600	0.58705500
C	-6.98773900	0.20200200	-0.19958700
C	-4.88202700	-1.08301800	1.79754000
C	-6.51204100	1.58216800	0.24126200
O	-7.63894800	2.28643400	0.69729900
O	-4.44228200	-2.12748500	2.62731000

C	5.48279500	1.82843300	-4.25266100	C	-7.33599100	3.61857700	1.05129200
C	7.78411400	-3.73731700	-0.18352100	C	-3.77248800	-1.65981800	3.77788500
S	-5.02707900	-0.94920900	-1.78558100	S	5.07577100	0.40354900	1.90256400
O	-5.39469700	-2.24556900	-2.32255000	O	5.54721400	-0.59401000	2.83049400
N	-5.82911300	-0.62561200	-0.38238300	N	6.05975000	0.40344600	0.60651400
O	-5.23358400	0.21035200	-2.62953900	O	4.91939700	1.77621200	2.31217400
C	-7.02837900	0.21731200	-0.47230300	C	6.25796300	1.62645000	-0.17305800
C	-5.83087100	-1.69648600	0.61400600	C	6.63550800	-0.84904300	0.12312400
C	-5.62897400	-1.14094700	2.01339900	C	6.01677800	-1.33442600	-1.18345300
C	-6.73482500	1.65228300	-0.06952400	C	5.08029000	2.00122200	-1.06639000
O	-5.65479300	-2.22783900	2.90778000	O	6.58706800	-2.58503600	-1.47163800
O	-7.94041400	2.37613700	-0.15571300	O	5.49512800	3.07516300	-1.87110800
C	-7.78438300	3.73672900	0.18595200	C	4.46302600	3.55299800	-2.70666500
C	-5.48270200	-1.83101900	4.25193400	C	6.10183100	-3.13352600	-2.67837800
C	-1.48018300	2.36437500	0.42527600	C	0.46872200	2.60327100	0.39331400
N	-2.09709200	3.33198800	0.59711100	N	0.65749400	3.73281500	0.54589000
C	1.48023900	-2.36367200	-0.42556800	C	-0.40859000	-2.93162400	-0.37646100
N	2.09714000	-3.33128100	-0.59745100	N	-0.59253800	-4.06143400	-0.53302800
H	0.72706800	3.17829500	1.32074300	H	-1.86072400	2.75424200	-0.54806400
H	3.10628200	3.08426000	1.94638600	H	-3.99401600	1.94298900	-1.43166500
H	3.32027100	-0.97000400	0.57356600	H	-2.64377500	-2.12183300	-1.17630900
H	-0.72702600	-3.17760900	-1.32099100	H	1.93863800	-3.07833500	0.52476800
H	-3.10622400	-3.08355700	-1.94668100	H	4.07111400	-2.26535500	1.40856300
H	-3.32021600	0.97070400	-0.57384400	H	2.68168300	1.79106300	1.23817400
H	7.78378100	0.20665300	-0.19369400	H	-6.45725700	-2.25798300	0.89483100
H	7.44234500	-0.20103400	1.48265100	H	-4.87656200	-2.46379000	0.15610800
H	5.01653100	2.38911200	-0.39589400	H	-7.61380200	0.29168300	-1.08842300
H	6.76618300	2.26536000	-0.56564200	H	-7.60397200	-0.23055700	0.59357100
H	5.97998000	-2.08226800	0.74324200	H	-4.03312700	-0.45984600	1.47592100
H	6.33827300	-1.69056900	-0.95432200	H	-5.58758400	-0.43684300	2.34171100
H	6.42240200	0.42278200	-2.27061900	H	-5.75692600	1.50014500	1.03805500
H	4.66577000	0.61248400	-2.07054400	H	-6.05408500	2.11187300	-0.60610700
H	5.51636900	2.72940900	-4.86661300	H	-8.25800900	4.07783300	1.41073300
H	6.28372800	1.14623600	-4.56694600	H	-6.58015500	3.65228200	1.84792900
H	4.51526900	1.32936200	-4.39493700	H	-6.96344300	4.18156000	0.18455900
H	8.76074100	-4.21356300	-0.08595100	H	-3.47143600	-2.53463200	4.35626100
H	7.07145400	-4.23265200	0.48910600	H	-2.87881500	-1.08145600	3.50461100
H	7.43038600	-3.84699700	-1.21728500	H	-4.43065400	-1.02894300	4.39093700
H	-7.44236500	0.20146200	-1.48232100	H	6.48875700	2.45368100	0.49967900
H	-7.78388000	-0.20729400	0.19375500	H	7.13942300	1.45219100	-0.79584000
H	-6.76603600	-2.26611200	0.56464900	H	7.71189200	-0.70191400	-0.01121000
H	-5.01635500	-2.38951400	0.39494300	H	6.50794300	-1.61228600	0.89221200
H	-4.66598600	-0.61365200	2.07051600	H	4.92367600	-1.42093400	-1.08201900
H	-6.42267100	-0.42448200	2.27068400	H	6.22480500	-0.62577900	-1.99958800
H	-6.33863300	1.68963700	0.95563400	H	4.77067000	1.14649800	-1.68696400
H	-5.98004500	2.08223600	-0.74165800	H	4.21670000	2.30141500	-0.45614700
H	-8.76099000	4.21303500	0.08847400	H	4.86962600	4.38685900	-3.28076400
H	-7.43092100	3.84566500	1.21988600	H	4.12028600	2.77044700	-3.39749300
H	-7.07155300	4.23255500	-0.48613300	H	3.60650300	3.90413100	-2.11462800
H	-5.51607900	-2.73234400	4.86538600	H	6.59114200	-4.09868800	-2.81707600
H	-4.51527700	-1.33182600	4.39447000	H	5.01415600	-3.28186900	-2.63310700
H	-6.28377200	-1.14916500	4.56661600	H	6.33741600	-2.48168800	-3.53086100
Sum of electronic and thermal Free Energies= -2703.349404 Hartree				Sum of electronic and thermal Free Energies= -2703.623224 Hartree			

TD-DFT for UV-vis simulation:

```
2.1681 eV  571.84 nm  f=0.0015
2.2681 eV  546.63 nm  f=0.0837
2.8156 eV  440.35 nm  f=0.1812
2.8700 eV  432.01 nm  f=0.0190
3.5374 eV  350.49 nm  f=0.1448
3.6019 eV  344.22 nm  f=0.0698
```

Tropine 1b

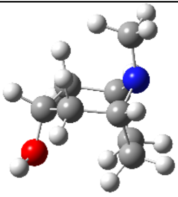

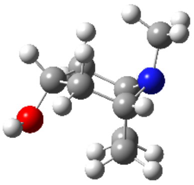

functional: CAM-B3LYP

Charge = 0, Multiplicity = 1

N	-1.46703300	0.17214500	0.01375000
C	-0.57773200	0.35912000	-1.13943700
C	-0.55511300	0.34795500	1.15048800
C	0.10667800	1.68680800	-0.77021000
C	0.12190900	1.67928900	0.78080200
C	0.41458500	-0.80622800	-1.28556600
C	1.24444400	-1.06522100	-0.02381300
C	0.43938200	-0.81920900	1.26205200
C	-2.31736900	-0.99889300	0.01651900
O	2.40443400	-0.22451400	-0.09418700
H	-1.17197700	0.44044400	-2.05498300
H	-1.13043500	0.41983900	2.07883600
H	-0.48594600	2.52440700	-1.14544600
H	1.10730200	1.76108800	-1.19710000
H	-0.46338300	2.51269400	1.17632200
H	1.13068200	1.75146000	1.18930800
H	1.09687100	-0.62202600	-2.12129600
H	-0.13959600	-1.71760400	-1.53173800
H	1.58490300	-2.10830800	-0.03296600
H	1.13036300	-0.64759400	2.09563900
H	-0.10940800	-1.73477300	1.50830500
H	-2.97136600	-0.97029400	-0.86070900
H	-2.95227800	-0.98012100	0.90792800
H	-1.78908900	-1.96464100	0.00538500
H	2.93641100	-0.37520000	0.69475000

```
Sum of electronic and thermal Free
Energies= -443.459558 Hartree
```

functional: ωB97X-D

Charge = 0, Multiplicity = 1

N	-1.47077600	0.17283000	0.01301200
C	-0.58047200	0.35851000	-1.13891200
C	-0.55912300	0.34814800	1.14931600
C	0.10300600	1.68885300	-0.77126600
C	0.11743200	1.68172500	0.78109400
C	0.41696200	-0.80535500	-1.28628300
C	1.24893100	-1.06340300	-0.02282800
C	0.43929800	-0.81798700	1.26381100
C	-2.30914900	-1.00740300	0.01573400
O	2.40671500	-0.22428900	-0.09452800
H	-1.17361100	0.43748700	-2.05593600
H	-1.13444600	0.41876600	2.07831500
H	-0.49099500	2.52567400	-1.14727300
H	1.10445100	1.76244200	-1.19814800
H	-0.46960400	2.51459500	1.17630000
H	1.12637100	1.75394000	1.19104000
H	1.09869000	-0.61574300	-2.12165600
H	-0.13441500	-1.71890400	-1.53388500
H	1.58636400	-2.10840300	-0.03098500
H	1.12738600	-0.64234500	2.09930600
H	-0.10796600	-1.73547100	1.50928700
H	-2.96503800	-0.98340900	-0.86080700
H	-2.94047300	-0.99626900	0.91043100
H	-1.76966900	-1.96835100	0.00116500
H	2.92335500	-0.35802800	0.70398300

```
Sum of electronic and thermal Free
Energies= -443.571850 Hartree
```

Recommended a0 for SCRF calculation = 4.32 angstrom	Recommended a0 for SCRF calculation = 4.67 angstrom

Tropine radical cation

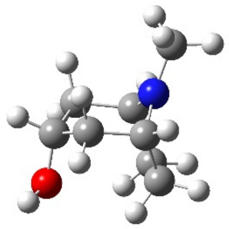

functional: CAM-B3LYP

Charge = 1, Multiplicity = 2

N	-1.32791200	-0.07883200	0.00667300
C	-0.55029700	0.27820600	-1.16176500
C	-0.53604400	0.26487900	1.16980600
C	0.02440500	1.64909000	-0.76789700
C	0.03188200	1.64108700	0.78445500
C	0.51531200	-0.84683600	-1.28117500
C	1.36579000	-0.99708800	-0.01871900
C	0.53050500	-0.86120400	1.26230900
C	-2.55212800	-0.81891300	0.01039700
O	2.41056700	-0.03161800	-0.08497500
H	-1.18259100	0.28714600	-2.04901300
H	-1.15820900	0.26400200	2.06429900
H	-0.62537200	2.43842000	-1.14716500
H	1.01812000	1.78685200	-1.18824400
H	-0.61678600	2.42438000	1.17799200
H	1.02895900	1.77868800	1.19703800
H	1.15473700	-0.62087200	-2.13693800
H	0.00348000	-1.79010900	-1.48958000
H	1.80649100	-2.00032400	-0.02910800
H	1.17148200	-0.64869800	2.12181900
H	0.02192400	-1.80845800	1.46549400
H	-3.38514400	-0.10240400	0.02356500
H	-2.61297600	-1.43757000	0.90664300
H	-2.62813900	-1.42225100	-0.89503400
H	3.03832200	-0.19936300	0.62684500

Sum of electronic and thermal Free Energies= -443.266695 Hartree

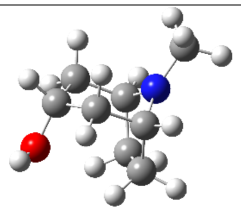

functional: ωB97X-D

Charge = 1, Multiplicity = 2

N	-1.32836700	-0.06631100	0.00345100
C	-0.54951600	0.28521000	-1.16202900
C	-0.54022500	0.27045800	1.16749400
C	0.03712600	1.65220500	-0.76770400
C	0.04091100	1.64349200	0.78582000
C	0.50869400	-0.85195200	-1.28102400
C	1.36135800	-1.00113500	-0.01732100
C	0.52020000	-0.86549400	1.26318300
C	-2.54405300	-0.81980300	0.00837700
O	2.40752100	-0.04213900	-0.08481200
H	-1.18008700	0.29829000	-2.05097900
H	-1.16497200	0.27178800	2.06075200
H	-0.60728600	2.44557600	-1.14806600
H	1.03352200	1.77989400	-1.18620300
H	-0.60418600	2.43058900	1.17800800
H	1.03876500	1.77065400	1.20140800
H	1.14529800	-0.63256700	-2.14093700
H	-0.01322400	-1.79203800	-1.48077400
H	1.79556500	-2.00802300	-0.02633300
H	1.15605900	-0.65744100	2.12785700
H	0.00394300	-1.81067800	1.45791000
H	-3.38001200	-0.11259100	0.09733700
H	-2.56446600	-1.48880700	0.87083000
H	-2.64616400	-1.37116300	-0.92667600
H	3.01868600	-0.20008600	0.63942900

Sum of electronic and thermal Free Energies= -443.379042 Hartree

SET *vs* HAT between 1b and $^1O_2$

Tropine–$^1O_2$ complex

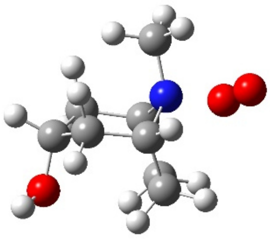

functional: CAM-B3LYP

Charge = 0, Multiplicity = 1

C	-0.04045500	0.01483100	1.26671900
C	0.26017900	0.23849100	-1.04133600
C	-0.27077100	1.50576200	0.98310000
C	-0.04782200	1.65522900	-0.54257700
C	-1.29941300	-0.84280800	1.12672200
C	-1.99309800	-0.70467400	-0.23291300
C	-0.98571600	-0.57688200	-1.38883800
C	1.31724400	-1.78506700	0.10006700
O	-2.85567600	0.43029200	-0.15285000
H	0.45944900	-0.18060000	2.21562600
H	0.99799400	0.21120200	-1.83979100
H	0.44135500	2.11254300	1.54047400
H	-1.27793200	1.79102800	1.28171400
H	0.79800600	2.31051900	-0.74654000
H	-0.92527600	2.05258000	-1.05053600
H	-2.00048200	-0.54556400	1.90978100
H	-1.05594500	-1.89186300	1.30919900
H	-2.60510800	-1.59714000	-0.40086000
H	-1.47233400	-0.09294800	-2.24036500
H	-0.70122800	-1.57262700	-1.73773500
H	1.61444400	-2.09568400	1.10107400
H	2.16864000	-1.81931100	-0.57799600
H	0.50682600	-2.40871100	-0.26267600
H	-3.36560800	0.49125900	-0.96834800
O	2.12944100	0.37598000	0.47484600
O	3.02018000	0.29121500	-0.61795300
N	0.91782200	-0.36769500	0.17271000

Sum of electronic and thermal Free Energies= -593.714501 Hartree

Tropine radical cation (see previous section)

Tropine α – amino radical

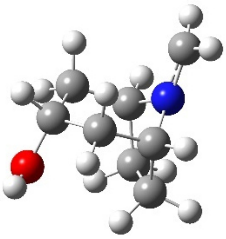

functional: CAM-B3LYP

Charge = 0, Multiplicity = 2

N	-1.45310700	-0.01308600	0.01335300
C	-0.61178200	0.28144300	-1.14741400
C	-0.58728500	0.26793300	1.15913900
C	-0.03842400	1.65782300	-0.76811300
C	-0.02151700	1.64864900	0.78321400
C	0.45657600	-0.81384100	-1.28799800
C	1.30379300	-1.00058200	-0.02558000
C	0.48177200	-0.83068300	1.26128800
C	-2.35112600	-1.05249400	0.01727200
O	2.38775000	-0.06494400	-0.09294000
H	-1.22514800	0.31615000	-2.05138800
H	-1.18096300	0.29280400	2.07650200
H	-0.69595500	2.44674500	-1.14018600
H	0.95240800	1.81269000	-1.19518300
H	-0.67058700	2.43290800	1.17922800
H	0.97823200	1.80036800	1.19078700
H	1.12119800	-0.59951200	-2.13030400
H	-0.05483600	-1.75673600	-1.50572100
H	1.72889800	-2.01172700	-0.03733300
H	1.15583600	-0.63321600	2.10247100
H	-0.02584900	-1.77735200	1.47539000
H	-2.86056300	-1.24739800	0.95511800
H	-2.88130300	-1.23658200	-0.91119300
H	2.93633600	-0.17747000	0.69101900

Sum of electronic and thermal Free Energies= -442.824686 Hartree

$O_2^{\cdot -}$

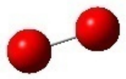

functional: CAM-B3LYP

Charge = -1, Multiplicity = 2

O	0.00000000	0.00000000	0.66353200
O	0.00000000	0.00000000	-0.66353200

Sum of electronic and thermal Free Energies= -150.438232 Hartree

$HO^{2\cdot -}$

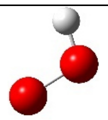

functional: CAM-B3LYP

Charge = 0, Multiplicity = 2

O	0.00000000	0.00000000	0.66353200
O	0.00000000	0.00000000	-0.66353200

Sum of electronic and thermal Free Energies= -150.889958 Hartree

14. References

1 ICSC 0088 - ACETONITRILE, https://inchem.org/documents/icsc/icsc/eics0088.htm, (accessed 15 September 2021.003Z).

2 J. A. Kent and E. R. Riegel, *Kent and Riegel's handbook of industrial chemistry and biotechnology*, Springer, New York, London, 11th edn., 2007.

3 H. H. Wasserman, R. W. DeSimone, K. R. X. Chia and M. G. Banwell, *Singlet Oxygen*, American Cancer Society, 2013.

4 a) M. L. Crossley, *J. Am. Chem. Soc.*, 1915, **37**, 2178–2181; b) E. Schwenk, *Angew. Chem.*, 1931, **44**, 912–913.

5 M. F. Acquavella, M. E. Evans, S. W. Farraher, C. J. Nevoret and C. J. Abelt, *J. Org. Chem.*, 1994, **59**, 2894–2897.

6 J. Santamaria, *Pure Appl. Chem.*, 1995, **67**, 141–147.

7 J. Santamaria, R. Ouchabane and J. Rigaudy, *Tetrahedron Lett.*, 1989, **30**, 3977–3980.

8 J. P. Barham, M. P. John and J. A. Murphy, *J. Am. Chem. Soc.*, 2016, **138**, 15482–15487.

9 C. Dai, D. R. Snead, P. Zhang and T. F. Jamison, *J Flow Chem*, 2015, **5**, 133–138.

10 D. D. D. Pham, G. F. Kelso, Y. Yang and M. T. W. Hearn, *Green Chem.*, 2012, **14**, 1189–1195.

11 E. Edink, A. Akdemir, C. Jansen, R. van Elk, O. Zuiderveld, F. J.J. de Kanter, J. E. van Muijlwijk-Koezen, A. B. Smit, R. Leurs and I. J.P. de Esch, *Bioorg. Med. Chem. Lett.*, 2012, **22**, 1448–1454.

12 X. J. Yin, C. A. Geng, X. Y. Huang, H. Chen, Y. B. Ma, X. L. Chen, C. L. Sun, T. H. Yang, J. Zhou, X. M. Zhang and J. J. Chen, *RSC Adv.*, 2016, **6**, 45059–45063.

13 V. Hassine, V. Jannet, N. Ghermani, M. Alami and S. Messaoudi, *Synthesis*, 2020, **52**, 450–458.

14 M. Kumar Parai, G. Panda, K. Srivastava and S. Kumar Puri, *Bioorganic Med. Chem. Lett.*, 2008, **18**, 776–781.

15 S. Yotphan, L. Sumunnee, D. Beukeaw, C. Buathongjan and V. Reutrakul, *Org. Biomol. Chem.*, 2015, **14**, 590–597.

16 H. Teramoto, T. Yamauchi, Y. Terado, S. Odagiri, S. Sasaki and K. Higashiyama, *Chem. Pharm. Bull.*, 2016, **64**, 410–419.

17 B. L. Laube, M. R. Asirvatham and C. K. Mann, *J. Org. Chem.*, 1977, **42**, 670–674.

18 K. Inoue, M. Yatsuzuka, T. Muramatsu, Y. Hirano and H. Inoue, *Yakugaku Zasshi*, 1988, **108**, 951–963.

19 Z. Y. Chan, P. Krishnan, S. M. Modaresi, L. W. Hii, C. W. Mai, W. M. Lim, C. O. Leong, Y. Y. Low, S. K. Wong, K. T. Yong, A. Z. X. Leong, M. K. Lee, K. N. Ting and K. H. Lim, *J. Nat. Prod.*, 2021, **84**, 2272–2282.

20 E. G. Sharova, S. F. Aripova and S. Yu. Yunusov, *Chem. Nat. Compd.*, 1980, **16**, 487–490.

21 S. Nakai, T. Yatabe, K. Suzuki, Y. Sasano, Y. Iwabuchi, J. y. Hasegawa, N. Mizuno and K. Yamaguchi, *Angew. Chem., Int. Ed.*, 2019, **58**, 16651–16659.

22 K. Bao, W. Zhang, X. Bu, Z. Song, L. Zhang and M. Cheng, *Chem. Commun.*, 2008, 5429–5431.

23 X. Tian, T. A. Karl, S. Reiter, S. Yakubov, R. de Vivie-Riedle, B. König and J. P. Barham, *Angew. Chem. Int. Ed.*, 2021, **60**, 20817–20825.

24 A. A. Abdel-Shafi, D. R. Worrall and A. Y. Ershov, *Dalton Trans.*, 2004, 30–36.

25 J. P. Barham, M. P. John and J. A. Murphy, *Beilstein J. Org. Chem.*, 2014, **10**, 2981–2988.

26 S. Blanc, T. Pigot, C. Cugnet, R. Brown and Sylvie Lacombe, *Phys. Chem. Chem. Phys.*, 2010, **12**, 11280–11290.

27 R. Pavela, G. Benelli, R. Petrelli, L. Cappellacci, G. Lupidi, S. Sut, S. Dall'Acqua and F. Maggi, *Molecules*, 2019, **24**, p. 879.

28 E. Baciocchi, T. Del Giacco and A. Lapi, *Org. Lett.*, 2004, **6**, 4791–4794.

29 I. Saito, T. Matsuura and K. Inoue, *J. Am. Chem. Soc.*, 1981, **103**, 188–190.

30 a) *SCALE3ABS, CrysAlisPro, Aglient Technologies Inc*, Oxford and GB, 2015; b) G. M. Sheldrick, *SADABS, Bruker AXS,*, Madison and USA, 2007.

31 R. C. Clark and J. S. Reid, *Acta Crystallogr A Found Crystallogr*, 1995, **51**, 887–897.

32 G. M. Sheldrick, *Acta Crystallogr A Found Adv*, 2015, **71**, 3–8.

33 O. V. Dolomanov, L. J. Bourhis, R. J. Gildea, J. A. K. Howard and H. Puschmann, *J. Appl. Crystallogr.*, 2009, **42**, 339–341.

34 G. M. Sheldrick, *Acta Crystallogr A Found Crystallogr*, 2008, **64**, 112–122.

35 Diamond Version 4., *Crystal Impact GbR, Bonn*, 2010.

36 a) V. Barone and M. Cossi, *J. Phys. Chem. A*, 1998, **102**, 1995–2001; b) W. Kohn and L. J. Sham, *Phys. Rev.*, 1965, **140**, A1133.

37 Gaussian 09, Revision D.01, M. J. Frisch, G. W. Trucks, H. B. Schlegel, G. E. Scuseria, M. A. Robb, J. R. Cheeseman, G. Scalmani, V. Barone, G. A. Petersson, H. Nakatsuji, X. Li, M. Caricato, A. Marenich, J. Bloino, B. G. Janesko, R. Gomperts, B. Mennucci, H. P. Hratchian, J. V. Ortiz, A. F. Izmaylov, J. L. Sonnenberg, D. Williams-Young, Young, F. Ding, F. Lipparini, F. Egidi, J. Goings, B. Peng, A. Petrone, T. Henderson, D. Ranasinghe, V. G. Zakrzewski, J. Gao, N. Rega, G. Zheng, W. Liang, M. Hada, M. Ehara, K. Toyota, R. Fukuda, J. Hasegawa, M. Ishida, T. Nakajima, Y. Honda, O. Kitao, H. Nakai, T. Vreven, K. Throssell, J. A. Montgomery, Jr., J. E. Peralta, F. Ogliaro, M. Bearpark, J. J. Heyd, E. Brothers, K. N. Kudin, V. N. Staroverov, T. Keith, R. Kobayashi, J. Normand, K. Raghavachari, A. Rendell, J. C. Burant, S. S. Iyengar, J. Tomasi, M. Cossi, J. M. Millam, M. Klene, C. Adamo, R. Cammi, J. W. Ochterski, R. L. Martin, K. Morokuma, O. Farkas, J. B. Foresman, and D. J. Fox, Gaussian Inc., Wallingford CT, 2, 2016.

38 T. Yanai, D. P. Tew and N. C. Handy, *Chemical Physics Letters*, 2004, **393**, 51–57.

39 J. D. Chai and M. H. Gordon, *Phys. Chem. Chem. Phys.*, 2008, **10**, 6615–6620.

40 a) R. Krishnan, J. S. Binkley, R. Seeger and J. A. Pople, *J. Chem. Phys.*, 1980, **72**, 650–654; b) M. J. Frisch, J. A. Pople and J. S. Binkley, *J. Chem. Phys.*, 1984, **80**, 3265–3269; c) A. D. McLean and G. S. Chandler, *J. Chem. Phys.*, 1980, **72**, 5639–5648.

41 M. Cossi, N. Rega, G. Scalmani and V. Barone, *J. Comput. Chem.*, 2003, **24**.

42 C. Adamo and D. Jacquemin, *Chem. Soc. Rev.*, 2013, **42**, 845–856.

43 M. Neumeier, D. Sampedro, M. Májek, V. A. D. O'Shea, A. J. von Wangelin and R. Pérez-Ruiz, *Chem. Eur. J.*, 2018, **24**, 105–108.

44 E. Runge and E. K. U. Gross, *Phys. Rev. Lett.*, 1984, **52**, 997–1000.

45 C. Y. Cheng, M. S. Ryley, M. J.G. Peach, D. J. Tozer, T. Helgaker and A. M. Teale, *Molecular Physics*, 2015, **113**, 1937–1951.

46 J. Phan, S. M. Ruser, K. Zeitler and J. Rehbein, *Eur. J. Org. Chem.*, 2019, **2019**, 557–561.

47 L. G. Gagliardi, C. B. Castells, C. Ràfols, M. Rosés and E. Bosch, *J. of Chem. & Eng. Data*, 2007, **52**, 1103–1107.

48 J. E. Saunders, C. Sanders, H. Chen and H. P. Loock, *Appl. Opt.*, 2016, **55**, 947–953.

49 M. Majek, U. Faltermeier, B. Dick, R. P. Ruiz and A. J. von Wangelin, *Chem. Eur. J.*, 2015, **21**, 15496–15501.
